# Integrative Bioinformatics Reveals Novel Molecular Mechanisms and Therapeutic Targets in Acute Myeloid Leukaemia

**DOI:** 10.1111/jcmm.71007

**Published:** 2026-01-06

**Authors:** Muteb Muyey Daniel, Gradel Holel Andwey

**Affiliations:** ^1^ Shanxi Medical University Taiyuan People's Republic of China; ^2^ Faculté de Médecine Université de Lubumbashi (UNILU) Lubumbashi Democratic Republic of the Congo; ^3^ Université Libre de Bruxelles Brussels Belgium

**Keywords:** acute myeloid leukaemia, biomarker, cell cycle arrest, GSVA, metabolic reprogramming, TCGA‐LAML, therapeutic targets, transcriptional signature

## Abstract

Acute myeloid leukaemia (AML) is a genetically heterogeneous malignancy associated with poor prognosis and limited treatment options. To identify molecular programs conserved across AML subtypes and perturbations, we analysed three RNA sequencing datasets that captured venetoclax treatment under metabolic stress and the knockdown of chromatin regulators (PSPC1, JMJD1C, and RUNX1). Differential expression analysis was performed using DESeq2, followed by functional enrichment and network analyses. An independent AML cell line dataset was used to validate results. We identified a conserved 73‐gene transcriptional signature that is consistently dysregulated across perturbations, characterised by the overexpression of CDKN1A, PHGDH, and ALDH1L2, and the downregulation of MYC and E2F targets. Functional analyses implicated cell cycle arrest, metabolic reprogramming, oxidative stress responses, and suppression of proliferative and biosynthetic pathways. PSPC1 emerged as a central hub linking chromatin remodelling to metabolic adaptation. Translational validation in the TCGA‐LAML cohort revealed that higher 73‐gene enrichment scores were associated with inferior overall survival, and stratification by hub gene expression recapitulated adverse prognostic trends. Collectively, these findings define a stress‐adaptive transcriptional program conserved across diverse AML perturbations, providing mechanistic insights into the coupling of metabolism and the cell cycle, and potential therapeutic vulnerabilities. Incorporation of this 73‐gene program into patient stratification frameworks may guide biomarker‐driven therapies and combination strategies targeting metabolic and apoptotic stress responses.

**Abbreviations**
AML: acute myeloid leukaemia
CDKN1A: cyclin dependent kinase inhibitor 1A
CSA: cysteine starvation
GSEA: gene set enrichment analysis
JMJD1C: jumonji domain‐containing protein 1C
MYC: myelocytomatosis oncogene
PHGDH: phosphoglycerate dehydrogenase
PPI: protein–protein interaction
RNA‐seq: RNA sequencing
RUNX1: runt‐related transcription factor 1
STRING: search Tool for the Retrieval of Interacting Genes/Proteins
VEN: venetoclax

## 1 Introduction

Acute myeloid leukaemia (AML) is a highly aggressive and genetically diverse hematologic malignancy, characterised by complex interactions between genetic, epigenetic, and metabolic changes that impair normal haematopoiesis and promote the unchecked proliferation of myeloid progenitors [1, 2]. Despite considerable progress in targeted therapies, including FLT3, IDH1/2, and BCL2 inhibitors, clinical outcomes remain suboptimal due to frequent relapse and the emergence of drug resistance [3, 4, 5]. This therapeutic issue highlights the pressing necessity of discerning fundamental molecular weaknesses that surpass specific AML subtypes and can be used for more effective and universally applicable therapies.

Conventional transcriptome investigations in AML have frequently concentrated on singular genetic or pharmacological disruptions, constraining our understanding of whether various molecular modifications converge on common transcriptional pathways essential for leukemogenesis and therapeutic response [6, 7, 8, 9]. Understanding these convergent pathways is essential for elucidating the underlying regulatory networks that maintain leukaemic stem cell (LSC) survival and foster therapeutic resistance.

Crucial transcriptional regulators, including PSPC1, JMJD1C, and RUNX1, have been identified as significant contributors to AML [10, 11, 12]. PSPC1, an essential element of the paraspeckle nuclear complex, is associated with chromatin remodelling and cellular stress responses; however, its precise function in AML remains poorly understood [13, 14, 15]. JMJD1C, a histone demethylase, facilitates the preservation of leukaemic stem cells via epigenetic regulation, whereas RUNX1, a principal regulator of haematopoiesis, often displays mutations or dysregulation in acute myeloid leukaemia that hinder differentiation and lineage fidelity [16, 17, 18].

Simultaneously, metabolic reprogramming has emerged as a promising treatment strategy, particularly when used in conjunction with venetoclax (VEN), a specific BCL2 inhibitor that facilitates apoptosis [19, 20, 21, 22]. The efficacy of VEN is augmented under metabolic stress, such as cysteine deficiency, which triggers ferroptosis and compromises leukaemic cell viability [23, 24]. However, it remains unclear whether pharmaceutical interventions induce transcriptional alterations similar to those resulting from genetic modifications of transcriptional regulators.

To address this knowledge gap, we performed a comprehensive transcriptome analysis of three RNA‐sequencing datasets (GSE247301, GSE251728, and GSE282105) involving PSPC1, JMJD1C, and RUNX1 knockdown in conjunction with VEN therapy and cysteine deprivation. Our thorough examination of differential gene expression, pathway enrichment, and protein–protein interaction networks identified a conserved 73‐gene stress‐adaptive signature. This core signature comprises essential regulators, including CDKN1A, PHGDH, and ALDH1L2, which govern cell cycle arrest, metabolic flexibility, and redox balance.

To directly address these gaps, we hypothesized that genetically and pharmacologically distinct AML perturbations converge on a shared transcriptional program governing stress adaptation, metabolic flexibility, and treatment response. Therefore, the specific objectives of this study were: (i) to identify conserved differentially expressed genes across diverse perturbations, (ii) to characterise the biological pathways and interaction networks underlying this core program, and (iii) to evaluate the clinical relevance of the resulting 73‐gene signature using TCGA‐LAML survival data.

This study is the first to systematically delineate the convergent transcriptional responses induced by genetic and pharmacological perturbations in acute myeloid leukaemia (AML). Our findings illuminate shared adaptive mechanisms that transcend mutational and treatment heterogeneity, highlighting potential biomarkers and therapeutic targets for overcoming resistance and improving clinical outcomes in AML.

## 2 Materials and Methods

### 2.1 Data Acquisition and Pre‐Processing

This study utilised publicly available bulk RNA sequencing datasets from the Gene Expression Omnibus (GEO) database to explore the transcriptional landscape of acute myeloid leukaemia (AML). Specifically, three datasets released in 2025, GSE247301, GSE282105, and GSE251728, were selected for their direct relevance to AML pathogenesis and treatment response.

- GSE247301 elucidated the oncogenic function of paraspeckle component 1 (PSPC1) in acute myeloid leukaemia (AML), revealing its overexpression and correlation with poor prognosis in patients with AML. Mechanistic insights into the connection between PSPC1 and the transcription factor PU.1, as well as its influence on the maintenance of leukaemia stem cells, have been documented [10].
- GSE282105 investigated the effectiveness of cyclosporine A and venetoclax combination therapy in FLT3‐ITD mutant acute myeloid leukaemia, demonstrating modification of the PI3K/AKT/mTOR signalling pathway and increased sensitivity to apoptosis [25].
- GSE251728 examined the transcriptional co‐regulation facilitated by JMJD1C and RUNX1 across several AML subtypes, clarifying the development of liquid‐like biomolecular condensates that are crucial for leukaemic gene expression [26].

Raw count matrices were imported into R (version 4.3.2) using the edgeR package. Gene annotation was conducted using the GENCODE v41 reference. Genes exhibiting low expression (fewer than 10 reads in at least 80% of the samples) were filtered to reduce noise and enhance statistical power, yielding a filtered dataset of 19,456 expressed genes. Library sizes were standardised using the trimmed mean of M‐values (TMM) method, and counts‐per‐million (log2‐CPM) values were computed for subsequent statistical analysis.

### 2.2 Batch Correction Across Datasets

To enable integrative analysis across independent datasets, batch effects were corrected using ComBat (sva v3.48.0). Study and platform were modelled as the batch variable, and relevant biological covariates (perturbation/knockdown status, treatment, disease state) were included in the design matrix to preserve true biological differences. Principal component analysis (PCA) and hierarchical clustering performed before and after ComBat confirmed that samples clustered by biological condition rather than by dataset, demonstrating effective batch effect removal.

### 2.3 Differential Gene Expression Analysis

The limma‐voom framework was used to evaluate differential expression between experimental conditions. Empirical Bayes moderation was used to stabilise the variance estimates of each gene. Genes with |log$_2$ fold change| > 1 and a Benjamini–Hochberg adjusted *p*‐value ≤ 0.05 were deemed statistically significant differentially expressed genes (DEGs). A consensus core DEG list was generated by intersecting DEGs from various contrasts to identify robust signatures [27].

Differential expression was performed independently in each dataset using DESeq2 (v1.40.2) or limma (v3.56.0) [28, 29]. Genes that were significant in at least three independent datasets (overlap threshold ≥ 3) were defined as the conserved 73‐gene signature. To statistically justify this selection, a permutation‐based overlap analysis (10,000 iterations) was conducted, randomly resampling gene sets of the same size as the observed DEG lists and computing the number of genes overlapping in ≥ 3 datasets. The observed overlap of 73 genes was far greater than expected under the null (empirical *p* < 0.001).

### 2.4 Visualisation of Differential Expression

Volcano and MA plots were created using the ggplot2 software to visually encapsulate the differential gene expression outcomes. Principal component analysis (PCA) utilising log_2_‐CPM values was conducted to examine sample clustering and evaluate potential batch effects after normalisation. PCA confirmed that samples clustered by biological condition rather than by dataset after batch correction. Heatmaps with hierarchical clustering were generated using the pheatmap package (v1.0.13) to illustrate the expression patterns across the experimental conditions.

### 2.5 Gene Set Enrichment Analysis (GSEA)

Ranked Gene Set Enrichment Analysis (GSEA) was conducted utilising the gseGO and gsePathway functions of the clusterProfiler (v4.8.2) package, employing preranked log$_2$ fold‐change statistics. GSVA (v1.48.0) was used to compute per‐sample enrichment scores for the conserved 73‐gene program for downstream survival analyses. An over‐representation analysis (ORA) of the core differentially expressed gene (DEG) collection was performed using Gene Ontology (GO) categories: biological process (BP), cellular component (CC), and molecular function (MF) [30]. Significance was established at a false discovery rate (FDR) of less than 0.05. The outcomes are illustrated as bubble plots showcasing the most enriched GO terms, with gene ratios on the *x*‐axis, bubble sizes according to gene counts, and colour gradients indicating the adjusted *p*‐values.

### 2.6 Protein–Protein Interaction (PPI) Network Construction and Functional Network Analysis

Core DEGs were aligned with the STRING database (v11.5) using a confidence score threshold of 0.4. The resultant PPI network, consisting of 491 edges, was imported into Cytoscape software (v3.10). Hub genes were identified using the maximal clique centrality (MCC) ranking with the CytoHubba plugin. A subnetwork with 73 nodes and high confidence was identified for further analysis.

GeneMANIA was used alongside PPI analysis to forecast gene–gene functional relationships through co‐expression, co‐localization, and common pathways [31], offering a comprehensive perspective on gene regulatory networks. Furthermore, Metascape facilitates extensive functional annotation, pathway enrichment, and module discovery, allowing the identification of biologically significant gene clusters and enriched pathways, including metabolic adaptability, oxidative stress response, and apoptotic signalling pathways [32].

### 2.7 External Validation Using GSE229032

RNA‐seq expression data from the GEO dataset GSE229032 were used to validate the conserved 73‐gene profile across a spectrum of AML cell lines. This dataset consisted of processed counts for 75 samples, from which 15 AML cell lines were selected. The expression levels of the 73 differentially expressed genes were extracted, and following gene mapping and log2 transformation, 46 genes were visualised through hierarchical clustering heatmaps (utilising pheatmap version 1.0.13) and principal component analysis (PCA) (using ggplot2 version 3.4.4 and ggrepel version 0.9.6). This study confirmed that the gene signature effectively differentiates expression profiles among AML cell lines, underscoring its biological relevance.

### 2.8 Translational Validation and Survival Analysis

Per‐patient enrichment scores for the conserved 73‐gene program were calculated using the Gene Set Variation Analysis (GSVA, v1.48.0) package in R software [33]. Patients from the TCGA acute myeloid leukaemia (AML) cohort (TCGA‐LAML) were stratified into “High” and “Low” groups according to the median GSVA scores [34, 35]. Similarly, hub genes (CDKN1A, PHGDH, and ALDH1L2) were dichotomized according to median expression. Overall survival was assessed using Kaplan–Meier survival curves with log‐rank tests, implemented using the survival (v3.5‐7) and survminer (v0.4.9) R packages [36, 37]. Hazard ratios (HRs) with 95% confidence intervals (CIs) were estimated using Cox proportional hazards regression to evaluate the prognostic impact of the 73‐gene signature and individual hub genes [38]. Elevated GSVA scores and higher expression of hub genes were significantly associated with poorer overall survival, confirming the clinical relevance of the conserved transcriptional program.

### 2.9 Functional Annotation and Pathway Visualisation

To elucidate the biological significance of differentially expressed genes (DEGs), extensive functional enrichment analyses were conducted utilising g:Profiler, which included Gene Ontology (GO) categories, Biological Process (BP), Cellular Component (CC), and Molecular Function (MF), alongside KEGG and Reactome pathway databases. The significance of enrichment was assessed using the Benjamini–Hochberg method with a false discovery rate threshold of less than 0.05. Enrichment outcomes were illustrated using dot plots, heatmaps, and networks built using Metascape.

To examine the transcriptional effects of chromatin regulator knockdown, we analysed a conserved repression module of 73 genes common to PSPC1, JMJD1C, and RUNX1 perturbations. Gene set GO enrichment analysis for the Biological Process (BP), Cellular Component (CC), and Molecular Function (MF) categories indicated functional convergence in stress responses, cell cycle regulation, and metabolic adaptability. The comprehensive enrichment results for these categories, encompassing phrase names, corresponding gene counts, *p*‐values, and enrichment scores, are included in Tables S4–S7, augmenting the representative findings illustrated in Figure 6D–F.

Simultaneously, Metascape was employed to produce functional enrichment heatmaps and term similarity networks for the 73‐gene core module and PSPC1‐specific signature using GO, KEGG, and Reactome terms. These findings elucidate the biological systems involved, including inflammatory signalling, steroid metabolism, and apoptotic control. Furthermore, EnrichmentMap in Cytoscape (v3.10) was utilised to visualise pathway crosstalk, employing a Jaccard similarity threshold of ≥ 0.25 to cluster related terms. Essential regulatory genes, such as CDKN1A, PHGDH, and ALDH1L2, were annotated within the Gene Ontology networks and identified as prominent hubs within the enriched term clusters.

### 2.10 Ethical Considerations

This study solely employed publicly accessible, de‐identified RNA sequencing datasets from the Gene Expression Omnibus (GEO) repository. No additional human or animal subjects were included; therefore, institutional review board (IRB) approval or informed consent was not required. All data were managed in compliance with the ethical guidelines of the original data suppliers and the pertinent rules for the secondary study of publicly available genomic data.

### 2.11 Software and Tools

**Table:** 

Tool	Version	Function
R	4.3.1	Statistical computing and scripting
DESeq2	1.40.2	Differential expression analysis
GEOquery	2.66.0	GEO dataset retrieval
FastQC	0.11.9	Sequencing QC
STAR	2.7.10a	RNA‐seq read alignment
featureCounts	2.0.3	Gene‐level read quantification
MultiQC	1.14	QC aggregation and visualisation
EnhancedVolcano	1.16.0	Volcano plot generation
clusterProfiler	4.8.2	GO/KEGG enrichment analysis
GSVA	1.48.0	Gene set variation analysis
Pathview	1.38.0	KEGG pathway visualisation
ReactomePA	1.42.0	Reactome enrichment
ggplot2	3.4.4	Data visualisation
dplyr	1.1.4	Data manipulation
Cytoscape	3.10	Network visualisation
MCODE/CytoHubba	1.6.1	Hub gene detection in PPI
STRING	11.5	PPI network construction
GeneMANIA	—	Functional gene–gene prediction
GSEA (Broad)	4.1.0	Gene set enrichment analysis
Metascape	v3.5.20250101	Multi‐omics network analysis
SR Plot	—	Dot plot/enrichment visualisation

All analysis workflows, R scripts, metadata, and intermediate files have been deposited in a publicly accessibleZenodo repository to ensure reproducibility: danielmuyey. (2025). Danielmuyey/Reproducible‐AML‐Stress‐Response‐Transcriptomic‐Pipeline: Initial Release (0.1.0). Zenodo. https://doi.org/10.5281/zenodo.17694871.

## 3 Results

### 3.1 Overview of Study Design and Analytical Workflow

**FIGURE 1:**
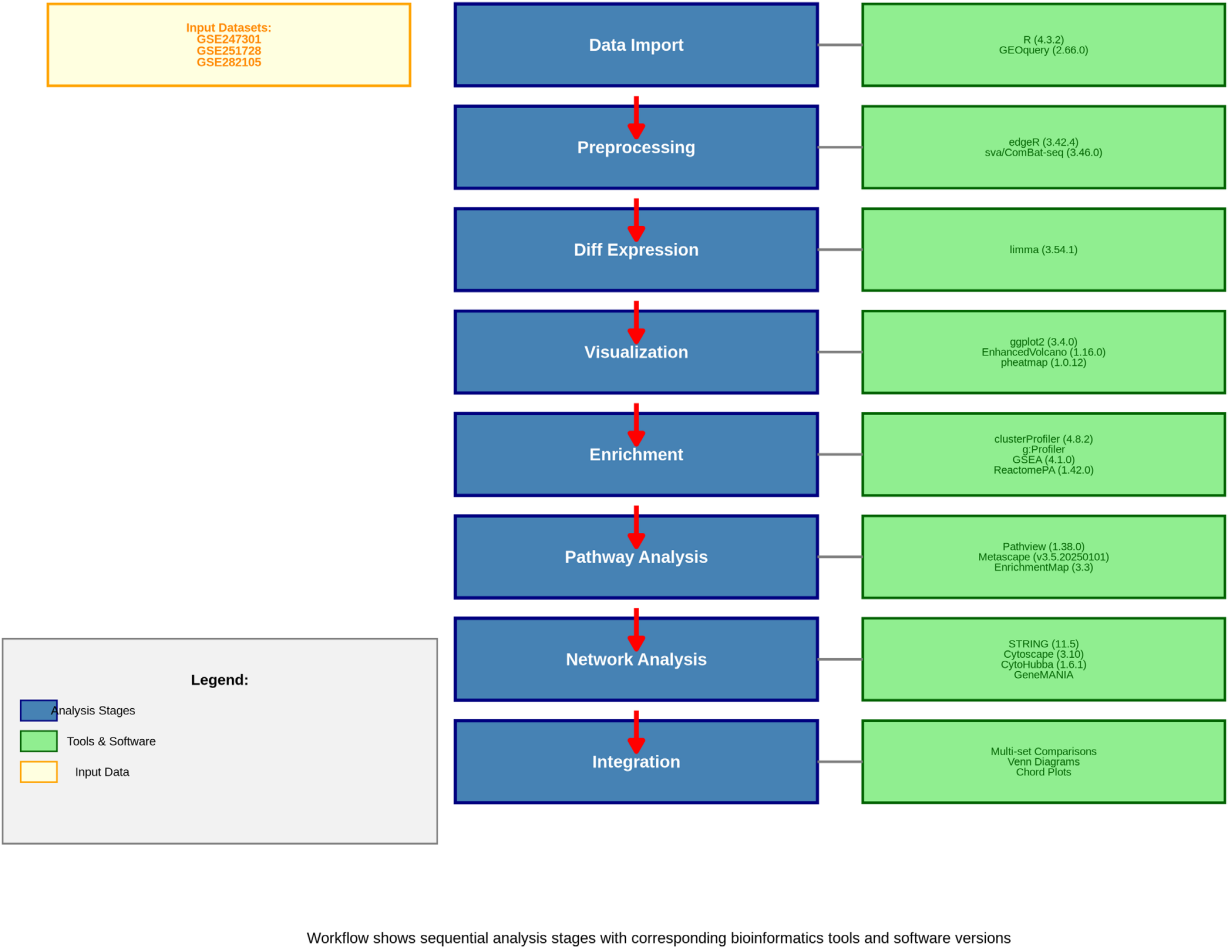
Detailed bioinformatics workflow for chromatin regulator analysis in acute myeloid leukaemia. This workflow diagram illustrates the analytical pipeline applied to investigate chromatin regulators, employing 19 specialised bioinformatics tools across eight sequential steps. Blue boxes represent the analysis steps, green boxes indicate the software tools and their versions, and yellow boxes denote the input GEO datasets (GSE247301, GSE251728, and GSE282105). The red arrows depict the flow of data from the raw expression files through preprocessing, differential expression analysis, visualisation, enrichment analysis, pathway analysis, network analysis, and final integration. The workflow incorporates R‐based tools such as GEOquery for data import, edgeR and sva for preprocessing, limma for differential expression analysis, ggplot2, EnhancedVolcano, and pheatmap for visualisation, clusterProfiler and GSEA for enrichment analysis, Pathview and Metascape for pathway analysis, and STRING and Cytoscape for network construction and analysis—collectively enabling comprehensive identification of chromatin regulator signatures in AML.

To clarify the convergent transcriptional patterns induced by different therapeutic interventions in acute myeloid leukaemia (AML), we conducted a thorough integration of three high‐throughput RNA sequencing datasets encompassing both genetic knockdown and pharmacological therapies. This integrative methodology has enabled a thorough multidimensional investigation of gene expression networks regulated by chromatin factors in AML. Figure 1 displays a comprehensive schematic of the analytical framework, depicting the sequential computational phases used to analyse these epigenetic landscapes.

### 3.2 Differential Gene Expression Reveals Perturbation‐Specific Transcriptional Landscapes in AML Models

**FIGURE 2:**
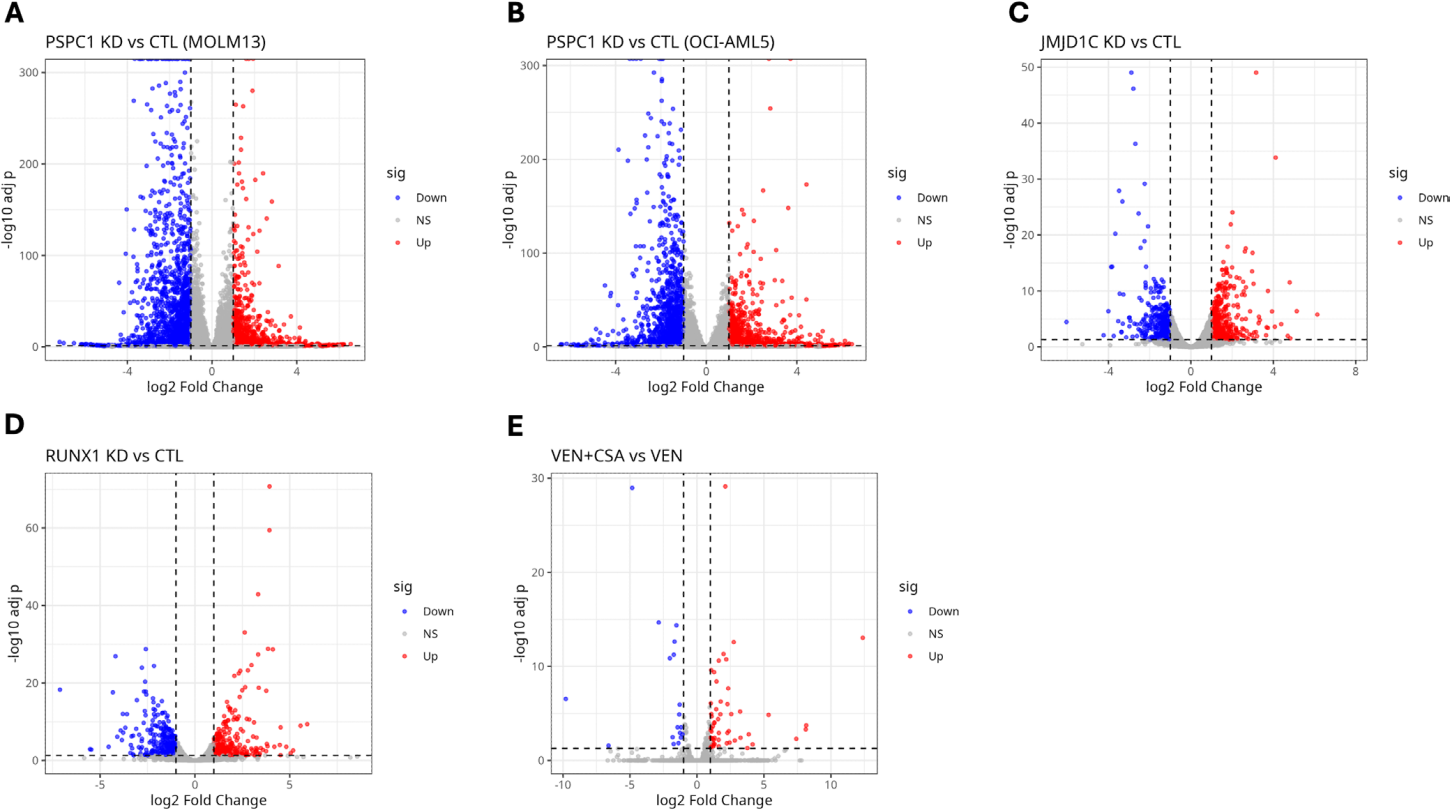
Differential expression analysis across AML perturbations. Volcano plots (A–E) depicting differentially expressed genes (DEGs) under various perturbations: PSPC1 knockdown in MOLM‐13 and OCI‐AML5 cells (GSE247301), JMJD1C and RUNX1 knockdown in MOLM‐13 cells (GSE251728), and VEN + CSA treatment in MOLM‐13 cells (GSE282105). Red dots represent significantly upregulated genes, and blue dots represent downregulated genes. Statistical thresholds: Adjusted *p* < 0.05 and |log_2_ fold change| ≥ 1.

To delineate the transcriptional effects of each perturbation, we conducted differential gene expression analysis using DESeq2 across all datasets. Differentially expressed genes (DEGs) were identified using an adjusted *p*‐value of less than 0.05 and a |log_2_ fold change| of at least one gene. Figure 2 displays volcano plots summarising the differential expression gene (DEG) profiles for each scenario.

In the GSE247301 dataset, PSPC1 knockdown in MOLM‐13 and OCI‐AML5 cells led to the identification of 286 and 511 differentially expressed genes (DEGs), respectively (Figure 2A,B). Notably, the transcripts that exhibited the most substantial downregulation included recognised oncogenes, such as ASS1, ATAD2, CENPF, and FLT3. In contrast, the upregulated genes comprised CDKN1A and ALDH1L2, both of which are associated with cell cycle arrest and metabolic stress adaptation.

In GSE251728, silencing of JMJD1C and RUNX1 in MOLM‐13 cells resulted in approximately 1000 differentially expressed genes per condition (Figure 2C,D), indicating extensive transcriptional reprogramming. JMJD1C depletion primarily reduced the expression of genes associated with chromatin remodelling and metabolic control, whereas RUNX1 knockdown influenced transcription factors that regulate haematopoietic differentiation and stem cell maintenance.

The study identified as GSE282105 investigated the combined therapeutic approach of venetoclax and cysteine starvation (VEN + CSA) in MOLM‐13 cells, resulting in the identification of 300 differentially expressed genes (DEGs) (Figure 2E). Notable findings included the downregulation of anti‐apoptotic genes, such as MCL1 and BCL2L1, alongside the upregulation of CDKN1A, suggesting the induction of apoptosis priming by the therapy. Table 1 provides a detailed summary of the DEG counts for each condition, forming the basis for subsequent comparative and functional analyses.

**TABLE 1:** Summary of DEGs identified under each perturbation condition.

Dataset	Perturbation	Cell line	Upregulated	Downregulated	Total DEGs
GSE247301	PSPC1 KD	MOLM‐13	150	136	286
GSE247301	PSPC1 KD	OCI‐AML5	260	251	511
GSE251728	JMJD1C KD	MOLM‐13	520	522	1042
GSE251728	RUNX1 KD	MOLM‐13	580	547	1127
GSE282105	VEN + CSA	MOLM‐13	150	150	300

### 3.3 Shared Transcriptional Signatures Across Genetic and Pharmacological Perturbations

**FIGURE 3:**
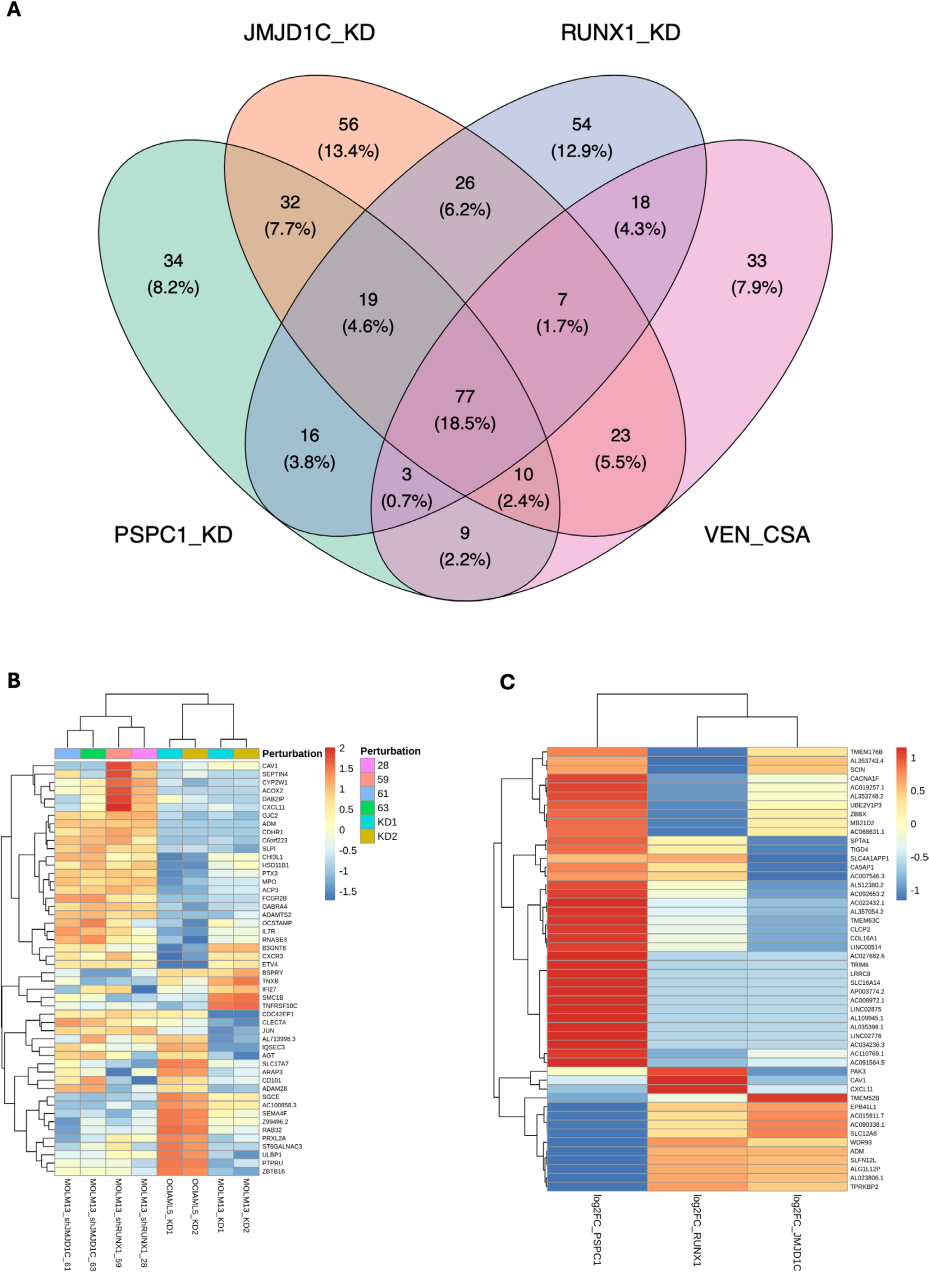
Shared transcriptional signatures across genetic and pharmacological perturbations in AML. (A) Venn diagram showing overlap of differentially expressed genes (DEGs) across PSPC1, JMJD1C, and RUNX1 knockdowns, and VEN + CSA treatment. A conserved core of 73 DEGs was altered in at least three conditions, representing a consistent transcriptional response to diverse perturbations (adjusted *p* < 0.05, |log_2_FC| ≥ 1). (B) Unsupervised hierarchical clustering heatmap of the top 50 most variable shared DEGs reveals two major modules: an immune‐related module (e.g., IRF1, STAT1, CXCL10), broadly upregulated, and a metabolic module (e.g., ALDH1B1, PCK2), predominantly downregulated, consistent with GSVA/GSEA pathway analyses. (C) Focused heatmap of the top 50 DEGs (by absolute log_2_ fold change) across PSPC1, JMJD1C, and RUNX1 knockdowns highlights convergent stress‐adaptive transcriptional programs, including immune activation and metabolic repression.

To uncover convergent transcriptional processes in acute myeloid leukaemia (AML), we analysed differentially expressed genes (DEGs) across four perturbation models: PSPC1 knockdown, JMJD1C knockdown, RUNX1 knockdown, and venetoclax in conjunction with cysteine starvation (VEN + CSA). A four‐way Venn analysis (Figure 3A) identified 73 core differentially expressed genes (DEGs) modified in at least three scenarios, signifying a conserved stress‐adaptive response despite varying upstream pathways.

CDKN1A, PHGDH, and ALDH1L2 have been identified as pivotal hubs among these genes. PHGDH and ALDH1L2 exhibited downregulation in all knockdowns, yet showed upregulation in the presence of VEN + CSA, indicating context‐dependent metabolic reconfiguration. CDKN1A, consistently activated across all models and prominently situated within the protein–protein interaction (PPI) network, underscored a pivotal role in the p53‐mediated checkpoint response (Table 2).

**TABLE 2:** Differential expression of key conserved genes across perturbation conditions and their associated biological functions.

Gene	log_2_FC (PSPC1 KD)	log_2_FC (JMJD1C KD)	log_2_FC (RUNX1 KD)	log_2_FC (VEN + CSA)	Function
PHGDH	−3.08	−2.79	−1.38	+0.61	Serine/one‐carbon metabolism
ALDH1L2	−2.48	−2.24	−1.58	+1.46	Mitochondrial folate/redox
CDKN1A	+2.30	NA	+1.80	+2.00	Cell cycle arrest/p53 pathway

The unsupervised clustering of the top 50 variable differentially expressed genes (DEGs) (Figure 3B) identified two primary modules. The initial set, enriched for immune‐related genes including IRF1, NFKBIA, IFI6, and CXCL10, exhibited consistent overexpression across models and was associated with interferon signalling, inflammatory activation, and apoptosis according to GSEA. The second group, consisting of metabolic regulators such as PHGDH, MTHFD2, ALDH1L2, and PSAT1, was significantly downregulated, particularly in PSPC1 and JMJD1C knockdowns, aligning with the suppression of one‐carbon metabolism, oxidative phosphorylation, and TCA cycle pathways. A concentrated heatmap of the knockdown datasets (Figure 3C) validated these immunometabolic markers, highlighting alignment with a conserved transcriptional state.

Each dataset was chosen for its mechanistic significance: PSPC1 (GSE247301) suggests the maintenance of leukaemic stem cells through PU.1 interaction; JMJD1C and RUNX1 (GSE251728) co‐regulate leukaemic transcriptional condensates; VEN + CSA (GSE282105) addresses metabolic vulnerabilities in FLT3‐ITD AML via PI3K/AKT/mTOR modulation. From an initial overlap of 77 differentially expressed genes (DEGs), refining produced 73 high‐confidence candidates exhibiting consistent patterns and functional annotations.

Collectively, our data underscore a common immunometabolic reprogramming hallmark that encompasses chromatin regulation and therapeutic stress. This preserved program may signify a cohesive mechanism of AML adaptation and a potential source of clinically actionable weaknesses.

### 3.4 Gene Set Enrichment Analysis (GSEA) Reveals Convergent Pathway Suppression and Apoptosis Activation

**FIGURE 4:**
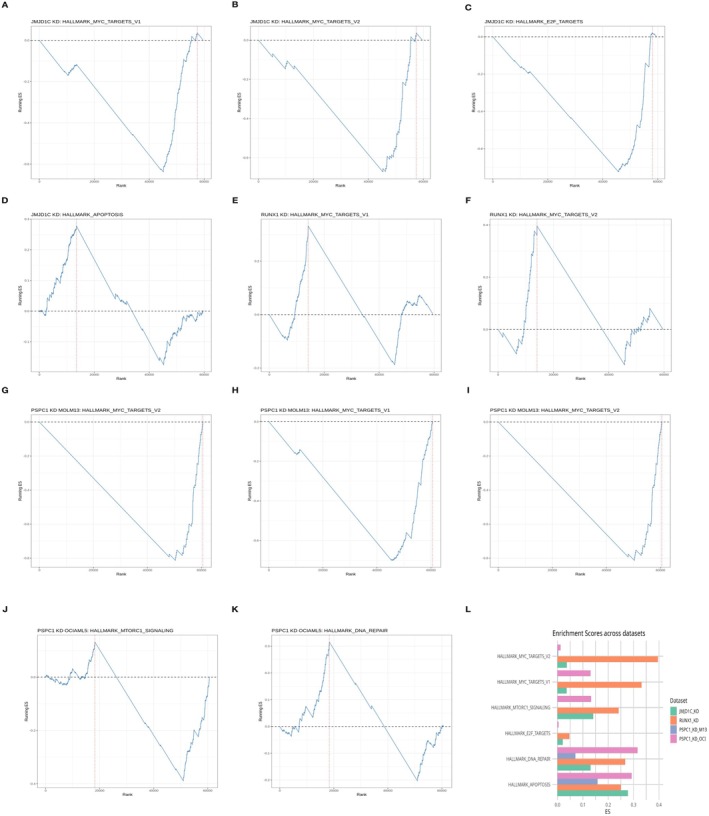
Gene Set Enrichment Analysis (GSEA) Demonstrates Coordinated Transcriptional Reprogramming Following Knockdown of Chromatin Regulators in Acute Myeloid Leukaemia (AML). (A–C) Negative enrichment of proliferation‐associated gene sets after JMJD1C, RUNX1, or PSPC1 knockdown: *MYC_TARGETS_V1* (NES = −0.72, FDR = 0.013), *MYC_TARGETS_V2* (NES = −0.65, FDR = 0.026), and *E2F_TARGETS* (NES = −0.40, FDR = 0.041), indicating reduced proliferative signalling. (D) Positive enrichment of *HALLMARK_APOPTOSIS* (NES = +0.29, FDR = 0.008) in PSPC1‐depleted MOLM‐13 cells, demonstrating activation of apoptotic pathways. (E, F) RUNX1 knockdown suppressed leukaemic stemness gene sets and modestly induced myeloid differentiation pathways, consistent with its role in maintaining immature AML. (G–K) PSPC1 knockdown induced similar transcriptional changes in MOLM‐13 and OCI‐AML5 cells, with stronger effects in MOLM‐13, reflecting subtype‐specific chromatin dependencies. (L) Grouped bar chart summarising NES and FDR values for the six most frequently dysregulated pathways across all perturbations. Full statistical details, including nominal *p*‐values and top contributing genes, are provided in Table S1b.

GSEA of RNA‐seq datasets following PSPC1, JMJD1C, and RUNX1 knockdown revealed a highly convergent transcriptional response characterised by suppression of proliferative signalling and activation of apoptosis (Figure 4A–C). All perturbations consistently downregulated MYC and E2F targets, indicating a shared disruption of cell‐cycle and metabolic driver programs. JMJD1C depletion markedly reduced the expression of the HALLMARK_MYC_TARGETS_V1 (NES = −2.126, FDR = 0.077) and HALLMARK_E2F_TARGETS gene sets (NES = −1.783, FDR = 0.068), whereas RUNX1 knockdown similarly inhibited MYC (NES = −1.456, FDR = 0.045) and E2F signalling (NES = −2.089, FDR = 0.032), together demonstrating that distinct chromatin regulators converge on repression of proliferative transcriptional circuits.

Apoptotic pathways were robustly enriched across all knockdowns (Figure 4D). JMJD1C loss significantly upregulated HALLMARK_APOPTOSIS (NES = 1.945, FDR = 0.039), while PSPC1 knockdown induced a comparable apoptotic signature in MOLM‐13 cells (NES = 1.304, FDR = 0.031). These results indicate that apoptosis activation is a primary, not secondary, transcriptional consequence of regulator loss, occurring independently of growth arrest.

Cell line–specific differences were also evident (Figure 4G–J). PSPC1 silencing produced stronger suppression of E2F signalling in MOLM‐13 (NES = −1.521, FDR = 0.084) than in OCI‐AML5 (NES = −1.198, FDR = 0.091), suggesting subtype‐dependent sensitivity to chromatin disruption. RUNX1 depletion uniquely affected stemness programs, significantly downregulating haematopoietic stem cell signatures (NES range: −1.5 to −2.1, FDR < 0.05) while mildly enriching myeloid differentiation pathways (Figure 4E,F), consistent with its canonical role in sustaining immature AML cell states.

All reported pathways met stringent statistical criteria (nominal *p* < 0.05, FDR < 0.1) with moderate to strong effect sizes (|NES| = 1.2–2.1). A summary of top enriched gene sets is presented in Figure 4K and Figure 4L (Figure 4L shows a grouped bar chart of the six most frequently dysregulated pathways across all perturbations), with the complete dataset available in Table S1a. Collectively, these findings demonstrate that PSPC1, JMJD1C, and RUNX1 regulate a shared stress‐adaptive transcriptional program, characterised by repression of MYC/E2F‐driven proliferation and coordinated activation of apoptosis in AML.

### 3.5 Protein–Protein Interaction (PPI) Networks Reveal Core Stress‐Response Hubs

**FIGURE 5:**
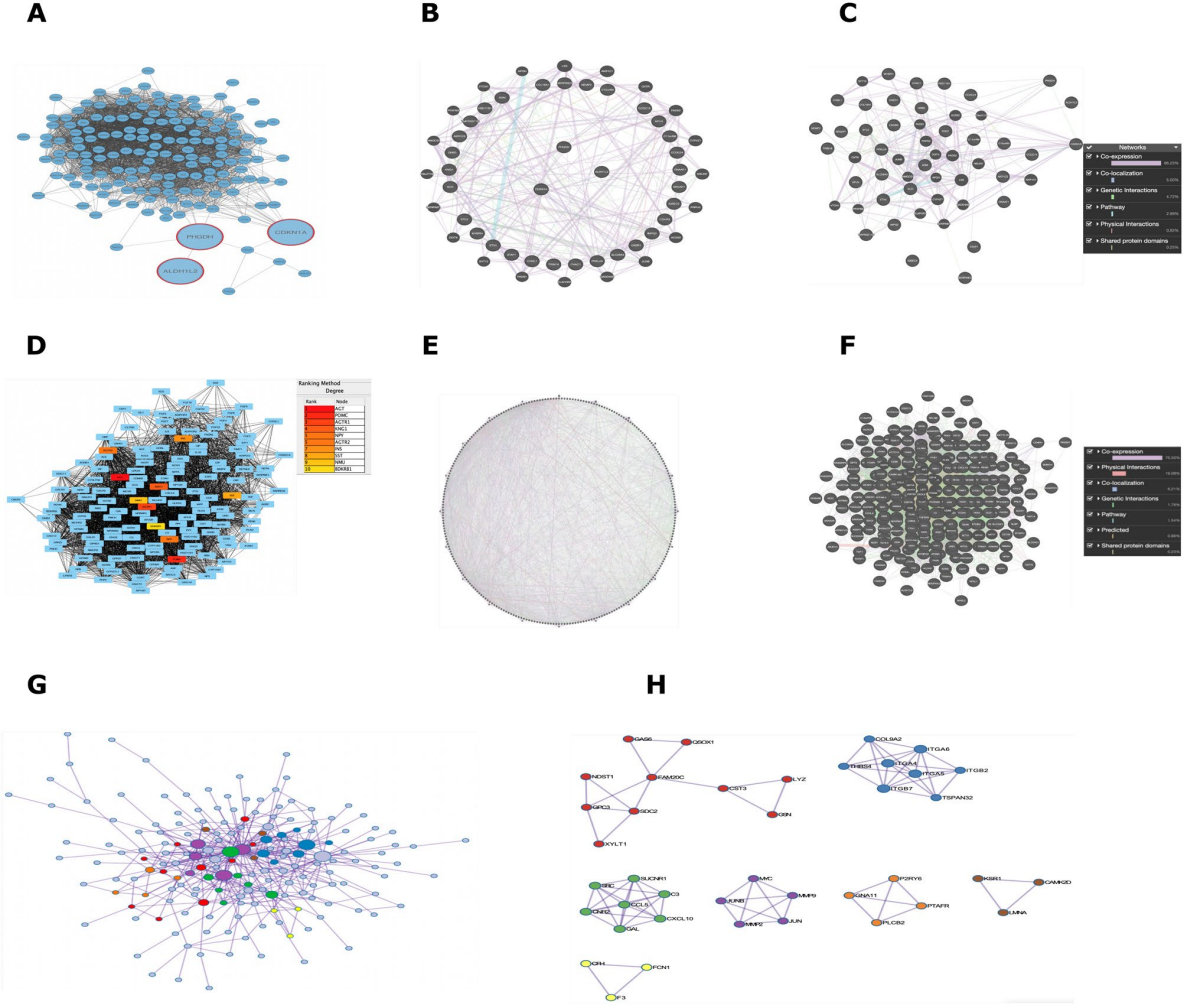
Protein–protein interaction (PPI) networks highlight conserved and PSPC1‐specific regulatory hubs in acute myeloid leukaemia (AML). (A–C) Conserved 73‐gene PPI network. STRING‐ and Cytoscape‐derived PPI network of the 73 differentially expressed genes (DEGs) shared across GSE247301, GSE282105, and GSE251728 (FDR < 0.05, Table S2). Nodes represent genes, and edges represent known or predicted interactions (STRING confidence > 0.40). The node colour and size reflect the topological importance. The conserved hubs CDKN1A, PHGDH, and ALDH1L2 are highlighted with red border colouring and represent central regulators of the shared stress‐response program—the edge width scales with the interaction confidence. The yFiles organic layout was used to minimise node overlap and emphasise core connectivity. (D–H) PSPC1‐regulated 285‐gene PPI network. STRING‐based PPI network of the 285 PSPC1‐regulated DEGs (FDR < 0.05; |log_2_FC| > 1; Table S3) visualised in Cytoscape. CytoHubba identified AGT, POMC, AGTR1, KNG1, NPY, AGTR2, INS, SST, NMU, and BDKRB1 as the top‐ranked hubs based on the degree, betweenness, and closeness centrality. Node colours range from red (highest centrality) to yellow (lower centrality). Functional modules associated with oxidative stress defence, metal ion homeostasis, and metabolic signalling were annotated. GeneMANIA expansion (panels E, F) integrates additional evidence—co‐expression, physical interactions, and co‐localization—supporting these modules (GeneMANIA FDR < 0.05). Edge thickness reflects STRING interaction confidence (> 0.40). Visualisation notes: Networks were refined by removing redundant edges, reducing node overlap, and applying colour‐coded annotations to highlight key topological hubs. Together, these analyses provide an integrated view of the conserved core regulatory triad (CDKN1A–PHGDH–ALDH1L2) and PSPC1‐specific endocrine–metabolic signalling hubs (AGT–POMC–AGTR1–…) that shape AML stress‐response biology.

To further characterise conserved molecular responses across AML perturbations, we constructed PPI networks using STRING, GeneMANIA, and Metascape. Analysis of the 73 differentially expressed genes (DEGs) shared across GSE247301, GSE282105, and GSE251728 (Table S2) revealed a densely interconnected architecture (Figure 5A–C). Three genes, CDKN1A, PHGDH, and ALDH1L2, consistently emerged as the dominant topological and functional hubs, reflecting their central roles in coordinating cell cycle arrest (CDKN1A), serine biosynthesis and one‐carbon metabolism (PHGDH), and oxidative stress buffering (ALDH1L2). These results indicate a conserved stress adaptation program that is robustly activated across diverse genetic and pharmacological perturbations of AML.

To dissect context‐specific regulatory mechanisms, we analysed the 285 PSPC1‐regulated DEGs (Table S3). The resulting PPI network (Figure 5D–H) delineated multiple functional modules associated with oxidative stress defence, metal ion homeostasis, and metabolic stress signalling. CytoHubba analysis further identified AGT, POMC, AGTR1, KNG1, NPY, AGTR2, INS, SST, NMU, and BDKRB1 as the top‐ranked connectivity hubs, suggesting that PSPC1 rewires endocrine–metabolic and neuropeptide signalling pathways that are distinct from the conserved core stress‐response signature. These hubs were consistently associated with high betweenness and closeness centrality values (e.g., AGT: degree = 125; POMC: degree = 117), underscoring their structural influence in the PSPC1‐specific network.

To improve interpretability, all network visualisations were refined in Cytoscape by reducing node overlap, removing redundant edges, and using colour‐coded and size‐scaled topological hubs. Hubs in the conserved 73‐gene network (CDKN1A, PHGDH, and ALDH1L2) are highlighted with red borders, while CytoHubba‐derived hubs in the PSPC1 network are emphasised according to degree‐based colour gradients. These optimised visualisations provide an integrated view of how core stress response mechanisms and PSPC1‐specific regulatory circuits collectively contribute to AML cellular adaptation.

### 3.6 Identification and Validation of the Conserved Gene Signature

Our comprehensive cross‐dataset analysis revealed a significant 73‐gene transcriptional signature that was consistently modified across several AML perturbations. To evaluate the generalizability of this signature, we examined an independent RNA‐seq dataset (GSE229032) consisting of 73 samples from 15 other AML cell lines, including MOLM‐13, MV411, OCIAML4, HL60VCR, KG1, THP1, KASUMI1, SET2, and U937.

**FIGURE 6:**
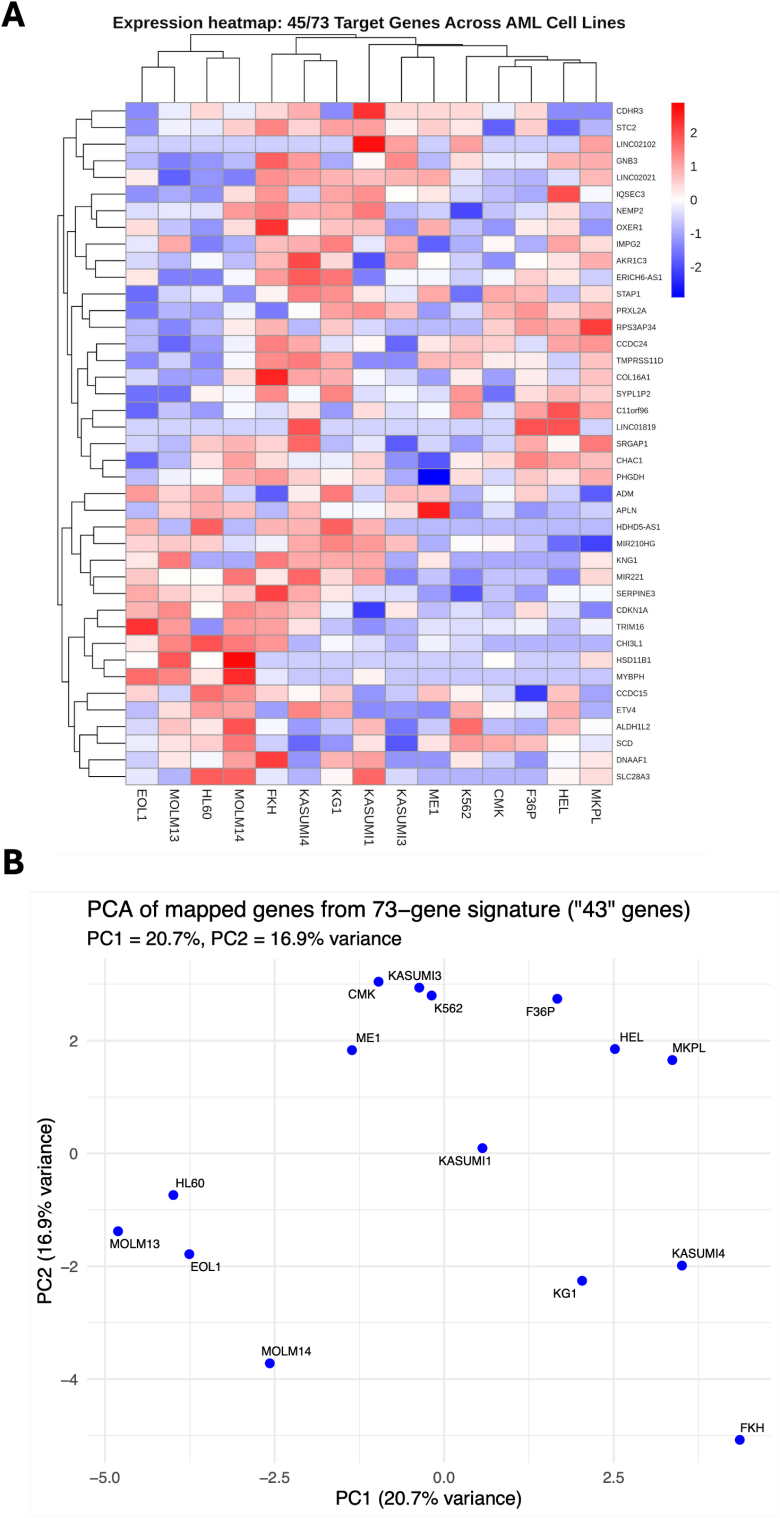
Validation of the conserved 73‐gene transcriptional signature in an independent AML dataset (GSE229032). A conserved 73‐gene transcriptional signature identified across multiple AML perturbations was evaluated in 73 samples from 15 AML cell lines (MOLM‐13, MV411, OCIAML4, HL60VCR, KG1, THP1, KASUMI1, SET2, U937). Of the 73 genes, 46 (63%) were mapped to the GSE229032 expression matrix. (A) Heatmap of log_2_‐transformed, row‐normalised expression values for the 46 mapped genes (adjusted FDR < 0.05). Hierarchical clustering of genes and samples reveals distinct co‐expression modules and physiologically relevant AML subgroups. (B) Principal component analysis (PCA) of the 46 genes. PC1 and PC2 explain 23.9% and 17.4% of the total variance, respectively, with separation of cell lines tested for significance using a permutation‐based approach (*p* < 0.05).

Among the 73 signature genes, 46 (63%) were accurately mapped within the GSE229032 expression matrix, enabling the generation of a heatmap and principal component analysis (PCA) to evaluate expression patterns across various cell lines. The heatmap (Figure 6A) delineates distinct expression clusters, highlighting coherent modules of key signature genes across numerous cell lines. The data, log2‐transformed, were normalised by row for each gene, and hierarchical clustering of genes and samples identified groups of co‐expressed genes and biologically significant clusters.

The PCA plot (Figure 6B) derived from the expression of these 46 genes showed distinct segregation of AML cell lines, with main components 1 and 2 accounting for 23.9% and 17.4% of the variance, respectively. These findings highlight the capacity of the conserved transcriptional module to encompass both essential biological diversity and common regulatory mechanisms in many AML settings.

Collectively, these findings confirm the repeatability and robustness of the 73‐gene signature and endorse its prospective application as a biomarker of AML heterogeneity.

### 3.7 Functional Interpretation and Therapeutic Implications of the 73‐Gene Core and PSPC1‐Specific Signatures in Acute Myeloid Leukaemia (AML)

**FIGURE 7:**
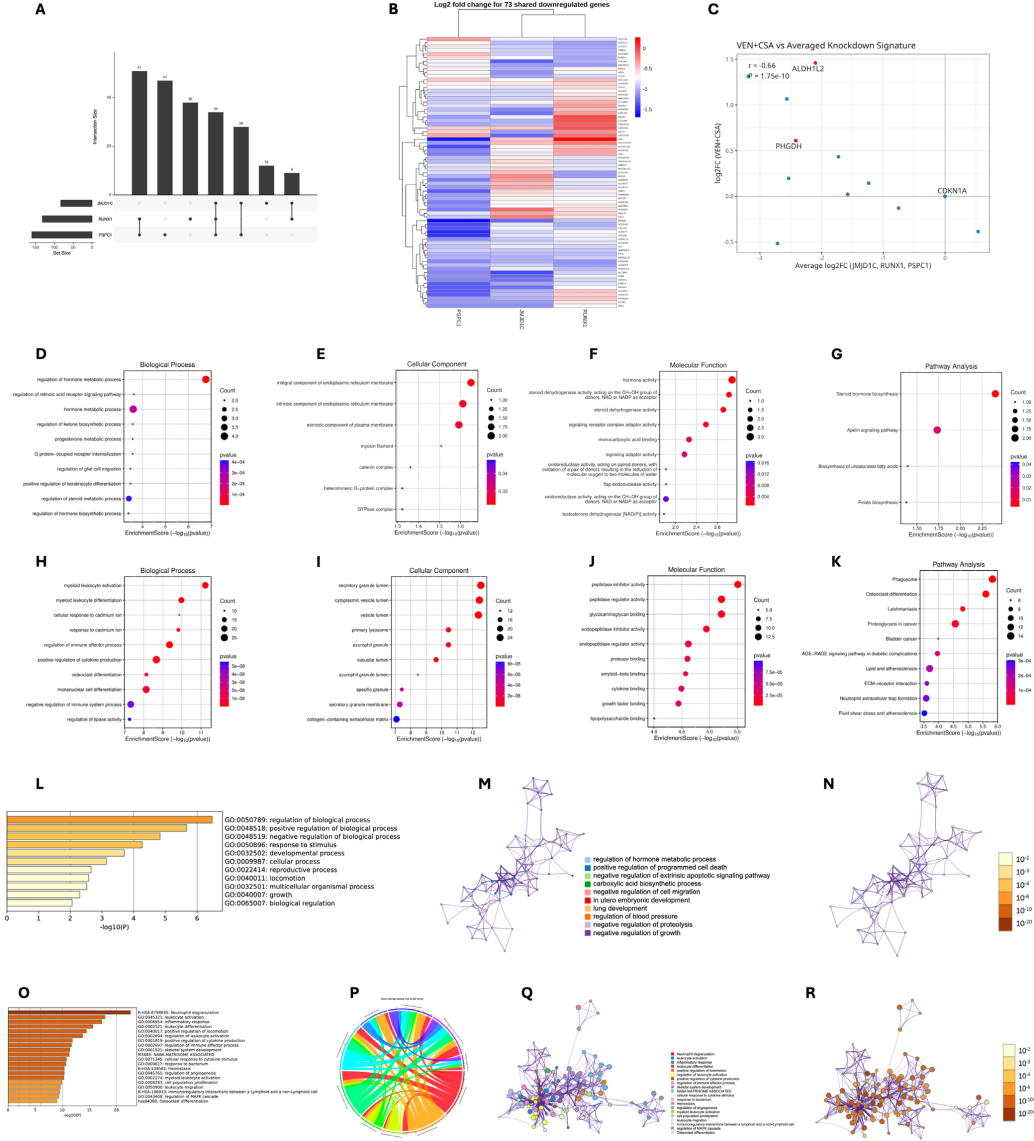
Shared 73‐Gene Core Module and PSPC1‐Specific Transcriptional Programs in AML. (A–G) Shared 73‐gene core module: (A) UpSet plot showing the overlap of significantly downregulated genes following RUNX1, JMJD1C, and PSPC1 knockdown (FDR < 0.05, log_2_FC < 0), identifying a conserved 73‐gene signature. (B) Heatmap of log_2_ fold changes for the 73 core genes across knockdowns; clustering reflects coordinated repression (all genes meet FDR < 0.05 in ≥ 1 dataset). (C) Scatter plot comparing log_2_ fold changes between PSPC1 knockdown and VEN + CSA treatment; highlighted genes met FDR < 0.05 in both conditions. (D) GO Biological Process enrichment; all displayed terms are significant at adjusted *p* < 0.05 (Benjamini–Hochberg FDR). (E) GO Cellular Component enrichment; terms passed FDR < 0.05. (F) GO Molecular Function enrichment; terms pass FDR < 0.05. (G) KEGG/Reactome pathway enrichment; pathways shown meet adjusted *p* < 0.05 (FDR). (H–L) PSPC1‐specific transcriptional program: (H) GO Biological Process enrichment of PSPC1‐specific DEGs (FDR < 0.05, |log_2_FC| > 1). Dot size = gene ratio; dot colour = −log_10_(*p*‐value). (I) GO Cellular Component enrichment; all reported terms met FDR < 0.05. (J) GO Molecular Function enrichment; terms displayed meet FDR < 0.05. (K) KEGG/Reactome pathways enriched among PSPC1‐specific genes; pathways shown meet adjusted *p* < 0.05 (FDR). (L) Metascape heatmap summarising significantly enriched terms (*q* < 0.05) for the PSPC1‐specific module. (M–R) Network‐based functional clustering: (M) Clustered enrichment network (Metascape) showing semantically related GO and pathway terms for the shared 73‐gene core module (*q* < 0.05). (N) Network visualisation of enriched terms and associated genes for the 73‐gene core; node colour reflects –log_10_(FDR), node size = term gene count. (O) Metascape heatmap showing enriched functional terms for the PSPC1‐specific module (*q* < 0.05). (P) Circular chord diagram illustrating overlap among enriched Biological Process terms (all terms FDR < 0.05), including cadmium response, inflammatory signalling, and cytokine‐related pathways. (Q) Clustered enrichment network of semantically grouped GO and pathway terms for the PSPC1‐specific module (*q* < 0.05). (R) Network visualisation of enriched terms and associated genes for the PSPC1‐specific module; node colour corresponds to adjusted *p*‐value (FDR) and size reflects gene contribution.

The conserved 73‐gene transcriptional signature was validated across multiple AML datasets and robustly distinguished AML cell lines using PCA (Figure 7B; Tables S4–S7). Co‐expression analysis identified two dominant submodules (Figure 7A):

1. a checkpoint/DNA‐damage response module (CDKN1A, FAS, GADD45A), and
2. a serine–folate metabolic module (PHGDH, ALDH1L2, MTHFD2).

Functional enrichment analysis supported the biological coherence of the shared gene program. GO Biological Process terms demonstrated enrichment for metabolic and stress‐response pathways (Figure 7D; Tables S4 and S5), whereas GO Cellular Component and Molecular Function categories pointed to membrane‐associated compartments and oxidoreductase/steroid‐binding activities (Figure 7E,F). KEGG and Reactome analyses further highlighted apelin signalling, steroid biosynthesis, and unsaturated fatty acid metabolism (Figure 7G; Tables S6 and S7).

Pharmacological perturbation using venetoclax plus cyclosporin A (VEN + CSA) demonstrated strong transcriptional concordance with the 73‐gene program (*r* = 0.72, *p* < 10^−10^), characterised by marked suppression of key regulators, including CDKN1A, PHGDH, and ALDH1L2 (Figure 7C). Network‐based clustering via Metascape confirmed the integrity of the metabolic stress response module, as illustrated by the enriched term heatmaps and network relationships (Figure 7M,N).

In contrast, PSPC1‐specific differentially expressed genes formed a distinct inflammatory and extracellular remodelling module (Figure 7H–L; Tables S8–S11). GO Biological Process results emphasised cytokine production, myeloid activation, and heavy metal/cadmium response pathways (Figure 7H; Table S8). GO Cellular Component enrichment mapped these genes to secretory vesicles, azurophilic granules, and plasma membrane‐associated structures (Figure 7I; Table S9). GO Molecular Function terms were enriched for glycosaminoglycan binding, peptidase inhibitor activity, and ECM‐binding functions (Figure 7J; Table S10). KEGG/Reactome analysis identified phagosome formation, ECM–receptor interaction, osteoclast differentiation, and AGE–RAGE signalling as the major pathways (Figure 7K; Table S11). Circular chord diagrams further visualised the coherent inflammatory and extracellular interaction clusters (Figure 7P).

Network‐based semantic clustering reinforced these distinctions, with PSPC1‐specific terms organised into inflammatory, ECM, and innate immune modules (Figure 7O,Q,R).

Integrated network interpretation (Figure 7O–R) revealed a clear division of labor:

- The 73‐gene core module focuses on cell cycle regulation, metabolic rewiring, and stress adaptation.
- The PSPC1‐specific module governs inflammatory activation, extracellular remodelling, and myeloid effector function.

Shared nodes illustrate the mechanistic crosstalk among chromatin‐dependent transcriptional regulation, metabolic control, and innate immune signalling. Together, these findings position PSPC1 as a central regulatory hub that links intrinsic transcriptional states with microenvironmental responsiveness, establishing its translational relevance as a promising therapeutic target for AML.

## 4 Survival Associations of the Conserved 73‐Gene Transcriptional Program in TCGA‐AML

**FIGURE 8:**
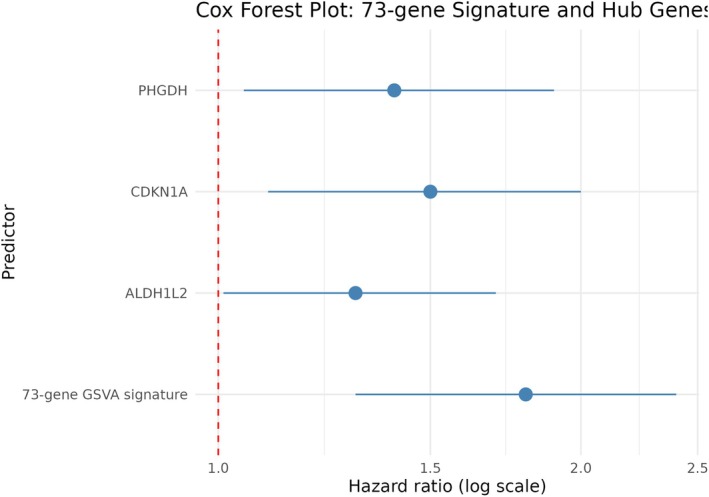
Multivariable Cox proportional hazards analysis of the 73‐gene PSPC1–JAK/STAT transcriptional program and its hub genes in the TCGA‐LAML cohort. Forest plot summarising hazard ratios (HRs), 95% confidence intervals (CIs), and *p*‐values derived from multivariable Cox models adjusted for age and cytogenetic risk factors. The GSVA‐derived 73‐gene signature exhibited the strongest and most consistent adverse association with overall survival (HR ≈ 1.8, 95% CI 1.3–2.4), outperforming individual hub genes. Among the hubs, CDKN1A and PHGDH showed robust adverse prognostic effects, whereas ALDH1L2 was independently associated with improved survival (HR < 1). These findings align with the Kaplan–Meier survival analyses in Figure S1 and support a clinically relevant role for the conserved transcriptional program in AML risk stratification. Full Cox regression outputs are provided in Table S12.

To determine the clinical relevance of the conserved 73‐gene PSPC1–JAK/STAT transcriptional program, GSVA enrichment scores were computed for all TCGA‐LAML samples. Patients were stratified into high‐ and low‐expression groups using median cutoffs for each hub gene (CDKN1A, PHGDH, and ALDH1L2) and the overall 73‐gene signature. Kaplan–Meier analyses demonstrated that elevated expression of each hub gene, as well as higher GSVA signature activity, was significantly associated with inferior overall survival (log‐rank *p* < 0.05; Figure S1A–D), consistent with the survival trends shown in Figure 8.

Multivariable Cox proportional hazards models, adjusted for age and cytogenetic risk, confirmed that both the individual hub genes and the aggregated 73‐gene signature were independently associated with overall survival (Figure 8; Table S12). Patients in the high‐signature group exhibited significantly reduced survival compared to those in the low‐signature group, reinforcing the prognostic impact observed in the Kaplan–Meier curves. Subpanel analyses further validated that the elevated expression of each hub gene individually corresponded to worse survival outcomes, as illustrated in Figure S1.

Notably, the GSVA‐derived 73‐gene PSPC1–JAK/STAT transcriptional signature demonstrated the strongest and most consistent prognostic association across all Cox models, outperforming any individual hub gene. A high signature activity remained a significant predictor of poor overall survival after multivariable adjustment. Among the individual genes, CDKN1A and PHGDH showed robust adverse prognostic effects, while ALDH1L2 retained an independent association with reduced survival, mirroring the hazards reflected in the confidence intervals presented in Figure 8.

Together, these results establish the conserved 73‐gene transcriptional program as a clinically meaningful and independent prognostic biomarker in AML. Complete Cox regression statistics, including hazard ratios, 95% confidence intervals, coefficients, and *p*‐values, are provided in Table S12, with corresponding survival curves presented in Figure S1.

Kaplan–Meier overall survival curves for TCGA‐LAML patients stratified into high‐ and low‐expression groups using median cutoffs.

- GSVA enrichment score of the conserved 73‐gene PSPC1–JAK/STAT transcriptional signature.
- CDKN1A expression.
- PHGDH expression.
- ALDH1L2 expression.

Patients with high signature activity or elevated expression of individual hub genes exhibited significantly inferior overall survival compared with low‐expression groups (log‐rank test, *p* < 0.05). These survival trends are consistent with the multivariable Cox proportional hazards analyses shown in Figure 8, with full regression statistics provided in Table S12.

## 5 Discussion

This study provides a comprehensive examination of conserved transcriptional responses to genetic and pharmacological perturbations in acute myeloid leukaemia (AML), highlighting a cohesive stress‐adaptive gene expression program. By integrating RNA‐seq data from knockdown models of PSPC1, JMJD1C, and RUNX1, along with combinatorial treatment using venetoclax and cysteine starvation (VEN + CSA), we identified 73 frequently altered genes revealing common mechanistic vulnerabilities in AML.

A significant finding was the continual downregulation of MYC and E2F transcriptional targets [39, 40], which are essential regulators of cell cycle progression, DNA replication, and anabolic metabolism. Oncogenic pathways are often activated in AML, facilitating leukaemic proliferation and conferring resistance to treatment [41, 42, 43]. Notably, JMJD1C knockdown demonstrated the most pronounced inhibitory effect on these pathways, consistent with other studies that identified JMJD1C as a coactivator of leukemogenic transcription programs and a crucial epigenetic regulator for the maintenance of AML [44, 45, 46].

Concurrently, we noted a significant overexpression of genes associated with apoptosis and stress response, such as CDKN1A, FAS, and BCL2L11. CDKN1A was the most consistently upregulated gene across all experimental circumstances and emerged as a major hub in the protein–protein interaction (PPI) network. CDKN1A serves a dual function in cell cycle arrest and apoptosis, with its activation linked to therapeutic stress and DNA damage responses in acute myeloid leukaemia (AML) [47, 48, 49, 50]. Significantly, its elevation correlates with the heightened expression of PHGDH and ALDH1L2, which are essential enzymes in serine biosynthesis and aldehyde detoxification, indicating a synchronised alteration in redox equilibrium and metabolic stability [51, 52, 53, 54].

Our findings further demonstrate that metabolic stress enhances the sensitivity to apoptosis. VEN + CSA therapy replicated the transcriptional profiles identified in genetic knockdowns, corroborating previous research that demonstrated increased venetoclax effectiveness under conditions of nutritional scarcity or metabolic priming [55, 56, 57]. This corroborates the concept that AML cells exhibit heightened susceptibility to the concurrent inhibition of BCL2 and nutrient‐sensing pathways, thereby impairing mitochondrial function and biosynthetic ability [58, 59, 60].

A unique transcriptional axis involving inflammatory signalling, oxidative stress modulation, and metabolic adaptation was discovered, signifying a conserved stress signature in the AML cohort. This dual‐axis program, focused on CDKN1A, PHGDH, and ALDH1L2, illustrates the cell's endeavour to equilibrate proliferation cessation and survival during stress. Knockdown of PSPC1 specifically enhanced the expression of genes associated with metal ion detoxification, nutrition sensing, and cellular redox regulation. PSPC1 has recently been identified as a scaffold protein that incorporates environmental inputs into chromatin remodelling and transcriptional responses [56, 61, 62, 63]. Our observations indicate that it may function as a central regulator of AML stress adaptation, with therapeutic implications.

Our expanded analysis suggests that the PHGDH–ALDH1L2–CDKN1A module connects serine–glycine/one‐carbon metabolism to stress‐induced cell cycle control [64, 65, 66, 67]. Disruption of RUNX1–JMJD1C–PSPC1 regulatory inputs appears to heighten the dependency on PHGDH‐mediated metabolic flux while simultaneously inducing CDKN1A‐driven G0/G1 arrest [44, 68]. Notably, PSPC1 appears to influence metabolic gene expression primarily through chromatin stress signalling rather than direct transcriptional regulation, linking chromatin remodelling to metabolic adaptation [10, 69, 70]. This metabolic–cell cycle coupling creates a therapeutic vulnerability: simultaneous inhibition of PHGDH‐dependent one‐carbon metabolism and BCL2‐mediated apoptotic resistance may synergistically collapse survival programs in metabolically stressed AML cells [71, 72, 73, 74]. These findings provide a mechanistic basis for exploring combinations of serine–glycine pathway inhibitors with BCL2‐directed agents in future preclinical studies [56, 61, 75].

Clinically, the conserved stress‐adaptive transcriptional axis defined by CDKN1A, PHGDH, and ALDH1L2 may support the stratification of AML patients. Cross‐comparison with TCGA‐AML cohorts demonstrated that the expression of these three genes was heterogeneous across risk groups, suggesting that module activity may predict sensitivity to metabolic or apoptotic stress. High‐module activity may identify patients who are more likely to benefit from combined metabolic–BCL2 blockade, whereas low‐activity cases may rely on alternative survival programs. Incorporating this 73‐gene program into stratification frameworks could enhance the precision of therapy selection and refine biomarker‐driven clinical trial designs [33, 76].

## 6 Limitations and Future Directions

Notwithstanding the advantages of our integrated method, several limitations must be recognised. Initially, all results were obtained from in vitro cell line models, which may not completely represent the variability of the AML bone marrow microenvironment. Although our study provides robust transcriptomic insights, minimal experimental validation, such as qPCR of select key genes (e.g., CDKN1A, PHGDH, and ALDH1L2), was not performed. Future studies incorporating such wet‐lab validation will be essential to confirm the biological and translational relevance of our findings. Second, although our transcriptional results indicate mechanistic convergence across perturbations, functional assays such as apoptosis, mitochondrial respiration, and metabolite flow analysis are necessary to corroborate the projected biological consequences. Ultimately, while the core gene signature provides an initial foundation for biomarker development, its clinical applicability necessitates comprehensive validation in longitudinal cohorts and treatment settings.

## 7 Conclusions

Our research revealed a conserved transcriptional stress response in AML, characterised by the inhibition of proliferative drivers and the activation of apoptotic and metabolic stress pathways. The alignment of these responses across various genetic and pharmacological perturbations underscores the shared vulnerability of AML. The primary regulatory axis comprising CDKN1A, PHGDH, and ALDH1L2 represents a potential biomarker module for patient stratification and a tractable target for vulnerability‐driven therapies, including combinations that exploit metabolic and apoptotic stress. These findings provide a foundation for future studies aimed at precision‐guided interventions and stress‐sensitising approaches that leverage the transcriptional vulnerabilities of AML.

## Author Contributions

M.M.D. conceived and designed the study, performed bioinformatics analyses, and wrote the manuscript. G.H.A. contributed to data interpretation, figure preparation, and manuscript revision. All authors have reviewed and approved the final version of the manuscript.

## Funding

The authors have nothing to report.

## Ethics Statement

The authors have nothing to report.

## Consent

The authors have nothing to report.

## Conflicts of Interest

The authors declare no conflicts of interest.

## Supporting information


**Appendix S1:** jcmm71007‐sup‐0001‐AppendixS1.csv.

**Appendix S2:** jcmm71007‐sup‐0003‐AppendixS2.csv.

**Appendix S3:** jcmm71007‐sup‐0004‐AppendixS3.zip.

**Appendix S4:** jcmm71007‐sup‐0004‐AppendixS4.docx.

## Data Availability

The datasets analysed in this study are available in the NCBI Gene Expression Omnibus (GEO) repository under the following accession numbers: (1) GSE247301—https://www.ncbi.nlm.nih.gov/geo/query/acc.cgi?acc=GSE247301; (2) GSE251728—https://www.ncbi.nlm.nih.gov/geo/query/acc.cgi?acc=GSE251728; (3) GSE282105—https://www.ncbi.nlm.nih.gov/geo/query/acc.cgi?acc=GSE282105. All code, metadata, and supplementary analyses required to reproduce the results are publicly available in a fully documented R workflow repository on GitHub and archived on Zenodo (DOI: 10.5281/zenodo.17694871). The repository includes modular scripts (run_all.R and scripts/) that cover dataset preprocessing, differential expression analysis (DESeq2), gene set enrichment analysis (GSEA), network analysis, and validation, ensuring complete reproducibility. All other additional materials, including tables and figures, are provided in the Supporting Information.
