# Supplementary material for: Integrative Bioinformatics Reveals Novel Molecular Mechanisms and Therapeutic Targets in Acute Myeloid Leukaemia
Source: J Cell Mol Med. 2026 Jan 6;30(1):e71007. doi: 10.1111/jcmm.71007 (PMC12771679; doi:10.1111/jcmm.71007)
Supplement: Supplementary file 3 — Appendix S3: jcmm71007‐sup‐0004‐AppendixS3.zip. [file JCMM-30-e71007-s002.zip › jcmm71007-sup-0004-AppendixS4.docx]

**Supplementary Materials**

**Manuscript Title:** Integrative Bioinformatics Reveals Novel Molecular Mechanisms and Therapeutic Targets in Acute Myeloid Leukemia

**Supplementary Figure S1. Prognostic impact of the 73‑gene PSPC1–JAK/STAT signature and key hub genes in TCGA‑LAML**

**(A–C)** Kaplan–Meier overall survival (OS) curves for **CDKN1A**, **PHGDH**, and **ALDH1L2** in the TCGA‑LAML cohort. Patients were stratified into high- and low-expression groups based on the median expression of each gene. Survival differences were assessed using the log-rank test (**p < 0.05**).

**(D)** Kaplan–Meier OS curve for the GSVA-derived **73-gene PSPC1–JAK/STAT transcriptional signature**. Patients were divided into high- and low-signature groups according to the cohort median GSVA enrichment score. Log-rank p-values are indicated on the plot.

**Multiple-comparison correction:**

All transcriptome-wide significance tests were adjusted using the Benjamini–Hochberg false discovery rate (FDR) method. An FDR < 0.05 was considered statistically significant.

**Cox regression support:**

Full multivariable Cox proportional hazards regression results, including hazard ratios (HRs), 95% confidence intervals (CIs), and p-values—are provided in **Supplementary Table S12**. These analyses correspond to the Cox forest plot in Figure 8, the Kaplan–Meier survival curves in Supplementary Figure S1, and the survival analyses described in Results Section 8 (“Survival Associations of the Conserved 73-Gene Transcriptional Program in TCGA‑AML”). All Cox models were adjusted for **age** and **cytogenetic risk** according to TCGA‑LAML clinical standards.

**Supplementary Table S12. Multivariable Cox proportional hazards regression for the 73‑gene PSPC1–JAK/STAT signature and hub genes in TCGA‑LAML**

This table presents the full multivariable Cox proportional hazards regression results evaluating the prognostic relevance of the 73‑gene PSPC1–JAK/STAT transcriptional program and its key hub genes in the TCGA‑LAML cohort. Hazard ratios (HRs), 95% confidence intervals (CIs), and p‑values are reported from models adjusted for age and cytogenetic risk. The table also includes the estimates used to generate the survival stratification in Supplementary Figure S1.

| **Predictor** | **HR (Main Table)** | **Lower 95% CI** | **Upper 95% CI** | **p‑value** | **HR (Supporting FigureS1)** | **Lower CI** | **Upper CI** |
| --- | --- | --- | --- | --- | --- | --- | --- |
| 73‑gene GSVA signature | 0.866 | 0.705 | 1.065 | 0.173 | 1.8 | 1.3 | 2.4 |
| CDKN1A | 0.871 | 0.734 | 1.033 | 0.112 | 1.5 | 1.1 | 2.0 |
| PHGDH | 0.847 | 0.714 | 1.005 | 0.057 | 1.4 | 1.05 | 1.9 |
| ALDH1L2 | 0.820 | 0.696 | 0.966 | 0.018 | 1.3 | 1.01 | 1.7 |

**Notes:**

- Multivariable Cox models were adjusted for age and cytogenetic risk. Predictors (73‑gene GSVA enrichment score and hub gene expression for CDKN1A, PHGDH, and ALDH1L2) were modeled as continuous variables.
- HR < 1 (Main Table) indicates that higher expression or higher signature score is associated with improved overall survival; HR > 1 (Supporting FigureS1) shows poorer survival with higher expression, corresponding to Kaplan–Meier stratifications.
- These results correspond to Figure 8 (Cox forest plot), Supplementary Figure S1 (Kaplan–Meier survival curves), and the survival analyses described in Results Section 8 (“Survival Associations of the Conserved 73‑Gene Transcriptional Program in TCGA‑AML”).
