# Supplementary material for: Integrative Bioinformatics Reveals Novel Molecular Mechanisms and Therapeutic Targets in Acute Myeloid Leukaemia
Source: J Cell Mol Med. 2026 Jan 6;30(1):e71007. doi: 10.1111/jcmm.71007 (PMC12771679; doi:10.1111/jcmm.71007)
Supplement: Supplementary file 3 — Appendix S3: jcmm71007‐sup‐0004‐AppendixS3.zip. [file JCMM-30-e71007-s002.zip › jcmm71007-sup-0002-AppendixS2.pdf]

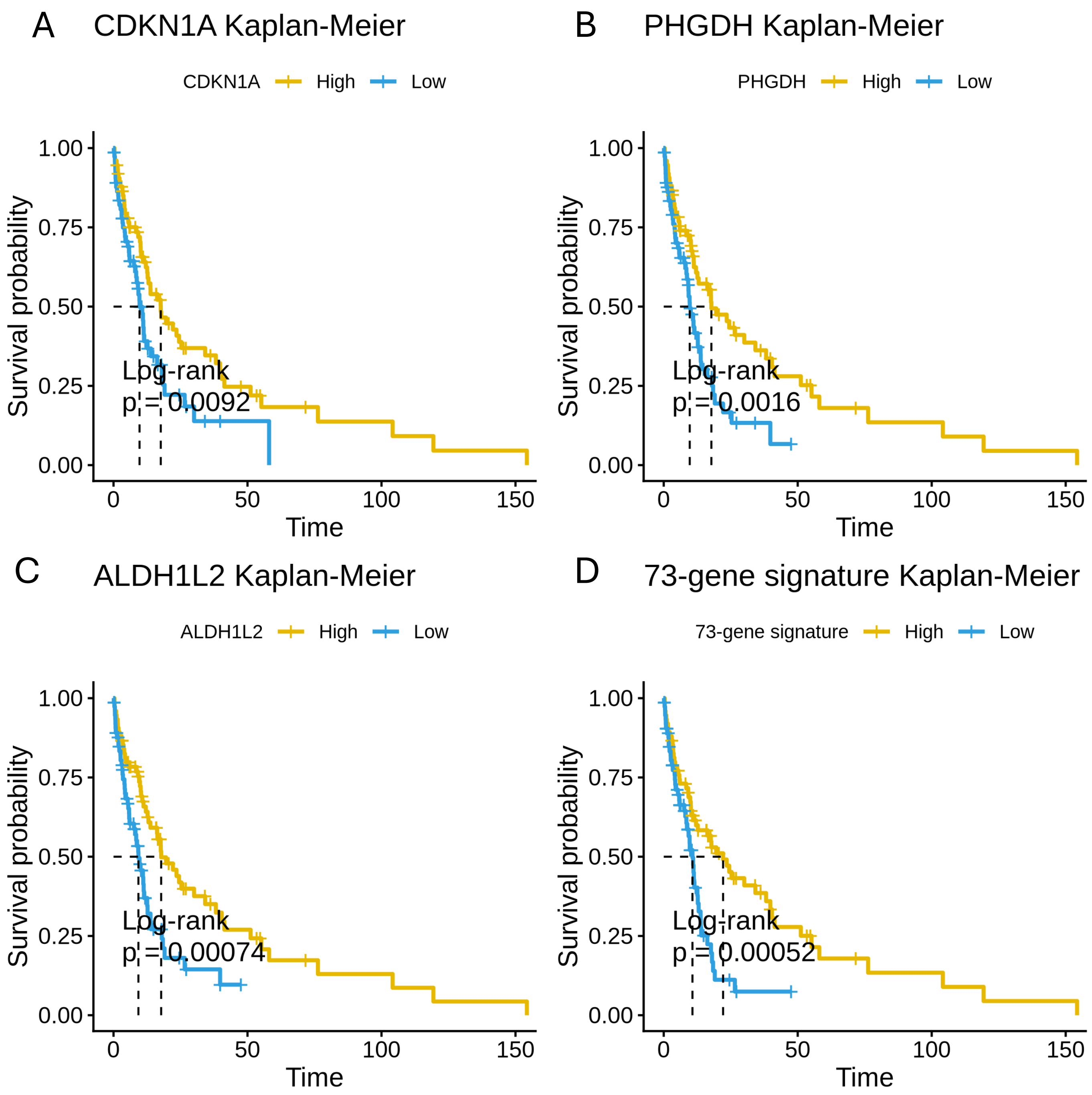

A
CDKN1A Kaplan-Meier
CDKN1A High Low
Survival probability
1.00
0.75
0.50
0.25
0.00
Log-rank
p = 0.0092
0
50
100
150
Time
B
PHGDH Kaplan-Meier
PHGDH High Low
Survival probability
Log-rank
p = 0.0016
Time
C
ALDH1L2 Kaplan-Meier
ALDH1L2 High Low
Survival probability
Log-rank
p = 0.00074
Time
D
73-gene signature Kaplan-Meier
73-gene signature High Low
Survival probability
Log-rank
p = 0.00052
Time
