# Supplementary material for: Integrative Bioinformatics Reveals Novel Molecular Mechanisms and Therapeutic Targets in Acute Myeloid Leukaemia
Source: J Cell Mol Med. 2026 Jan 6;30(1):e71007. doi: 10.1111/jcmm.71007 (PMC12771679; doi:10.1111/jcmm.71007)
Supplement: Supplementary file 4 — Appendix S4: jcmm71007‐sup‐0004‐AppendixS4.docx. [file JCMM-30-e71007-s004.docx]

- **Supplementary Table S1a. Statistical summary of differential expression analysis.**

This table summarizes the DESeq2 outputs, including the number of upregulated and downregulated genes, adjusted p-values (padj), and log2 fold changes across all datasets analyzed.

In addition, representative GSEA results are provided by panel, highlighting enriched pathway categories with their corresponding normalized enrichment score (NES) ranges, false discovery rate (FDR) ranges, and biological interpretation:

| Panel | Pathway_Category | Typical_NES_Range | FDR_Range | Interpretation |
| --- | --- | --- | --- | --- |
| A-C | Proliferation (MYC/E2F) | -2.1 to -1.3 | 0.007 - 0.084 | Downregulated |
| D | Apoptosis | 1.2 to 1.9 | 0.031 - 0.058 | Upregulated |
| E-F | Stemness/Differentiation | -2.1 to -1.5 | 0.032 - 0.045 | Downregulated |
| G-J | Mixed Effects | -1.6 to 1.3 | 0.058 - 0.091 | Context-dependent |

- **Supplementary Table S1b. Leading-edge analysis summary of enriched pathways.**

Results of GSEA leading-edge subsets, showing normalized enrichment score (NES), false discovery rate (FDR), and contributing gene members for significantly enriched pathways

| Pathway | NES_overall | FDR_overall | NES_MOLM13_PSPC1KD | NES_OCIAML5_PSPC1KD |
| --- | --- | --- | --- | --- |
| HALLMARK_APOPTOSIS | 0.27766138 | 0.249858 | 0.15781832 | 0.29216339 |
| HALLMARK_DNA_REPAIR | 0.13032276 | 0.2668643 | 0.07032031 | 0.31562649 |
| HALLMARK_E2F_TARGETS | 0.02038353 | 0.0466088 | 0.0002316 | 0.0039869 |
| HALLMARK_MTORC1_SIGNALING | 0.1404889 | 0.2409994 | 0.00028123 | 0.13218893 |
| HALLMARK_MYC_TARGETS_V1 | 0.03574492 | 0.3317545 | 0.00062863 | 0.1302865 |
| HALLMARK_MYC_TARGETS_V2 | 0.03668339 | 0.3960904 | 0.00267366 | 0.0120645 |

- **Supplementary Table S2. Conserved 73-gene core signature.**

Shared stress-adaptive gene signature identified across PSPC1, JMJD1C, and RUNX1 knockdowns combined with venetoclax treatment and cysteine deprivation.

Gene

AC004449

AC005838

AC007541

AC008972

AC009533

AC011815

AC022306

AC022432

AC090186

AC092171

AC093249

AC100858

AC104561

AC108727

AC122719

AC124248

AC131281

ADM

AKR1C3

AL023806

AL138824

AL139125

AL353743

AL353804

AL359878

AL591767

APLN

C11orf96

C15orf65

CCDC15

CCDC24

CDHR3

CHAC1

CHI3L1

COL16A1

DNAAF1

ERICH6-AS1

ETV4

FAM96AP2

GNB3

Gene

H4C10P

HDHD5-AS1

HSD11B1

IMPG2

IQSEC3

KNG1

LINC01819

LINC02021

LINC02102

MIR210HG

MIR221

MRPS30-DT

MYBPH

NEMP2

OXER1

PACERR

PRXL2A

RPS3AP34

SCD

SERPINE3

SLC28A3

SLC4A1APP1

SRGAP1

STAP1

STC2

SYPL1P2

TMPRSS11D

TRIM16

UBE2V1P3

CDKN1A

PHGDH

ALDH1L2

- **Supplementary Table S3. Protein–protein interaction (PPI) network genes.**

List of genes included in the PPI network derived from 285 DEGs, with network statistics and interaction confidence scores.

| Gene | log2FC_PSPC1 | pval_PSPC1 |
| --- | --- | --- |
| A2M | 1.34751951 | 1.12E-07 |
| ABHD15 | 0.33712727 | 0.00398025 |
| AC068473.3 | 0.92488445 | 0.02696284 |
| AC246817.2 | -0.882149 | 8.57E-05 |
| ACP3 | 1.11052173 | 1.24E-11 |
| ADA2 | -0.3272829 | 5.13E-05 |
| ADAM28 | 1.69279183 | 6.55E-74 |
| ADAMDEC1 | 0.71790242 | 0.01163862 |
| ADAMTS10 | -2.1521914 | 7.00E-14 |
| ADAMTS5 | -2.7140551 | 1.55E-05 |
| ADM2 | -2.2560462 | 3.65E-17 |
| AKAP7 | -0.417587 | 4.09E-08 |
| ALDH1L2 | -2.4794529 | 0 |
| ALDH2 | 0.42115053 | 0.44395879 |
| ALDH3B1 | 0.06635033 | 0.00034399 |
| ALOX5AP | -0.9399976 | 1.03E-27 |
| ANKRD18A | 0.33852984 | 1.17E-05 |
| ANXA2 | -0.1113691 | 8.15E-06 |
| ANXA2R | -0.2389448 | 0.11128346 |
| ANXA4 | 0.75795797 | 3.37E-20 |
| ARHGAP11A | -1.9730258 | 3.18E-107 |
| ARHGEF40 | -0.2943165 | 6.19E-11 |
| ASAH1 | 0.67783543 | 2.73E-60 |
| ASF1A | 0.14924474 | 0.01010951 |
| ASS1 | -3.0154269 | 0 |
| ATG9A | 0.28673737 | 0.00256912 |
| ATP6V0A1 | 0.3754539 | 3.36E-22 |
| ATP6V1C2 | 0.11389441 | 0.06945943 |
| ATP8B2 | 0.24080242 | 1.65E-07 |
| AZU1 | 0.54248834 | 2.56E-52 |
| B4GALT2 | -1.6026069 | 4.53E-50 |
| BCL2A1 | -0.9099448 | 0.16593012 |
| BPI | -2.271751 | 0.0135607 |
| C11orf21 | 0.07318005 | 0.15631305 |
| C12orf76 | 1.02167863 | 2.03E-10 |
| C3 | 0.30008477 | 1.06E-57 |
| CA2 | -0.8906016 | 4.47E-128 |
| CAMK2D | 0.80085269 | 3.77E-34 |
| CAP2 | -0.2601914 | 0.78874135 |
| CCL5 | -0.7733899 | 6.32E-08 |
| CD109 | 0.13197037 | 2.35E-24 |
| CD180 | -1.5845281 | 2.07E-89 |
| CD1D | -0.8404513 | 0.00219293 |
| CD300LF | -0.9302359 | 0.00198364 |
| CD36 | 0.01964999 | 0.00439738 |
| CD86 | 0.16653459 | 0.00043782 |
| CDC42EP1 | -3.07309 | 5.51E-163 |
| CENPH | -1.9849632 | 1.52E-49 |
| CEP70 | -1.0081043 | 2.05E-38 |
| CFH | 0.58090652 | 2.09E-87 |
| CHAC1 | -3.9336474 | 1.23E-70 |
| CHI3L1 | -3.3429152 | 0 |
| CKAP4 | -0.5652556 | 2.23E-65 |
| CLEC10A | -1.8477485 | 2.14E-18 |
| CLEC12A | -0.1106754 | 0.00030877 |
| CMAHP | -0.3510409 | 6.12E-13 |
| CNR2 | -0.98512 | 0.14095586 |
| COL9A2 | 1.17057873 | 1.44E-125 |
| COLQ | 1.18959754 | 3.11E-08 |
| CSF1R | -0.0566798 | 0.00117155 |
| CST3 | 0.2211597 | 2.60E-17 |
| CSTA | -0.6483213 | 1.10E-06 |
| CTTN | -0.3002148 | 0.00266736 |
| CXCL10 | -2.5814408 | 2.51E-17 |
| CYBB | -0.6825947 | 7.38E-27 |
| CYP26B1 | NA | NA |
| CYP4F26P | -0.3903376 | 0.05876972 |
| CYTIP | 0.79674556 | 2.40E-13 |
| DDN | -2.6875582 | 5.77E-72 |
| DIXDC1 | -0.3442616 | 3.67E-09 |
| DLGAP1-AS3 | -0.2595804 | 0.03264808 |
| DNM1 | 0.03771058 | 1.47E-40 |
| DOCK9 | -3.0176797 | 0.02097307 |
| DTL | -2.481902 | 8.65E-131 |
| DYNC1I1 | 1.06314027 | 0.06539821 |
| EBF3 | -0.5744408 | 3.94E-07 |
| ECM1 | -0.4172809 | 9.48E-05 |
| EGFL7 | -1.4101634 | 2.89E-05 |
| EGR1 | 2.32879334 | 3.97E-110 |
| EPHB4 | -0.3152619 | 2.74E-05 |
| F3 | -1.8761478 | 5.57E-26 |
| FADS2 | -3.5166194 | 0 |
| FAM135B | NA | NA |
| FAM20C | -0.5086241 | 8.73E-09 |
| FANCI | -1.9906085 | 8.67E-164 |
| FAP | NA | NA |
| FCER2 | -1.7518212 | 4.74E-21 |
| FCGR2A | -0.8183558 | 9.84E-41 |
| FCGR2B | -1.2133485 | 0.01045609 |
| FCGR2C | -2.2252695 | 6.77E-10 |
| FCMR | -1.0761479 | 2.42E-10 |
| FCN1 | NA | NA |
| FCRLA | NA | NA |
| FGFR3 | -2.3452063 | 0.12454596 |
| FRY | 0.53408591 | 2.82E-249 |
| FTH1 | 0.25791231 | 4.51E-16 |
| FUCA1 | 0.92960891 | 8.38E-14 |
| FURIN | 0.10227602 | 0.01437561 |
| FYB1 | -0.1059794 | 0.13111865 |
| GAL | -1.5876377 | 1.02E-104 |
| GAS6 | -0.5615513 | 1.02E-17 |
| GCLM | -0.1094687 | 0.00521841 |
| GM2A | -0.2310272 | 4.78E-05 |
| GNA11 | -0.2818736 | 0.05148139 |
| GNS | 0.79648346 | 3.33E-57 |
| GPC3 | 0.01481728 | 0.01135586 |
| GPR35 | -0.5424772 | 0.08288788 |
| GPR68 | 0.41380276 | 1.03E-05 |
| GPR88 | -0.800052 | 0.11444505 |
| GPT2 | -2.6971602 | 1.54E-126 |
| GRK6 | -0.9356073 | 2.55E-46 |
| GRN | 0.51474367 | 5.83E-25 |
| GSN | 0.28771417 | 3.43E-233 |
| HDAC9 | 0.34634763 | 4.65E-24 |
| HMOX1 | 1.72918496 | 0.00021631 |
| HOMER3 | -0.6015525 | 2.77E-08 |
| HOXC4 | NA | NA |
| HPD | 0.29763296 | 0.19644886 |
| HPS4 | -0.5043696 | 1.65E-25 |
| HSPA7 | -1.7726114 | 4.75E-06 |
| HSPG2 | 0.54155616 | 3.61E-11 |
| IGFBP2 | -2.6699871 | 0.00068253 |
| IQGAP2 | -0.3697289 | 7.04E-14 |
| IQSEC3 | -2.9811972 | 0.00068976 |
| ITGA4 | -0.3754189 | 6.68E-119 |
| ITGA5 | -0.587557 | 1.28E-52 |
| ITGA6 | 1.09453049 | 0.196852 |
| ITGB2 | 0.32678858 | 1.22E-28 |
| ITGB7 | -0.3110539 | 0.00842414 |
| JAML | 0.61242969 | 5.98E-10 |
| JMJD1C | 0.39138938 | 1.49E-18 |
| JUN | -1.2207718 | 6.47E-06 |
| JUNB | 0.60786214 | 3.34E-20 |
| KCNF1 | NA | NA |
| KCTD15 | -0.8082583 | 3.25E-17 |
| KIT | 1.15844774 | 0.41154611 |
| KLHL4 | NA | NA |
| KSR1 | -1.4700274 | 1.64E-23 |
| LGALS3BP | 1.9718263 | 1.79E-16 |
| LILRB2 | 2.86676969 | 1.89E-09 |
| LILRB4 | -0.4783733 | 3.40E-57 |
| LINC00599 | -0.428627 | 1.15E-07 |
| LINC00996 | -2.5271555 | 3.51E-15 |
| LMNA | 0.58876096 | 6.46E-142 |
| LRRK2 | 1.21598753 | 0.00053761 |
| LYZ | 0.42326715 | 8.32E-76 |
| MCM6 | -2.0081241 | 0 |
| MCOLN2 | -0.2567792 | 1.17E-16 |
| ME1 | 0.8659701 | 1.11E-06 |
| METTL7B | -1.5793404 | 1.11E-156 |
| MFAP4 | -4.2848156 | 0.10231102 |
| MIR1915HG | -1.1775318 | 2.35E-06 |
| MIR223HG | -0.4823664 | 6.01E-10 |
| MIR3648 | NA | NA |
| MIR3687 | NA | NA |
| MMP2 | 0.60967835 | 0.10102179 |
| MMP9 | -1.8972878 | 0.01765485 |
| MSC | -1.3533841 | 0.0162598 |
| MT1E | -1.8678377 | 2.24E-59 |
| MT1G | -3.0136403 | 2.24E-49 |
| MT1X | -1.8457037 | 5.42E-06 |
| MT2A | -2.8802987 | 3.48E-40 |
| MYBPH | -1.9849809 | 6.65E-29 |
| MYC | -1.7998532 | 0 |
| MYO10 | -1.4801972 | 9.83E-14 |
| NAPSB | -1.0222461 | 8.59E-29 |
| NCALD | -1.0487773 | 0.18561725 |
| NCF1 | -0.5668744 | 1.99E-13 |
| NCF1B | -0.9118241 | 1.94E-11 |
| NCF1C | -0.9847256 | 2.20E-14 |
| NCR3LG1 | -0.3231632 | 9.21E-16 |
| NDST1 | -0.5472125 | 2.79E-10 |
| NDUFA4L2 | -1.0594924 | 0.00042139 |
| NEMP2 | -2.2512614 | 1.30E-29 |
| NFE2 | -1.4400901 | 3.43E-34 |
| NFXL1 | 0.13457019 | 0.0130687 |
| NHSL2 | -1.3199541 | 0.00021534 |
| NLRC3 | -0.1270934 | 1.16E-05 |
| NLRP12 | -1.5058191 | 8.51E-07 |
| NOG | -0.9970165 | 2.84E-30 |
| NR2F1 | 1.30542498 | 0.62114172 |
| NUCB2 | -1.4390885 | 5.93E-203 |
| P2RY6 | 0.05529272 | 0.37082796 |
| PADI4 | NA | NA |
| PAX5 | -0.9440329 | 1.03E-07 |
| PCK2 | -0.9735207 | 1.86E-63 |
| PHGDH | -3.0807133 | 0 |
| PIK3R3 | 1.09822344 | 2.85E-100 |
| PKIB | 1.05703034 | 2.59E-35 |
| PLCB2 | 0.49751687 | 4.73E-25 |
| PRAM1 | -0.4008967 | 3.84E-21 |
| PRDX1 | -0.3149549 | 4.04E-08 |
| PRLR | NA | NA |
| PRSS57 | -0.8067521 | 1.16E-135 |
| PSAT1 | -3.1734565 | 0 |
| PTAFR | -0.7498505 | 0.00011243 |
| PTCH1 | -0.8513901 | 1.15E-13 |
| PTGS1 | 0.27815094 | 2.74E-09 |
| PTPN14 | -0.7307601 | 2.78E-10 |
| PTPN22 | -0.318421 | 0.00203564 |
| QSOX1 | 0.03252705 | 5.26E-09 |
| RAB37 | -1.1554171 | 1.43E-12 |
| RAP1B | 0.25314917 | 6.24E-06 |
| RASD1 | 1.28786415 | 0.07981196 |
| RASGRP2 | -1.0681566 | 2.73E-63 |
| RASGRP4 | 0.5466949 | 9.71E-21 |
| RBM24 | NA | NA |
| RCBTB2 | -0.2369693 | 4.82E-16 |
| RFC3 | -1.686809 | 3.17E-129 |
| RFLNB | -1.6754866 | 1.53E-153 |
| RFX8 | -0.6355047 | 1.91E-12 |
| RN7SK | NA | NA |
| RNASE3 | -2.5385827 | 2.21E-35 |
| RNF144A | -1.1013039 | 8.82E-75 |
| RNF213-AS1 | 1.58932157 | 0.00300063 |
| RXFP1 | -0.7683715 | 1.55E-33 |
| S100P | -2.9585834 | 0.00382153 |
| SAMD4A | -0.8761788 | 0.47201643 |
| SAMHD1 | 0.78688394 | 2.52E-120 |
| SAMSN1 | 0.18390889 | 0.00764926 |
| SCD | -2.4746645 | 0 |
| SDC2 | -0.585256 | 3.07E-216 |
| SEMA4A | -1.0662651 | 0.06455159 |
| SEMA4C | -0.3942147 | 3.33E-10 |
| SEMA6A | 0.55483583 | 0.10632724 |
| SEMA6B | 0.56143231 | 1.57E-49 |
| SEPTIN11 | -0.5228307 | 1.82E-24 |
| SERPINI1 | 1.88677006 | 1.13E-23 |
| SH3RF3 | -1.3660592 | 6.26E-20 |
| SHISA2 | -3.2625874 | 5.37E-12 |
| SKIDA1 | -0.5570805 | 0.00067559 |
| SLBP | -0.9463074 | 9.45E-57 |
| SLC15A4 | -0.5335904 | 3.78E-05 |
| SLC22A23 | -0.0507626 | 0.08989594 |
| SLC29A1 | -1.7677494 | 3.53E-150 |
| SLC35F2 | -0.4721172 | 1.99E-14 |
| SLC38A5 | -1.7955615 | 1.59E-90 |
| SLC44A1 | -0.4820054 | 5.50E-69 |
| SLC7A5 | -2.6899456 | 0 |
| SLCO4C1 | -1.4266285 | 2.15E-15 |
| SLFN5 | 1.3858146 | 8.06E-20 |
| SLPI | -1.6826651 | 0.01320002 |
| SMIM14 | 1.92497966 | 5.03E-200 |
| SNORD3A | 1.15237139 | 1.02E-07 |
| SORT1 | 0.57259438 | 1.43E-14 |
| SOX4 | -0.1642829 | 0.06736589 |
| SPOCK2 | 1.68803174 | 0.04094409 |
| SRC | 0.03024256 | 0.01036682 |
| SRGN | -0.3854268 | 1.74E-70 |
| STAR | -0.9395629 | 1.28E-13 |
| STC2 | -2.7586603 | 1.63E-150 |
| STRIP2 | -0.8692192 | 1.79E-06 |
| STS | -1.8820496 | 8.12E-48 |
| SUCNR1 | -0.8999593 | 1.06E-76 |
| SYNPO2 | -0.2955495 | 1.03E-55 |
| TBC1D4 | -0.2943451 | 2.91E-06 |
| TCTEX1D1 | -0.1500789 | 0.04226306 |
| TENT5A | 0.81715134 | 1.74E-30 |
| TERT | -3.4901296 | 6.77E-30 |
| TGFBR2 | 0.37892606 | 3.39E-46 |
| TGM5 | 0.14698812 | 0.14064533 |
| THBS4 | -0.4413904 | 8.90E-06 |
| TIMP1 | 0.6557199 | 5.95E-14 |
| TIPARP | 0.17548676 | 5.83E-05 |
| TLR4 | -0.1936135 | 0.00056648 |
| TMOD2 | 0.52289915 | 0.12239888 |
| TNS1 | -2.1742846 | 2.00E-46 |
| TOP2A | -2.1168953 | 0 |
| TPP1 | 0.63824532 | 6.09E-144 |
| TREM2 | 0.10701259 | 0.33642785 |
| TRIB3 | -1.7867504 | 2.21E-85 |
| TRIM14 | -0.8534158 | 5.74E-32 |
| TRIM16 | -1.3417927 | 4.34E-22 |
| TSPAN12 | -0.7742038 | 0.53846757 |
| TSPAN13 | 0.9858911 | 0.37003082 |
| TSPAN32 | 0.02263699 | 0.31574145 |
| TWF1 | 0.99822747 | 1.04E-27 |
| TYROBP | 0.99732773 | 7.09E-40 |
| UNG | -1.3917149 | 7.40E-35 |
| VEGFA | -1.9002631 | 2.23E-79 |
| XBP1 | -1.7762347 | 0 |
| XYLT1 | -1.0119584 | 4.54E-63 |
| ZEB1 | -0.3797415 | 0.03017645 |
| ZFP36L2 | 2.77060192 | 0.07474831 |
| ZNF366 | 2.77060192 | 0.07474831 |

- **Supplementary Table S4. GO Biological Process (BP) enrichment results for core DEGs.**

Enriched BP terms for the 73 core genes, including term description, gene counts, and adjusted p-values.

| **ID** | **Description** | **GeneRatio** | **BgRatio** | **pvalue** | **p.adjust** | **qvalue** | **geneID** | **Count** |
| --- | --- | --- | --- | --- | --- | --- | --- | --- |
| GO:0032350 | regulation of hormone metabolic process | 4/27 | 35/18723 | 1.74134E-07 | 0.000141571 | 0.000105214 | ADM/AKR1C3/GNB3/STC2 | 4 |
| GO:0048385 | regulation of retinoic acid receptor signaling pathway | 2/27 | 16/18723 | 0.000237344 | 0.030789298 | 0.022882187 | AKR1C3/TRIM16 | 2 |
| GO:0042445 | hormone metabolic process | 4/27 | 218/18723 | 0.000254272 | 0.030789298 | 0.022882187 | ADM/AKR1C3/GNB3/STC2 | 4 |
| GO:0002031 | G protein-coupled receptor internalization | 2/27 | 17/18723 | 0.00026875 | 0.030789298 | 0.022882187 | ADM/APLN | 2 |
| GO:0010566 | regulation of ketone biosynthetic process | 2/27 | 17/18723 | 0.00026875 | 0.030789298 | 0.022882187 | ADM/AKR1C3 | 2 |
| GO:0042448 | progesterone metabolic process | 2/27 | 17/18723 | 0.00026875 | 0.030789298 | 0.022882187 | ADM/AKR1C3 | 2 |
| GO:0045618 | positive regulation of keratinocyte differentiation | 2/27 | 19/18723 | 0.000337313 | 0.030789298 | 0.022882187 | ETV4/TRIM16 | 2 |
| GO:1903975 | regulation of glial cell migration | 2/27 | 19/18723 | 0.000337313 | 0.030789298 | 0.022882187 | MIR221/STAP1 | 2 |
| GO:0019218 | regulation of steroid metabolic process | 3/27 | 100/18723 | 0.000393956 | 0.030789298 | 0.022882187 | ADM/AKR1C3/GNB3 | 3 |
| GO:0046885 | regulation of hormone biosynthetic process | 2/27 | 21/18723 | 0.000413508 | 0.030789298 | 0.022882187 | ADM/STC2 | 2 |
| GO:0002029 | desensitization of G protein-coupled receptor signaling pathway | 2/27 | 22/18723 | 0.000454455 | 0.030789298 | 0.022882187 | ADM/APLN | 2 |
| GO:0022401 | negative adaptation of signaling pathway | 2/27 | 22/18723 | 0.000454455 | 0.030789298 | 0.022882187 | ADM/APLN | 2 |
| GO:0023058 | adaptation of signaling pathway | 2/27 | 23/18723 | 0.000497293 | 0.031099969 | 0.023113074 | ADM/APLN | 2 |
| GO:0120255 | olefinic compound biosynthetic process | 2/27 | 24/18723 | 0.00054202 | 0.03147586 | 0.023392431 | ADM/AKR1C3 | 2 |
| GO:0002026 | regulation of the force of heart contraction | 2/27 | 26/18723 | 0.000637114 | 0.032877308 | 0.024433967 | ADM/APLN | 2 |
| GO:0051897 | positive regulation of protein kinase B signaling | 3/27 | 120/18723 | 0.000671214 | 0.032877308 | 0.024433967 | AKR1C3/CHI3L1/MIR221 | 3 |
| GO:0045606 | positive regulation of epidermal cell differentiation | 2/27 | 27/18723 | 0.000687471 | 0.032877308 | 0.024433967 | ETV4/TRIM16 | 2 |
| GO:0010460 | positive regulation of heart rate | 2/27 | 28/18723 | 0.000739696 | 0.033409597 | 0.024829557 | ADM/APLN | 2 |
| GO:0048384 | retinoic acid receptor signaling pathway | 2/27 | 31/18723 | 0.00090752 | 0.0388323 | 0.028859636 | AKR1C3/TRIM16 | 2 |
| GO:0045684 | positive regulation of epidermis development | 2/27 | 32/18723 | 0.000967161 | 0.039315105 | 0.02921845 | ETV4/TRIM16 | 2 |
| GO:0030216 | keratinocyte differentiation | 3/27 | 139/18723 | 0.001027899 | 0.039351981 | 0.029245856 | AKR1C3/ETV4/TRIM16 | 3 |
| GO:0008202 | steroid metabolic process | 4/27 | 319/18723 | 0.001064875 | 0.039351981 | 0.029245856 | ADM/AKR1C3/GNB3/HSD11B1 | 4 |
| GO:0008207 | C21-steroid hormone metabolic process | 2/27 | 36/18723 | 0.001224092 | 0.043268988 | 0.032156922 | ADM/AKR1C3 | 2 |
| GO:0045616 | regulation of keratinocyte differentiation | 2/27 | 37/18723 | 0.001292891 | 0.043796676 | 0.032549093 | ETV4/TRIM16 | 2 |
| GO:0035886 | vascular associated smooth muscle cell differentiation | 2/27 | 38/18723 | 0.001363506 | 0.04434122 | 0.032953791 | ADM/MIR221 | 2 |
| GO:0045823 | positive regulation of heart contraction | 2/27 | 39/18723 | 0.001435933 | 0.044900521 | 0.033369455 | ADM/APLN | 2 |
| GO:1903524 | positive regulation of blood circulation | 2/27 | 41/18723 | 0.001586201 | 0.047762283 | 0.035496278 | ADM/APLN | 2 |
| GO:0042181 | ketone biosynthetic process | 2/27 | 43/18723 | 0.001743656 | 0.050628294 | 0.037626258 | ADM/AKR1C3 | 2 |
| GO:0030324 | lung development | 3/27 | 177/18723 | 0.002056084 | 0.057641257 | 0.042838197 | CHI3L1/DNAAF1/HSD11B1 | 3 |
| GO:0030323 | respiratory tube development | 3/27 | 181/18723 | 0.002191073 | 0.059378067 | 0.04412897 | CHI3L1/DNAAF1/HSD11B1 | 3 |
| GO:0051896 | regulation of protein kinase B signaling | 3/27 | 185/18723 | 0.002331467 | 0.06014723 | 0.044700602 | AKR1C3/CHI3L1/MIR221 | 3 |
| GO:0008217 | regulation of blood pressure | 3/27 | 186/18723 | 0.002367419 | 0.06014723 | 0.044700602 | ADM/APLN/GNB3 | 3 |
| GO:0006636 | unsaturated fatty acid biosynthetic process | 2/27 | 51/18723 | 0.002444549 | 0.060224788 | 0.044758242 | AKR1C3/SCD | 2 |
| GO:0008347 | glial cell migration | 2/27 | 53/18723 | 0.002637344 | 0.061261744 | 0.045528894 | MIR221/STAP1 | 2 |
| GO:0045744 | negative regulation of G protein-coupled receptor signaling pathway | 2/27 | 53/18723 | 0.002637344 | 0.061261744 | 0.045528894 | ADM/APLN | 2 |
| GO:0009913 | epidermal cell differentiation | 3/27 | 202/18723 | 0.002990168 | 0.066624599 | 0.049514494 | AKR1C3/ETV4/TRIM16 | 3 |
| GO:0060541 | respiratory system development | 3/27 | 203/18723 | 0.003032116 | 0.066624599 | 0.049514494 | CHI3L1/DNAAF1/HSD11B1 | 3 |
| GO:0045604 | regulation of epidermal cell differentiation | 2/27 | 58/18723 | 0.003149661 | 0.067386176 | 0.050080488 | ETV4/TRIM16 | 2 |
| GO:0031102 | neuron projection regeneration | 2/27 | 60/18723 | 0.003366618 | 0.068715519 | 0.051068437 | ADM/MIR221 | 2 |
| GO:0043491 | protein kinase B signaling | 3/27 | 211/18723 | 0.003380837 | 0.068715519 | 0.051068437 | AKR1C3/CHI3L1/MIR221 | 3 |
| GO:0030858 | positive regulation of epithelial cell differentiation | 2/27 | 64/18723 | 0.003820943 | 0.073318775 | 0.054489515 | ETV4/TRIM16 | 2 |
| GO:0070542 | response to fatty acid | 2/27 | 64/18723 | 0.003820943 | 0.073318775 | 0.054489515 | AKR1C3/SCD | 2 |
| GO:0045682 | regulation of epidermis development | 2/27 | 65/18723 | 0.003938749 | 0.073318775 | 0.054489515 | ETV4/TRIM16 | 2 |
| GO:0019229 | regulation of vasoconstriction | 2/27 | 66/18723 | 0.004058235 | 0.073318775 | 0.054489515 | ADM/APLN | 2 |
| GO:0042446 | hormone biosynthetic process | 2/27 | 66/18723 | 0.004058235 | 0.073318775 | 0.054489515 | ADM/STC2 | 2 |
| GO:0050810 | regulation of steroid biosynthetic process | 2/27 | 69/18723 | 0.004426729 | 0.078237622 | 0.058145135 | ADM/AKR1C3 | 2 |
| GO:0051145 | smooth muscle cell differentiation | 2/27 | 73/18723 | 0.004941295 | 0.085473882 | 0.063523025 | ADM/MIR221 | 2 |
| GO:0043588 | skin development | 3/27 | 263/18723 | 0.006247004 | 0.105808627 | 0.078635531 | AKR1C3/ETV4/TRIM16 | 3 |
| GO:0042310 | vasoconstriction | 2/27 | 84/18723 | 0.006490997 | 0.107697559 | 0.080039359 | ADM/APLN | 2 |
| GO:1904705 | regulation of vascular associated smooth muscle cell proliferation | 2/27 | 90/18723 | 0.007417883 | 0.120614778 | 0.08963926 | APLN/MIR221 | 2 |
| GO:1990874 | vascular associated smooth muscle cell proliferation | 2/27 | 91/18723 | 0.007577868 | 0.120800133 | 0.089777013 | APLN/MIR221 | 2 |
| GO:0003073 | regulation of systemic arterial blood pressure | 2/27 | 96/18723 | 0.008401112 | 0.13134816 | 0.097616163 | ADM/APLN | 2 |
| GO:0045807 | positive regulation of endocytosis | 2/27 | 100/18723 | 0.009087422 | 0.139397631 | 0.103598421 | APLN/STAP1 | 2 |
| GO:0002027 | regulation of heart rate | 2/27 | 103/18723 | 0.009618136 | 0.143951714 | 0.106982953 | ADM/APLN | 2 |
| GO:0003014 | renal system process | 2/27 | 110/18723 | 0.010909018 | 0.143951714 | 0.106982953 | ADM/AKR1C3 | 2 |
| GO:0008544 | epidermis development | 3/27 | 324/18723 | 0.011042743 | 0.143951714 | 0.106982953 | AKR1C3/ETV4/TRIM16 | 3 |
| GO:0031623 | receptor internalization | 2/27 | 113/18723 | 0.011484492 | 0.143951714 | 0.106982953 | ADM/APLN | 2 |
| GO:0019216 | regulation of lipid metabolic process | 3/27 | 331/18723 | 0.011697981 | 0.143951714 | 0.106982953 | ADM/AKR1C3/GNB3 | 3 |
| GO:0062012 | regulation of small molecule metabolic process | 3/27 | 334/18723 | 0.011985579 | 0.143951714 | 0.106982953 | ADM/AKR1C3/GNB3 | 3 |
| GO:0033559 | unsaturated fatty acid metabolic process | 2/27 | 116/18723 | 0.012073142 | 0.143951714 | 0.106982953 | AKR1C3/SCD | 2 |
| GO:0045765 | regulation of angiogenesis | 3/27 | 342/18723 | 0.012772498 | 0.143951714 | 0.106982953 | ADM/CHI3L1/MIR221 | 3 |
| GO:1901342 | regulation of vasculature development | 3/27 | 348/18723 | 0.013381858 | 0.143951714 | 0.106982953 | ADM/CHI3L1/MIR221 | 3 |
| GO:0001909 | leukocyte mediated cytotoxicity | 2/27 | 124/18723 | 0.013706404 | 0.143951714 | 0.106982953 | STAP1/ULBP1 | 2 |
| GO:0008300 | isoprenoid catabolic process | 1/27 | 10/18723 | 0.014330966 | 0.143951714 | 0.106982953 | AKR1C3 | 1 |
| GO:0030638 | polyketide metabolic process | 1/27 | 10/18723 | 0.014330966 | 0.143951714 | 0.106982953 | AKR1C3 | 1 |
| GO:0030647 | aminoglycoside antibiotic metabolic process | 1/27 | 10/18723 | 0.014330966 | 0.143951714 | 0.106982953 | AKR1C3 | 1 |
| GO:0042756 | drinking behavior | 1/27 | 10/18723 | 0.014330966 | 0.143951714 | 0.106982953 | APLN | 1 |
| GO:0044598 | doxorubicin metabolic process | 1/27 | 10/18723 | 0.014330966 | 0.143951714 | 0.106982953 | AKR1C3 | 1 |
| GO:0050861 | positive regulation of B cell receptor signaling pathway | 1/27 | 10/18723 | 0.014330966 | 0.143951714 | 0.106982953 | STAP1 | 1 |
| GO:0097084 | vascular associated smooth muscle cell development | 1/27 | 10/18723 | 0.014330966 | 0.143951714 | 0.106982953 | ADM | 1 |
| GO:1903980 | positive regulation of microglial cell activation | 1/27 | 10/18723 | 0.014330966 | 0.143951714 | 0.106982953 | STAP1 | 1 |
| GO:0010565 | regulation of cellular ketone metabolic process | 2/27 | 133/18723 | 0.015652127 | 0.143951714 | 0.106982953 | ADM/AKR1C3 | 2 |
| GO:0006751 | glutathione catabolic process | 1/27 | 11/18723 | 0.015753136 | 0.143951714 | 0.106982953 | CHAC1 | 1 |
| GO:0014010 | Schwann cell proliferation | 1/27 | 11/18723 | 0.015753136 | 0.143951714 | 0.106982953 | MIR221 | 1 |
| GO:0030656 | regulation of vitamin metabolic process | 1/27 | 11/18723 | 0.015753136 | 0.143951714 | 0.106982953 | AKR1C3 | 1 |
| GO:0031652 | positive regulation of heat generation | 1/27 | 11/18723 | 0.015753136 | 0.143951714 | 0.106982953 | APLN | 1 |
| GO:0032351 | negative regulation of hormone metabolic process | 1/27 | 11/18723 | 0.015753136 | 0.143951714 | 0.106982953 | AKR1C3 | 1 |
| GO:0034115 | negative regulation of heterotypic cell-cell adhesion | 1/27 | 11/18723 | 0.015753136 | 0.143951714 | 0.106982953 | MIR221 | 1 |
| GO:0048680 | positive regulation of axon regeneration | 1/27 | 11/18723 | 0.015753136 | 0.143951714 | 0.106982953 | MIR221 | 1 |
| GO:0070587 | regulation of cell-cell adhesion involved in gastrulation | 1/27 | 11/18723 | 0.015753136 | 0.143951714 | 0.106982953 | MIR221 | 1 |
| GO:1902033 | regulation of hematopoietic stem cell proliferation | 1/27 | 11/18723 | 0.015753136 | 0.143951714 | 0.106982953 | MIR221 | 1 |
| GO:1905522 | negative regulation of macrophage migration | 1/27 | 11/18723 | 0.015753136 | 0.143951714 | 0.106982953 | STAP1 | 1 |
| GO:0008277 | regulation of G protein-coupled receptor signaling pathway | 2/27 | 134/18723 | 0.015875272 | 0.143951714 | 0.106982953 | ADM/APLN | 2 |
| GO:0034754 | cellular hormone metabolic process | 2/27 | 137/18723 | 0.016552941 | 0.143951714 | 0.106982953 | ADM/AKR1C3 | 2 |
| GO:0043696 | dedifferentiation | 1/27 | 12/18723 | 0.01717333 | 0.143951714 | 0.106982953 | MIR221 | 1 |
| GO:0043697 | cell dedifferentiation | 1/27 | 12/18723 | 0.01717333 | 0.143951714 | 0.106982953 | MIR221 | 1 |
| GO:0060287 | epithelial cilium movement involved in determination of left/right asymmetry | 1/27 | 12/18723 | 0.01717333 | 0.143951714 | 0.106982953 | DNAAF1 | 1 |
| GO:0070572 | positive regulation of neuron projection regeneration | 1/27 | 12/18723 | 0.01717333 | 0.143951714 | 0.106982953 | MIR221 | 1 |
| GO:0070586 | cell-cell adhesion involved in gastrulation | 1/27 | 12/18723 | 0.01717333 | 0.143951714 | 0.106982953 | MIR221 | 1 |
| GO:0090677 | reversible differentiation | 1/27 | 12/18723 | 0.01717333 | 0.143951714 | 0.106982953 | MIR221 | 1 |
| GO:2001214 | positive regulation of vasculogenesis | 1/27 | 12/18723 | 0.01717333 | 0.143951714 | 0.106982953 | ADM | 1 |
| GO:0035296 | regulation of tube diameter | 2/27 | 141/18723 | 0.017475565 | 0.143951714 | 0.106982953 | ADM/APLN | 2 |
| GO:0097746 | blood vessel diameter maintenance | 2/27 | 141/18723 | 0.017475565 | 0.143951714 | 0.106982953 | ADM/APLN | 2 |
| GO:0035150 | regulation of tube size | 2/27 | 142/18723 | 0.017709598 | 0.143951714 | 0.106982953 | ADM/APLN | 2 |
| GO:0001890 | placenta development | 2/27 | 144/18723 | 0.018181688 | 0.143951714 | 0.106982953 | ADM/STC2 | 2 |
| GO:0032352 | positive regulation of hormone metabolic process | 1/27 | 13/18723 | 0.01859155 | 0.143951714 | 0.106982953 | ADM | 1 |
| GO:0036005 | response to macrophage colony-stimulating factor | 1/27 | 13/18723 | 0.01859155 | 0.143951714 | 0.106982953 | STAP1 | 1 |
| GO:0036006 | cellular response to macrophage colony-stimulating factor stimulus | 1/27 | 13/18723 | 0.01859155 | 0.143951714 | 0.106982953 | STAP1 | 1 |
| GO:0036462 | TRAIL-activated apoptotic signaling pathway | 1/27 | 13/18723 | 0.01859155 | 0.143951714 | 0.106982953 | MIR221 | 1 |
| GO:0060100 | positive regulation of phagocytosis, engulfment | 1/27 | 13/18723 | 0.01859155 | 0.143951714 | 0.106982953 | STAP1 | 1 |
| GO:0060670 | branching involved in labyrinthine layer morphogenesis | 1/27 | 13/18723 | 0.01859155 | 0.143951714 | 0.106982953 | ADM | 1 |
| GO:1903977 | positive regulation of glial cell migration | 1/27 | 13/18723 | 0.01859155 | 0.143951714 | 0.106982953 | MIR221 | 1 |
| GO:1904995 | negative regulation of leukocyte adhesion to vascular endothelial cell | 1/27 | 13/18723 | 0.01859155 | 0.143951714 | 0.106982953 | MIR221 | 1 |
| GO:1905064 | negative regulation of vascular associated smooth muscle cell differentiation | 1/27 | 13/18723 | 0.01859155 | 0.143951714 | 0.106982953 | MIR221 | 1 |
| GO:1905155 | positive regulation of membrane invagination | 1/27 | 13/18723 | 0.01859155 | 0.143951714 | 0.106982953 | STAP1 | 1 |
| GO:0031650 | regulation of heat generation | 1/27 | 14/18723 | 0.020007799 | 0.147647221 | 0.109729404 | APLN | 1 |
| GO:0046348 | amino sugar catabolic process | 1/27 | 14/18723 | 0.020007799 | 0.147647221 | 0.109729404 | CHI3L1 | 1 |
| GO:0060354 | negative regulation of cell adhesion molecule production | 1/27 | 14/18723 | 0.020007799 | 0.147647221 | 0.109729404 | MIR221 | 1 |
| GO:0072531 | pyrimidine-containing compound transmembrane transport | 1/27 | 14/18723 | 0.020007799 | 0.147647221 | 0.109729404 | SLC28A3 | 1 |
| GO:0120254 | olefinic compound metabolic process | 2/27 | 153/18723 | 0.02037169 | 0.147647221 | 0.109729404 | ADM/AKR1C3 | 2 |
| GO:0030856 | regulation of epithelial cell differentiation | 2/27 | 154/18723 | 0.02062157 | 0.147647221 | 0.109729404 | ETV4/TRIM16 | 2 |
| GO:0043434 | response to peptide hormone | 3/27 | 414/18723 | 0.021187343 | 0.147647221 | 0.109729404 | ADM/STC2/TRIM16 | 3 |
| GO:0003356 | regulation of cilium beat frequency | 1/27 | 15/18723 | 0.021422081 | 0.147647221 | 0.109729404 | DNAAF1 | 1 |
| GO:0016114 | terpenoid biosynthetic process | 1/27 | 15/18723 | 0.021422081 | 0.147647221 | 0.109729404 | AKR1C3 | 1 |
| GO:0034310 | primary alcohol catabolic process | 1/27 | 15/18723 | 0.021422081 | 0.147647221 | 0.109729404 | AKR1C3 | 1 |
| GO:0044793 | negative regulation by host of viral process | 1/27 | 15/18723 | 0.021422081 | 0.147647221 | 0.109729404 | MIR221 | 1 |
| GO:0060099 | regulation of phagocytosis, engulfment | 1/27 | 15/18723 | 0.021422081 | 0.147647221 | 0.109729404 | STAP1 | 1 |
| GO:0048608 | reproductive structure development | 3/27 | 424/18723 | 0.022548401 | 0.147647221 | 0.109729404 | ADM/AKR1C3/STC2 | 3 |
| GO:0006750 | glutathione biosynthetic process | 1/27 | 16/18723 | 0.022834396 | 0.147647221 | 0.109729404 | CHAC1 | 1 |
| GO:0036159 | inner dynein arm assembly | 1/27 | 16/18723 | 0.022834396 | 0.147647221 | 0.109729404 | DNAAF1 | 1 |
| GO:0042574 | retinal metabolic process | 1/27 | 16/18723 | 0.022834396 | 0.147647221 | 0.109729404 | AKR1C3 | 1 |
| GO:1905153 | regulation of membrane invagination | 1/27 | 16/18723 | 0.022834396 | 0.147647221 | 0.109729404 | STAP1 | 1 |
| GO:2001212 | regulation of vasculogenesis | 1/27 | 16/18723 | 0.022834396 | 0.147647221 | 0.109729404 | ADM | 1 |
| GO:0006633 | fatty acid biosynthetic process | 2/27 | 163/18723 | 0.022928269 | 0.147647221 | 0.109729404 | AKR1C3/SCD | 2 |
| GO:0061458 | reproductive system development | 3/27 | 427/18723 | 0.022965939 | 0.147647221 | 0.109729404 | ADM/AKR1C3/STC2 | 3 |
| GO:0043112 | receptor metabolic process | 2/27 | 166/18723 | 0.023719976 | 0.147647221 | 0.109729404 | ADM/APLN | 2 |
| GO:0031960 | response to corticosteroid | 2/27 | 167/18723 | 0.023986378 | 0.147647221 | 0.109729404 | ADM/AKR1C3 | 2 |
| GO:0036166 | phenotypic switching | 1/27 | 17/18723 | 0.024244749 | 0.147647221 | 0.109729404 | MIR221 | 1 |
| GO:0070293 | renal absorption | 1/27 | 17/18723 | 0.024244749 | 0.147647221 | 0.109729404 | AKR1C3 | 1 |
| GO:0046890 | regulation of lipid biosynthetic process | 2/27 | 171/18723 | 0.025064388 | 0.147647221 | 0.109729404 | ADM/AKR1C3 | 2 |
| GO:0006694 | steroid biosynthetic process | 2/27 | 173/18723 | 0.025610779 | 0.147647221 | 0.109729404 | ADM/AKR1C3 | 2 |
| GO:0007250 | activation of NF-kappaB-inducing kinase activity | 1/27 | 18/18723 | 0.025653142 | 0.147647221 | 0.109729404 | CHI3L1 | 1 |
| GO:0019184 | nonribosomal peptide biosynthetic process | 1/27 | 18/18723 | 0.025653142 | 0.147647221 | 0.109729404 | CHAC1 | 1 |
| GO:0031649 | heat generation | 1/27 | 18/18723 | 0.025653142 | 0.147647221 | 0.109729404 | APLN | 1 |
| GO:0043116 | negative regulation of vascular permeability | 1/27 | 18/18723 | 0.025653142 | 0.147647221 | 0.109729404 | ADM | 1 |
| GO:0060546 | negative regulation of necroptotic process | 1/27 | 18/18723 | 0.025653142 | 0.147647221 | 0.109729404 | MIR221 | 1 |
| GO:0150078 | positive regulation of neuroinflammatory response | 1/27 | 18/18723 | 0.025653142 | 0.147647221 | 0.109729404 | STAP1 | 1 |
| GO:0001659 | temperature homeostasis | 2/27 | 174/18723 | 0.025885807 | 0.147647221 | 0.109729404 | APLN/SCD | 2 |
| GO:0007584 | response to nutrient | 2/27 | 174/18723 | 0.025885807 | 0.147647221 | 0.109729404 | AKR1C3/STC2 | 2 |
| GO:0003341 | cilium movement | 2/27 | 175/18723 | 0.026162052 | 0.147647221 | 0.109729404 | DNAAF1/ZBBX | 2 |
| GO:0002888 | positive regulation of myeloid leukocyte mediated immunity | 1/27 | 19/18723 | 0.027059577 | 0.147647221 | 0.109729404 | STAP1 | 1 |
| GO:0003085 | negative regulation of systemic arterial blood pressure | 1/27 | 19/18723 | 0.027059577 | 0.147647221 | 0.109729404 | APLN | 1 |
| GO:0032011 | ARF protein signal transduction | 1/27 | 19/18723 | 0.027059577 | 0.147647221 | 0.109729404 | IQSEC3 | 1 |
| GO:0032012 | regulation of ARF protein signal transduction | 1/27 | 19/18723 | 0.027059577 | 0.147647221 | 0.109729404 | IQSEC3 | 1 |
| GO:0035313 | wound healing, spreading of epidermal cells | 1/27 | 19/18723 | 0.027059577 | 0.147647221 | 0.109729404 | MIR221 | 1 |
| GO:0044827 | modulation by host of viral genome replication | 1/27 | 19/18723 | 0.027059577 | 0.147647221 | 0.109729404 | MIR221 | 1 |
| GO:0062099 | negative regulation of programmed necrotic cell death | 1/27 | 19/18723 | 0.027059577 | 0.147647221 | 0.109729404 | MIR221 | 1 |
| GO:1903978 | regulation of microglial cell activation | 1/27 | 19/18723 | 0.027059577 | 0.147647221 | 0.109729404 | STAP1 | 1 |
| GO:2000251 | positive regulation of actin cytoskeleton reorganization | 1/27 | 19/18723 | 0.027059577 | 0.147647221 | 0.109729404 | STAP1 | 1 |
| GO:0048660 | regulation of smooth muscle cell proliferation | 2/27 | 180/18723 | 0.0275614 | 0.148341526 | 0.110245402 | APLN/MIR221 | 2 |
| GO:0045766 | positive regulation of angiogenesis | 2/27 | 181/18723 | 0.027844867 | 0.148341526 | 0.110245402 | ADM/CHI3L1 | 2 |
| GO:1904018 | positive regulation of vasculature development | 2/27 | 181/18723 | 0.027844867 | 0.148341526 | 0.110245402 | ADM/CHI3L1 | 2 |
| GO:0006700 | C21-steroid hormone biosynthetic process | 1/27 | 20/18723 | 0.028464057 | 0.148341526 | 0.110245402 | ADM | 1 |
| GO:0010893 | positive regulation of steroid biosynthetic process | 1/27 | 20/18723 | 0.028464057 | 0.148341526 | 0.110245402 | ADM | 1 |
| GO:0060252 | positive regulation of glial cell proliferation | 1/27 | 20/18723 | 0.028464057 | 0.148341526 | 0.110245402 | MIR221 | 1 |
| GO:0071379 | cellular response to prostaglandin stimulus | 1/27 | 20/18723 | 0.028464057 | 0.148341526 | 0.110245402 | AKR1C3 | 1 |
| GO:0048659 | smooth muscle cell proliferation | 2/27 | 184/18723 | 0.028702397 | 0.148630884 | 0.110460449 | APLN/MIR221 | 2 |
| GO:0007369 | gastrulation | 2/27 | 185/18723 | 0.028990604 | 0.149173171 | 0.110863469 | APLN/MIR221 | 2 |
| GO:0001906 | cell killing | 2/27 | 188/18723 | 0.029862263 | 0.149870658 | 0.111381832 | STAP1/ULBP1 | 2 |
| GO:0002689 | negative regulation of leukocyte chemotaxis | 1/27 | 21/18723 | 0.029866584 | 0.149870658 | 0.111381832 | STAP1 | 1 |
| GO:0060972 | left/right pattern formation | 1/27 | 21/18723 | 0.029866584 | 0.149870658 | 0.111381832 | DNAAF1 | 1 |
| GO:1902644 | tertiary alcohol metabolic process | 1/27 | 21/18723 | 0.029866584 | 0.149870658 | 0.111381832 | AKR1C3 | 1 |
| GO:0031667 | response to nutrient levels | 3/27 | 474/18723 | 0.030063517 | 0.149870658 | 0.111381832 | ADM/AKR1C3/STC2 | 3 |
| GO:0016137 | glycoside metabolic process | 1/27 | 22/18723 | 0.031267162 | 0.149870658 | 0.111381832 | AKR1C3 | 1 |
| GO:0035809 | regulation of urine volume | 1/27 | 22/18723 | 0.031267162 | 0.149870658 | 0.111381832 | ADM | 1 |
| GO:0036158 | outer dynein arm assembly | 1/27 | 22/18723 | 0.031267162 | 0.149870658 | 0.111381832 | DNAAF1 | 1 |
| GO:0051151 | negative regulation of smooth muscle cell differentiation | 1/27 | 22/18723 | 0.031267162 | 0.149870658 | 0.111381832 | MIR221 | 1 |
| GO:0060713 | labyrinthine layer morphogenesis | 1/27 | 22/18723 | 0.031267162 | 0.149870658 | 0.111381832 | ADM | 1 |
| GO:0007565 | female pregnancy | 2/27 | 193/18723 | 0.031338268 | 0.149870658 | 0.111381832 | ADM/STC2 | 2 |
| GO:0050731 | positive regulation of peptidyl-tyrosine phosphorylation | 2/27 | 193/18723 | 0.031338268 | 0.149870658 | 0.111381832 | MIR221/STAP1 | 2 |
| GO:0042594 | response to starvation | 2/27 | 197/18723 | 0.032539723 | 0.153455733 | 0.11404621 | ADM/AKR1C3 | 2 |
| GO:0060353 | regulation of cell adhesion molecule production | 1/27 | 23/18723 | 0.032665792 | 0.153455733 | 0.11404621 | MIR221 | 1 |
| GO:0060547 | negative regulation of necrotic cell death | 1/27 | 23/18723 | 0.032665792 | 0.153455733 | 0.11404621 | MIR221 | 1 |
| GO:0031099 | regeneration | 2/27 | 198/18723 | 0.032842924 | 0.153455733 | 0.11404621 | ADM/MIR221 | 2 |
| GO:0040037 | negative regulation of fibroblast growth factor receptor signaling pathway | 1/27 | 24/18723 | 0.034062478 | 0.153785691 | 0.114291431 | APLN | 1 |
| GO:0050855 | regulation of B cell receptor signaling pathway | 1/27 | 24/18723 | 0.034062478 | 0.153785691 | 0.114291431 | STAP1 | 1 |
| GO:0072530 | purine-containing compound transmembrane transport | 1/27 | 24/18723 | 0.034062478 | 0.153785691 | 0.114291431 | SLC28A3 | 1 |
| GO:2000353 | positive regulation of endothelial cell apoptotic process | 1/27 | 24/18723 | 0.034062478 | 0.153785691 | 0.114291431 | AKR1C3 | 1 |
| GO:0008016 | regulation of heart contraction | 2/27 | 206/18723 | 0.035308835 | 0.153785691 | 0.114291431 | ADM/APLN | 2 |
| GO:1904754 | positive regulation of vascular associated smooth muscle cell migration | 1/27 | 25/18723 | 0.035457222 | 0.153785691 | 0.114291431 | MIR221 | 1 |
| GO:1905564 | positive regulation of vascular endothelial cell proliferation | 1/27 | 25/18723 | 0.035457222 | 0.153785691 | 0.114291431 | APLN | 1 |
| GO:0045637 | regulation of myeloid cell differentiation | 2/27 | 210/18723 | 0.036568284 | 0.153785691 | 0.114291431 | MIR221/PRXL2A | 2 |
| GO:0002092 | positive regulation of receptor internalization | 1/27 | 26/18723 | 0.036850026 | 0.153785691 | 0.114291431 | APLN | 1 |
| GO:0006706 | steroid catabolic process | 1/27 | 26/18723 | 0.036850026 | 0.153785691 | 0.114291431 | HSD11B1 | 1 |
| GO:0034114 | regulation of heterotypic cell-cell adhesion | 1/27 | 26/18723 | 0.036850026 | 0.153785691 | 0.114291431 | MIR221 | 1 |
| GO:0046697 | decidualization | 1/27 | 26/18723 | 0.036850026 | 0.153785691 | 0.114291431 | STC2 | 1 |
| GO:0050857 | positive regulation of antigen receptor-mediated signaling pathway | 1/27 | 26/18723 | 0.036850026 | 0.153785691 | 0.114291431 | STAP1 | 1 |
| GO:0060352 | cell adhesion molecule production | 1/27 | 26/18723 | 0.036850026 | 0.153785691 | 0.114291431 | MIR221 | 1 |
| GO:0060544 | regulation of necroptotic process | 1/27 | 26/18723 | 0.036850026 | 0.153785691 | 0.114291431 | MIR221 | 1 |
| GO:0060669 | embryonic placenta morphogenesis | 1/27 | 26/18723 | 0.036850026 | 0.153785691 | 0.114291431 | ADM | 1 |
| GO:0071425 | hematopoietic stem cell proliferation | 1/27 | 26/18723 | 0.036850026 | 0.153785691 | 0.114291431 | MIR221 | 1 |
| GO:1901071 | glucosamine-containing compound metabolic process | 1/27 | 26/18723 | 0.036850026 | 0.153785691 | 0.114291431 | CHI3L1 | 1 |
| GO:1905063 | regulation of vascular associated smooth muscle cell differentiation | 1/27 | 26/18723 | 0.036850026 | 0.153785691 | 0.114291431 | MIR221 | 1 |
| GO:0030100 | regulation of endocytosis | 2/27 | 211/18723 | 0.036885867 | 0.153785691 | 0.114291431 | APLN/STAP1 | 2 |
| GO:0042180 | cellular ketone metabolic process | 2/27 | 211/18723 | 0.036885867 | 0.153785691 | 0.114291431 | ADM/AKR1C3 | 2 |
| GO:0072330 | monocarboxylic acid biosynthetic process | 2/27 | 214/18723 | 0.037845091 | 0.155449232 | 0.115527752 | AKR1C3/SCD | 2 |
| GO:0001835 | blastocyst hatching | 1/27 | 27/18723 | 0.038240893 | 0.155449232 | 0.115527752 | CCDC24 | 1 |
| GO:0010758 | regulation of macrophage chemotaxis | 1/27 | 27/18723 | 0.038240893 | 0.155449232 | 0.115527752 | STAP1 | 1 |
| GO:0035188 | hatching | 1/27 | 27/18723 | 0.038240893 | 0.155449232 | 0.115527752 | CCDC24 | 1 |
| GO:0071684 | organism emergence from protective structure | 1/27 | 27/18723 | 0.038240893 | 0.155449232 | 0.115527752 | CCDC24 | 1 |
| GO:0070374 | positive regulation of ERK1 and ERK2 cascade | 2/27 | 217/18723 | 0.038813952 | 0.156993746 | 0.116675613 | CHI3L1/MIR221 | 2 |
| GO:0008299 | isoprenoid biosynthetic process | 1/27 | 28/18723 | 0.039629827 | 0.158584243 | 0.11785765 | AKR1C3 | 1 |
| GO:1903671 | negative regulation of sprouting angiogenesis | 1/27 | 28/18723 | 0.039629827 | 0.158584243 | 0.11785765 | MIR221 | 1 |
| GO:0044706 | multi-multicellular organism process | 2/27 | 220/18723 | 0.039792356 | 0.158584243 | 0.11785765 | ADM/STC2 | 2 |
| GO:0010955 | negative regulation of protein processing | 1/27 | 29/18723 | 0.041016828 | 0.160320583 | 0.119148074 | CHAC1 | 1 |
| GO:0048679 | regulation of axon regeneration | 1/27 | 29/18723 | 0.041016828 | 0.160320583 | 0.119148074 | MIR221 | 1 |
| GO:0062098 | regulation of programmed necrotic cell death | 1/27 | 29/18723 | 0.041016828 | 0.160320583 | 0.119148074 | MIR221 | 1 |
| GO:1903318 | negative regulation of protein maturation | 1/27 | 29/18723 | 0.041016828 | 0.160320583 | 0.119148074 | CHAC1 | 1 |
| GO:0006884 | cell volume homeostasis | 1/27 | 30/18723 | 0.042401901 | 0.16033835 | 0.119161278 | GNB3 | 1 |
| GO:0008209 | androgen metabolic process | 1/27 | 30/18723 | 0.042401901 | 0.16033835 | 0.119161278 | ADM | 1 |
| GO:0010800 | positive regulation of peptidyl-threonine phosphorylation | 1/27 | 30/18723 | 0.042401901 | 0.16033835 | 0.119161278 | CHI3L1 | 1 |
| GO:0034694 | response to prostaglandin | 1/27 | 30/18723 | 0.042401901 | 0.16033835 | 0.119161278 | AKR1C3 | 1 |
| GO:0043032 | positive regulation of macrophage activation | 1/27 | 30/18723 | 0.042401901 | 0.16033835 | 0.119161278 | STAP1 | 1 |
| GO:0044788 | modulation by host of viral process | 1/27 | 30/18723 | 0.042401901 | 0.16033835 | 0.119161278 | MIR221 | 1 |
| GO:0045940 | positive regulation of steroid metabolic process | 1/27 | 30/18723 | 0.042401901 | 0.16033835 | 0.119161278 | ADM | 1 |
| GO:0001516 | prostaglandin biosynthetic process | 1/27 | 31/18723 | 0.043785047 | 0.16180565 | 0.120251755 | AKR1C3 | 1 |
| GO:0043171 | peptide catabolic process | 1/27 | 31/18723 | 0.043785047 | 0.16180565 | 0.120251755 | CHAC1 | 1 |
| GO:0046457 | prostanoid biosynthetic process | 1/27 | 31/18723 | 0.043785047 | 0.16180565 | 0.120251755 | AKR1C3 | 1 |
| GO:1900027 | regulation of ruffle assembly | 1/27 | 31/18723 | 0.043785047 | 0.16180565 | 0.120251755 | STAP1 | 1 |
| GO:1902042 | negative regulation of extrinsic apoptotic signaling pathway via death domain receptors | 1/27 | 31/18723 | 0.043785047 | 0.16180565 | 0.120251755 | MIR221 | 1 |
| GO:0007188 | adenylate cyclase-modulating G protein-coupled receptor signaling pathway | 2/27 | 233/18723 | 0.044140108 | 0.162379675 | 0.120678362 | ADM/OXER1 | 2 |
| GO:0006026 | aminoglycan catabolic process | 1/27 | 32/18723 | 0.045166269 | 0.164664469 | 0.122376391 | CHI3L1 | 1 |
| GO:0042219 | cellular modified amino acid catabolic process | 1/27 | 32/18723 | 0.045166269 | 0.164664469 | 0.122376391 | CHAC1 | 1 |
| GO:0045648 | positive regulation of erythrocyte differentiation | 1/27 | 33/18723 | 0.04654557 | 0.167440478 | 0.124439483 | MIR221 | 1 |
| GO:0070570 | regulation of neuron projection regeneration | 1/27 | 33/18723 | 0.04654557 | 0.167440478 | 0.124439483 | MIR221 | 1 |
| GO:0120033 | negative regulation of plasma membrane bounded cell projection assembly | 1/27 | 33/18723 | 0.04654557 | 0.167440478 | 0.124439483 | STAP1 | 1 |
| GO:0060047 | heart contraction | 2/27 | 241/18723 | 0.046900589 | 0.167931972 | 0.124804754 | ADM/APLN | 2 |
| GO:0042573 | retinoic acid metabolic process | 1/27 | 34/18723 | 0.047922952 | 0.167931972 | 0.124804754 | AKR1C3 | 1 |
| GO:0044319 | wound healing, spreading of cells | 1/27 | 34/18723 | 0.047922952 | 0.167931972 | 0.124804754 | MIR221 | 1 |
| GO:0090505 | epiboly involved in wound healing | 1/27 | 34/18723 | 0.047922952 | 0.167931972 | 0.124804754 | MIR221 | 1 |
| GO:2000780 | negative regulation of double-strand break repair | 1/27 | 34/18723 | 0.047922952 | 0.167931972 | 0.124804754 | MIR221 | 1 |
| GO:0006898 | receptor-mediated endocytosis | 2/27 | 244/18723 | 0.047952011 | 0.167931972 | 0.124804754 | ADM/APLN | 2 |
| GO:0001893 | maternal placenta development | 1/27 | 35/18723 | 0.049298417 | 0.167931972 | 0.124804754 | STC2 | 1 |
| GO:0003351 | epithelial cilium movement involved in extracellular fluid movement | 1/27 | 35/18723 | 0.049298417 | 0.167931972 | 0.124804754 | DNAAF1 | 1 |
| GO:0033280 | response to vitamin D | 1/27 | 35/18723 | 0.049298417 | 0.167931972 | 0.124804754 | STC2 | 1 |
| GO:0044273 | sulfur compound catabolic process | 1/27 | 35/18723 | 0.049298417 | 0.167931972 | 0.124804754 | CHAC1 | 1 |
| GO:0090181 | regulation of cholesterol metabolic process | 1/27 | 35/18723 | 0.049298417 | 0.167931972 | 0.124804754 | GNB3 | 1 |
| GO:0090504 | epiboly | 1/27 | 35/18723 | 0.049298417 | 0.167931972 | 0.124804754 | MIR221 | 1 |
| GO:0033002 | muscle cell proliferation | 2/27 | 248/18723 | 0.049367455 | 0.167931972 | 0.124804754 | APLN/MIR221 | 2 |

- **Supplementary Table S5. GO Cellular Component (CC) enrichment results for core DEGs.**

Enriched CC terms for the 73 core genes, including subcellular localization categories and significance values.

| **ID** | **Description** | **GeneRatio** | **BgRatio** | **pvalue** | **p.adjust** | **qvalue** | **geneID** | **Count** |
| --- | --- | --- | --- | --- | --- | --- | --- | --- |
| GO:0030176 | integral component of endoplasmic reticulum membrane | 2/28 | 162/19550 | 0.022401898 | 0.319610215 | 0.28632494 | HSD11B1/SCD | 2 |
| GO:0031227 | intrinsic component of endoplasmic reticulum membrane | 2/28 | 170/19550 | 0.024503919 | 0.319610215 | 0.28632494 | HSD11B1/SCD | 2 |
| GO:0019897 | extrinsic component of plasma membrane | 2/28 | 174/19550 | 0.025584328 | 0.319610215 | 0.28632494 | CDHR3/GNB3 | 2 |
| GO:0032982 | myosin filament | 1/28 | 22/19550 | 0.031056035 | 0.319610215 | 0.28632494 | MYBPH | 1 |
| GO:0016342 | catenin complex | 1/28 | 31/19550 | 0.043490877 | 0.319610215 | 0.28632494 | CDHR3 | 1 |
| GO:0005834 | heterotrimeric G-protein complex | 1/28 | 34/19550 | 0.047601521 | 0.319610215 | 0.28632494 | GNB3 | 1 |
| GO:1905360 | GTPase complex | 1/28 | 34/19550 | 0.047601521 | 0.319610215 | 0.28632494 | GNB3 | 1 |
|  |  |  |  |  |  |  |  |  |

- **Supplementary Table S6. GO Molecular Function (MF) enrichment results for core DEGs.**

Enriched MF terms for the 73 core genes, including molecular activity categories and significance values.

| **ID** | **Description** | **GeneRatio** | **BgRatio** | **pvalue** | **p.adjust** | **qvalue** | **geneID** | **Count** |
| --- | --- | --- | --- | --- | --- | --- | --- | --- |
| GO:0005179 | hormone activity | 3/28 | 122/18368 | 0.000829553 | 0.044318332 | 0.031243247 | ADM/APLN/STC2 | 3 |
| GO:0033764 | steroid dehydrogenase activity, acting on the CH-OH group of donors, NAD or NADP as acceptor | 2/28 | 30/18368 | 0.000949382 | 0.044318332 | 0.031243247 | AKR1C3/HSD11B1 | 2 |
| GO:0016229 | steroid dehydrogenase activity | 2/28 | 34/18368 | 0.001219771 | 0.044318332 | 0.031243247 | AKR1C3/HSD11B1 | 2 |
| GO:0030159 | signaling receptor complex adaptor activity | 2/28 | 50/18368 | 0.002623678 | 0.071495225 | 0.050402235 | GNB3/STAP1 | 2 |
| GO:0033293 | monocarboxylic acid binding | 2/28 | 73/18368 | 0.005508228 | 0.120079374 | 0.08465277 | AKR1C3/OXER1 | 2 |
| GO:0035591 | signaling adaptor activity | 2/28 | 81/18368 | 0.006740161 | 0.12126934 | 0.085491664 | GNB3/STAP1 | 2 |
| GO:0016717 | oxidoreductase activity, acting on paired donors, with oxidation of a pair of donors resulting in the reduction of molecular oxygen to two molecules of water | 1/28 | 10/18368 | 0.015143442 | 0.12126934 | 0.085491664 | SCD | 1 |
| GO:0048256 | flap endonuclease activity | 1/28 | 10/18368 | 0.015143442 | 0.12126934 | 0.085491664 | ZGRF1 | 1 |
| GO:0016616 | oxidoreductase activity, acting on the CH-OH group of donors, NAD or NADP as acceptor | 2/28 | 124/18368 | 0.015238041 | 0.12126934 | 0.085491664 | AKR1C3/HSD11B1 | 2 |
| GO:0030283 | testosterone dehydrogenase [NAD(P)] activity | 1/28 | 11/18368 | 0.016645565 | 0.12126934 | 0.085491664 | AKR1C3 | 1 |
| GO:0032052 | bile acid binding | 1/28 | 11/18368 | 0.016645565 | 0.12126934 | 0.085491664 | AKR1C3 | 1 |
| GO:0016614 | oxidoreductase activity, acting on CH-OH group of donors | 2/28 | 136/18368 | 0.018138625 | 0.12126934 | 0.085491664 | AKR1C3/HSD11B1 | 2 |
| GO:0004032 | alditol:NADP+ 1-oxidoreductase activity | 1/28 | 12/18368 | 0.01814548 | 0.12126934 | 0.085491664 | AKR1C3 | 1 |
| GO:0016840 | carbon-nitrogen lyase activity | 1/28 | 13/18368 | 0.019643187 | 0.12126934 | 0.085491664 | CHAC1 | 1 |
| GO:0052650 | NADP-retinol dehydrogenase activity | 1/28 | 13/18368 | 0.019643187 | 0.12126934 | 0.085491664 | AKR1C3 | 1 |
| GO:0072582 | 17-beta-hydroxysteroid dehydrogenase (NADP+) activity | 1/28 | 13/18368 | 0.019643187 | 0.12126934 | 0.085491664 | AKR1C3 | 1 |
| GO:0005068 | transmembrane receptor protein tyrosine kinase adaptor activity | 1/28 | 14/18368 | 0.021138692 | 0.12126934 | 0.085491664 | STAP1 | 1 |
| GO:0036041 | long-chain fatty acid binding | 1/28 | 14/18368 | 0.021138692 | 0.12126934 | 0.085491664 | OXER1 | 1 |
| GO:0044594 | 17-beta-hydroxysteroid dehydrogenase (NAD+) activity | 1/28 | 14/18368 | 0.021138692 | 0.12126934 | 0.085491664 | AKR1C3 | 1 |
| GO:0016888 | endodeoxyribonuclease activity, producing 5'-phosphomonoesters | 1/28 | 16/18368 | 0.024123105 | 0.12289537 | 0.086637972 | ZGRF1 | 1 |
| GO:0004303 | estradiol 17-beta-dehydrogenase activity | 1/28 | 18/18368 | 0.027098743 | 0.12289537 | 0.086637972 | AKR1C3 | 1 |
| GO:0047834 | D-threo-aldose 1-dehydrogenase activity | 1/28 | 18/18368 | 0.027098743 | 0.12289537 | 0.086637972 | AKR1C3 | 1 |
| GO:0016705 | oxidoreductase activity, acting on paired donors, with incorporation or reduction of molecular oxygen | 2/28 | 170/18368 | 0.027497536 | 0.12289537 | 0.086637972 | AKR1C3/SCD | 2 |
| GO:0005201 | extracellular matrix structural constituent | 2/28 | 172/18368 | 0.028097892 | 0.12289537 | 0.086637972 | CHI3L1/IMPG2 | 2 |
| GO:0004745 | NAD-retinol dehydrogenase activity | 1/28 | 19/18368 | 0.028583279 | 0.12289537 | 0.086637972 | AKR1C3 | 1 |
| GO:0031406 | carboxylic acid binding | 2/28 | 176/18368 | 0.029314492 | 0.12289537 | 0.086637972 | AKR1C3/OXER1 | 2 |
| GO:0005540 | hyaluronic acid binding | 1/28 | 22/18368 | 0.033023792 | 0.127546109 | 0.08991662 | IMPG2 | 1 |
| GO:0008106 | alcohol dehydrogenase (NADP+) activity | 1/28 | 22/18368 | 0.033023792 | 0.127546109 | 0.08991662 | AKR1C3 | 1 |
| GO:1901567 | fatty acid derivative binding | 1/28 | 23/18368 | 0.034499609 | 0.127546109 | 0.08991662 | OXER1 | 1 |
| GO:0070840 | dynein complex binding | 1/28 | 24/18368 | 0.035973254 | 0.127546109 | 0.08991662 | DNAAF1 | 1 |
| GO:0048018 | receptor ligand activity | 3/28 | 487/18368 | 0.037147107 | 0.127546109 | 0.08991662 | ADM/APLN/STC2 | 3 |
| GO:0005112 | Notch binding | 1/28 | 25/18368 | 0.037444729 | 0.127546109 | 0.08991662 | CHAC1 | 1 |
| GO:0030546 | signaling receptor activator activity | 3/28 | 495/18368 | 0.038699228 | 0.127824724 | 0.090113036 | ADM/APLN/STC2 | 3 |
| GO:0030507 | spectrin binding | 1/28 | 27/18368 | 0.040381186 | 0.129457331 | 0.09126398 | GNB3 | 1 |
| GO:0004033 | aldo-keto reductase (NADP) activity | 1/28 | 29/18368 | 0.043309003 | 0.131130037 | 0.092443194 | AKR1C3 | 1 |
| GO:0016628 | oxidoreductase activity, acting on the CH-CH group of donors, NAD or NADP as acceptor | 1/28 | 29/18368 | 0.043309003 | 0.131130037 | 0.092443194 | AKR1C3 | 1 |

- **Supplementary Table S7. KEGG pathway enrichment results for core DEGs.**

Significant KEGG pathways identified for the 73 core genes with pathway name, gene members, and FDR values.

| **ID** | **Description** | **GeneRatio** | **BgRatio** | **pvalue** | **p.adjust** | **qvalue** | **geneID** | **Count** |
| --- | --- | --- | --- | --- | --- | --- | --- | --- |
| hsa00140 | Steroid hormone biosynthesis | 2/13 | 63/8535 | 0.003969242 | 0.158769667 | 0.150413369 | AKR1C3/HSD11B1 | 2 |
| hsa04371 | Apelin signaling pathway | 2/13 | 140/8535 | 0.018510988 | 0.310096222 | 0.293775368 | APLN/GNB3 | 2 |
| hsa01040 | Biosynthesis of unsaturated fatty acids | 1/13 | 27/8535 | 0.040381046 | 0.310096222 | 0.293775368 | SCD | 1 |
| hsa00790 | Folate biosynthesis | 1/13 | 28/8535 | 0.041847318 | 0.310096222 | 0.293775368 | AKR1C3 | 1 |

- **Supplementary Table S8. GO Biological Process (BP) enrichment of PSPC1-specific DEGs.**

Functional enrichment results specific to PSPC1 knockdown.

| **ID** | **Description** | **GeneRatio** | **BgRatio** | **pvalue** | **p.adjust** | **qvalue** | **geneID** | **Count** |
| --- | --- | --- | --- | --- | --- | --- | --- | --- |
| GO:0002274 | myeloid leukocyte activation | 21/261 | 223/18723 | 5.96489E-12 | 2.22729E-08 | 1.62559E-08 | AZU1/BPI/CCL5/CD300LF/CNR2/FCGR2B/GRN/HMOX1/ITGB2/KIT/LILRB4/LRRK2/MT1G/PRAM1/PTAFR/SUCNR1/TGFBR2/TLR4/TREM2/TSPAN32/TYROBP | 21 |
| GO:0002573 | myeloid leukocyte differentiation | 19/261 | 208/18723 | 1.06335E-10 | 1.42307E-07 | 1.03863E-07 | ANXA2/CA2/CD109/CSF1R/GPC3/GPR68/JUN/JUNB/KIT/LILRB4/MMP9/MT1G/MYC/SRC/TGFBR2/TLR4/TREM2/TYROBP/VEGFA | 19 |
| GO:0071276 | cellular response to cadmium ion | 10/261 | 40/18723 | 1.36898E-10 | 1.42307E-07 | 1.03863E-07 | CYBB/HMOX1/JUN/MMP9/MT1E/MT1G/MT1X/MT2A/NCF1/STAR | 10 |
| GO:0046686 | response to cadmium ion | 12/261 | 68/18723 | 1.52445E-10 | 1.42307E-07 | 1.03863E-07 | CYBB/HMOX1/JUN/KIT/MMP9/MT1E/MT1G/MT1X/MT2A/NCF1/STAR/TERT | 12 |
| GO:0002697 | regulation of immune effector process | 23/261 | 339/18723 | 4.46101E-10 | 3.33148E-07 | 2.43148E-07 | A2M/C3/CD1D/CD36/CD86/CFH/FCER2/FCGR2B/GRN/HMOX1/ITGB2/KIT/LILRB4/NCF1/PRAM1/PTAFR/PTPN22/SLC15A4/SLC7A5/TLR4/TREM2/TYROBP/XBP1 | 23 |
| GO:0001819 | positive regulation of cytokine production | 26/261 | 467/18723 | 2.18243E-09 | 1.3582E-06 | 9.91284E-07 | AZU1/C3/CD36/CD86/CHI3L1/CSF1R/CYBB/EGR1/F3/FCN1/FURIN/HMOX1/KIT/LILRB2/LRRK2/MCOLN2/NLRP12/PTAFR/PTPN22/SLC7A5/SRC/TLR4/TREM2/TRIM16/TYROBP/XBP1 | 26 |
| GO:0030316 | osteoclast differentiation | 12/261 | 94/18723 | 7.25415E-09 | 3.47428E-06 | 2.53571E-06 | ANXA2/CA2/CD109/CSF1R/GPC3/GPR68/JUNB/LILRB4/SRC/TLR4/TREM2/TYROBP | 12 |
| GO:1903131 | mononuclear cell differentiation | 24/261 | 426/18723 | 7.44356E-09 | 3.47428E-06 | 2.53571E-06 | CD1D/CD86/CSF1R/CYP26B1/EGR1/FCGR2B/GAS6/GPR68/HDAC9/ITGA4/JUN/KIT/LILRB2/LILRB4/MT1G/MYC/PTPN22/SEMA4A/SOX4/TGFBR2/TREM2/VEGFA/XBP1/ZFP36L2 | 24 |
| GO:0002683 | negative regulation of immune system process | 23/261 | 434/18723 | 4.78648E-08 | 1.92217E-05 | 1.40289E-05 | A2M/BPI/CD300LF/CD86/CNR2/FCGR2B/FURIN/GAL/GPR68/GRN/HMOX1/LILRB2/LILRB4/MYC/NLRC3/PTPN22/SAMHD1/SAMSN1/SRC/TLR4/TREM2/TSPAN32/TYROBP | 23 |
| GO:0060191 | regulation of lipase activity | 11/261 | 91/18723 | 5.53468E-08 | 1.92217E-05 | 1.40289E-05 | CCL5/CD86/FGFR3/FURIN/HDAC9/KIT/P2RY6/PLCB2/PTAFR/RASGRP4/SORT1 | 11 |
| GO:0050900 | leukocyte migration | 21/261 | 369/18723 | 5.66251E-08 | 1.92217E-05 | 1.40289E-05 | AZU1/CCL5/CNR2/CSF1R/CXCL10/ECM1/GAS6/HMOX1/ITGA4/ITGB2/ITGB7/JAML/KIT/MCOLN2/MMP9/NLRP12/PTAFR/SRC/THBS4/TREM2/VEGFA | 21 |
| GO:0010038 | response to metal ion | 21/261 | 373/18723 | 6.80042E-08 | 2.11606E-05 | 1.54441E-05 | ALOX5AP/ASS1/CA2/CAMK2D/CYBB/HMOX1/IGFBP2/JUN/JUNB/KIT/LRRK2/MMP9/MT1E/MT1G/MT1X/MT2A/NCF1/RASGRP2/STAR/SUCNR1/TERT | 21 |
| GO:0071219 | cellular response to molecule of bacterial origin | 16/261 | 221/18723 | 8.9305E-08 | 2.56511E-05 | 1.87215E-05 | ASS1/BPI/CCL5/CD180/CD36/CD86/CXCL10/FCGR2B/LILRB2/PTAFR/PTPN22/SRC/STAR/TLR4/TREM2/XBP1 | 16 |
| GO:0071248 | cellular response to metal ion | 15/261 | 197/18723 | 1.17854E-07 | 3.14333E-05 | 2.29416E-05 | ALOX5AP/CAMK2D/CYBB/HMOX1/JUN/JUNB/LRRK2/MMP9/MT1E/MT1G/MT1X/MT2A/NCF1/RASGRP2/STAR | 15 |
| GO:0032103 | positive regulation of response to external stimulus | 22/261 | 427/18723 | 1.54723E-07 | 3.85157E-05 | 2.81107E-05 | ALOX5AP/AZU1/C3/CCL5/CD180/CD1D/CSF1R/CXCL10/F3/FCN1/GAS6/GRN/LRRK2/NLRP12/SLC15A4/SRC/SUCNR1/THBS4/TLR4/TREM2/TYROBP/VEGFA | 22 |
| GO:0010466 | negative regulation of peptidase activity | 17/261 | 262/18723 | 1.71415E-07 | 4.00039E-05 | 2.91969E-05 | A2M/ANXA2/C3/CD109/CST3/CSTA/ECM1/FURIN/GAS6/GPC3/MMP9/SERPINI1/SLPI/SPOCK2/SRC/TIMP1/VEGFA | 17 |
| GO:0002237 | response to molecule of bacterial origin | 20/261 | 363/18723 | 1.99824E-07 | 4.38907E-05 | 3.20336E-05 | ASS1/BPI/CCL5/CD180/CD36/CD86/CNR2/CXCL10/FCGR2B/JUN/LILRB2/PCK2/PTAFR/PTPN22/SLPI/SRC/STAR/TLR4/TREM2/XBP1 | 20 |
| GO:0050866 | negative regulation of cell activation | 15/261 | 210/18723 | 2.70858E-07 | 5.61879E-05 | 4.10088E-05 | BPI/CD300LF/CD86/CNR2/FCGR2B/GAL/GRN/HMOX1/LILRB2/LILRB4/PTPN22/SAMSN1/TREM2/TSPAN32/TYROBP | 15 |
| GO:0002366 | leukocyte activation involved in immune response | 17/261 | 275/18723 | 3.40906E-07 | 5.64139E-05 | 4.11737E-05 | CD180/CD86/FCGR2B/GRN/HMOX1/ITGB2/KIT/PRAM1/PTAFR/SEMA4A/SLC15A4/SUCNR1/TLR4/TREM2/TYROBP/UNG/XBP1 | 17 |
| GO:0045765 | regulation of angiogenesis | 19/261 | 342/18723 | 3.58593E-07 | 5.64139E-05 | 4.11737E-05 | ADM2/C3/CHI3L1/CXCL10/CYBB/ECM1/F3/GRN/HMOX1/HSPG2/ITGA5/SEMA4A/SEMA6A/TERT/TGFBR2/THBS4/TSPAN12/VEGFA/XBP1 | 19 |
| GO:1902563 | regulation of neutrophil activation | 5/261 | 12/18723 | 3.7045E-07 | 5.64139E-05 | 4.11737E-05 | FCGR2B/GRN/ITGB2/PRAM1/PTAFR | 5 |
| GO:0032496 | response to lipopolysaccharide | 19/261 | 343/18723 | 3.74937E-07 | 5.64139E-05 | 4.11737E-05 | ASS1/BPI/CCL5/CD180/CD36/CD86/CNR2/CXCL10/JUN/LILRB2/PCK2/PTAFR/PTPN22/SLPI/SRC/STAR/TLR4/TREM2/XBP1 | 19 |
| GO:0002695 | negative regulation of leukocyte activation | 14/261 | 187/18723 | 3.85321E-07 | 5.64139E-05 | 4.11737E-05 | BPI/CD300LF/CD86/CNR2/FCGR2B/GAL/GRN/HMOX1/LILRB2/LILRB4/PTPN22/SAMSN1/TSPAN32/TYROBP | 14 |
| GO:0071216 | cellular response to biotic stimulus | 16/261 | 246/18723 | 3.85476E-07 | 5.64139E-05 | 4.11737E-05 | ASS1/BPI/CCL5/CD180/CD36/CD86/CXCL10/FCGR2B/LILRB2/PTAFR/PTPN22/SRC/STAR/TLR4/TREM2/XBP1 | 16 |
| GO:0051346 | negative regulation of hydrolase activity | 20/261 | 379/18723 | 3.9716E-07 | 5.64139E-05 | 4.11737E-05 | A2M/ANXA2/C3/CD109/CST3/CSTA/ECM1/FURIN/GAS6/GPC3/HDAC9/LRRK2/MMP9/SERPINI1/SLPI/SORT1/SPOCK2/SRC/TIMP1/VEGFA | 20 |
| GO:0002263 | cell activation involved in immune response | 17/261 | 279/18723 | 4.17734E-07 | 5.64139E-05 | 4.11737E-05 | CD180/CD86/FCGR2B/GRN/HMOX1/ITGB2/KIT/PRAM1/PTAFR/SEMA4A/SLC15A4/SUCNR1/TLR4/TREM2/TYROBP/UNG/XBP1 | 17 |
| GO:1902105 | regulation of leukocyte differentiation | 17/261 | 279/18723 | 4.17734E-07 | 5.64139E-05 | 4.11737E-05 | CA2/CD86/CYP26B1/FCGR2B/GAS6/GPR68/JUN/LILRB2/LILRB4/MYC/SOX4/TGFBR2/TLR4/TREM2/TYROBP/XBP1/ZFP36L2 | 17 |
| GO:0030099 | myeloid cell differentiation | 20/261 | 381/18723 | 4.31598E-07 | 5.64139E-05 | 4.11737E-05 | ANXA2/CA2/CD109/CSF1R/GPC3/GPR68/JUN/JUNB/KIT/LILRB4/MMP9/MT1G/MYC/RASGRP4/SRC/TGFBR2/TLR4/TREM2/TYROBP/VEGFA | 20 |
| GO:0045088 | regulation of innate immune response | 15/261 | 218/18723 | 4.38137E-07 | 5.64139E-05 | 4.11737E-05 | A2M/CCL5/CD1D/FCGR2B/FCN1/GRN/NCF1/NLRC3/PTPN22/SAMHD1/SLC15A4/SRC/TLR4/TREM2/TYROBP | 15 |
| GO:1901342 | regulation of vasculature development | 19/261 | 348/18723 | 4.67346E-07 | 5.8169E-05 | 4.24547E-05 | ADM2/C3/CHI3L1/CXCL10/CYBB/ECM1/F3/GRN/HMOX1/HSPG2/ITGA5/SEMA4A/SEMA6A/TERT/TGFBR2/THBS4/TSPAN12/VEGFA/XBP1 | 19 |
| GO:0045861 | negative regulation of proteolysis | 19/261 | 351/18723 | 5.3233E-07 | 6.08589E-05 | 4.44179E-05 | A2M/ANXA2/C3/CD109/CHAC1/CST3/CSTA/ECM1/FURIN/GAS6/GPC3/LRRK2/MMP9/SERPINI1/SLPI/SPOCK2/SRC/TIMP1/VEGFA | 19 |
| GO:0042119 | neutrophil activation | 7/261 | 36/18723 | 5.57866E-07 | 6.08589E-05 | 4.44179E-05 | CCL5/FCGR2B/GRN/ITGB2/PRAM1/PTAFR/TYROBP | 7 |
| GO:0032635 | interleukin-6 production | 13/261 | 165/18723 | 5.64093E-07 | 6.08589E-05 | 4.44179E-05 | BPI/CD36/GAS6/LILRB2/LILRB4/NLRC3/NLRP12/PTAFR/PTPN22/TLR4/TREM2/TYROBP/XBP1 | 13 |
| GO:0032675 | regulation of interleukin-6 production | 13/261 | 165/18723 | 5.64093E-07 | 6.08589E-05 | 4.44179E-05 | BPI/CD36/GAS6/LILRB2/LILRB4/NLRC3/NLRP12/PTAFR/PTPN22/TLR4/TREM2/TYROBP/XBP1 | 13 |
| GO:0052547 | regulation of peptidase activity | 22/261 | 461/18723 | 5.70451E-07 | 6.08589E-05 | 4.44179E-05 | A2M/ANXA2/C3/CD109/CST3/CSTA/ECM1/F3/FURIN/GAS6/GPC3/GRN/GSN/MMP9/MYC/NLRP12/SERPINI1/SLPI/SPOCK2/SRC/TIMP1/VEGFA | 22 |
| GO:0071241 | cellular response to inorganic substance | 15/261 | 226/18723 | 6.93587E-07 | 7.19404E-05 | 5.25057E-05 | ALOX5AP/CAMK2D/CYBB/HMOX1/JUN/JUNB/LRRK2/MMP9/MT1E/MT1G/MT1X/MT2A/NCF1/RASGRP2/STAR | 15 |
| GO:0002831 | regulation of response to biotic stimulus | 18/261 | 327/18723 | 8.38981E-07 | 8.46691E-05 | 6.17958E-05 | A2M/CCL5/CD180/CD1D/CD36/FCGR2B/FCN1/GRN/NCF1/NLRC3/PTPN22/SAMHD1/SLC15A4/SRC/TLR4/TREM2/TSPAN32/TYROBP | 18 |
| GO:0010518 | positive regulation of phospholipase activity | 8/261 | 56/18723 | 1.02047E-06 | 9.77038E-05 | 7.13092E-05 | CCL5/CD86/FGFR3/KIT/P2RY6/PLCB2/PTAFR/RASGRP4 | 8 |
| GO:1904645 | response to amyloid-beta | 8/261 | 56/18723 | 1.02047E-06 | 9.77038E-05 | 7.13092E-05 | CD36/DNM1/FCGR2B/ITGA4/MMP2/MMP9/TLR4/TREM2 | 8 |
| GO:0043410 | positive regulation of MAPK cascade | 22/261 | 480/18723 | 1.11828E-06 | 0.000103406 | 7.54712E-05 | CCL5/CD36/CHI3L1/CSF1R/FCGR2B/FGFR3/GAS6/KIT/KSR1/LRRK2/NCF1/NDST1/P2RY6/PTPN22/RAP1B/SEMA4C/SH3RF3/SRC/TLR4/TNFRSF19/TREM2/VEGFA | 22 |
| GO:0002699 | positive regulation of immune effector process | 15/261 | 235/18723 | 1.13542E-06 | 0.000103406 | 7.54712E-05 | C3/CD1D/CD36/CD86/FCER2/HMOX1/ITGB2/KIT/PTAFR/PTPN22/SLC7A5/TLR4/TREM2/TYROBP/XBP1 | 15 |
| GO:0071222 | cellular response to lipopolysaccharide | 14/261 | 209/18723 | 1.46981E-06 | 0.000130673 | 9.53721E-05 | ASS1/BPI/CCL5/CD180/CD36/CD86/CXCL10/LILRB2/PTAFR/PTPN22/SRC/STAR/TLR4/XBP1 | 14 |
| GO:0045766 | positive regulation of angiogenesis | 13/261 | 181/18723 | 1.61147E-06 | 0.000136755 | 9.9811E-05 | ADM2/C3/CHI3L1/CYBB/ECM1/F3/GRN/HMOX1/ITGA5/TERT/TGFBR2/VEGFA/XBP1 | 13 |
| GO:1904018 | positive regulation of vasculature development | 13/261 | 181/18723 | 1.61147E-06 | 0.000136755 | 9.9811E-05 | ADM2/C3/CHI3L1/CYBB/ECM1/F3/GRN/HMOX1/ITGA5/TERT/TGFBR2/VEGFA/XBP1 | 13 |
| GO:0048661 | positive regulation of smooth muscle cell proliferation | 10/261 | 104/18723 | 1.92801E-06 | 0.000159982 | 0.000116763 | CCL5/HMOX1/JUN/MMP2/MMP9/P2RY6/PTAFR/TERT/TGFBR2/TLR4 | 10 |
| GO:0036230 | granulocyte activation | 7/261 | 43/18723 | 1.98155E-06 | 0.00016085 | 0.000117397 | CCL5/FCGR2B/GRN/ITGB2/PRAM1/PTAFR/TYROBP | 7 |
| GO:1902107 | positive regulation of leukocyte differentiation | 12/261 | 157/18723 | 2.12768E-06 | 0.000163216 | 0.000119124 | CA2/CD86/GAS6/GPR68/JUN/LILRB2/LILRB4/SOX4/TGFBR2/TREM2/TYROBP/XBP1 | 12 |
| GO:1903708 | positive regulation of hemopoiesis | 12/261 | 157/18723 | 2.12768E-06 | 0.000163216 | 0.000119124 | CA2/CD86/GAS6/GPR68/JUN/LILRB2/LILRB4/SOX4/TGFBR2/TREM2/TYROBP/XBP1 | 12 |
| GO:0071706 | tumor necrosis factor superfamily cytokine production | 13/261 | 186/18723 | 2.18554E-06 | 0.000163216 | 0.000119124 | AZU1/BPI/CD36/CD86/CYBB/GAS6/LRRK2/NLRC3/PTAFR/PTPN22/TLR4/TREM2/TYROBP | 13 |
| GO:1903555 | regulation of tumor necrosis factor superfamily cytokine production | 13/261 | 186/18723 | 2.18554E-06 | 0.000163216 | 0.000119124 | AZU1/BPI/CD36/CD86/CYBB/GAS6/LRRK2/NLRC3/PTAFR/PTPN22/TLR4/TREM2/TYROBP | 13 |
| GO:0042116 | macrophage activation | 10/261 | 106/18723 | 2.29624E-06 | 0.000168121 | 0.000122703 | AZU1/BPI/FCGR2B/GRN/ITGB2/LRRK2/SUCNR1/TLR4/TREM2/TYROBP | 10 |
| GO:0007229 | integrin-mediated signaling pathway | 10/261 | 107/18723 | 2.50227E-06 | 0.000179682 | 0.000131141 | FYB1/ITGA4/ITGA5/ITGA6/ITGB2/ITGB7/PRAM1/SRC/TIMP1/TSPAN32 | 10 |
| GO:0010951 | negative regulation of endopeptidase activity | 15/261 | 252/18723 | 2.7063E-06 | 0.000190666 | 0.000139158 | A2M/ANXA2/C3/CD109/CST3/CSTA/FURIN/GAS6/MMP9/SERPINI1/SLPI/SPOCK2/SRC/TIMP1/VEGFA | 15 |
| GO:0050920 | regulation of chemotaxis | 14/261 | 223/18723 | 3.15302E-06 | 0.000218026 | 0.000159126 | AZU1/CCL5/CSF1R/CXCL10/F3/GAS6/SEMA4A/SEMA4C/SEMA6A/SEMA6B/SUCNR1/THBS4/TREM2/VEGFA | 14 |
| GO:0045785 | positive regulation of cell adhesion | 20/261 | 437/18723 | 3.57141E-06 | 0.000242466 | 0.000176964 | AZU1/CCL5/CD1D/CD36/CD86/IGFBP2/ITGA4/ITGA6/ITGB2/LILRB2/LILRB4/MYO10/PTAFR/PTPN22/SOX4/SPOCK2/SRC/TGFBR2/VEGFA/XBP1 | 20 |
| GO:0002703 | regulation of leukocyte mediated immunity | 14/261 | 226/18723 | 3.68409E-06 | 0.00024565 | 0.000179288 | C3/CD1D/FCER2/FCGR2B/HMOX1/ITGB2/KIT/LILRB4/PRAM1/PTAFR/SLC15A4/TLR4/TREM2/TYROBP | 14 |
| GO:0010517 | regulation of phospholipase activity | 8/261 | 67/18723 | 4.10546E-06 | 0.000268943 | 0.000196289 | CCL5/CD86/FGFR3/KIT/P2RY6/PLCB2/PTAFR/RASGRP4 | 8 |
| GO:0050730 | regulation of peptidyl-tyrosine phosphorylation | 15/261 | 264/18723 | 4.78047E-06 | 0.000307763 | 0.000224621 | CCL5/CD36/CSF1R/FGFR3/GAS6/ITGA5/ITGB2/KIT/LILRB4/NCF1/SAMSN1/SRC/THBS4/TREM2/VEGFA | 15 |
| GO:0002275 | myeloid cell activation involved in immune response | 9/261 | 91/18723 | 5.01273E-06 | 0.000317246 | 0.000231543 | GRN/HMOX1/ITGB2/KIT/PRAM1/PTAFR/SUCNR1/TREM2/TYROBP | 9 |
| GO:0060193 | positive regulation of lipase activity | 8/261 | 69/18723 | 5.13818E-06 | 0.000319766 | 0.000233382 | CCL5/CD86/FGFR3/KIT/P2RY6/PLCB2/PTAFR/RASGRP4 | 8 |
| GO:0007584 | response to nutrient | 12/261 | 174/18723 | 6.18949E-06 | 0.000378878 | 0.000276524 | ASS1/CXCL10/CYBB/CYP26B1/GAS6/GCLM/HMOX1/IGFBP2/STAR/STC2/TGFBR2/XBP1 | 12 |
| GO:1903039 | positive regulation of leukocyte cell-cell adhesion | 14/261 | 239/18723 | 7.02318E-06 | 0.000422977 | 0.00030871 | CCL5/CD1D/CD86/IGFBP2/ITGA4/ITGB2/LILRB2/LILRB4/PTAFR/PTPN22/SOX4/SRC/TGFBR2/XBP1 | 14 |
| GO:0072203 | cell proliferation involved in metanephros development | 4/261 | 10/18723 | 7.25507E-06 | 0.000430007 | 0.000313841 | EGR1/GPC3/MYC/PTCH1 | 4 |
| GO:0043277 | apoptotic cell clearance | 7/261 | 52/18723 | 7.39304E-06 | 0.000431338 | 0.000314812 | C3/CD300LF/CD36/FCN1/GAS6/TREM2/TYROBP | 7 |
| GO:0042060 | wound healing | 19/261 | 422/18723 | 7.94637E-06 | 0.000456488 | 0.000333168 | ANXA2/CD109/CD36/F3/FAP/GAS6/HMOX1/HPS4/ITGA5/JAML/JMJD1C/NOG/SRC/TGFBR2/TIMP1/TLR4/TSPAN32/VEGFA/XBP1 | 19 |
| GO:0007566 | embryo implantation | 7/261 | 53/18723 | 8.41761E-06 | 0.000476233 | 0.000347579 | MMP2/MMP9/PRLR/STC2/TGFBR2/TIMP1/VEGFA | 7 |
| GO:0006801 | superoxide metabolic process | 8/261 | 74/18723 | 8.72213E-06 | 0.000486096 | 0.000354778 | CD36/CYBB/ITGB2/NCF1/NCF1B/NCF1C/PRDX1/TYROBP | 8 |
| GO:0071456 | cellular response to hypoxia | 11/261 | 151/18723 | 8.99844E-06 | 0.000493849 | 0.000360436 | CYBB/EGR1/HMOX1/LMNA/MYC/SLC29A1/SRC/STC2/TERT/TREM2/VEGFA | 11 |
| GO:0032640 | tumor necrosis factor production | 12/261 | 181/18723 | 9.25802E-06 | 0.000493849 | 0.000360436 | AZU1/BPI/CD36/CYBB/GAS6/LRRK2/NLRC3/PTAFR/PTPN22/TLR4/TREM2/TYROBP | 12 |
| GO:0032680 | regulation of tumor necrosis factor production | 12/261 | 181/18723 | 9.25802E-06 | 0.000493849 | 0.000360436 | AZU1/BPI/CD36/CYBB/GAS6/LRRK2/NLRC3/PTAFR/PTPN22/TLR4/TREM2/TYROBP | 12 |
| GO:0030224 | monocyte differentiation | 6/261 | 36/18723 | 9.5008E-06 | 0.000499662 | 0.000364679 | CSF1R/GPR68/JUN/MT1G/MYC/VEGFA | 6 |
| GO:0002444 | myeloid leukocyte mediated immunity | 9/261 | 99/18723 | 1.00495E-05 | 0.000521178 | 0.000380382 | AZU1/C3/FCGR2B/HMOX1/ITGB2/KIT/PRAM1/PTAFR/TYROBP | 9 |
| GO:0070661 | leukocyte proliferation | 16/261 | 318/18723 | 1.07681E-05 | 0.000550798 | 0.000402 | CCL5/CD180/CD1D/CD86/CSF1R/FCGR2B/GAL/IGFBP2/KIT/LILRB2/LILRB4/PTPN22/TGFBR2/TLR4/TREM2/TYROBP | 16 |
| GO:0052548 | regulation of endopeptidase activity | 19/261 | 432/18723 | 1.10584E-05 | 0.000558 | 0.000407257 | A2M/ANXA2/C3/CD109/CST3/CSTA/F3/FURIN/GAS6/GSN/MMP9/MYC/NLRP12/SERPINI1/SLPI/SPOCK2/SRC/TIMP1/VEGFA | 19 |
| GO:0045730 | respiratory burst | 6/261 | 37/18723 | 1.12081E-05 | 0.000558012 | 0.000407266 | CYBB/GRN/NCF1/NCF1B/NCF1C/TREM2 | 6 |
| GO:0022409 | positive regulation of cell-cell adhesion | 15/261 | 284/18723 | 1.14903E-05 | 0.000564537 | 0.000412028 | CCL5/CD1D/CD86/IGFBP2/ITGA4/ITGB2/LILRB2/LILRB4/MYO10/PTAFR/PTPN22/SOX4/SRC/TGFBR2/XBP1 | 15 |
| GO:0002886 | regulation of myeloid leukocyte mediated immunity | 7/261 | 56/18723 | 1.22221E-05 | 0.000580529 | 0.0004237 | C3/FCGR2B/HMOX1/ITGB2/PRAM1/PTAFR/TYROBP | 7 |
| GO:0001667 | ameboidal-type cell migration | 20/261 | 475/18723 | 1.22362E-05 | 0.000580529 | 0.0004237 | EPHB4/FAP/GPC3/GRN/HDAC9/HMOX1/ITGA4/ITGB7/KIT/MMP9/PIK3R3/S100P/SEMA4A/SEMA4C/SEMA6A/SEMA6B/SRC/TGFBR2/TIMP1/VEGFA | 20 |
| GO:0032612 | interleukin-1 production | 10/261 | 128/18723 | 1.25081E-05 | 0.000580529 | 0.0004237 | AZU1/CD36/EGR1/GAS6/LILRB4/NLRP12/TLR4/TREM2/TRIM16/TYROBP | 10 |
| GO:0032652 | regulation of interleukin-1 production | 10/261 | 128/18723 | 1.25081E-05 | 0.000580529 | 0.0004237 | AZU1/CD36/EGR1/GAS6/LILRB4/NLRP12/TLR4/TREM2/TRIM16/TYROBP | 10 |
| GO:1901653 | cellular response to peptide | 17/261 | 359/18723 | 1.25932E-05 | 0.000580529 | 0.0004237 | ASS1/CA2/CD36/FCGR2B/HDAC9/ITGA4/PCK2/PIK3R3/PTPN22/RAP1B/SRC/STAR/TBC1D4/TLR4/TREM2/TRIB3/XBP1 | 17 |
| GO:0036294 | cellular response to decreased oxygen levels | 11/261 | 161/18723 | 1.6507E-05 | 0.000751674 | 0.00054861 | CYBB/EGR1/HMOX1/LMNA/MYC/SLC29A1/SRC/STC2/TERT/TREM2/VEGFA | 11 |
| GO:1903706 | regulation of hemopoiesis | 17/261 | 367/18723 | 1.67291E-05 | 0.000752606 | 0.00054929 | CA2/CD86/CYP26B1/FCGR2B/GAS6/GPR68/JUN/LILRB2/LILRB4/MYC/SOX4/TGFBR2/TLR4/TREM2/TYROBP/XBP1/ZFP36L2 | 17 |
| GO:0070371 | ERK1 and ERK2 cascade | 16/261 | 330/18723 | 1.70269E-05 | 0.000756887 | 0.000552414 | CCL5/CD36/CHI3L1/CSF1R/FGFR3/GAS6/MYC/NLRP12/P2RY6/PTPN22/RAP1B/SEMA6A/SRC/TLR4/TREM2/ZFP36L2 | 16 |
| GO:0042110 | T cell activation | 20/261 | 487/18723 | 1.75397E-05 | 0.00076692 | 0.000559737 | CCL5/CD1D/CD86/CYP26B1/EGR1/FCGR2B/GSN/IGFBP2/JAML/KIT/LILRB2/LILRB4/NLRC3/PTPN22/SEMA4A/SOX4/SRC/TGFBR2/XBP1/ZFP36L2 | 20 |
| GO:0050731 | positive regulation of peptidyl-tyrosine phosphorylation | 12/261 | 193/18723 | 1.76741E-05 | 0.00076692 | 0.000559737 | CCL5/CD36/CSF1R/FGFR3/GAS6/ITGA5/KIT/NCF1/SRC/THBS4/TREM2/VEGFA | 12 |
| GO:0010863 | positive regulation of phospholipase C activity | 6/261 | 40/18723 | 1.78688E-05 | 0.00076692 | 0.000559737 | CD86/KIT/P2RY6/PLCB2/PTAFR/RASGRP4 | 6 |
| GO:0002696 | positive regulation of leukocyte activation | 18/261 | 409/18723 | 1.87496E-05 | 0.000795579 | 0.000580654 | CCL5/CD1D/CD86/GAS6/IGFBP2/ITGB2/LILRB2/LILRB4/LRRK2/PTAFR/PTPN22/SOX4/SRC/TGFBR2/TLR4/TREM2/TYROBP/XBP1 | 18 |
| GO:0007159 | leukocyte cell-cell adhesion | 17/261 | 371/18723 | 1.92201E-05 | 0.000805203 | 0.000587678 | ASS1/CCL5/CD1D/CD86/FCGR2B/IGFBP2/ITGA4/ITGB2/ITGB7/LILRB2/LILRB4/PTAFR/PTPN22/SOX4/SRC/TGFBR2/XBP1 | 17 |
| GO:0031663 | lipopolysaccharide-mediated signaling pathway | 7/261 | 60/18723 | 1.94109E-05 | 0.000805203 | 0.000587678 | BPI/CCL5/CD180/CD36/PTAFR/PTPN22/TLR4 | 7 |
| GO:0002700 | regulation of production of molecular mediator of immune response | 11/261 | 164/18723 | 1.96233E-05 | 0.000805203 | 0.000587678 | CD36/CD86/FCGR2B/HMOX1/KIT/LILRB4/PTPN22/SLC15A4/SLC7A5/TLR4/XBP1 | 11 |
| GO:1903037 | regulation of leukocyte cell-cell adhesion | 16/261 | 336/18723 | 2.12316E-05 | 0.000855207 | 0.000624173 | ASS1/CCL5/CD1D/CD86/FCGR2B/IGFBP2/ITGA4/ITGB2/LILRB2/LILRB4/PTAFR/PTPN22/SOX4/SRC/TGFBR2/XBP1 | 16 |
| GO:0030098 | lymphocyte differentiation | 17/261 | 374/18723 | 2.13E-05 | 0.000855207 | 0.000624173 | CD1D/CD86/CYP26B1/EGR1/FCGR2B/GAS6/HDAC9/ITGA4/KIT/LILRB2/LILRB4/PTPN22/SEMA4A/SOX4/TGFBR2/XBP1/ZFP36L2 | 17 |
| GO:0043434 | response to peptide hormone | 18/261 | 414/18723 | 2.2032E-05 | 0.000866153 | 0.000632162 | ASS1/CA2/CYBB/EGR1/GAL/HDAC9/PCK2/PIK3R3/RAP1B/SORT1/SRC/STAR/STC2/TBC1D4/TIMP1/TRIB3/TRIM16/XBP1 | 18 |
| GO:0018108 | peptidyl-tyrosine phosphorylation | 17/261 | 375/18723 | 2.20366E-05 | 0.000866153 | 0.000632162 | CCL5/CD36/CSF1R/EPHB4/FGFR3/GAS6/ITGA5/ITGB2/KIT/LILRB4/NCF1/PRLR/SAMSN1/SRC/THBS4/TREM2/VEGFA | 17 |
| GO:0030198 | extracellular matrix organization | 15/261 | 301/18723 | 2.27577E-05 | 0.000885181 | 0.00064605 | ADAMTS10/ADAMTS5/ANXA2/COL9A2/COLQ/CST3/FAP/FURIN/GAS6/MFAP4/MMP2/MMP9/QSOX1/RXFP1/SPOCK2 | 15 |
| GO:0032611 | interleukin-1 beta production | 9/261 | 110/18723 | 2.35999E-05 | 0.000890847 | 0.000650186 | AZU1/CD36/EGR1/LILRB4/NLRP12/TLR4/TREM2/TRIM16/TYROBP | 9 |
| GO:0032651 | regulation of interleukin-1 beta production | 9/261 | 110/18723 | 2.35999E-05 | 0.000890847 | 0.000650186 | AZU1/CD36/EGR1/LILRB4/NLRP12/TLR4/TREM2/TRIM16/TYROBP | 9 |
| GO:0043062 | extracellular structure organization | 15/261 | 302/18723 | 2.36529E-05 | 0.000890847 | 0.000650186 | ADAMTS10/ADAMTS5/ANXA2/COL9A2/COLQ/CST3/FAP/FURIN/GAS6/MFAP4/MMP2/MMP9/QSOX1/RXFP1/SPOCK2 | 15 |
| GO:1900274 | regulation of phospholipase C activity | 6/261 | 42/18723 | 2.38577E-05 | 0.000890847 | 0.000650186 | CD86/KIT/P2RY6/PLCB2/PTAFR/RASGRP4 | 6 |
| GO:0032731 | positive regulation of interleukin-1 beta production | 7/261 | 62/18723 | 2.41391E-05 | 0.000892428 | 0.00065134 | AZU1/CD36/EGR1/NLRP12/TLR4/TRIM16/TYROBP | 7 |
| GO:0018212 | peptidyl-tyrosine modification | 17/261 | 378/18723 | 2.43842E-05 | 0.000892654 | 0.000651504 | CCL5/CD36/CSF1R/EPHB4/FGFR3/GAS6/ITGA5/ITGB2/KIT/LILRB4/NCF1/PRLR/SAMSN1/SRC/THBS4/TREM2/VEGFA | 17 |
| GO:0045229 | external encapsulating structure organization | 15/261 | 304/18723 | 2.55371E-05 | 0.000925782 | 0.000675683 | ADAMTS10/ADAMTS5/ANXA2/COL9A2/COLQ/CST3/FAP/FURIN/GAS6/MFAP4/MMP2/MMP9/QSOX1/RXFP1/SPOCK2 | 15 |
| GO:0032102 | negative regulation of response to external stimulus | 18/261 | 420/18723 | 2.66417E-05 | 0.000947431 | 0.000691483 | A2M/ANXA2/CD109/CNR2/FAP/FCGR2B/FURIN/GRN/NLRC3/NLRP12/NUCB2/SAMHD1/SEMA4A/SEMA4C/SEMA6A/SEMA6B/SRC/TREM2 | 18 |
| GO:0050867 | positive regulation of cell activation | 18/261 | 420/18723 | 2.66417E-05 | 0.000947431 | 0.000691483 | CCL5/CD1D/CD86/GAS6/IGFBP2/ITGB2/LILRB2/LILRB4/LRRK2/PTAFR/PTPN22/SOX4/SRC/TGFBR2/TLR4/TREM2/TYROBP/XBP1 | 18 |
| GO:0050921 | positive regulation of chemotaxis | 10/261 | 141/18723 | 2.90654E-05 | 0.001023871 | 0.000747273 | AZU1/CCL5/CSF1R/CXCL10/F3/GAS6/SUCNR1/THBS4/TREM2/VEGFA | 10 |
| GO:0042554 | superoxide anion generation | 6/261 | 44/18723 | 3.13647E-05 | 0.001094539 | 0.00079885 | CYBB/ITGB2/NCF1/NCF1B/NCF1C/TYROBP | 6 |
| GO:0050679 | positive regulation of epithelial cell proliferation | 12/261 | 207/18723 | 3.53291E-05 | 0.001221469 | 0.00089149 | ECM1/EGFL7/F3/GRN/HMOX1/ITGA4/JAML/MYC/NOG/THBS4/VEGFA/XBP1 | 12 |
| GO:0002920 | regulation of humoral immune response | 6/261 | 45/18723 | 3.57708E-05 | 0.001225395 | 0.000894356 | A2M/C3/CFH/FCER2/FCGR2B/TREM2 | 6 |
| GO:0032715 | negative regulation of interleukin-6 production | 7/261 | 66/18723 | 3.64575E-05 | 0.001226418 | 0.000895102 | BPI/GAS6/LILRB4/NLRC3/NLRP12/PTPN22/TLR4 | 7 |
| GO:0050766 | positive regulation of phagocytosis | 7/261 | 66/18723 | 3.64575E-05 | 0.001226418 | 0.000895102 | AZU1/C3/CD300LF/CD36/FCGR2B/GAS6/TREM2 | 7 |
| GO:0031349 | positive regulation of defense response | 14/261 | 278/18723 | 3.80689E-05 | 0.001269192 | 0.000926321 | ALOX5AP/C3/CCL5/CD1D/FCN1/GRN/LRRK2/NLRP12/SLC15A4/SRC/SUCNR1/TLR4/TREM2/TYROBP | 14 |
| GO:0071453 | cellular response to oxygen levels | 11/261 | 177/18723 | 3.97518E-05 | 0.001313568 | 0.000958709 | CYBB/EGR1/HMOX1/LMNA/MYC/SLC29A1/SRC/STC2/TERT/TREM2/VEGFA | 11 |
| GO:0070663 | regulation of leukocyte proliferation | 13/261 | 245/18723 | 4.21779E-05 | 0.001381513 | 0.001008298 | CCL5/CD1D/CD86/CSF1R/FCGR2B/GAL/IGFBP2/LILRB2/LILRB4/PTPN22/TGFBR2/TLR4/TYROBP | 13 |
| GO:0001818 | negative regulation of cytokine production | 16/261 | 357/18723 | 4.41304E-05 | 0.001403141 | 0.001024084 | ANXA4/BPI/FCGR2B/FURIN/GAS6/HDAC9/HMOX1/HOMER3/LILRB4/NLRC3/NLRP12/PTPN22/SRGN/TLR4/TREM2/TYROBP | 16 |
| GO:0010273 | detoxification of copper ion | 4/261 | 15/18723 | 4.46328E-05 | 0.001403141 | 0.001024084 | MT1E/MT1G/MT1X/MT2A | 4 |
| GO:0090594 | inflammatory response to wounding | 4/261 | 15/18723 | 4.46328E-05 | 0.001403141 | 0.001024084 | GRN/HMOX1/TIMP1/TLR4 | 4 |
| GO:1990169 | stress response to copper ion | 4/261 | 15/18723 | 4.46328E-05 | 0.001403141 | 0.001024084 | MT1E/MT1G/MT1X/MT2A | 4 |
| GO:0050673 | epithelial cell proliferation | 18/261 | 437/18723 | 4.47171E-05 | 0.001403141 | 0.001024084 | CD109/ECM1/EGFL7/F3/FAP/GPC3/GRN/HMOX1/ITGA4/JAML/KIT/MYC/NLRC3/NOG/PTCH1/THBS4/VEGFA/XBP1 | 18 |
| GO:0001774 | microglial cell activation | 6/261 | 47/18723 | 4.60705E-05 | 0.001433561 | 0.001046286 | AZU1/GRN/ITGB2/LRRK2/TREM2/TYROBP | 6 |
| GO:0045926 | negative regulation of growth | 13/261 | 249/18723 | 4.98249E-05 | 0.001537573 | 0.001122199 | FGFR3/GPC3/MT1E/MT1G/MT1X/MT2A/NOG/PTCH1/SEMA4A/SEMA4C/SEMA6A/SEMA6B/TGFBR2 | 13 |
| GO:0002532 | production of molecular mediator involved in inflammatory response | 8/261 | 95/18723 | 5.46962E-05 | 0.001674061 | 0.001221815 | ALOX5AP/CD36/GRN/LILRB4/NCF1/NLRC3/TLR4/TREM2 | 8 |
| GO:0070374 | positive regulation of ERK1 and ERK2 cascade | 12/261 | 217/18723 | 5.58946E-05 | 0.001696833 | 0.001238435 | CCL5/CD36/CHI3L1/CSF1R/FGFR3/GAS6/P2RY6/PTPN22/RAP1B/SRC/TLR4/TREM2 | 12 |
| GO:0009743 | response to carbohydrate | 13/261 | 253/18723 | 5.86518E-05 | 0.001766175 | 0.001289045 | EGR1/GAS6/GCLM/GPR68/ME1/PCK2/RAP1B/SLC29A1/SOX4/STAR/TGFBR2/TREM2/XBP1 | 13 |
| GO:0022407 | regulation of cell-cell adhesion | 18/261 | 448/18723 | 6.15729E-05 | 0.001839307 | 0.00134242 | ASS1/CCL5/CD1D/CD86/FCGR2B/IGFBP2/ITGA4/ITGB2/LILRB2/LILRB4/MYO10/PTAFR/PTPN22/SOX4/SRC/TGFBR2/VEGFA/XBP1 | 18 |
| GO:0044706 | multi-multicellular organism process | 12/261 | 220/18723 | 6.38001E-05 | 0.00189071 | 0.001379936 | IGFBP2/JUNB/MMP2/MMP9/PRLR/PTAFR/RXFP1/STC2/STS/TGFBR2/TIMP1/VEGFA | 12 |
| GO:0032642 | regulation of chemokine production | 8/261 | 98/18723 | 6.83166E-05 | 0.002007253 | 0.001464995 | AZU1/CSF1R/EGR1/HMOX1/LILRB4/MCOLN2/TLR4/TREM2 | 8 |
| GO:0030217 | T cell differentiation | 13/261 | 257/18723 | 6.88078E-05 | 0.002007253 | 0.001464995 | CD1D/CD86/CYP26B1/EGR1/KIT/LILRB2/LILRB4/PTPN22/SEMA4A/SOX4/TGFBR2/XBP1/ZFP36L2 | 13 |
| GO:0032732 | positive regulation of interleukin-1 production | 7/261 | 73/18723 | 7.02136E-05 | 0.002032384 | 0.001483337 | AZU1/CD36/EGR1/NLRP12/TLR4/TRIM16/TYROBP | 7 |
| GO:0032602 | chemokine production | 8/261 | 99/18723 | 7.3439E-05 | 0.002109393 | 0.001539542 | AZU1/CSF1R/EGR1/HMOX1/LILRB4/MCOLN2/TLR4/TREM2 | 8 |
| GO:0048841 | regulation of axon extension involved in axon guidance | 5/261 | 32/18723 | 7.49772E-05 | 0.002137136 | 0.00155979 | SEMA4A/SEMA4C/SEMA6A/SEMA6B/VEGFA | 5 |
| GO:0036035 | osteoclast development | 4/261 | 17/18723 | 7.61295E-05 | 0.002153541 | 0.001571764 | ANXA2/GPR68/SRC/TYROBP | 4 |
| GO:0045807 | positive regulation of endocytosis | 8/261 | 100/18723 | 7.88762E-05 | 0.002204935 | 0.001609274 | ANXA2/C3/CD36/DNM1/GPC3/LRRK2/TREM2/VEGFA | 8 |
| GO:0050670 | regulation of lymphocyte proliferation | 12/261 | 225/18723 | 7.91273E-05 | 0.002204935 | 0.001609274 | CCL5/CD1D/CD86/FCGR2B/GAL/IGFBP2/LILRB2/LILRB4/PTPN22/TGFBR2/TLR4/TYROBP | 12 |
| GO:0032872 | regulation of stress-activated MAPK cascade | 11/261 | 192/18723 | 8.30599E-05 | 0.002293505 | 0.001673917 | FCGR2B/MYC/NCF1/PRDX1/PTPN22/SEMA4C/SH3RF3/TLR4/TNFRSF19/TREM2/VEGFA | 11 |
| GO:0043627 | response to estrogen | 7/261 | 75/18723 | 8.35342E-05 | 0.002293505 | 0.001673917 | CA2/GAL/HMOX1/IGFBP2/STAR/TGFBR2/ZNF366 | 7 |
| GO:0032944 | regulation of mononuclear cell proliferation | 12/261 | 227/18723 | 8.60926E-05 | 0.002346494 | 0.001712591 | CCL5/CD1D/CD86/FCGR2B/GAL/IGFBP2/LILRB2/LILRB4/PTPN22/TGFBR2/TLR4/TYROBP | 12 |
| GO:0050878 | regulation of body fluid levels | 16/261 | 379/18723 | 8.92816E-05 | 0.002415778 | 0.001763158 | ANXA2/CD36/CYP26B1/F3/FAP/GAS6/HPS4/JMJD1C/NFE2/PRLR/SLC29A1/SRC/TLR4/TSPAN32/VEGFA/XBP1 | 16 |
| GO:0050777 | negative regulation of immune response | 11/261 | 194/18723 | 9.11234E-05 | 0.002447876 | 0.001786584 | A2M/FCGR2B/FURIN/GRN/HMOX1/LILRB4/NLRC3/SAMHD1/SAMSN1/SRC/TREM2 | 11 |
| GO:0050678 | regulation of epithelial cell proliferation | 16/261 | 381/18723 | 9.49141E-05 | 0.002510379 | 0.001832202 | CD109/ECM1/EGFL7/F3/GPC3/GRN/HMOX1/ITGA4/JAML/MYC/NLRC3/NOG/PTCH1/THBS4/VEGFA/XBP1 | 16 |
| GO:0070302 | regulation of stress-activated protein kinase signaling cascade | 11/261 | 195/18723 | 9.54E-05 | 0.002510379 | 0.001832202 | FCGR2B/MYC/NCF1/PRDX1/PTPN22/SEMA4C/SH3RF3/TLR4/TNFRSF19/TREM2/VEGFA | 11 |
| GO:0002283 | neutrophil activation involved in immune response | 4/261 | 18/18723 | 9.68116E-05 | 0.002510379 | 0.001832202 | ITGB2/PRAM1/PTAFR/TYROBP | 4 |
| GO:0061687 | detoxification of inorganic compound | 4/261 | 18/18723 | 9.68116E-05 | 0.002510379 | 0.001832202 | MT1E/MT1G/MT1X/MT2A | 4 |
| GO:1900225 | regulation of NLRP3 inflammasome complex assembly | 4/261 | 18/18723 | 9.68116E-05 | 0.002510379 | 0.001832202 | CD36/NLRC3/TLR4/TREM2 | 4 |
| GO:0098751 | bone cell development | 5/261 | 34/18723 | 0.000101272 | 0.002607937 | 0.001903405 | ANXA2/GPR68/KIT/SRC/TYROBP | 5 |
| GO:0001706 | endoderm formation | 6/261 | 54/18723 | 0.000102146 | 0.002612411 | 0.00190667 | ITGA4/ITGA5/ITGB2/MMP2/MMP9/NOG | 6 |
| GO:0033674 | positive regulation of kinase activity | 18/261 | 467/18723 | 0.000104219 | 0.002647316 | 0.001932146 | AZU1/CCL5/CD86/CHI3L1/CSF1R/EGR1/EPHB4/FGFR3/GAS6/KIT/LRRK2/NCF1/PRLR/SRC/TGFBR2/TLR4/TREM2/VEGFA | 18 |
| GO:0050727 | regulation of inflammatory response | 16/261 | 386/18723 | 0.00011038 | 0.002784846 | 0.002032522 | ALOX5AP/C3/CCL5/CNR2/FCGR2B/FURIN/GRN/LRRK2/MMP9/NCF1/NLRC3/NLRP12/SRC/SUCNR1/TLR4/TREM2 | 16 |
| GO:0006909 | phagocytosis | 14/261 | 308/18723 | 0.00011411 | 0.002859641 | 0.002087111 | AZU1/C3/CD300LF/CD36/FCGR2B/FCN1/GAS6/GSN/ITGB2/P2RY6/SRC/TLR4/TREM2/TYROBP | 14 |
| GO:0002687 | positive regulation of leukocyte migration | 9/261 | 135/18723 | 0.000117485 | 0.002919487 | 0.00213079 | CCL5/CSF1R/CXCL10/GAS6/ITGA4/PTAFR/THBS4/TREM2/VEGFA | 9 |
| GO:0070372 | regulation of ERK1 and ERK2 cascade | 14/261 | 309/18723 | 0.000118062 | 0.002919487 | 0.00213079 | CCL5/CD36/CHI3L1/CSF1R/FGFR3/GAS6/NLRP12/P2RY6/PTPN22/RAP1B/SEMA6A/SRC/TLR4/TREM2 | 14 |
| GO:0002281 | macrophage activation involved in immune response | 4/261 | 19/18723 | 0.000121289 | 0.002921896 | 0.002132548 | GRN/SUCNR1/TREM2/TYROBP | 4 |
| GO:0002888 | positive regulation of myeloid leukocyte mediated immunity | 4/261 | 19/18723 | 0.000121289 | 0.002921896 | 0.002132548 | C3/ITGB2/PTAFR/TYROBP | 4 |
| GO:0044546 | NLRP3 inflammasome complex assembly | 4/261 | 19/18723 | 0.000121289 | 0.002921896 | 0.002132548 | CD36/NLRC3/TLR4/TREM2 | 4 |
| GO:0097501 | stress response to metal ion | 4/261 | 19/18723 | 0.000121289 | 0.002921896 | 0.002132548 | MT1E/MT1G/MT1X/MT2A | 4 |
| GO:1903557 | positive regulation of tumor necrosis factor superfamily cytokine production | 8/261 | 107/18723 | 0.000127072 | 0.003041585 | 0.002219903 | AZU1/CD36/CD86/CYBB/LRRK2/PTAFR/TLR4/TYROBP | 8 |
| GO:0030879 | mammary gland development | 9/261 | 137/18723 | 0.00013145 | 0.003126341 | 0.002281763 | CSF1R/PRLR/PTCH1/RXFP1/SLC29A1/SRC/TGFBR2/VEGFA/XBP1 | 9 |
| GO:0051403 | stress-activated MAPK cascade | 12/261 | 239/18723 | 0.00014001 | 0.003308839 | 0.002414959 | FCGR2B/LRRK2/MYC/NCF1/PRDX1/PTPN22/SEMA4C/SH3RF3/TLR4/TNFRSF19/TREM2/VEGFA | 12 |
| GO:0140632 | inflammasome complex assembly | 4/261 | 20/18723 | 0.000149957 | 0.00352163 | 0.002570265 | CD36/NLRC3/TLR4/TREM2 | 4 |
| GO:0010043 | response to zinc ion | 6/261 | 58/18723 | 0.000152808 | 0.0035321 | 0.002577906 | ASS1/CA2/MT1E/MT1G/MT1X/MT2A | 6 |
| GO:0048846 | axon extension involved in axon guidance | 5/261 | 37/18723 | 0.000153331 | 0.0035321 | 0.002577906 | SEMA4A/SEMA4C/SEMA6A/SEMA6B/VEGFA | 5 |
| GO:1902284 | neuron projection extension involved in neuron projection guidance | 5/261 | 37/18723 | 0.000153331 | 0.0035321 | 0.002577906 | SEMA4A/SEMA4C/SEMA6A/SEMA6B/VEGFA | 5 |
| GO:0006959 | humoral immune response | 14/261 | 317/18723 | 0.000154186 | 0.0035321 | 0.002577906 | A2M/AZU1/C3/CFH/CXCL10/FCER2/FCGR2B/FCN1/LYZ/MFAP4/PAX5/RNASE3/SLPI/TREM2 | 14 |
| GO:0002443 | leukocyte mediated immunity | 17/261 | 440/18723 | 0.000157563 | 0.00358745 | 0.002618303 | AZU1/C3/CD1D/FCER2/FCGR2B/HMOX1/ITGB2/KIT/LILRB4/PRAM1/PRDX1/PTAFR/SLC15A4/TLR4/TREM2/TYROBP/UNG | 17 |
| GO:1901214 | regulation of neuron death | 14/261 | 319/18723 | 0.000164586 | 0.003724636 | 0.002718429 | CCL5/EGR1/FCGR2B/GCLM/GRN/HMOX1/ITGB2/JUN/LRRK2/STAR/TERT/TLR4/TREM2/TYROBP | 14 |
| GO:0006898 | receptor-mediated endocytosis | 12/261 | 244/18723 | 0.000169832 | 0.003820191 | 0.00278817 | ANXA2/C3/CD36/CTTN/DNM1/FCGR2B/HSPG2/ITGA4/ITGB2/LILRB4/TGFBR2/VEGFA | 12 |
| GO:0048640 | negative regulation of developmental growth | 8/261 | 112/18723 | 0.000174629 | 0.003904571 | 0.002849754 | FGFR3/NOG/PTCH1/SEMA4A/SEMA4C/SEMA6A/SEMA6B/TGFBR2 | 8 |
| GO:0030449 | regulation of complement activation | 4/261 | 21/18723 | 0.00018322 | 0.004026624 | 0.002938835 | A2M/C3/CFH/TREM2 | 4 |
| GO:0072111 | cell proliferation involved in kidney development | 4/261 | 21/18723 | 0.00018322 | 0.004026624 | 0.002938835 | EGR1/GPC3/MYC/PTCH1 | 4 |
| GO:0002685 | regulation of leukocyte migration | 11/261 | 210/18723 | 0.000183322 | 0.004026624 | 0.002938835 | CCL5/CSF1R/CXCL10/ECM1/GAS6/HMOX1/ITGA4/PTAFR/THBS4/TREM2/VEGFA | 11 |
| GO:0044344 | cellular response to fibroblast growth factor stimulus | 8/261 | 113/18723 | 0.000185704 | 0.004055083 | 0.002959606 | CCL5/FGFR3/GCLM/NDST1/NOG/SHISA2/STAR/ZFP36L2 | 8 |
| GO:0031098 | stress-activated protein kinase signaling cascade | 12/261 | 247/18723 | 0.00019021 | 0.004129321 | 0.003013788 | FCGR2B/LRRK2/MYC/NCF1/PRDX1/PTPN22/SEMA4C/SH3RF3/TLR4/TNFRSF19/TREM2/VEGFA | 12 |
| GO:0009749 | response to glucose | 11/261 | 212/18723 | 0.000199079 | 0.004296874 | 0.003136077 | EGR1/GAS6/GCLM/GPR68/PCK2/SLC29A1/SOX4/STAR/TGFBR2/TREM2/XBP1 | 11 |
| GO:0034113 | heterotypic cell-cell adhesion | 6/261 | 61/18723 | 0.00020245 | 0.004319714 | 0.003152747 | CD1D/ITGA4/ITGA5/ITGB2/ITGB7/LILRB2 | 6 |
| GO:0043030 | regulation of macrophage activation | 6/261 | 61/18723 | 0.00020245 | 0.004319714 | 0.003152747 | BPI/FCGR2B/GRN/LRRK2/TLR4/TREM2 | 6 |
| GO:0046651 | lymphocyte proliferation | 13/261 | 288/18723 | 0.000213716 | 0.004534173 | 0.00330927 | CCL5/CD180/CD1D/CD86/FCGR2B/GAL/IGFBP2/LILRB2/LILRB4/PTPN22/TGFBR2/TLR4/TYROBP | 13 |
| GO:0048660 | regulation of smooth muscle cell proliferation | 10/261 | 180/18723 | 0.000223675 | 0.004718658 | 0.003443916 | CCL5/HMOX1/JUN/MMP2/MMP9/P2RY6/PTAFR/TERT/TGFBR2/TLR4 | 10 |
| GO:0050870 | positive regulation of T cell activation | 11/261 | 216/18723 | 0.000234054 | 0.004909879 | 0.003583479 | CCL5/CD1D/CD86/IGFBP2/LILRB2/LILRB4/PTPN22/SOX4/SRC/TGFBR2/XBP1 | 11 |
| GO:0032943 | mononuclear cell proliferation | 13/261 | 291/18723 | 0.000236373 | 0.004930824 | 0.003598766 | CCL5/CD180/CD1D/CD86/FCGR2B/GAL/IGFBP2/LILRB2/LILRB4/PTPN22/TGFBR2/TLR4/TYROBP | 13 |
| GO:0022617 | extracellular matrix disassembly | 6/261 | 63/18723 | 0.000242052 | 0.005021233 | 0.003664751 | ADAMTS5/CST3/FAP/FURIN/MMP2/MMP9 | 6 |
| GO:0001822 | kidney development | 13/261 | 293/18723 | 0.000252595 | 0.005211003 | 0.003803255 | ASS1/CA2/CYP26B1/EGR1/GPC3/LRRK2/MMP9/MYC/NOG/PTCH1/SOX4/TIPARP/VEGFA | 13 |
| GO:0070665 | positive regulation of leukocyte proliferation | 9/261 | 150/18723 | 0.000260274 | 0.005339903 | 0.003897333 | CCL5/CD1D/CD86/CSF1R/IGFBP2/LILRB2/PTPN22/TGFBR2/TLR4 | 9 |
| GO:0009746 | response to hexose | 11/261 | 219/18723 | 0.000263578 | 0.005357708 | 0.003910328 | EGR1/GAS6/GCLM/GPR68/PCK2/SLC29A1/SOX4/STAR/TGFBR2/TREM2/XBP1 | 11 |
| GO:0045670 | regulation of osteoclast differentiation | 6/261 | 64/18723 | 0.000264011 | 0.005357708 | 0.003910328 | CA2/GPR68/LILRB4/TLR4/TREM2/TYROBP | 6 |
| GO:0048659 | smooth muscle cell proliferation | 10/261 | 184/18723 | 0.000266976 | 0.005388596 | 0.003932871 | CCL5/HMOX1/JUN/MMP2/MMP9/P2RY6/PTAFR/TERT/TGFBR2/TLR4 | 10 |
| GO:0071333 | cellular response to glucose stimulus | 9/261 | 151/18723 | 0.000273461 | 0.005473053 | 0.003994512 | GAS6/GCLM/GPR68/PCK2/SLC29A1/SOX4/STAR/TREM2/XBP1 | 9 |
| GO:0097529 | myeloid leukocyte migration | 11/261 | 220/18723 | 0.000274092 | 0.005473053 | 0.003994512 | AZU1/CCL5/CSF1R/CXCL10/ITGB2/JAML/KIT/MCOLN2/THBS4/TREM2/VEGFA | 11 |
| GO:0002761 | regulation of myeloid leukocyte differentiation | 8/261 | 120/18723 | 0.000280507 | 0.005527245 | 0.004034064 | CA2/GPR68/JUN/LILRB4/MYC/TLR4/TREM2/TYROBP | 8 |
| GO:0071774 | response to fibroblast growth factor | 8/261 | 120/18723 | 0.000280507 | 0.005527245 | 0.004034064 | CCL5/FGFR3/GCLM/NDST1/NOG/SHISA2/STAR/ZFP36L2 | 8 |
| GO:0030574 | collagen catabolic process | 5/261 | 42/18723 | 0.000282727 | 0.005527245 | 0.004034064 | CST3/FAP/FURIN/MMP2/MMP9 | 5 |
| GO:0035019 | somatic stem cell population maintenance | 5/261 | 42/18723 | 0.000282727 | 0.005527245 | 0.004034064 | KIT/MYC/NOG/SOX4/ZFP36L2 | 5 |
| GO:0007599 | hemostasis | 11/261 | 222/18723 | 0.000296187 | 0.005665282 | 0.004134811 | ANXA2/CD36/F3/FAP/GAS6/HPS4/JMJD1C/NFE2/SRC/TLR4/TSPAN32 | 11 |
| GO:0045348 | positive regulation of MHC class II biosynthetic process | 3/261 | 10/18723 | 0.000298891 | 0.005665282 | 0.004134811 | AZU1/TLR4/XBP1 | 3 |
| GO:0070391 | response to lipoteichoic acid | 3/261 | 10/18723 | 0.000298891 | 0.005665282 | 0.004134811 | CD36/TLR4/TREM2 | 3 |
| GO:0071223 | cellular response to lipoteichoic acid | 3/261 | 10/18723 | 0.000298891 | 0.005665282 | 0.004134811 | CD36/TLR4/TREM2 | 3 |
| GO:1901725 | regulation of histone deacetylase activity | 3/261 | 10/18723 | 0.000298891 | 0.005665282 | 0.004134811 | CAMK2D/LRRK2/VEGFA | 3 |
| GO:2000425 | regulation of apoptotic cell clearance | 3/261 | 10/18723 | 0.000298891 | 0.005665282 | 0.004134811 | C3/CD300LF/TREM2 | 3 |
| GO:0071331 | cellular response to hexose stimulus | 9/261 | 153/18723 | 0.000301499 | 0.005685841 | 0.004149816 | GAS6/GCLM/GPR68/PCK2/SLC29A1/SOX4/STAR/TREM2/XBP1 | 9 |
| GO:0002922 | positive regulation of humoral immune response | 4/261 | 24/18723 | 0.000314776 | 0.005848428 | 0.004268481 | C3/FCER2/FCGR2B/TREM2 | 4 |
| GO:0071294 | cellular response to zinc ion | 4/261 | 24/18723 | 0.000314776 | 0.005848428 | 0.004268481 | MT1E/MT1G/MT1X/MT2A | 4 |
| GO:0002861 | regulation of inflammatory response to antigenic stimulus | 5/261 | 43/18723 | 0.000316322 | 0.005848428 | 0.004268481 | C3/FCGR2B/FURIN/SRC/TREM2 | 5 |
| GO:0071326 | cellular response to monosaccharide stimulus | 9/261 | 154/18723 | 0.000316385 | 0.005848428 | 0.004268481 | GAS6/GCLM/GPR68/PCK2/SLC29A1/SOX4/STAR/TREM2/XBP1 | 9 |
| GO:2001234 | negative regulation of apoptotic signaling pathway | 11/261 | 224/18723 | 0.000319764 | 0.005881764 | 0.004292811 | CTTN/GCLM/HMOX1/ITGA6/LMNA/LRRK2/MMP9/NOG/SRC/TERT/XBP1 | 11 |
| GO:0060993 | kidney morphogenesis | 7/261 | 93/18723 | 0.000322425 | 0.005901641 | 0.004307318 | GPC3/LRRK2/MYC/NOG/PTCH1/SOX4/VEGFA | 7 |
| GO:0034284 | response to monosaccharide | 11/261 | 225/18723 | 0.000332133 | 0.006049681 | 0.004415365 | EGR1/GAS6/GCLM/GPR68/PCK2/SLC29A1/SOX4/STAR/TGFBR2/TREM2/XBP1 | 11 |
| GO:0072001 | renal system development | 13/261 | 302/18723 | 0.000337927 | 0.006125334 | 0.00447058 | ASS1/CA2/CYP26B1/EGR1/GPC3/LRRK2/MMP9/MYC/NOG/PTCH1/SOX4/TIPARP/VEGFA | 13 |
| GO:0019221 | cytokine-mediated signaling pathway | 17/261 | 472/18723 | 0.000357335 | 0.006445843 | 0.004704504 | CCL5/CD300LF/CSF1R/CXCL10/ECM1/EGR1/F3/GAS6/GPR35/KIT/LILRB2/LILRB4/PRLR/SAMHD1/SRC/TNFRSF19/TREM2 | 17 |
| GO:0045860 | positive regulation of protein kinase activity | 15/261 | 386/18723 | 0.000359713 | 0.006457534 | 0.004713037 | AZU1/CCL5/CD86/CHI3L1/CSF1R/EGR1/GAS6/KIT/LRRK2/NCF1/PRLR/SRC/TGFBR2/TLR4/VEGFA | 15 |
| GO:0050764 | regulation of phagocytosis | 7/261 | 95/18723 | 0.000367324 | 0.006562624 | 0.004789737 | AZU1/C3/CD300LF/CD36/FCGR2B/GAS6/TREM2 | 7 |
| GO:0031667 | response to nutrient levels | 17/261 | 474/18723 | 0.000375048 | 0.006668715 | 0.004867167 | ASS1/CXCL10/CYBB/CYP26B1/GAS6/GCLM/HMOX1/IGFBP2/JUN/LRRK2/NUCB2/PCK2/SRC/STAR/STC2/TGFBR2/XBP1 | 17 |
| GO:0007565 | female pregnancy | 10/261 | 193/18723 | 0.00039055 | 0.006880568 | 0.005021789 | IGFBP2/JUNB/MMP2/MMP9/PRLR/STC2/STS/TGFBR2/TIMP1/VEGFA | 10 |
| GO:0035987 | endodermal cell differentiation | 5/261 | 45/18723 | 0.000392491 | 0.006880568 | 0.005021789 | ITGA4/ITGA5/ITGB2/MMP2/MMP9 | 5 |
| GO:1904646 | cellular response to amyloid-beta | 5/261 | 45/18723 | 0.000392491 | 0.006880568 | 0.005021789 | CD36/FCGR2B/ITGA4/TLR4/TREM2 | 5 |
| GO:0001666 | response to hypoxia | 13/261 | 307/18723 | 0.000395163 | 0.006895039 | 0.00503235 | CYBB/EGR1/HMOX1/LMNA/MMP2/MYC/SLC29A1/SRC/STC2/TERT/TGFBR2/TREM2/VEGFA | 13 |
| GO:0030595 | leukocyte chemotaxis | 11/261 | 230/18723 | 0.000400192 | 0.006917207 | 0.005048529 | AZU1/CCL5/CNR2/CSF1R/CXCL10/GAS6/ITGB2/JAML/KIT/THBS4/VEGFA | 11 |
| GO:0001867 | complement activation, lectin pathway | 3/261 | 11/18723 | 0.000406747 | 0.006917207 | 0.005048529 | A2M/FCN1/MFAP4 | 3 |
| GO:0002645 | positive regulation of tolerance induction | 3/261 | 11/18723 | 0.000406747 | 0.006917207 | 0.005048529 | LILRB2/LILRB4/TGFBR2 | 3 |
| GO:0043312 | neutrophil degranulation | 3/261 | 11/18723 | 0.000406747 | 0.006917207 | 0.005048529 | ITGB2/PRAM1/PTAFR | 3 |
| GO:0071492 | cellular response to UV-A | 3/261 | 11/18723 | 0.000406747 | 0.006917207 | 0.005048529 | MMP2/MMP9/TIMP1 | 3 |
| GO:0002440 | production of molecular mediator of immune response | 13/261 | 308/18723 | 0.000407548 | 0.006917207 | 0.005048529 | CD36/CD86/FCGR2B/HMOX1/KIT/LILRB4/PTPN22/SAMHD1/SLC15A4/SLC7A5/TLR4/UNG/XBP1 | 13 |
| GO:1901216 | positive regulation of neuron death | 7/261 | 97/18723 | 0.000417095 | 0.007015466 | 0.005120244 | EGR1/FCGR2B/GRN/ITGB2/JUN/TLR4/TYROBP | 7 |
| GO:2001237 | negative regulation of extrinsic apoptotic signaling pathway | 7/261 | 97/18723 | 0.000417095 | 0.007015466 | 0.005120244 | CTTN/GCLM/HMOX1/ITGA6/LMNA/SRC/TERT | 7 |
| GO:0045672 | positive regulation of osteoclast differentiation | 4/261 | 26/18723 | 0.000433276 | 0.00722256 | 0.005271392 | CA2/GPR68/TREM2/TYROBP | 4 |
| GO:0048843 | negative regulation of axon extension involved in axon guidance | 4/261 | 26/18723 | 0.000433276 | 0.00722256 | 0.005271392 | SEMA4A/SEMA4C/SEMA6A/SEMA6B | 4 |
| GO:0043300 | regulation of leukocyte degranulation | 5/261 | 46/18723 | 0.000435397 | 0.007225657 | 0.005273652 | FCGR2B/HMOX1/ITGB2/PRAM1/PTAFR | 5 |
| GO:0032722 | positive regulation of chemokine production | 6/261 | 71/18723 | 0.000465027 | 0.007683241 | 0.00560762 | AZU1/CSF1R/EGR1/HMOX1/MCOLN2/TLR4 | 6 |
| GO:0071322 | cellular response to carbohydrate stimulus | 9/261 | 163/18723 | 0.000479803 | 0.007855187 | 0.005733115 | GAS6/GCLM/GPR68/PCK2/SLC29A1/SOX4/STAR/TREM2/XBP1 | 9 |
| GO:0014911 | positive regulation of smooth muscle cell migration | 5/261 | 47/18723 | 0.000481746 | 0.007855187 | 0.005733115 | CCL5/P2RY6/SRC/TERT/TLR4 | 5 |
| GO:0097028 | dendritic cell differentiation | 5/261 | 47/18723 | 0.000481746 | 0.007855187 | 0.005733115 | FCGR2B/GAS6/LILRB2/TGFBR2/TREM2 | 5 |
| GO:0046683 | response to organophosphorus | 8/261 | 131/18723 | 0.000506554 | 0.008223792 | 0.006002141 | ASS1/JUN/P2RY6/PTAFR/RAP1B/RFC3/STAR/TRIM16 | 8 |
| GO:0022411 | cellular component disassembly | 16/261 | 443/18723 | 0.000515025 | 0.008325124 | 0.006076099 | ADAMTS5/ATG9A/C3/CST3/CTTN/FAP/FURIN/GSN/MMP2/MMP9/MYC/NFE2/TMOD2/TOP2A/TREM2/TWF1 | 16 |
| GO:0042102 | positive regulation of T cell proliferation | 7/261 | 101/18723 | 0.00053277 | 0.008456697 | 0.006172128 | CCL5/CD1D/CD86/IGFBP2/LILRB2/PTPN22/TGFBR2 | 7 |
| GO:0002664 | regulation of T cell tolerance induction | 3/261 | 12/18723 | 0.000536753 | 0.008456697 | 0.006172128 | LILRB2/LILRB4/TGFBR2 | 3 |
| GO:0033004 | negative regulation of mast cell activation | 3/261 | 12/18723 | 0.000536753 | 0.008456697 | 0.006172128 | CD300LF/CNR2/HMOX1 | 3 |
| GO:0042117 | monocyte activation | 3/261 | 12/18723 | 0.000536753 | 0.008456697 | 0.006172128 | AZU1/LILRB4/MT1G | 3 |
| GO:0140052 | cellular response to oxidised low-density lipoprotein particle stimulus | 3/261 | 12/18723 | 0.000536753 | 0.008456697 | 0.006172128 | CD36/TLR4/TREM2 | 3 |
| GO:0150065 | regulation of deacetylase activity | 3/261 | 12/18723 | 0.000536753 | 0.008456697 | 0.006172128 | CAMK2D/LRRK2/VEGFA | 3 |
| GO:0043299 | leukocyte degranulation | 6/261 | 73/18723 | 0.000540082 | 0.008473394 | 0.006184314 | FCGR2B/HMOX1/ITGB2/KIT/PRAM1/PTAFR | 6 |
| GO:0072593 | reactive oxygen species metabolic process | 11/261 | 239/18723 | 0.00055225 | 0.008628038 | 0.006297181 | CD36/CYBB/ITGB2/LRRK2/NCF1/NCF1B/NCF1C/PRDX1/TGFBR2/TLR4/TYROBP | 11 |
| GO:0032677 | regulation of interleukin-8 production | 7/261 | 102/18723 | 0.00056534 | 0.008795743 | 0.006419581 | ANXA4/BPI/CHI3L1/F3/FCN1/PTPN22/TLR4 | 7 |
| GO:0070997 | neuron death | 14/261 | 361/18723 | 0.000573874 | 0.008891468 | 0.006489445 | CCL5/EGR1/FCGR2B/GCLM/GRN/HMOX1/ITGB2/JUN/LRRK2/STAR/TERT/TLR4/TREM2/TYROBP | 14 |
| GO:0061082 | myeloid leukocyte cytokine production | 4/261 | 28/18723 | 0.000580562 | 0.008914256 | 0.006506077 | CD36/HMOX1/KIT/TLR4 | 4 |
| GO:0071280 | cellular response to copper ion | 4/261 | 28/18723 | 0.000580562 | 0.008914256 | 0.006506077 | MT1E/MT1G/MT1X/MT2A | 4 |
| GO:1904707 | positive regulation of vascular associated smooth muscle cell proliferation | 5/261 | 49/18723 | 0.000585472 | 0.008914256 | 0.006506077 | JUN/MMP2/MMP9/P2RY6/TERT | 5 |
| GO:0002705 | positive regulation of leukocyte mediated immunity | 8/261 | 134/18723 | 0.000588777 | 0.008914256 | 0.006506077 | C3/CD1D/FCER2/ITGB2/KIT/PTAFR/TREM2/TYROBP | 8 |
| GO:0032147 | activation of protein kinase activity | 8/261 | 134/18723 | 0.000588777 | 0.008914256 | 0.006506077 | CCL5/CD86/CHI3L1/GAS6/PRLR/SRC/TGFBR2/VEGFA | 8 |
| GO:0051251 | positive regulation of lymphocyte activation | 14/261 | 362/18723 | 0.000589668 | 0.008914256 | 0.006506077 | CCL5/CD1D/CD86/GAS6/IGFBP2/LILRB2/LILRB4/PTPN22/SOX4/SRC/TGFBR2/TLR4/TYROBP/XBP1 | 14 |
| GO:0002833 | positive regulation of response to biotic stimulus | 9/261 | 168/18723 | 0.000597094 | 0.008953725 | 0.006534884 | CCL5/CD180/CD1D/FCN1/GRN/SLC15A4/SRC/TLR4/TYROBP | 9 |
| GO:0032637 | interleukin-8 production | 7/261 | 103/18723 | 0.000599473 | 0.008953725 | 0.006534884 | ANXA4/BPI/CHI3L1/F3/FCN1/PTPN22/TLR4 | 7 |
| GO:0032760 | positive regulation of tumor necrosis factor production | 7/261 | 103/18723 | 0.000599473 | 0.008953725 | 0.006534884 | AZU1/CD36/CYBB/LRRK2/PTAFR/TLR4/TYROBP | 7 |
| GO:0036293 | response to decreased oxygen levels | 13/261 | 322/18723 | 0.000618771 | 0.009205147 | 0.006718384 | CYBB/EGR1/HMOX1/LMNA/MMP2/MYC/SLC29A1/SRC/STC2/TERT/TGFBR2/TREM2/VEGFA | 13 |
| GO:0060348 | bone development | 10/261 | 205/18723 | 0.000626242 | 0.009279323 | 0.006772522 | ANXA2/CYP26B1/FGFR3/GPR68/KIT/RFLNB/SRC/TGFBR2/TYROBP/XYLT1 | 10 |
| GO:0045621 | positive regulation of lymphocyte differentiation | 7/261 | 104/18723 | 0.000635223 | 0.009375184 | 0.006842486 | CD86/GAS6/LILRB2/LILRB4/SOX4/TGFBR2/XBP1 | 7 |
| GO:0002862 | negative regulation of inflammatory response to antigenic stimulus | 4/261 | 29/18723 | 0.000666131 | 0.009792655 | 0.007147178 | FCGR2B/FURIN/SRC/TREM2 | 4 |
| GO:0032720 | negative regulation of tumor necrosis factor production | 6/261 | 76/18723 | 0.000669942 | 0.009810051 | 0.007159874 | BPI/GAS6/NLRC3/PTPN22/TLR4/TREM2 | 6 |
| GO:0050671 | positive regulation of lymphocyte proliferation | 8/261 | 137/18723 | 0.000681468 | 0.009842506 | 0.007183562 | CCL5/CD1D/CD86/IGFBP2/LILRB2/PTPN22/TGFBR2/TLR4 | 8 |
| GO:0015801 | aromatic amino acid transport | 3/261 | 13/18723 | 0.00069061 | 0.009842506 | 0.007183562 | SLC15A4/SLC38A5/SLC7A5 | 3 |
| GO:0036005 | response to macrophage colony-stimulating factor | 3/261 | 13/18723 | 0.00069061 | 0.009842506 | 0.007183562 | CSF1R/TLR4/TREM2 | 3 |
| GO:0036006 | cellular response to macrophage colony-stimulating factor stimulus | 3/261 | 13/18723 | 0.00069061 | 0.009842506 | 0.007183562 | CSF1R/TLR4/TREM2 | 3 |
| GO:0060100 | positive regulation of phagocytosis, engulfment | 3/261 | 13/18723 | 0.00069061 | 0.009842506 | 0.007183562 | C3/CD36/TREM2 | 3 |
| GO:0070424 | regulation of nucleotide-binding oligomerization domain containing signaling pathway | 3/261 | 13/18723 | 0.00069061 | 0.009842506 | 0.007183562 | PTPN22/SLC15A4/TLR4 | 3 |
| GO:1905155 | positive regulation of membrane invagination | 3/261 | 13/18723 | 0.00069061 | 0.009842506 | 0.007183562 | C3/CD36/TREM2 | 3 |
| GO:0001678 | cellular glucose homeostasis | 9/261 | 172/18723 | 0.000706982 | 0.010037536 | 0.007325905 | GAS6/GCLM/GPR68/PCK2/SLC29A1/SOX4/STAR/TREM2/XBP1 | 9 |
| GO:0032946 | positive regulation of mononuclear cell proliferation | 8/261 | 138/18723 | 0.000714854 | 0.010110857 | 0.007379418 | CCL5/CD1D/CD86/IGFBP2/LILRB2/PTPN22/TGFBR2/TLR4 | 8 |
| GO:0007492 | endoderm development | 6/261 | 77/18723 | 0.000718204 | 0.010119894 | 0.007386014 | ITGA4/ITGA5/ITGB2/MMP2/MMP9/NOG | 6 |
| GO:0033002 | muscle cell proliferation | 11/261 | 248/18723 | 0.000749832 | 0.010514147 | 0.007673759 | CCL5/HMOX1/JUN/MMP2/MMP9/NOG/P2RY6/PTAFR/TERT/TGFBR2/TLR4 | 11 |
| GO:0050863 | regulation of T cell activation | 13/261 | 329/18723 | 0.000755105 | 0.010514147 | 0.007673759 | CCL5/CD1D/CD86/CYP26B1/FCGR2B/IGFBP2/LILRB2/LILRB4/PTPN22/SOX4/SRC/TGFBR2/XBP1 | 13 |
| GO:0034599 | cellular response to oxidative stress | 12/261 | 288/18723 | 0.000756841 | 0.010514147 | 0.007673759 | ALDH3B1/CD36/HMOX1/JUN/LRRK2/MMP2/MMP9/NCF1/PRDX1/SRC/TLR4/TREM2 | 12 |
| GO:0007202 | activation of phospholipase C activity | 4/261 | 30/18723 | 0.000760262 | 0.010514147 | 0.007673759 | CD86/P2RY6/PLCB2/RASGRP4 | 4 |
| GO:0070168 | negative regulation of biomineral tissue development | 4/261 | 30/18723 | 0.000760262 | 0.010514147 | 0.007673759 | ECM1/GAS6/RFLNB/SRGN | 4 |
| GO:0045619 | regulation of lymphocyte differentiation | 9/261 | 174/18723 | 0.000767816 | 0.010558308 | 0.00770599 | CD86/CYP26B1/GAS6/LILRB2/LILRB4/SOX4/TGFBR2/XBP1/ZFP36L2 | 9 |
| GO:1903556 | negative regulation of tumor necrosis factor superfamily cytokine production | 6/261 | 78/18723 | 0.000769111 | 0.010558308 | 0.00770599 | BPI/GAS6/NLRC3/PTPN22/TLR4/TREM2 | 6 |
| GO:0002253 | activation of immune response | 14/261 | 375/18723 | 0.000831072 | 0.011367119 | 0.008296302 | A2M/C3/CFH/FCGR2B/FCN1/FYB1/LILRB4/MFAP4/PRAM1/PTPN22/SRC/TLR4/TREM2/TYROBP | 14 |
| GO:0043113 | receptor clustering | 5/261 | 53/18723 | 0.00084213 | 0.011476322 | 0.008376004 | COLQ/GSN/ITGA4/ITGB2/ITGB7 | 5 |
| GO:0050729 | positive regulation of inflammatory response | 8/261 | 142/18723 | 0.000861827 | 0.011642753 | 0.008497474 | ALOX5AP/C3/GRN/LRRK2/NLRP12/SUCNR1/TLR4/TREM2 | 8 |
| GO:0110150 | negative regulation of biomineralization | 4/261 | 31/18723 | 0.000863414 | 0.011642753 | 0.008497474 | ECM1/GAS6/RFLNB/SRGN | 4 |
| GO:0042113 | B cell activation | 13/261 | 334/18723 | 0.000867272 | 0.011642753 | 0.008497474 | CD180/CD86/FCGR2B/HDAC9/ITGA4/KIT/SAMSN1/SLC15A4/TLR4/TYROBP/UNG/XBP1/ZFP36L2 | 13 |
| GO:0002517 | T cell tolerance induction | 3/261 | 14/18723 | 0.000869933 | 0.011642753 | 0.008497474 | LILRB2/LILRB4/TGFBR2 | 3 |
| GO:0070141 | response to UV-A | 3/261 | 14/18723 | 0.000869933 | 0.011642753 | 0.008497474 | MMP2/MMP9/TIMP1 | 3 |
| GO:0071229 | cellular response to acid chemical | 6/261 | 80/18723 | 0.000879253 | 0.011725463 | 0.00855784 | ASS1/CYBB/GCLM/MMP2/VEGFA/XBP1 | 6 |
| GO:0038061 | NIK/NF-kappaB signaling | 8/261 | 143/18723 | 0.000902117 | 0.011947483 | 0.008719881 | CD86/CHI3L1/NLRC3/NLRP12/PRDX1/PTPN22/TLR4/TREM2 | 8 |
| GO:0048015 | phosphatidylinositol-mediated signaling | 9/261 | 178/18723 | 0.000902301 | 0.011947483 | 0.008719881 | CCL5/CSF1R/KIT/NCF1/NLRC3/PLCB2/PTAFR/SRC/TREM2 | 9 |
| GO:0006968 | cellular defense response | 5/261 | 54/18723 | 0.000917638 | 0.012107637 | 0.00883677 | FCMR/LGALS3BP/LILRB2/NCF1/TYROBP | 5 |
| GO:0001938 | positive regulation of endothelial cell proliferation | 7/261 | 111/18723 | 0.000935428 | 0.012256753 | 0.008945602 | ECM1/EGFL7/F3/HMOX1/ITGA4/THBS4/VEGFA | 7 |
| GO:0009612 | response to mechanical stimulus | 10/261 | 216/18723 | 0.000935505 | 0.012256753 | 0.008945602 | CHI3L1/CXCL10/GSN/IGFBP2/JUN/KIT/PTCH1/SRC/TGFBR2/TLR4 | 10 |
| GO:0001655 | urogenital system development | 13/261 | 338/18723 | 0.000966791 | 0.01260624 | 0.009200675 | ASS1/CA2/CYP26B1/EGR1/GPC3/LRRK2/MMP9/MYC/NOG/PTCH1/SOX4/TIPARP/VEGFA | 13 |
| GO:0007596 | blood coagulation | 10/261 | 217/18723 | 0.000968932 | 0.01260624 | 0.009200675 | ANXA2/CD36/F3/FAP/GAS6/HPS4/JMJD1C/SRC/TLR4/TSPAN32 | 10 |
| GO:1901222 | regulation of NIK/NF-kappaB signaling | 7/261 | 112/18723 | 0.000986148 | 0.012758859 | 0.009312064 | CD86/NLRC3/NLRP12/PRDX1/PTPN22/TLR4/TREM2 | 7 |
| GO:0000768 | syncytium formation by plasma membrane fusion | 5/261 | 55/18723 | 0.000998069 | 0.012758859 | 0.009312064 | ADAMTS5/CD109/CXCL10/TREM2/TYROBP | 5 |
| GO:0061098 | positive regulation of protein tyrosine kinase activity | 5/261 | 55/18723 | 0.000998069 | 0.012758859 | 0.009312064 | CCL5/CSF1R/GAS6/NCF1/SRC | 5 |
| GO:0140253 | cell-cell fusion | 5/261 | 55/18723 | 0.000998069 | 0.012758859 | 0.009312064 | ADAMTS5/CD109/CXCL10/TREM2/TYROBP | 5 |
| GO:1905517 | macrophage migration | 5/261 | 55/18723 | 0.000998069 | 0.012758859 | 0.009312064 | AZU1/CCL5/CSF1R/MCOLN2/TREM2 | 5 |
| GO:0002312 | B cell activation involved in immune response | 6/261 | 82/18723 | 0.001001164 | 0.012758859 | 0.009312064 | CD180/FCGR2B/SLC15A4/TLR4/UNG/XBP1 | 6 |
| GO:0042593 | glucose homeostasis | 11/261 | 258/18723 | 0.001034952 | 0.013144601 | 0.009593598 | GAS6/GCLM/GPR68/PCK2/PTCH1/SLC29A1/SOX4/STAR/SUCNR1/TREM2/XBP1 | 11 |
| GO:0097191 | extrinsic apoptotic signaling pathway | 10/261 | 219/18723 | 0.001038727 | 0.013147817 | 0.009595946 | BCL2A1/CTTN/FGFR3/GCLM/HMOX1/ITGA6/LMNA/SORT1/SRC/TERT | 10 |
| GO:0048017 | inositol lipid-mediated signaling | 9/261 | 182/18723 | 0.00105533 | 0.013263186 | 0.009680148 | CCL5/CSF1R/KIT/NCF1/NLRC3/PLCB2/PTAFR/SRC/TREM2 | 9 |
| GO:0050672 | negative regulation of lymphocyte proliferation | 6/261 | 83/18723 | 0.001066788 | 0.013263186 | 0.009680148 | CD86/FCGR2B/GAL/LILRB2/LILRB4/TYROBP | 6 |
| GO:0033500 | carbohydrate homeostasis | 11/261 | 259/18723 | 0.00106782 | 0.013263186 | 0.009680148 | GAS6/GCLM/GPR68/PCK2/PTCH1/SLC29A1/SOX4/STAR/SUCNR1/TREM2/XBP1 | 11 |
| GO:0048705 | skeletal system morphogenesis | 10/261 | 220/18723 | 0.001075138 | 0.013263186 | 0.009680148 | CYP26B1/FGFR3/HOXC4/MMP2/NDST1/NOG/PAX5/RFLNB/TGFBR2/TIPARP | 10 |
| GO:0002679 | respiratory burst involved in defense response | 3/261 | 15/18723 | 0.001076257 | 0.013263186 | 0.009680148 | GRN/NCF1/TREM2 | 3 |
| GO:0023035 | CD40 signaling pathway | 3/261 | 15/18723 | 0.001076257 | 0.013263186 | 0.009680148 | CD86/ITGA5/TREM2 | 3 |
| GO:0045346 | regulation of MHC class II biosynthetic process | 3/261 | 15/18723 | 0.001076257 | 0.013263186 | 0.009680148 | AZU1/TLR4/XBP1 | 3 |
| GO:0060099 | regulation of phagocytosis, engulfment | 3/261 | 15/18723 | 0.001076257 | 0.013263186 | 0.009680148 | C3/CD36/TREM2 | 3 |
| GO:0001658 | branching involved in ureteric bud morphogenesis | 5/261 | 56/18723 | 0.001083619 | 0.01330998 | 0.0097143 | GPC3/MYC/NOG/PTCH1/VEGFA | 5 |
| GO:0032945 | negative regulation of mononuclear cell proliferation | 6/261 | 84/18723 | 0.001135663 | 0.0139035 | 0.010147481 | CD86/FCGR2B/GAL/LILRB2/LILRB4/TYROBP | 6 |
| GO:0000302 | response to reactive oxygen species | 10/261 | 222/18723 | 0.001151097 | 0.013958808 | 0.010187848 | CD36/HMOX1/JUN/LRRK2/MMP2/MMP9/NCF1/PRDX1/SRC/STAR | 10 |
| GO:0050817 | coagulation | 10/261 | 222/18723 | 0.001151097 | 0.013958808 | 0.010187848 | ANXA2/CD36/F3/FAP/GAS6/HPS4/JMJD1C/SRC/TLR4/TSPAN32 | 10 |
| GO:0046916 | cellular transition metal ion homeostasis | 7/261 | 115/18723 | 0.001151396 | 0.013958808 | 0.010187848 | FTH1/HMOX1/MT1E/MT1G/MT1X/MT2A/MYC | 7 |
| GO:0009636 | response to toxic substance | 11/261 | 262/18723 | 0.001171632 | 0.014101388 | 0.01029191 | ALOX5AP/ASS1/CCL5/CD36/MT1E/MT1G/MT1X/MT2A/PRDX1/PTGS1/STAR | 11 |
| GO:0031330 | negative regulation of cellular catabolic process | 11/261 | 262/18723 | 0.001171632 | 0.014101388 | 0.01029191 | ANXA2/CST3/FURIN/HMOX1/LRRK2/PTPN22/QSOX1/RBM24/TENT5A/TIMP1/TREM2 | 11 |
| GO:0006949 | syncytium formation | 5/261 | 57/18723 | 0.001174486 | 0.014101388 | 0.01029191 | ADAMTS5/CD109/CXCL10/TREM2/TYROBP | 5 |
| GO:0070482 | response to oxygen levels | 13/261 | 347/18723 | 0.001226122 | 0.014553212 | 0.010621674 | CYBB/EGR1/HMOX1/LMNA/MMP2/MYC/SLC29A1/SRC/STC2/TERT/TGFBR2/TREM2/VEGFA | 13 |
| GO:0002446 | neutrophil mediated immunity | 4/261 | 34/18723 | 0.001231606 | 0.014553212 | 0.010621674 | AZU1/ITGB2/PRAM1/PTAFR | 4 |
| GO:0034383 | low-density lipoprotein particle clearance | 4/261 | 34/18723 | 0.001231606 | 0.014553212 | 0.010621674 | ANXA2/CD36/HMOX1/TREM2 | 4 |
| GO:0055094 | response to lipoprotein particle | 4/261 | 34/18723 | 0.001231606 | 0.014553212 | 0.010621674 | CD36/ITGB2/TLR4/TREM2 | 4 |
| GO:1990000 | amyloid fibril formation | 4/261 | 34/18723 | 0.001231606 | 0.014553212 | 0.010621674 | CD36/FURIN/GSN/TREM2 | 4 |
| GO:0032868 | response to insulin | 11/261 | 264/18723 | 0.001245355 | 0.014669264 | 0.010706375 | EGR1/GAL/HDAC9/PCK2/PIK3R3/SORT1/SRC/STAR/TBC1D4/TRIB3/XBP1 | 11 |
| GO:0002763 | positive regulation of myeloid leukocyte differentiation | 5/261 | 58/18723 | 0.001270871 | 0.014855555 | 0.01084234 | CA2/GPR68/JUN/TREM2/TYROBP | 5 |
| GO:0002702 | positive regulation of production of molecular mediator of immune response | 7/261 | 117/18723 | 0.001273106 | 0.014855555 | 0.01084234 | CD36/CD86/KIT/PTPN22/SLC7A5/TLR4/XBP1 | 7 |
| GO:0014902 | myotube differentiation | 7/261 | 117/18723 | 0.001273106 | 0.014855555 | 0.01084234 | ADAMTS5/CXCL10/CYP26B1/HDAC9/RBM24/SORT1/XBP1 | 7 |
| GO:0044409 | entry into host | 8/261 | 151/18723 | 0.001281767 | 0.014910021 | 0.010882092 | CD86/FCN1/GAS6/GSN/ITGA5/ITGB7/SRC/TRIM14 | 8 |
| GO:0045342 | MHC class II biosynthetic process | 3/261 | 16/18723 | 0.001311042 | 0.015062859 | 0.010993641 | AZU1/TLR4/XBP1 | 3 |
| GO:0051770 | positive regulation of nitric-oxide synthase biosynthetic process | 3/261 | 16/18723 | 0.001311042 | 0.015062859 | 0.010993641 | FCER2/LRRK2/TLR4 | 3 |
| GO:0070431 | nucleotide-binding oligomerization domain containing 2 signaling pathway | 3/261 | 16/18723 | 0.001311042 | 0.015062859 | 0.010993641 | PTPN22/SLC15A4/TLR4 | 3 |
| GO:1905153 | regulation of membrane invagination | 3/261 | 16/18723 | 0.001311042 | 0.015062859 | 0.010993641 | C3/CD36/TREM2 | 3 |
| GO:0001906 | cell killing | 9/261 | 188/18723 | 0.001323695 | 0.015161582 | 0.011065694 | AZU1/C3/CD1D/CFH/FCER2/FCGR2B/LYZ/PRDX1/TYROBP | 9 |
| GO:0098754 | detoxification | 8/261 | 152/18723 | 0.001337021 | 0.015267385 | 0.011142914 | ALOX5AP/CD36/MT1E/MT1G/MT1X/MT2A/PRDX1/PTGS1 | 8 |
| GO:0050829 | defense response to Gram-negative bacterium | 6/261 | 88/18723 | 0.001445817 | 0.016459393 | 0.012012902 | AZU1/BPI/LYZ/RNASE3/TLR4/TREM2 | 6 |
| GO:0008360 | regulation of cell shape | 8/261 | 154/18723 | 0.001453209 | 0.016493255 | 0.012037616 | CDC42EP1/CSF1R/ITGB2/KIT/MYO10/SEMA4A/STRIP2/VEGFA | 8 |
| GO:0002712 | regulation of B cell mediated immunity | 5/261 | 60/18723 | 0.001481003 | 0.016707149 | 0.012193727 | C3/FCER2/FCGR2B/SLC15A4/TREM2 | 5 |
| GO:0002889 | regulation of immunoglobulin mediated immune response | 5/261 | 60/18723 | 0.001481003 | 0.016707149 | 0.012193727 | C3/FCER2/FCGR2B/SLC15A4/TREM2 | 5 |
| GO:0019932 | second-messenger-mediated signaling | 12/261 | 312/18723 | 0.001502933 | 0.016903473 | 0.012337015 | AZU1/CAMK2D/CAP2/CD36/GAL/HOMER3/KSR1/LRRK2/NCALD/RASD1/TREM2/VEGFA | 12 |
| GO:0034614 | cellular response to reactive oxygen species | 8/261 | 155/18723 | 0.001514234 | 0.016979424 | 0.012392447 | CD36/JUN/LRRK2/MMP2/MMP9/NCF1/PRDX1/SRC | 8 |
| GO:0071402 | cellular response to lipoprotein particle stimulus | 4/261 | 36/18723 | 0.001530615 | 0.017111724 | 0.012489007 | CD36/ITGB2/TLR4/TREM2 | 4 |
| GO:1904019 | epithelial cell apoptotic process | 7/261 | 121/18723 | 0.001546602 | 0.017238841 | 0.012581783 | GAS6/GSN/HMOX1/ITGA4/SORT1/TERT/TGFBR2 | 7 |
| GO:0006979 | response to oxidative stress | 15/261 | 446/18723 | 0.001557969 | 0.017313858 | 0.012636534 | ALDH3B1/CD36/GCLM/HMOX1/JUN/LRRK2/MMP2/MMP9/NCF1/PRDX1/PTGS1/SRC/STAR/TLR4/TREM2 | 15 |
| GO:0010631 | epithelial cell migration | 13/261 | 357/18723 | 0.001579893 | 0.017505399 | 0.01277633 | EPHB4/FAP/GRN/HDAC9/HMOX1/KIT/MMP9/PIK3R3/S100P/SEMA4A/SRC/TGFBR2/VEGFA | 13 |
| GO:0061097 | regulation of protein tyrosine kinase activity | 6/261 | 90/18723 | 0.001623216 | 0.017826734 | 0.013010858 | CCL5/CSF1R/GAS6/LILRB4/NCF1/SRC | 6 |
| GO:0070664 | negative regulation of leukocyte proliferation | 6/261 | 90/18723 | 0.001623216 | 0.017826734 | 0.013010858 | CD86/FCGR2B/GAL/LILRB2/LILRB4/TYROBP | 6 |
| GO:1904705 | regulation of vascular associated smooth muscle cell proliferation | 6/261 | 90/18723 | 0.001623216 | 0.017826734 | 0.013010858 | HMOX1/JUN/MMP2/MMP9/P2RY6/TERT | 6 |
| GO:0051250 | negative regulation of lymphocyte activation | 8/261 | 157/18723 | 0.001642377 | 0.017984267 | 0.013125833 | CD86/FCGR2B/GAL/LILRB2/LILRB4/PTPN22/SAMSN1/TYROBP | 8 |
| GO:0090132 | epithelium migration | 13/261 | 360/18723 | 0.001701205 | 0.018485858 | 0.01349192 | EPHB4/FAP/GRN/HDAC9/HMOX1/KIT/MMP9/PIK3R3/S100P/SEMA4A/SRC/TGFBR2/VEGFA | 13 |
| GO:0032613 | interleukin-10 production | 5/261 | 62/18723 | 0.001715651 | 0.018485858 | 0.01349192 | FCGR2B/LILRB4/TLR4/TREM2/TYROBP | 5 |
| GO:0032653 | regulation of interleukin-10 production | 5/261 | 62/18723 | 0.001715651 | 0.018485858 | 0.01349192 | FCGR2B/LILRB4/TLR4/TREM2/TYROBP | 5 |
| GO:0060675 | ureteric bud morphogenesis | 5/261 | 62/18723 | 0.001715651 | 0.018485858 | 0.01349192 | GPC3/MYC/NOG/PTCH1/VEGFA | 5 |
| GO:0045582 | positive regulation of T cell differentiation | 6/261 | 91/18723 | 0.001717888 | 0.018485858 | 0.01349192 | CD86/LILRB2/LILRB4/SOX4/TGFBR2/XBP1 | 6 |
| GO:1990874 | vascular associated smooth muscle cell proliferation | 6/261 | 91/18723 | 0.001717888 | 0.018485858 | 0.01349192 | HMOX1/JUN/MMP2/MMP9/P2RY6/TERT | 6 |
| GO:0043393 | regulation of protein binding | 9/261 | 196/18723 | 0.001764409 | 0.018877655 | 0.013777873 | ANXA2/ITGA4/LRRK2/MMP9/NOG/SLPI/SRC/TERT/TRIB3 | 9 |
| GO:0071674 | mononuclear cell migration | 9/261 | 196/18723 | 0.001764409 | 0.018877655 | 0.013777873 | CCL5/CSF1R/CXCL10/ECM1/GAS6/ITGA4/ITGB7/JAML/NLRP12 | 9 |
| GO:0072171 | mesonephric tubule morphogenesis | 5/261 | 63/18723 | 0.001842686 | 0.019658826 | 0.014348011 | GPC3/MYC/NOG/PTCH1/VEGFA | 5 |
| GO:0002643 | regulation of tolerance induction | 3/261 | 18/18723 | 0.001871436 | 0.019740065 | 0.014407304 | LILRB2/LILRB4/TGFBR2 | 3 |
| GO:0002923 | regulation of humoral immune response mediated by circulating immunoglobulin | 3/261 | 18/18723 | 0.001871436 | 0.019740065 | 0.014407304 | FCER2/FCGR2B/TREM2 | 3 |
| GO:0006882 | cellular zinc ion homeostasis | 4/261 | 38/18723 | 0.001876734 | 0.019740065 | 0.014407304 | MT1E/MT1G/MT1X/MT2A | 4 |
| GO:0009595 | detection of biotic stimulus | 4/261 | 38/18723 | 0.001876734 | 0.019740065 | 0.014407304 | CD1D/FAP/TLR4/TREM2 | 4 |
| GO:0097242 | amyloid-beta clearance | 4/261 | 38/18723 | 0.001876734 | 0.019740065 | 0.014407304 | C3/CD36/ITGB2/TREM2 | 4 |
| GO:0032755 | positive regulation of interleukin-6 production | 6/261 | 93/18723 | 0.001919745 | 0.020087256 | 0.014660701 | CD36/LILRB2/PTAFR/TLR4/TYROBP/XBP1 | 6 |
| GO:0090130 | tissue migration | 13/261 | 365/18723 | 0.001920501 | 0.020087256 | 0.014660701 | EPHB4/FAP/GRN/HDAC9/HMOX1/KIT/MMP9/PIK3R3/S100P/SEMA4A/SRC/TGFBR2/VEGFA | 13 |
| GO:0032874 | positive regulation of stress-activated MAPK cascade | 7/261 | 126/18723 | 0.001950527 | 0.020344323 | 0.014848322 | FCGR2B/NCF1/SEMA4C/SH3RF3/TLR4/TNFRSF19/VEGFA | 7 |
| GO:0070542 | response to fatty acid | 5/261 | 64/18723 | 0.001976472 | 0.02055751 | 0.015003916 | ASS1/CD36/PTAFR/SCD/SRC | 5 |
| GO:0002690 | positive regulation of leukocyte chemotaxis | 6/261 | 94/18723 | 0.002027163 | 0.021026182 | 0.015345977 | CCL5/CSF1R/CXCL10/GAS6/THBS4/VEGFA | 6 |
| GO:0010463 | mesenchymal cell proliferation | 4/261 | 39/18723 | 0.002068591 | 0.021337344 | 0.015573079 | GPC3/LMNA/MYC/TGFBR2 | 4 |
| GO:0071548 | response to dexamethasone | 4/261 | 39/18723 | 0.002068591 | 0.021337344 | 0.015573079 | ASS1/PCK2/PTAFR/STAR | 4 |
| GO:0070304 | positive regulation of stress-activated protein kinase signaling cascade | 7/261 | 128/18723 | 0.002133227 | 0.021943014 | 0.016015128 | FCGR2B/NCF1/SEMA4C/SH3RF3/TLR4/TNFRSF19/VEGFA | 7 |
| GO:0030516 | regulation of axon extension | 6/261 | 95/18723 | 0.002139062 | 0.021943014 | 0.016015128 | CTTN/SEMA4A/SEMA4C/SEMA6A/SEMA6B/VEGFA | 6 |
| GO:0009410 | response to xenobiotic stimulus | 15/261 | 462/18723 | 0.002189602 | 0.022379469 | 0.016333675 | ASS1/CYBB/CYP26B1/GAL/GAS6/GCLM/HMOX1/IGFBP2/JUN/MYC/PTCH1/RAP1B/SRC/STAR/TGFBR2 | 15 |
| GO:0043031 | negative regulation of macrophage activation | 3/261 | 19/18723 | 0.002199589 | 0.022379469 | 0.016333675 | BPI/FCGR2B/GRN | 3 |
| GO:1903978 | regulation of microglial cell activation | 3/261 | 19/18723 | 0.002199589 | 0.022379469 | 0.016333675 | GRN/LRRK2/TREM2 | 3 |
| GO:0051235 | maintenance of location | 12/261 | 327/18723 | 0.002221677 | 0.022504399 | 0.016424855 | C3/CAMK2D/CD36/CXCL10/FTH1/GM2A/GSN/MCOLN2/P2RY6/SRGN/TREM2/TWF1 | 12 |
| GO:0002718 | regulation of cytokine production involved in immune response | 6/261 | 96/18723 | 0.002255561 | 0.022504399 | 0.016424855 | CD36/HMOX1/KIT/LILRB4/SLC7A5/TLR4 | 6 |
| GO:0043648 | dicarboxylic acid metabolic process | 6/261 | 96/18723 | 0.002255561 | 0.022504399 | 0.016424855 | ALDH1L2/ASS1/GCLM/GPT2/ME1/PCK2 | 6 |
| GO:1901655 | cellular response to ketone | 6/261 | 96/18723 | 0.002255561 | 0.022504399 | 0.016424855 | ASS1/GAS6/P2RY6/PCK2/SRC/STAR | 6 |
| GO:1904035 | regulation of epithelial cell apoptotic process | 6/261 | 96/18723 | 0.002255561 | 0.022504399 | 0.016424855 | GAS6/GSN/HMOX1/ITGA4/SORT1/TERT | 6 |
| GO:0050918 | positive chemotaxis | 5/261 | 66/18723 | 0.002265138 | 0.022504399 | 0.016424855 | AZU1/CCL5/CXCL10/F3/VEGFA | 5 |
| GO:0072678 | T cell migration | 5/261 | 66/18723 | 0.002265138 | 0.022504399 | 0.016424855 | CCL5/CXCL10/ECM1/ITGA4/ITGB7 | 5 |
| GO:0055069 | zinc ion homeostasis | 4/261 | 40/18723 | 0.002273578 | 0.022504399 | 0.016424855 | MT1E/MT1G/MT1X/MT2A | 4 |
| GO:0071526 | semaphorin-plexin signaling pathway | 4/261 | 40/18723 | 0.002273578 | 0.022504399 | 0.016424855 | SEMA4A/SEMA4C/SEMA6A/SEMA6B | 4 |
| GO:0150077 | regulation of neuroinflammatory response | 4/261 | 40/18723 | 0.002273578 | 0.022504399 | 0.016424855 | GRN/LRRK2/MMP9/TREM2 | 4 |
| GO:0030111 | regulation of Wnt signaling pathway | 12/261 | 328/18723 | 0.002278164 | 0.022504399 | 0.016424855 | ATP6V1C2/CMAHP/DIXDC1/EGR1/GPC3/IGFBP2/LRRK2/NOG/SHISA2/SOX4/SRC/TERT | 12 |
| GO:0031960 | response to corticosteroid | 8/261 | 167/18723 | 0.002417485 | 0.023783997 | 0.017358771 | ASS1/CYBB/IGFBP2/PCK2/PTAFR/SRC/STAR/ZFP36L2 | 8 |
| GO:1903317 | regulation of protein maturation | 5/261 | 67/18723 | 0.002420439 | 0.023783997 | 0.017358771 | CHAC1/GSN/LRRK2/SOX4/SRC | 5 |
| GO:0045089 | positive regulation of innate immune response | 7/261 | 131/18723 | 0.002431756 | 0.023832489 | 0.017394163 | CCL5/CD1D/FCN1/SLC15A4/SRC/TLR4/TYROBP | 7 |
| GO:0002764 | immune response-regulating signaling pathway | 15/261 | 468/18723 | 0.002475722 | 0.024199854 | 0.017662285 | CD300LF/CD36/FCGR2B/FCN1/FYB1/KIT/LILRB2/LILRB4/PRAM1/PTPN22/SLC15A4/SRC/TLR4/TREM2/TYROBP | 15 |
| GO:0002367 | cytokine production involved in immune response | 6/261 | 98/18723 | 0.002502834 | 0.024400999 | 0.01780909 | CD36/HMOX1/KIT/LILRB4/SLC7A5/TLR4 | 6 |
| GO:0002577 | regulation of antigen processing and presentation | 3/261 | 20/18723 | 0.002561289 | 0.024776819 | 0.018083383 | FCGR2B/LILRB2/TREM2 | 3 |
| GO:0051004 | regulation of lipoprotein lipase activity | 3/261 | 20/18723 | 0.002561289 | 0.024776819 | 0.018083383 | FURIN/HDAC9/SORT1 | 3 |
| GO:0051900 | regulation of mitochondrial depolarization | 3/261 | 20/18723 | 0.002561289 | 0.024776819 | 0.018083383 | GCLM/LRRK2/SRC | 3 |
| GO:0044403 | biological process involved in symbiotic interaction | 11/261 | 290/18723 | 0.002603495 | 0.025055285 | 0.018286622 | AZU1/CCL5/CD86/FCN1/GAS6/GSN/ITGA5/ITGB7/JUN/SRC/TRIM14 | 11 |
| GO:0071375 | cellular response to peptide hormone stimulus | 11/261 | 290/18723 | 0.002603495 | 0.025055285 | 0.018286622 | ASS1/CA2/HDAC9/PCK2/PIK3R3/RAP1B/SRC/STAR/TBC1D4/TRIB3/XBP1 | 11 |
| GO:0043122 | regulation of I-kappaB kinase/NF-kappaB signaling | 10/261 | 249/18723 | 0.002680078 | 0.025725997 | 0.018776141 | CD36/ECM1/HMOX1/LILRB4/NLRC3/NLRP12/TLR4/TNFRSF19/TREM2/TRIM14 | 10 |
| GO:0046688 | response to copper ion | 4/261 | 42/18723 | 0.002724706 | 0.02602059 | 0.01899115 | MT1E/MT1G/MT1X/MT2A | 4 |
| GO:2000404 | regulation of T cell migration | 4/261 | 42/18723 | 0.002724706 | 0.02602059 | 0.01899115 | CCL5/CXCL10/ECM1/ITGA4 | 4 |
| GO:0008277 | regulation of G protein-coupled receptor signaling pathway | 7/261 | 134/18723 | 0.002761474 | 0.026304449 | 0.019198325 | ACP3/C3/CCL5/DNM1/GRK6/RASGRP4/TMOD2 | 7 |
| GO:0042129 | regulation of T cell proliferation | 8/261 | 171/18723 | 0.00279775 | 0.026582183 | 0.01940103 | CCL5/CD1D/CD86/IGFBP2/LILRB2/LILRB4/PTPN22/TGFBR2 | 8 |
| GO:0062197 | cellular response to chemical stress | 12/261 | 337/18723 | 0.002841328 | 0.026927709 | 0.019653212 | ALDH3B1/CD36/HMOX1/JUN/LRRK2/MMP2/MMP9/NCF1/PRDX1/SRC/TLR4/TREM2 | 12 |
| GO:1901136 | carbohydrate derivative catabolic process | 8/261 | 172/18723 | 0.002899739 | 0.027117418 | 0.019791671 | ADA2/CHI3L1/CST3/FUCA1/GM2A/GNS/SAMHD1/UNG | 8 |
| GO:0030100 | regulation of endocytosis | 9/261 | 211/18723 | 0.002903017 | 0.027117418 | 0.019791671 | ANXA2/C3/CD36/DNM1/GPC3/LRRK2/SRC/TREM2/VEGFA | 9 |
| GO:0061448 | connective tissue development | 10/261 | 252/18723 | 0.002920467 | 0.027117418 | 0.019791671 | CHI3L1/ECM1/EGR1/FGFR3/NOG/RFLNB/RXFP1/TGFBR2/TIMP1/XBP1 | 10 |
| GO:0045123 | cellular extravasation | 5/261 | 70/18723 | 0.002932754 | 0.027117418 | 0.019791671 | AZU1/ITGA4/ITGB7/JAML/PTAFR | 5 |
| GO:0030502 | negative regulation of bone mineralization | 3/261 | 21/18723 | 0.002957633 | 0.027117418 | 0.019791671 | ECM1/RFLNB/SRGN | 3 |
| GO:0045655 | regulation of monocyte differentiation | 3/261 | 21/18723 | 0.002957633 | 0.027117418 | 0.019791671 | GPR68/JUN/MYC | 3 |
| GO:0051767 | nitric-oxide synthase biosynthetic process | 3/261 | 21/18723 | 0.002957633 | 0.027117418 | 0.019791671 | FCER2/LRRK2/TLR4 | 3 |
| GO:0051769 | regulation of nitric-oxide synthase biosynthetic process | 3/261 | 21/18723 | 0.002957633 | 0.027117418 | 0.019791671 | FCER2/LRRK2/TLR4 | 3 |
| GO:0061081 | positive regulation of myeloid leukocyte cytokine production involved in immune response | 3/261 | 21/18723 | 0.002957633 | 0.027117418 | 0.019791671 | CD36/KIT/TLR4 | 3 |
| GO:0071498 | cellular response to fluid shear stress | 3/261 | 21/18723 | 0.002957633 | 0.027117418 | 0.019791671 | ASS1/CA2/SRC | 3 |
| GO:0097062 | dendritic spine maintenance | 3/261 | 21/18723 | 0.002957633 | 0.027117418 | 0.019791671 | CTTN/FCGR2B/TREM2 | 3 |
| GO:0009268 | response to pH | 4/261 | 43/18723 | 0.002971719 | 0.027117418 | 0.019791671 | CA2/GNA11/GPR68/SRC | 4 |
| GO:0010171 | body morphogenesis | 4/261 | 43/18723 | 0.002971719 | 0.027117418 | 0.019791671 | GPC3/MMP2/NOG/TIPARP | 4 |
| GO:0030517 | negative regulation of axon extension | 4/261 | 43/18723 | 0.002971719 | 0.027117418 | 0.019791671 | SEMA4A/SEMA4C/SEMA6A/SEMA6B | 4 |
| GO:0060443 | mammary gland morphogenesis | 4/261 | 43/18723 | 0.002971719 | 0.027117418 | 0.019791671 | CSF1R/PTCH1/SRC/TGFBR2 | 4 |
| GO:0042692 | muscle cell differentiation | 13/261 | 384/18723 | 0.002977542 | 0.027117418 | 0.019791671 | ADAMTS5/ASF1A/CXCL10/CYP26B1/HDAC9/KIT/LMNA/RBM24/SEMA4C/SORT1/TMOD2/VEGFA/XBP1 | 13 |
| GO:1903305 | regulation of regulated secretory pathway | 7/261 | 136/18723 | 0.002999641 | 0.02725221 | 0.019890049 | FCGR2B/HMOX1/ITGB2/LRRK2/PRAM1/PTAFR/RAP1B | 7 |
| GO:0061515 | myeloid cell development | 5/261 | 71/18723 | 0.003119703 | 0.028137614 | 0.020536262 | ANXA2/GPR68/KIT/SRC/TYROBP | 5 |
| GO:0071230 | cellular response to amino acid stimulus | 5/261 | 71/18723 | 0.003119703 | 0.028137614 | 0.020536262 | ASS1/CYBB/GCLM/MMP2/XBP1 | 5 |
| GO:0072078 | nephron tubule morphogenesis | 5/261 | 71/18723 | 0.003119703 | 0.028137614 | 0.020536262 | GPC3/MYC/NOG/PTCH1/VEGFA | 5 |
| GO:0048771 | tissue remodeling | 8/261 | 175/18723 | 0.003223185 | 0.028885846 | 0.02108236 | CA2/CSF1R/CST3/MMP2/SRC/THBS4/TIMP1/TPP1 | 8 |
| GO:0052126 | movement in host environment | 8/261 | 175/18723 | 0.003223185 | 0.028885846 | 0.02108236 | CD86/FCN1/GAS6/GSN/ITGA5/ITGB7/SRC/TRIM14 | 8 |
| GO:0031295 | T cell costimulation | 4/261 | 44/18723 | 0.003233606 | 0.028885846 | 0.02108236 | CD86/LILRB2/LILRB4/SRC | 4 |
| GO:0150076 | neuroinflammatory response | 4/261 | 44/18723 | 0.003233606 | 0.028885846 | 0.02108236 | GRN/LRRK2/MMP9/TREM2 | 4 |
| GO:0055076 | transition metal ion homeostasis | 7/261 | 138/18723 | 0.003253223 | 0.028991731 | 0.02115964 | FTH1/HMOX1/MT1E/MT1G/MT1X/MT2A/MYC | 7 |
| GO:0033627 | cell adhesion mediated by integrin | 5/261 | 72/18723 | 0.003315094 | 0.029472768 | 0.021510725 | CCL5/ITGA4/ITGA5/ITGB2/ITGB7 | 5 |
| GO:0050728 | negative regulation of inflammatory response | 8/261 | 176/18723 | 0.00333701 | 0.029597138 | 0.021601497 | CNR2/FCGR2B/FURIN/GRN/NLRC3/NLRP12/SRC/TREM2 | 8 |
| GO:0032963 | collagen metabolic process | 6/261 | 104/18723 | 0.003367555 | 0.029781114 | 0.021735772 | CST3/FAP/FURIN/MFAP4/MMP2/MMP9 | 6 |
| GO:0002220 | innate immune response activating cell surface receptor signaling pathway | 3/261 | 22/18723 | 0.003389655 | 0.029781114 | 0.021735772 | FCN1/SRC/TYROBP | 3 |
| GO:0032693 | negative regulation of interleukin-10 production | 3/261 | 22/18723 | 0.003389655 | 0.029781114 | 0.021735772 | FCGR2B/LILRB4/TYROBP | 3 |
| GO:1900120 | regulation of receptor binding | 3/261 | 22/18723 | 0.003389655 | 0.029781114 | 0.021735772 | ANXA2/MMP9/NOG | 3 |
| GO:0031348 | negative regulation of defense response | 10/261 | 258/18723 | 0.003452861 | 0.030203714 | 0.022044207 | A2M/CNR2/FCGR2B/FURIN/GRN/NLRC3/NLRP12/SAMHD1/SRC/TREM2 | 10 |
| GO:0043405 | regulation of MAP kinase activity | 8/261 | 177/18723 | 0.003453933 | 0.030203714 | 0.022044207 | KIT/KSR1/LRRK2/PTPN22/SRC/TLR4/TRIB3/VEGFA | 8 |
| GO:0003254 | regulation of membrane depolarization | 4/261 | 45/18723 | 0.003510788 | 0.030590065 | 0.022326186 | CAMK2D/GCLM/LRRK2/SRC | 4 |
| GO:0072088 | nephron epithelium morphogenesis | 5/261 | 73/18723 | 0.003519139 | 0.030590065 | 0.022326186 | GPC3/MYC/NOG/PTCH1/VEGFA | 5 |
| GO:0030177 | positive regulation of Wnt signaling pathway | 7/261 | 140/18723 | 0.003522858 | 0.030590065 | 0.022326186 | ATP6V1C2/DIXDC1/GPC3/LRRK2/SOX4/SRC/TERT | 7 |
| GO:0062207 | regulation of pattern recognition receptor signaling pathway | 6/261 | 105/18723 | 0.003530883 | 0.030590065 | 0.022326186 | CD300LF/CD36/PTPN22/SLC15A4/TLR4/TREM2 | 6 |
| GO:0034764 | positive regulation of transmembrane transport | 9/261 | 219/18723 | 0.003712347 | 0.032035573 | 0.023381191 | C3/CA2/CXCL10/GAL/GPC3/P2RY6/PTAFR/TERT/TREM2 | 9 |
| GO:0002437 | inflammatory response to antigenic stimulus | 5/261 | 74/18723 | 0.00373205 | 0.032035573 | 0.023381191 | C3/FCGR2B/FURIN/SRC/TREM2 | 5 |
| GO:0007040 | lysosome organization | 5/261 | 74/18723 | 0.00373205 | 0.032035573 | 0.023381191 | ACP3/GRN/HPS4/LRRK2/TPP1 | 5 |
| GO:0080171 | lytic vacuole organization | 5/261 | 74/18723 | 0.00373205 | 0.032035573 | 0.023381191 | ACP3/GRN/HPS4/LRRK2/TPP1 | 5 |
| GO:0010830 | regulation of myotube differentiation | 4/261 | 46/18723 | 0.003803682 | 0.032426823 | 0.023666745 | CYP26B1/HDAC9/RBM24/XBP1 | 4 |
| GO:0031294 | lymphocyte costimulation | 4/261 | 46/18723 | 0.003803682 | 0.032426823 | 0.023666745 | CD86/LILRB2/LILRB4/SRC | 4 |
| GO:0050919 | negative chemotaxis | 4/261 | 46/18723 | 0.003803682 | 0.032426823 | 0.023666745 | SEMA4A/SEMA4C/SEMA6A/SEMA6B | 4 |
| GO:0007033 | vacuole organization | 8/261 | 180/18723 | 0.003823851 | 0.032448152 | 0.023682312 | ACP3/ANXA2/ATG9A/GRN/HPS4/LRRK2/SYNPO2/TPP1 | 8 |
| GO:0010721 | negative regulation of cell development | 8/261 | 180/18723 | 0.003823851 | 0.032448152 | 0.023682312 | NOG/RFLNB/SEMA4A/SEMA4C/SEMA6A/SEMA6B/TREM2/VEGFA | 8 |
| GO:0002758 | innate immune response-activating signal transduction | 3/261 | 23/18723 | 0.003858323 | 0.032448152 | 0.023682312 | FCN1/SRC/TYROBP | 3 |
| GO:0035162 | embryonic hemopoiesis | 3/261 | 23/18723 | 0.003858323 | 0.032448152 | 0.023682312 | KIT/TGFBR2/VEGFA | 3 |
| GO:0051882 | mitochondrial depolarization | 3/261 | 23/18723 | 0.003858323 | 0.032448152 | 0.023682312 | GCLM/LRRK2/SRC | 3 |
| GO:1903306 | negative regulation of regulated secretory pathway | 3/261 | 23/18723 | 0.003858323 | 0.032448152 | 0.023682312 | FCGR2B/HMOX1/RAP1B | 3 |
| GO:0034121 | regulation of toll-like receptor signaling pathway | 5/261 | 75/18723 | 0.003954037 | 0.033103978 | 0.024160967 | CD300LF/CD36/PTPN22/SLC15A4/TREM2 | 5 |
| GO:0061333 | renal tubule morphogenesis | 5/261 | 75/18723 | 0.003954037 | 0.033103978 | 0.024160967 | GPC3/MYC/NOG/PTCH1/VEGFA | 5 |
| GO:0007595 | lactation | 4/261 | 47/18723 | 0.004112699 | 0.034127624 | 0.024908076 | PRLR/SLC29A1/VEGFA/XBP1 | 4 |
| GO:0043618 | regulation of transcription from RNA polymerase II promoter in response to stress | 4/261 | 47/18723 | 0.004112699 | 0.034127624 | 0.024908076 | EGR1/HMOX1/JUN/VEGFA | 4 |
| GO:1900744 | regulation of p38MAPK cascade | 4/261 | 47/18723 | 0.004112699 | 0.034127624 | 0.024908076 | NCF1/PTPN22/TREM2/VEGFA | 4 |
| GO:0046718 | viral entry into host cell | 7/261 | 144/18723 | 0.004112863 | 0.034127624 | 0.024908076 | CD86/FCN1/GAS6/GSN/ITGA5/ITGB7/TRIM14 | 7 |
| GO:0072028 | nephron morphogenesis | 5/261 | 76/18723 | 0.004185311 | 0.034575116 | 0.025234679 | GPC3/MYC/NOG/PTCH1/VEGFA | 5 |
| GO:2000379 | positive regulation of reactive oxygen species metabolic process | 5/261 | 76/18723 | 0.004185311 | 0.034575116 | 0.025234679 | CD36/ITGB2/TGFBR2/TLR4/TYROBP | 5 |
| GO:0002819 | regulation of adaptive immune response | 8/261 | 183/18723 | 0.004223688 | 0.034815122 | 0.025409847 | C3/CD1D/FCER2/FCGR2B/LILRB4/SAMSN1/SLC15A4/TREM2 | 8 |
| GO:0060326 | cell chemotaxis | 11/261 | 310/18723 | 0.004311358 | 0.035459496 | 0.025880144 | AZU1/CCL5/CNR2/CSF1R/CXCL10/GAS6/ITGB2/JAML/KIT/THBS4/VEGFA | 11 |
| GO:2001233 | regulation of apoptotic signaling pathway | 12/261 | 356/18723 | 0.004403483 | 0.03602696 | 0.026294309 | CTTN/GCLM/HMOX1/ITGA6/LMNA/LRRK2/MMP9/NOG/SRC/TERT/TREM2/XBP1 | 12 |
| GO:0045580 | regulation of T cell differentiation | 7/261 | 146/18723 | 0.004434539 | 0.03602696 | 0.026294309 | CD86/CYP26B1/LILRB2/LILRB4/SOX4/TGFBR2/XBP1 | 7 |
| GO:0014812 | muscle cell migration | 6/261 | 110/18723 | 0.00443728 | 0.03602696 | 0.026294309 | CCL5/P2RY6/SRC/TERT/THBS4/TLR4 | 6 |
| GO:0061387 | regulation of extent of cell growth | 6/261 | 110/18723 | 0.00443728 | 0.03602696 | 0.026294309 | CTTN/SEMA4A/SEMA4C/SEMA6A/SEMA6B/VEGFA | 6 |
| GO:1905954 | positive regulation of lipid localization | 6/261 | 110/18723 | 0.00443728 | 0.03602696 | 0.026294309 | ANXA2/C3/CD36/GAL/PTCH1/TREM2 | 6 |
| GO:0090311 | regulation of protein deacetylation | 4/261 | 48/18723 | 0.004438244 | 0.03602696 | 0.026294309 | CAMK2D/FRY/LRRK2/VEGFA | 4 |
| GO:0007369 | gastrulation | 8/261 | 185/18723 | 0.004507636 | 0.036510873 | 0.026647492 | GPC3/ITGA4/ITGA5/ITGB2/MMP2/MMP9/NOG/TGFBR2 | 8 |
| GO:0014066 | regulation of phosphatidylinositol 3-kinase signaling | 6/261 | 111/18723 | 0.004637386 | 0.037480519 | 0.027355189 | CCL5/KIT/NCF1/NLRC3/SRC/TREM2 | 6 |
| GO:0046777 | protein autophosphorylation | 9/261 | 227/18723 | 0.004688982 | 0.037815674 | 0.027599803 | CAMK2D/CSF1R/EPHB4/FGFR3/KIT/LRRK2/NLRP12/SRC/VEGFA | 9 |
| GO:0097530 | granulocyte migration | 7/261 | 148/18723 | 0.004774876 | 0.038307253 | 0.027958582 | CCL5/CSF1R/CXCL10/ITGB2/JAML/MCOLN2/THBS4 | 7 |
| GO:0002762 | negative regulation of myeloid leukocyte differentiation | 4/261 | 49/18723 | 0.004780712 | 0.038307253 | 0.027958582 | GPR68/LILRB4/MYC/TLR4 | 4 |
| GO:0032692 | negative regulation of interleukin-1 production | 4/261 | 49/18723 | 0.004780712 | 0.038307253 | 0.027958582 | GAS6/LILRB4/NLRP12/TREM2 | 4 |
| GO:0030318 | melanocyte differentiation | 3/261 | 25/18723 | 0.004909166 | 0.038918952 | 0.028405031 | GNA11/HPS4/KIT | 3 |
| GO:0050927 | positive regulation of positive chemotaxis | 3/261 | 25/18723 | 0.004909166 | 0.038918952 | 0.028405031 | AZU1/F3/VEGFA | 3 |
| GO:0070423 | nucleotide-binding oligomerization domain containing signaling pathway | 3/261 | 25/18723 | 0.004909166 | 0.038918952 | 0.028405031 | PTPN22/SLC15A4/TLR4 | 3 |
| GO:0098581 | detection of external biotic stimulus | 3/261 | 25/18723 | 0.004909166 | 0.038918952 | 0.028405031 | CD1D/TLR4/TREM2 | 3 |
| GO:1904996 | positive regulation of leukocyte adhesion to vascular endothelial cell | 3/261 | 25/18723 | 0.004909166 | 0.038918952 | 0.028405031 | ITGA4/ITGB2/PTAFR | 3 |
| GO:0014068 | positive regulation of phosphatidylinositol 3-kinase signaling | 5/261 | 79/18723 | 0.004936952 | 0.039055912 | 0.028504992 | CCL5/KIT/NCF1/SRC/TREM2 | 5 |
| GO:0001503 | ossification | 13/261 | 408/18723 | 0.004947361 | 0.039055912 | 0.028504992 | ASF1A/ECM1/FGFR3/GPC3/JUNB/MMP2/MMP9/NOG/PTCH1/RFLNB/SORT1/SRGN/XYLT1 | 13 |
| GO:0009314 | response to radiation | 14/261 | 456/18723 | 0.004975095 | 0.039191991 | 0.028604309 | B4GALT2/CXCL10/DTL/EGR1/GNA11/GPR88/JUN/KIT/MFAP4/MMP2/MMP9/MYC/STAR/TIMP1 | 14 |
| GO:0019058 | viral life cycle | 11/261 | 317/18723 | 0.005085521 | 0.039977549 | 0.029177649 | CCL5/CD86/FCN1/FURIN/GAS6/GSN/ITGA5/ITGB7/SLPI/TOP2A/TRIM14 | 11 |
| GO:0001570 | vasculogenesis | 5/261 | 80/18723 | 0.005207465 | 0.040764519 | 0.02975202 | EGFL7/JUNB/TGFBR2/TIPARP/VEGFA | 5 |
| GO:0048145 | regulation of fibroblast proliferation | 5/261 | 80/18723 | 0.005207465 | 0.040764519 | 0.02975202 | ANXA2/FTH1/GAS6/MYC/NLRC3 | 5 |
| GO:0048754 | branching morphogenesis of an epithelial tube | 7/261 | 151/18723 | 0.005321827 | 0.041485806 | 0.030278452 | GPC3/MYC/NOG/PTCH1/SRC/TGFBR2/VEGFA | 7 |
| GO:2001236 | regulation of extrinsic apoptotic signaling pathway | 7/261 | 151/18723 | 0.005321827 | 0.041485806 | 0.030278452 | CTTN/GCLM/HMOX1/ITGA6/LMNA/SRC/TERT | 7 |
| GO:0009895 | negative regulation of catabolic process | 11/261 | 320/18723 | 0.005449278 | 0.041878292 | 0.030564908 | ANXA2/CST3/FURIN/HMOX1/LRRK2/PTPN22/QSOX1/RBM24/TENT5A/TIMP1/TREM2 | 11 |
| GO:0001960 | negative regulation of cytokine-mediated signaling pathway | 5/261 | 81/18723 | 0.005488306 | 0.041878292 | 0.030564908 | CCL5/ECM1/GAS6/SAMHD1/TREM2 | 5 |
| GO:0048144 | fibroblast proliferation | 5/261 | 81/18723 | 0.005488306 | 0.041878292 | 0.030564908 | ANXA2/FTH1/GAS6/MYC/NLRC3 | 5 |
| GO:0003416 | endochondral bone growth | 3/261 | 26/18723 | 0.005492978 | 0.041878292 | 0.030564908 | ECM1/FGFR3/TGFBR2 | 3 |
| GO:0035872 | nucleotide-binding domain, leucine rich repeat containing receptor signaling pathway | 3/261 | 26/18723 | 0.005492978 | 0.041878292 | 0.030564908 | PTPN22/SLC15A4/TLR4 | 3 |
| GO:0045745 | positive regulation of G protein-coupled receptor signaling pathway | 3/261 | 26/18723 | 0.005492978 | 0.041878292 | 0.030564908 | ACP3/C3/TMOD2 | 3 |
| GO:0050926 | regulation of positive chemotaxis | 3/261 | 26/18723 | 0.005492978 | 0.041878292 | 0.030564908 | AZU1/F3/VEGFA | 3 |
| GO:0070977 | bone maturation | 3/261 | 26/18723 | 0.005492978 | 0.041878292 | 0.030564908 | FGFR3/RFLNB/XYLT1 | 3 |
| GO:0097066 | response to thyroid hormone | 3/261 | 26/18723 | 0.005492978 | 0.041878292 | 0.030564908 | ANXA2/GCLM/KIT | 3 |
| GO:1905523 | positive regulation of macrophage migration | 3/261 | 26/18723 | 0.005492978 | 0.041878292 | 0.030564908 | CCL5/CSF1R/TREM2 | 3 |
| GO:0071675 | regulation of mononuclear cell migration | 6/261 | 115/18723 | 0.005504745 | 0.041878292 | 0.030564908 | CCL5/CSF1R/CXCL10/ECM1/GAS6/ITGA4 | 6 |
| GO:0043124 | negative regulation of I-kappaB kinase/NF-kappaB signaling | 4/261 | 51/18723 | 0.005517975 | 0.041878292 | 0.030564908 | LILRB4/NLRC3/NLRP12/TREM2 | 4 |
| GO:0048013 | ephrin receptor signaling pathway | 4/261 | 51/18723 | 0.005517975 | 0.041878292 | 0.030564908 | EPHB4/MMP2/MMP9/SRC | 4 |
| GO:0002218 | activation of innate immune response | 4/261 | 52/18723 | 0.005913527 | 0.044789272 | 0.032689489 | FCN1/SRC/TLR4/TYROBP | 4 |
| GO:0002285 | lymphocyte activation involved in immune response | 8/261 | 194/18723 | 0.005971145 | 0.04493079 | 0.032792776 | CD180/CD86/FCGR2B/SEMA4A/SLC15A4/TLR4/UNG/XBP1 | 8 |
| GO:1901654 | response to ketone | 8/261 | 194/18723 | 0.005971145 | 0.04493079 | 0.032792776 | ASS1/CYBB/GAS6/P2RY6/PCK2/PTAFR/SRC/STAR | 8 |
| GO:0030301 | cholesterol transport | 6/261 | 117/18723 | 0.005980343 | 0.04493079 | 0.032792776 | ANXA2/CD36/FURIN/PTCH1/STAR/TREM2 | 6 |
| GO:0072676 | lymphocyte migration | 6/261 | 117/18723 | 0.005980343 | 0.04493079 | 0.032792776 | CCL5/CXCL10/ECM1/GAS6/ITGA4/ITGB7 | 6 |
| GO:0051899 | membrane depolarization | 5/261 | 83/18723 | 0.006081787 | 0.045497624 | 0.03320648 | CAMK2D/GCLM/JUN/LRRK2/SRC | 5 |
| GO:0060562 | epithelial tube morphogenesis | 11/261 | 325/18723 | 0.006100944 | 0.045497624 | 0.03320648 | CSF1R/CXCL10/GPC3/MYC/NOG/PTCH1/SEMA4C/SOX4/SRC/TGFBR2/VEGFA | 11 |
| GO:0007263 | nitric oxide mediated signal transduction | 3/261 | 27/18723 | 0.006116713 | 0.045497624 | 0.03320648 | CD36/RASD1/VEGFA | 3 |
| GO:0060142 | regulation of syncytium formation by plasma membrane fusion | 3/261 | 27/18723 | 0.006116713 | 0.045497624 | 0.03320648 | CXCL10/TREM2/TYROBP | 3 |
| GO:1903319 | positive regulation of protein maturation | 3/261 | 27/18723 | 0.006116713 | 0.045497624 | 0.03320648 | GSN/SOX4/SRC | 3 |
| GO:0031334 | positive regulation of protein-containing complex assembly | 9/261 | 237/18723 | 0.006178685 | 0.045867215 | 0.033476227 | CD36/CDC42EP1/CTTN/GSN/IQGAP2/RAP1B/SRC/TLR4/VEGFA | 9 |
| GO:0022612 | gland morphogenesis | 6/261 | 118/18723 | 0.006229061 | 0.046149431 | 0.033682202 | CSF1R/NOG/PTCH1/SRC/TGFBR2/XBP1 | 6 |
| GO:0007249 | I-kappaB kinase/NF-kappaB signaling | 10/261 | 281/18723 | 0.006245588 | 0.046180246 | 0.033704693 | CD36/ECM1/HMOX1/LILRB4/NLRC3/NLRP12/TLR4/TNFRSF19/TREM2/TRIM14 | 10 |
| GO:0034381 | plasma lipoprotein particle clearance | 4/261 | 53/18723 | 0.006327519 | 0.046509756 | 0.033945186 | ANXA2/CD36/HMOX1/TREM2 | 4 |
| GO:0043620 | regulation of DNA-templated transcription in response to stress | 4/261 | 53/18723 | 0.006327519 | 0.046509756 | 0.033945186 | EGR1/HMOX1/JUN/VEGFA | 4 |
| GO:0071320 | cellular response to cAMP | 4/261 | 53/18723 | 0.006327519 | 0.046509756 | 0.033945186 | ASS1/PTAFR/RAP1B/STAR | 4 |
| GO:0001763 | morphogenesis of a branching structure | 8/261 | 196/18723 | 0.006340491 | 0.046513543 | 0.03394795 | GPC3/LRRK2/MYC/NOG/PTCH1/SRC/TGFBR2/VEGFA | 8 |
| GO:0051146 | striated muscle cell differentiation | 10/261 | 283/18723 | 0.006553522 | 0.047982062 | 0.03501975 | ADAMTS5/CXCL10/CYP26B1/HDAC9/LMNA/RBM24/SORT1/TMOD2/VEGFA/XBP1 | 10 |
| GO:0010507 | negative regulation of autophagy | 5/261 | 85/18723 | 0.006719017 | 0.04888117 | 0.035675964 | HMOX1/LRRK2/PTPN22/QSOX1/TREM2 | 5 |
| GO:0032092 | positive regulation of protein binding | 5/261 | 85/18723 | 0.006719017 | 0.04888117 | 0.035675964 | ANXA2/LRRK2/MMP9/TERT/TRIB3 | 5 |
| GO:0048675 | axon extension | 6/261 | 120/18723 | 0.006748956 | 0.04888117 | 0.035675964 | CTTN/SEMA4A/SEMA4C/SEMA6A/SEMA6B/VEGFA | 6 |
| GO:1902893 | regulation of pri-miRNA transcription by RNA polymerase II | 4/261 | 54/18723 | 0.006760309 | 0.04888117 | 0.035675964 | EGR1/JUN/LILRB4/TERT | 4 |
| GO:0002507 | tolerance induction | 3/261 | 28/18723 | 0.006781051 | 0.04888117 | 0.035675964 | LILRB2/LILRB4/TGFBR2 | 3 |
| GO:0010464 | regulation of mesenchymal cell proliferation | 3/261 | 28/18723 | 0.006781051 | 0.04888117 | 0.035675964 | LMNA/MYC/TGFBR2 | 3 |
| GO:0060325 | face morphogenesis | 3/261 | 28/18723 | 0.006781051 | 0.04888117 | 0.035675964 | MMP2/NOG/TIPARP | 3 |
| GO:1990776 | response to angiotensin | 3/261 | 28/18723 | 0.006781051 | 0.04888117 | 0.035675964 | CA2/CYBB/SRC | 3 |
| GO:0048638 | regulation of developmental growth | 11/261 | 330/18723 | 0.006812446 | 0.049012856 | 0.035772075 | COLQ/CTTN/FGFR3/NOG/PTCH1/SEMA4A/SEMA4C/SEMA6A/SEMA6B/TGFBR2/VEGFA | 11 |
| GO:0042098 | T cell proliferation | 8/261 | 199/18723 | 0.006926707 | 0.049739086 | 0.036302115 | CCL5/CD1D/CD86/IGFBP2/LILRB2/LILRB4/PTPN22/TGFBR2 | 8 |
| GO:0071214 | cellular response to abiotic stimulus | 11/261 | 331/18723 | 0.006962243 | 0.049802711 | 0.036348552 | EGR1/GNA11/GPR68/GPR88/MFAP4/MMP2/MMP9/MYC/PTAFR/TIMP1/TLR4 | 11 |
| GO:0104004 | cellular response to environmental stimulus | 11/261 | 331/18723 | 0.006962243 | 0.049802711 | 0.036348552 | EGR1/GNA11/GPR68/GPR88/MFAP4/MMP2/MMP9/MYC/PTAFR/TIMP1/TLR4 | 11 |
| GO:0001704 | formation of primary germ layer | 6/261 | 121/18723 | 0.007020384 | 0.049931643 | 0.036442653 | ITGA4/ITGA5/ITGB2/MMP2/MMP9/NOG | 6 |
| GO:0002224 | toll-like receptor signaling pathway | 6/261 | 121/18723 | 0.007020384 | 0.049931643 | 0.036442653 | CD300LF/CD36/PTPN22/SLC15A4/TLR4/TREM2 | 6 |
| GO:0048706 | embryonic skeletal system development | 6/261 | 121/18723 | 0.007020384 | 0.049931643 | 0.036442653 | HOXC4/NDST1/NOG/PAX5/TGFBR2/XYLT1 | 6 |
| GO:0034103 | regulation of tissue remodeling | 5/261 | 86/18723 | 0.007054537 | 0.049984136 | 0.036480966 | CA2/CSF1R/CST3/SRC/THBS4 | 5 |
| GO:0060761 | negative regulation of response to cytokine stimulus | 5/261 | 86/18723 | 0.007054537 | 0.049984136 | 0.036480966 | CCL5/ECM1/GAS6/SAMHD1/TREM2 | 5 |
| GO:0001755 | neural crest cell migration | 4/261 | 55/18723 | 0.00721225 | 0.05071665 | 0.037015591 | SEMA4A/SEMA4C/SEMA6A/SEMA6B | 4 |
| GO:0038066 | p38MAPK cascade | 4/261 | 55/18723 | 0.00721225 | 0.05071665 | 0.037015591 | NCF1/PTPN22/TREM2/VEGFA | 4 |
| GO:0061614 | pri-miRNA transcription by RNA polymerase II | 4/261 | 55/18723 | 0.00721225 | 0.05071665 | 0.037015591 | EGR1/JUN/LILRB4/TERT | 4 |
| GO:0098900 | regulation of action potential | 4/261 | 55/18723 | 0.00721225 | 0.05071665 | 0.037015591 | CAMK2D/CD36/CNR2/GPR35 | 4 |
| GO:0043254 | regulation of protein-containing complex assembly | 13/261 | 428/18723 | 0.007291564 | 0.051042725 | 0.037253577 | CD36/CDC42EP1/CTTN/GSN/IQGAP2/NLRC3/RAP1B/SRC/TLR4/TMOD2/TREM2/TWF1/VEGFA | 13 |
| GO:0002688 | regulation of leukocyte chemotaxis | 6/261 | 122/18723 | 0.007299629 | 0.051042725 | 0.037253577 | CCL5/CSF1R/CXCL10/GAS6/THBS4/VEGFA | 6 |
| GO:1990266 | neutrophil migration | 6/261 | 122/18723 | 0.007299629 | 0.051042725 | 0.037253577 | CCL5/CXCL10/ITGB2/JAML/MCOLN2/THBS4 | 6 |
| GO:0150104 | transport across blood-brain barrier | 5/261 | 87/18723 | 0.00740159 | 0.051658947 | 0.037703327 | CD36/SLC29A1/SLC38A5/SLC44A1/SLC7A5 | 5 |
| GO:0071549 | cellular response to dexamethasone stimulus | 3/261 | 29/18723 | 0.007486619 | 0.051961033 | 0.037923805 | ASS1/PCK2/STAR | 3 |
| GO:0098868 | bone growth | 3/261 | 29/18723 | 0.007486619 | 0.051961033 | 0.037923805 | ECM1/FGFR3/TGFBR2 | 3 |
| GO:2000406 | positive regulation of T cell migration | 3/261 | 29/18723 | 0.007486619 | 0.051961033 | 0.037923805 | CCL5/CXCL10/ITGA4 | 3 |
| GO:0071482 | cellular response to light stimulus | 6/261 | 123/18723 | 0.007586816 | 0.052558761 | 0.038360058 | GPR88/MFAP4/MMP2/MMP9/MYC/TIMP1 | 6 |
| GO:0048008 | platelet-derived growth factor receptor signaling pathway | 4/261 | 56/18723 | 0.007683683 | 0.053033036 | 0.038706208 | F3/SRC/TIPARP/VEGFA | 4 |
| GO:0071385 | cellular response to glucocorticoid stimulus | 4/261 | 56/18723 | 0.007683683 | 0.053033036 | 0.038706208 | ASS1/PCK2/STAR/ZFP36L2 | 4 |
| GO:0060759 | regulation of response to cytokine stimulus | 7/261 | 162/18723 | 0.007735956 | 0.053144484 | 0.038787548 | CCL5/CD300LF/ECM1/GAS6/SAMHD1/TLR4/TREM2 | 7 |
| GO:0010232 | vascular transport | 5/261 | 88/18723 | 0.007760371 | 0.053144484 | 0.038787548 | CD36/SLC29A1/SLC38A5/SLC44A1/SLC7A5 | 5 |
| GO:0032869 | cellular response to insulin stimulus | 8/261 | 203/18723 | 0.007770993 | 0.053144484 | 0.038787548 | HDAC9/PCK2/PIK3R3/SRC/STAR/TBC1D4/TRIB3/XBP1 | 8 |
| GO:0051701 | biological process involved in interaction with host | 8/261 | 203/18723 | 0.007770993 | 0.053144484 | 0.038787548 | CD86/FCN1/GAS6/GSN/ITGA5/ITGB7/SRC/TRIM14 | 8 |
| GO:0060541 | respiratory system development | 8/261 | 203/18723 | 0.007770993 | 0.053144484 | 0.038787548 | ASS1/CHI3L1/GPC3/MSC/NOG/RXFP1/TGFBR2/VEGFA | 8 |
| GO:0002524 | hypersensitivity | 2/261 | 10/18723 | 0.008091578 | 0.054559405 | 0.03982023 | C3/FCGR2B | 2 |
| GO:0002765 | immune response-inhibiting signal transduction | 2/261 | 10/18723 | 0.008091578 | 0.054559405 | 0.03982023 | LILRB2/LILRB4 | 2 |
| GO:0002887 | negative regulation of myeloid leukocyte mediated immunity | 2/261 | 10/18723 | 0.008091578 | 0.054559405 | 0.03982023 | FCGR2B/HMOX1 | 2 |
| GO:0070099 | regulation of chemokine-mediated signaling pathway | 2/261 | 10/18723 | 0.008091578 | 0.054559405 | 0.03982023 | CCL5/TREM2 | 2 |
| GO:0071372 | cellular response to follicle-stimulating hormone stimulus | 2/261 | 10/18723 | 0.008091578 | 0.054559405 | 0.03982023 | GCLM/STAR | 2 |
| GO:0097278 | complement-dependent cytotoxicity | 2/261 | 10/18723 | 0.008091578 | 0.054559405 | 0.03982023 | C3/CFH | 2 |
| GO:1903980 | positive regulation of microglial cell activation | 2/261 | 10/18723 | 0.008091578 | 0.054559405 | 0.03982023 | LRRK2/TREM2 | 2 |
| GO:0014910 | regulation of smooth muscle cell migration | 5/261 | 89/18723 | 0.008131074 | 0.054559405 | 0.03982023 | CCL5/P2RY6/SRC/TERT/TLR4 | 5 |
| GO:0046330 | positive regulation of JNK cascade | 5/261 | 89/18723 | 0.008131074 | 0.054559405 | 0.03982023 | FCGR2B/NCF1/SH3RF3/TLR4/TNFRSF19 | 5 |
| GO:0060760 | positive regulation of response to cytokine stimulus | 4/261 | 57/18723 | 0.008174943 | 0.054559405 | 0.03982023 | CD300LF/GAS6/TLR4/TREM2 | 4 |
| GO:0061756 | leukocyte adhesion to vascular endothelial cell | 4/261 | 57/18723 | 0.008174943 | 0.054559405 | 0.03982023 | ITGA4/ITGB2/ITGB7/PTAFR | 4 |
| GO:0071621 | granulocyte chemotaxis | 6/261 | 125/18723 | 0.008185508 | 0.054559405 | 0.03982023 | CCL5/CSF1R/CXCL10/ITGB2/JAML/THBS4 | 6 |
| GO:0043032 | positive regulation of macrophage activation | 3/261 | 30/18723 | 0.008233992 | 0.054559405 | 0.03982023 | LRRK2/TLR4/TREM2 | 3 |
| GO:0048147 | negative regulation of fibroblast proliferation | 3/261 | 30/18723 | 0.008233992 | 0.054559405 | 0.03982023 | FTH1/MYC/NLRC3 | 3 |
| GO:0048799 | animal organ maturation | 3/261 | 30/18723 | 0.008233992 | 0.054559405 | 0.03982023 | FGFR3/RFLNB/XYLT1 | 3 |
| GO:0050858 | negative regulation of antigen receptor-mediated signaling pathway | 3/261 | 30/18723 | 0.008233992 | 0.054559405 | 0.03982023 | FCGR2B/LILRB4/PTPN22 | 3 |
| GO:0060603 | mammary gland duct morphogenesis | 3/261 | 30/18723 | 0.008233992 | 0.054559405 | 0.03982023 | CSF1R/PTCH1/SRC | 3 |
| GO:0007568 | aging | 11/261 | 339/18723 | 0.008255507 | 0.054559405 | 0.03982023 | ASS1/BCL2A1/GCLM/IGFBP2/JUN/LMNA/LRRK2/PAX5/TERT/TGFBR2/TIMP1 | 11 |
| GO:0048545 | response to steroid hormone | 11/261 | 339/18723 | 0.008255507 | 0.054559405 | 0.03982023 | ASS1/CA2/CYBB/IGFBP2/PCK2/PTAFR/SRC/STAR/TGFBR2/ZFP36L2/ZNF366 | 11 |
| GO:0048732 | gland development | 13/261 | 436/18723 | 0.008445538 | 0.055640032 | 0.040608926 | ASS1/CSF1R/HMOX1/NOG/PCK2/PRLR/PTCH1/RXFP1/SLC29A1/SRC/TGFBR2/VEGFA/XBP1 | 13 |
| GO:0048863 | stem cell differentiation | 8/261 | 206/18723 | 0.00845335 | 0.055640032 | 0.040608926 | A2M/KIT/RBM24/SEMA4A/SEMA4C/SEMA6A/SEMA6B/ZFP36L2 | 8 |
| GO:0051604 | protein maturation | 10/261 | 294/18723 | 0.008463722 | 0.055640032 | 0.040608926 | CHAC1/F3/FURIN/GSN/LRRK2/PTCH1/SOX4/SRC/SRGN/TSPAN32 | 10 |
| GO:0034644 | cellular response to UV | 5/261 | 90/18723 | 0.008513889 | 0.055871464 | 0.040777837 | MFAP4/MMP2/MMP9/MYC/TIMP1 | 5 |
| GO:0043112 | receptor metabolic process | 7/261 | 166/18723 | 0.008788211 | 0.057469669 | 0.041944288 | ANXA2/CD36/DNM1/FURIN/ITGB2/LILRB4/VEGFA | 7 |
| GO:0060538 | skeletal muscle organ development | 7/261 | 166/18723 | 0.008788211 | 0.057469669 | 0.041944288 | ASS1/CYP26B1/EGR1/HDAC9/MSC/PAX5/RBM24 | 7 |
| GO:0006911 | phagocytosis, engulfment | 6/261 | 127/18723 | 0.008817437 | 0.057559983 | 0.042010204 | C3/CD36/FCGR2B/GSN/ITGB2/TREM2 | 6 |
| GO:0001657 | ureteric bud development | 5/261 | 91/18723 | 0.008909006 | 0.0579551 | 0.04229858 | GPC3/MYC/NOG/PTCH1/VEGFA | 5 |
| GO:0072080 | nephron tubule development | 5/261 | 91/18723 | 0.008909006 | 0.0579551 | 0.04229858 | GPC3/MYC/NOG/PTCH1/VEGFA | 5 |
| GO:0002063 | chondrocyte development | 3/261 | 31/18723 | 0.009023697 | 0.058294955 | 0.042546624 | ECM1/RFLNB/TGFBR2 | 3 |
| GO:0031063 | regulation of histone deacetylation | 3/261 | 31/18723 | 0.009023697 | 0.058294955 | 0.042546624 | CAMK2D/LRRK2/VEGFA | 3 |
| GO:0045987 | positive regulation of smooth muscle contraction | 3/261 | 31/18723 | 0.009023697 | 0.058294955 | 0.042546624 | CTTN/KIT/PTAFR | 3 |
| GO:0090183 | regulation of kidney development | 3/261 | 31/18723 | 0.009023697 | 0.058294955 | 0.042546624 | MYC/NOG/VEGFA | 3 |
| GO:0007254 | JNK cascade | 7/261 | 167/18723 | 0.009066931 | 0.058372274 | 0.042603055 | FCGR2B/LRRK2/NCF1/PTPN22/SH3RF3/TLR4/TNFRSF19 | 7 |
| GO:1903034 | regulation of response to wounding | 7/261 | 167/18723 | 0.009066931 | 0.058372274 | 0.042603055 | ANXA2/CD109/CD36/F3/FAP/GRN/XBP1 | 7 |
| GO:0043281 | regulation of cysteine-type endopeptidase activity involved in apoptotic process | 8/261 | 209/18723 | 0.009179669 | 0.058996361 | 0.043058545 | F3/GAS6/GSN/MMP9/MYC/NLRP12/SRC/VEGFA | 8 |
| GO:0043551 | regulation of phosphatidylinositol 3-kinase activity | 4/261 | 59/18723 | 0.009218238 | 0.059142442 | 0.043165163 | FGFR3/KIT/PIK3R3/SRC | 4 |
| GO:0048525 | negative regulation of viral process | 5/261 | 92/18723 | 0.00931661 | 0.059388886 | 0.04334503 | CCL5/FCN1/GSN/SLPI/TRIM14 | 5 |
| GO:0072163 | mesonephric epithelium development | 5/261 | 92/18723 | 0.00931661 | 0.059388886 | 0.04334503 | GPC3/MYC/NOG/PTCH1/VEGFA | 5 |
| GO:0072164 | mesonephric tubule development | 5/261 | 92/18723 | 0.00931661 | 0.059388886 | 0.04334503 | GPC3/MYC/NOG/PTCH1/VEGFA | 5 |
| GO:0002706 | regulation of lymphocyte mediated immunity | 7/261 | 168/18723 | 0.00935208 | 0.059388886 | 0.04334503 | C3/CD1D/FCER2/FCGR2B/LILRB4/SLC15A4/TREM2 | 7 |
| GO:0002822 | regulation of adaptive immune response based on somatic recombination of immune receptors built from immunoglobulin superfamily domains | 7/261 | 168/18723 | 0.00935208 | 0.059388886 | 0.04334503 | C3/CD1D/FCER2/FCGR2B/LILRB4/SLC15A4/TREM2 | 7 |
| GO:0099173 | postsynapse organization | 7/261 | 168/18723 | 0.00935208 | 0.059388886 | 0.04334503 | COLQ/CTTN/FCGR2B/LILRB2/LRRK2/SRGN/TREM2 | 7 |
| GO:0045637 | regulation of myeloid cell differentiation | 8/261 | 210/18723 | 0.009431832 | 0.059793653 | 0.04364045 | CA2/GPR68/JUN/LILRB4/MYC/TLR4/TREM2/TYROBP | 8 |
| GO:0006022 | aminoglycan metabolic process | 6/261 | 129/18723 | 0.009483572 | 0.059939896 | 0.043747186 | CHI3L1/FUCA1/GNS/NDST1/SPOCK2/XYLT1 | 6 |
| GO:0031214 | biomineral tissue development | 7/261 | 169/18723 | 0.009643742 | 0.059939896 | 0.043747186 | ECM1/FAM20C/FGFR3/GAS6/GPC3/RFLNB/SRGN | 7 |
| GO:0050807 | regulation of synapse organization | 8/261 | 211/18723 | 0.009689125 | 0.059939896 | 0.043747186 | COLQ/FCGR2B/LILRB2/LRRK2/SEMA4A/SEPTIN11/SRGN/TREM2 | 8 |
| GO:0007589 | body fluid secretion | 5/261 | 93/18723 | 0.009736886 | 0.059939896 | 0.043747186 | ANXA2/PRLR/SLC29A1/VEGFA/XBP1 | 5 |
| GO:0051591 | response to cAMP | 5/261 | 93/18723 | 0.009736886 | 0.059939896 | 0.043747186 | ASS1/JUN/PTAFR/RAP1B/STAR | 5 |
| GO:0016055 | Wnt signaling pathway | 13/261 | 444/18723 | 0.009738869 | 0.059939896 | 0.043747186 | ATP6V1C2/CMAHP/DIXDC1/EGR1/GPC3/GRK6/IGFBP2/LRRK2/NOG/SHISA2/SOX4/SRC/TERT | 13 |
| GO:0010986 | positive regulation of lipoprotein particle clearance | 2/261 | 11/18723 | 0.009799318 | 0.059939896 | 0.043747186 | ANXA2/TREM2 | 2 |
| GO:0032957 | inositol trisphosphate metabolic process | 2/261 | 11/18723 | 0.009799318 | 0.059939896 | 0.043747186 | P2RY6/PTAFR | 2 |
| GO:0033625 | positive regulation of integrin activation | 2/261 | 11/18723 | 0.009799318 | 0.059939896 | 0.043747186 | RAP1B/SRC | 2 |
| GO:0034163 | regulation of toll-like receptor 9 signaling pathway | 2/261 | 11/18723 | 0.009799318 | 0.059939896 | 0.043747186 | PTPN22/SLC15A4 | 2 |
| GO:0034350 | regulation of glial cell apoptotic process | 2/261 | 11/18723 | 0.009799318 | 0.059939896 | 0.043747186 | GAS6/TREM2 | 2 |
| GO:0035457 | cellular response to interferon-alpha | 2/261 | 11/18723 | 0.009799318 | 0.059939896 | 0.043747186 | GAS6/STAR | 2 |
| GO:0042989 | sequestering of actin monomers | 2/261 | 11/18723 | 0.009799318 | 0.059939896 | 0.043747186 | GSN/TWF1 | 2 |
| GO:0048103 | somatic stem cell division | 2/261 | 11/18723 | 0.009799318 | 0.059939896 | 0.043747186 | KIT/ZFP36L2 | 2 |
| GO:0071679 | commissural neuron axon guidance | 2/261 | 11/18723 | 0.009799318 | 0.059939896 | 0.043747186 | PTCH1/VEGFA | 2 |
| GO:0098883 | synapse pruning | 2/261 | 11/18723 | 0.009799318 | 0.059939896 | 0.043747186 | C3/TREM2 | 2 |
| GO:1900227 | positive regulation of NLRP3 inflammasome complex assembly | 2/261 | 11/18723 | 0.009799318 | 0.059939896 | 0.043747186 | CD36/TLR4 | 2 |
| GO:1903238 | positive regulation of leukocyte tethering or rolling | 2/261 | 11/18723 | 0.009799318 | 0.059939896 | 0.043747186 | ITGA4/PTAFR | 2 |
| GO:1903423 | positive regulation of synaptic vesicle recycling | 2/261 | 11/18723 | 0.009799318 | 0.059939896 | 0.043747186 | DNM1/LRRK2 | 2 |
| GO:2000343 | positive regulation of chemokine (C-X-C motif) ligand 2 production | 2/261 | 11/18723 | 0.009799318 | 0.059939896 | 0.043747186 | MCOLN2/TLR4 | 2 |
| GO:2000644 | regulation of receptor catabolic process | 2/261 | 11/18723 | 0.009799318 | 0.059939896 | 0.043747186 | ANXA2/FURIN | 2 |
| GO:0006956 | complement activation | 6/261 | 130/18723 | 0.009829767 | 0.059939896 | 0.043747186 | A2M/C3/CFH/FCN1/MFAP4/TREM2 | 6 |
| GO:0015918 | sterol transport | 6/261 | 130/18723 | 0.009829767 | 0.059939896 | 0.043747186 | ANXA2/CD36/FURIN/PTCH1/STAR/TREM2 | 6 |
| GO:0006026 | aminoglycan catabolic process | 3/261 | 32/18723 | 0.009856212 | 0.059939896 | 0.043747186 | CHI3L1/FUCA1/GNS | 3 |
| GO:0060323 | head morphogenesis | 3/261 | 32/18723 | 0.009856212 | 0.059939896 | 0.043747186 | MMP2/NOG/TIPARP | 3 |
| GO:0043523 | regulation of neuron apoptotic process | 8/261 | 212/18723 | 0.00995161 | 0.060421647 | 0.044098792 | GCLM/GRN/HMOX1/JUN/STAR/TERT/TREM2/TYROBP | 8 |
| GO:0198738 | cell-cell signaling by wnt | 13/261 | 446/18723 | 0.010085244 | 0.061133609 | 0.044618418 | ATP6V1C2/CMAHP/DIXDC1/EGR1/GPC3/GRK6/IGFBP2/LRRK2/NOG/SHISA2/SOX4/SRC/TERT | 13 |
| GO:0002042 | cell migration involved in sprouting angiogenesis | 5/261 | 94/18723 | 0.010170017 | 0.061339209 | 0.044768475 | EPHB4/HDAC9/HMOX1/PIK3R3/VEGFA | 5 |
| GO:0048704 | embryonic skeletal system morphogenesis | 5/261 | 94/18723 | 0.010170017 | 0.061339209 | 0.044768475 | HOXC4/NDST1/NOG/PAX5/TGFBR2 | 5 |
| GO:0061326 | renal tubule development | 5/261 | 94/18723 | 0.010170017 | 0.061339209 | 0.044768475 | GPC3/MYC/NOG/PTCH1/VEGFA | 5 |
| GO:0019827 | stem cell population maintenance | 6/261 | 131/18723 | 0.010184871 | 0.061339209 | 0.044768475 | KIT/MYC/NOG/PADI4/SOX4/ZFP36L2 | 6 |
| GO:0110148 | biomineralization | 7/261 | 171/18723 | 0.010246937 | 0.06161363 | 0.044968762 | ECM1/FAM20C/FGFR3/GAS6/GPC3/RFLNB/SRGN | 7 |
| GO:0007162 | negative regulation of cell adhesion | 10/261 | 303/18723 | 0.010323447 | 0.06180298 | 0.045106959 | ADAMDEC1/ASS1/CD86/FCGR2B/LILRB2/LILRB4/PTPN22/SEMA6A/SRC/VEGFA | 10 |
| GO:0043954 | cellular component maintenance | 4/261 | 61/18723 | 0.010344634 | 0.06180298 | 0.045106959 | CSF1R/CTTN/FCGR2B/TREM2 | 4 |
| GO:0071384 | cellular response to corticosteroid stimulus | 4/261 | 61/18723 | 0.010344634 | 0.06180298 | 0.045106959 | ASS1/PCK2/STAR/ZFP36L2 | 4 |
| GO:2000401 | regulation of lymphocyte migration | 4/261 | 61/18723 | 0.010344634 | 0.06180298 | 0.045106959 | CCL5/CXCL10/ECM1/ITGA4 | 4 |
| GO:0051651 | maintenance of location in cell | 8/261 | 214/18723 | 0.0104924 | 0.062585656 | 0.045678196 | CAMK2D/CXCL10/FTH1/GSN/MCOLN2/P2RY6/SRGN/TWF1 | 8 |
| GO:0002221 | pattern recognition receptor signaling pathway | 7/261 | 172/18723 | 0.010558637 | 0.062780174 | 0.045820165 | CD300LF/CD36/FCN1/PTPN22/SLC15A4/TLR4/TREM2 | 7 |
| GO:1990138 | neuron projection extension | 7/261 | 172/18723 | 0.010558637 | 0.062780174 | 0.045820165 | CTTN/ITGA4/SEMA4A/SEMA4C/SEMA6A/SEMA6B/VEGFA | 7 |
| GO:0010737 | protein kinase A signaling | 3/261 | 33/18723 | 0.010731971 | 0.06330676 | 0.046204494 | AKAP7/GAL/LRRK2 | 3 |
| GO:0036336 | dendritic cell migration | 3/261 | 33/18723 | 0.010731971 | 0.06330676 | 0.046204494 | CCL5/GAS6/NLRP12 | 3 |
| GO:0043552 | positive regulation of phosphatidylinositol 3-kinase activity | 3/261 | 33/18723 | 0.010731971 | 0.06330676 | 0.046204494 | FGFR3/KIT/SRC | 3 |
| GO:0045920 | negative regulation of exocytosis | 3/261 | 33/18723 | 0.010731971 | 0.06330676 | 0.046204494 | FCGR2B/HMOX1/RAP1B | 3 |
| GO:0050901 | leukocyte tethering or rolling | 3/261 | 33/18723 | 0.010731971 | 0.06330676 | 0.046204494 | ITGA4/ITGB7/PTAFR | 3 |
| GO:0046328 | regulation of JNK cascade | 6/261 | 133/18723 | 0.010922281 | 0.064127118 | 0.046803233 | FCGR2B/NCF1/PTPN22/SH3RF3/TLR4/TNFRSF19 | 6 |
| GO:0032757 | positive regulation of interleukin-8 production | 4/261 | 62/18723 | 0.010939736 | 0.064127118 | 0.046803233 | CHI3L1/F3/FCN1/TLR4 | 4 |
| GO:0045576 | mast cell activation | 4/261 | 62/18723 | 0.010939736 | 0.064127118 | 0.046803233 | CD300LF/CNR2/HMOX1/KIT | 4 |
| GO:0046173 | polyol biosynthetic process | 4/261 | 62/18723 | 0.010939736 | 0.064127118 | 0.046803233 | ASAH1/P2RY6/PCK2/PTAFR | 4 |
| GO:0001823 | mesonephros development | 5/261 | 96/18723 | 0.011075556 | 0.064821513 | 0.047310038 | GPC3/MYC/NOG/PTCH1/VEGFA | 5 |
| GO:0001659 | temperature homeostasis | 7/261 | 174/18723 | 0.01120265 | 0.065462747 | 0.047778043 | ADAMTS5/CD36/EGR1/PRLR/SCD/TLR4/VEGFA | 7 |
| GO:0060537 | muscle tissue development | 12/261 | 403/18723 | 0.011285692 | 0.065622831 | 0.04789488 | CYP26B1/EGR1/HDAC9/LMNA/MSC/NOG/PAX5/PTCH1/RBM24/TGFBR2/TIPARP/VEGFA | 12 |
| GO:0001508 | action potential | 6/261 | 134/18723 | 0.011304818 | 0.065622831 | 0.04789488 | CAMK2D/CD36/CNR2/GNA11/GPR35/GPR88 | 6 |
| GO:0061041 | regulation of wound healing | 6/261 | 134/18723 | 0.011304818 | 0.065622831 | 0.04789488 | ANXA2/CD109/CD36/F3/FAP/XBP1 | 6 |
| GO:0098727 | maintenance of cell number | 6/261 | 134/18723 | 0.011304818 | 0.065622831 | 0.04789488 | KIT/MYC/NOG/PADI4/SOX4/ZFP36L2 | 6 |
| GO:0014909 | smooth muscle cell migration | 5/261 | 97/18723 | 0.011548315 | 0.065622831 | 0.04789488 | CCL5/P2RY6/SRC/TERT/TLR4 | 5 |
| GO:0070059 | intrinsic apoptotic signaling pathway in response to endoplasmic reticulum stress | 4/261 | 63/18723 | 0.011556485 | 0.065622831 | 0.04789488 | CHAC1/LRRK2/TRIB3/XBP1 | 4 |
| GO:0050803 | regulation of synapse structure or activity | 8/261 | 218/18723 | 0.011638966 | 0.065622831 | 0.04789488 | COLQ/FCGR2B/LILRB2/LRRK2/SEMA4A/SEPTIN11/SRGN/TREM2 | 8 |
| GO:0016242 | negative regulation of macroautophagy | 3/261 | 34/18723 | 0.011651361 | 0.065622831 | 0.04789488 | HMOX1/LRRK2/QSOX1 | 3 |
| GO:0035590 | purinergic nucleotide receptor signaling pathway | 3/261 | 34/18723 | 0.011651361 | 0.065622831 | 0.04789488 | ACP3/P2RY6/PTAFR | 3 |
| GO:0050869 | negative regulation of B cell activation | 3/261 | 34/18723 | 0.011651361 | 0.065622831 | 0.04789488 | FCGR2B/SAMSN1/TYROBP | 3 |
| GO:0050931 | pigment cell differentiation | 3/261 | 34/18723 | 0.011651361 | 0.065622831 | 0.04789488 | GNA11/HPS4/KIT | 3 |
| GO:0006068 | ethanol catabolic process | 2/261 | 12/18723 | 0.011651831 | 0.065622831 | 0.04789488 | ALDH2/ALDH3B1 | 2 |
| GO:0006563 | L-serine metabolic process | 2/261 | 12/18723 | 0.011651831 | 0.065622831 | 0.04789488 | PHGDH/PSAT1 | 2 |
| GO:0014831 | gastro-intestinal system smooth muscle contraction | 2/261 | 12/18723 | 0.011651831 | 0.065622831 | 0.04789488 | KIT/PTAFR | 2 |
| GO:0032490 | detection of molecule of bacterial origin | 2/261 | 12/18723 | 0.011651831 | 0.065622831 | 0.04789488 | TLR4/TREM2 | 2 |
| GO:0032908 | regulation of transforming growth factor beta1 production | 2/261 | 12/18723 | 0.011651831 | 0.065622831 | 0.04789488 | FURIN/TYROBP | 2 |
| GO:0034154 | toll-like receptor 7 signaling pathway | 2/261 | 12/18723 | 0.011651831 | 0.065622831 | 0.04789488 | PTPN22/SLC15A4 | 2 |
| GO:0043301 | negative regulation of leukocyte degranulation | 2/261 | 12/18723 | 0.011651831 | 0.065622831 | 0.04789488 | FCGR2B/HMOX1 | 2 |
| GO:0045117 | azole transmembrane transport | 2/261 | 12/18723 | 0.011651831 | 0.065622831 | 0.04789488 | SLC15A4/SLC38A5 | 2 |
| GO:0045602 | negative regulation of endothelial cell differentiation | 2/261 | 12/18723 | 0.011651831 | 0.065622831 | 0.04789488 | VEGFA/ZEB1 | 2 |
| GO:0060907 | positive regulation of macrophage cytokine production | 2/261 | 12/18723 | 0.011651831 | 0.065622831 | 0.04789488 | CD36/TLR4 | 2 |
| GO:0061517 | macrophage proliferation | 2/261 | 12/18723 | 0.011651831 | 0.065622831 | 0.04789488 | CSF1R/TREM2 | 2 |
| GO:0071635 | negative regulation of transforming growth factor beta production | 2/261 | 12/18723 | 0.011651831 | 0.065622831 | 0.04789488 | FURIN/TYROBP | 2 |
| GO:2001204 | regulation of osteoclast development | 2/261 | 12/18723 | 0.011651831 | 0.065622831 | 0.04789488 | GPR68/TYROBP | 2 |
| GO:0001101 | response to acid chemical | 6/261 | 135/18723 | 0.011696732 | 0.065776503 | 0.048007038 | ASS1/CYBB/GCLM/MMP2/VEGFA/XBP1 | 6 |
| GO:0003018 | vascular process in circulatory system | 9/261 | 263/18723 | 0.011762271 | 0.066045596 | 0.048203436 | AZU1/CD36/GCLM/SLC29A1/SLC38A5/SLC44A1/SLC7A5/SRC/VEGFA | 9 |
| GO:0010634 | positive regulation of epithelial cell migration | 7/261 | 176/18723 | 0.011874695 | 0.066576742 | 0.048591094 | GRN/HDAC9/HMOX1/MMP9/SRC/TGFBR2/VEGFA | 7 |
| GO:0099024 | plasma membrane invagination | 6/261 | 136/18723 | 0.012098136 | 0.067727798 | 0.049431193 | C3/CD36/FCGR2B/GSN/ITGB2/TREM2 | 6 |
| GO:0050771 | negative regulation of axonogenesis | 4/261 | 64/18723 | 0.012195153 | 0.068168713 | 0.049752995 | SEMA4A/SEMA4C/SEMA6A/SEMA6B | 4 |
| GO:0010977 | negative regulation of neuron projection development | 6/261 | 137/18723 | 0.012509144 | 0.069679576 | 0.0508557 | LRRK2/NR2F1/SEMA4A/SEMA4C/SEMA6A/SEMA6B | 6 |
| GO:0031341 | regulation of cell killing | 5/261 | 99/18723 | 0.012534673 | 0.069679576 | 0.0508557 | CD1D/CFH/FCER2/FCGR2B/TYROBP | 5 |
| GO:0018149 | peptide cross-linking | 3/261 | 35/18723 | 0.012614728 | 0.069679576 | 0.0508557 | CSTA/SPOCK2/TGM5 | 3 |
| GO:0032350 | regulation of hormone metabolic process | 3/261 | 35/18723 | 0.012614728 | 0.069679576 | 0.0508557 | EGR1/GAL/STC2 | 3 |
| GO:0032814 | regulation of natural killer cell activation | 3/261 | 35/18723 | 0.012614728 | 0.069679576 | 0.0508557 | GAS6/PTPN22/TYROBP | 3 |
| GO:0034405 | response to fluid shear stress | 3/261 | 35/18723 | 0.012614728 | 0.069679576 | 0.0508557 | ASS1/CA2/SRC | 3 |
| GO:0090050 | positive regulation of cell migration involved in sprouting angiogenesis | 3/261 | 35/18723 | 0.012614728 | 0.069679576 | 0.0508557 | HDAC9/HMOX1/VEGFA | 3 |
| GO:2000403 | positive regulation of lymphocyte migration | 3/261 | 35/18723 | 0.012614728 | 0.069679576 | 0.0508557 | CCL5/CXCL10/ITGA4 | 3 |
| GO:0002720 | positive regulation of cytokine production involved in immune response | 4/261 | 65/18723 | 0.012856001 | 0.070594569 | 0.051523508 | CD36/KIT/SLC7A5/TLR4 | 4 |
| GO:0045453 | bone resorption | 4/261 | 65/18723 | 0.012856001 | 0.070594569 | 0.051523508 | CA2/CSF1R/SRC/TPP1 | 4 |
| GO:0070613 | regulation of protein processing | 4/261 | 65/18723 | 0.012856001 | 0.070594569 | 0.051523508 | CHAC1/GSN/LRRK2/SRC | 4 |
| GO:0071677 | positive regulation of mononuclear cell migration | 4/261 | 65/18723 | 0.012856001 | 0.070594569 | 0.051523508 | CCL5/CXCL10/GAS6/ITGA4 | 4 |
| GO:0001936 | regulation of endothelial cell proliferation | 7/261 | 179/18723 | 0.012936739 | 0.070933603 | 0.051770952 | ECM1/EGFL7/F3/HMOX1/ITGA4/THBS4/VEGFA | 7 |
| GO:0046394 | carboxylic acid biosynthetic process | 10/261 | 314/18723 | 0.013001569 | 0.071184545 | 0.051954102 | ALOX5AP/ASS1/FADS2/PHGDH/PSAT1/PTGS1/SCD/STAR/TRIB3/XBP1 | 10 |
| GO:0019218 | regulation of steroid metabolic process | 5/261 | 100/18723 | 0.013048607 | 0.071337477 | 0.05206572 | ASAH1/EGR1/GAL/KIT/STAR | 5 |
| GO:0002768 | immune response-regulating cell surface receptor signaling pathway | 10/261 | 315/18723 | 0.013268706 | 0.072166586 | 0.052670846 | FCGR2B/FCN1/FYB1/KIT/LILRB2/LILRB4/PRAM1/PTPN22/SRC/TYROBP | 10 |
| GO:0031589 | cell-substrate adhesion | 11/261 | 363/18723 | 0.013274266 | 0.072166586 | 0.052670846 | CD36/CTTN/GAS6/ITGA4/ITGA5/ITGA6/ITGB2/ITGB7/SPOCK2/SRC/VEGFA | 11 |
| GO:0051098 | regulation of binding | 11/261 | 363/18723 | 0.013274266 | 0.072166586 | 0.052670846 | ANXA2/HMOX1/ITGA4/JUN/LRRK2/MMP9/NOG/SLPI/SRC/TERT/TRIB3 | 11 |
| GO:0043409 | negative regulation of MAPK cascade | 7/261 | 180/18723 | 0.013305469 | 0.072166586 | 0.052670846 | LILRB4/MYC/NLRP12/PTPN22/SEMA6A/TLR4/TREM2 | 7 |
| GO:0030193 | regulation of blood coagulation | 4/261 | 66/18723 | 0.013539282 | 0.072166586 | 0.052670846 | ANXA2/CD36/F3/FAP | 4 |
| GO:0050922 | negative regulation of chemotaxis | 4/261 | 66/18723 | 0.013539282 | 0.072166586 | 0.052670846 | SEMA4A/SEMA4C/SEMA6A/SEMA6B | 4 |
| GO:2000573 | positive regulation of DNA biosynthetic process | 4/261 | 66/18723 | 0.013539282 | 0.072166586 | 0.052670846 | MYC/PKIB/RFC3/SRC | 4 |
| GO:0016053 | organic acid biosynthetic process | 10/261 | 316/18723 | 0.01353995 | 0.072166586 | 0.052670846 | ALOX5AP/ASS1/FADS2/PHGDH/PSAT1/PTGS1/SCD/STAR/TRIB3/XBP1 | 10 |
| GO:0006476 | protein deacetylation | 5/261 | 101/18723 | 0.013576595 | 0.072166586 | 0.052670846 | CAMK2D/FRY/HDAC9/LRRK2/VEGFA | 5 |
| GO:0046466 | membrane lipid catabolic process | 3/261 | 36/18723 | 0.013622374 | 0.072166586 | 0.052670846 | ASAH1/FUCA1/GM2A | 3 |
| GO:0051385 | response to mineralocorticoid | 3/261 | 36/18723 | 0.013622374 | 0.072166586 | 0.052670846 | CYBB/SRC/STAR | 3 |
| GO:0090322 | regulation of superoxide metabolic process | 3/261 | 36/18723 | 0.013622374 | 0.072166586 | 0.052670846 | CD36/ITGB2/TYROBP | 3 |
| GO:0001768 | establishment of T cell polarity | 2/261 | 13/18723 | 0.01364478 | 0.072166586 | 0.052670846 | CYP26B1/GSN | 2 |
| GO:0002551 | mast cell chemotaxis | 2/261 | 13/18723 | 0.01364478 | 0.072166586 | 0.052670846 | KIT/VEGFA | 2 |
| GO:0007567 | parturition | 2/261 | 13/18723 | 0.01364478 | 0.072166586 | 0.052670846 | PTAFR/RXFP1 | 2 |
| GO:0031272 | regulation of pseudopodium assembly | 2/261 | 13/18723 | 0.01364478 | 0.072166586 | 0.052670846 | CDC42EP1/KIT | 2 |
| GO:0031274 | positive regulation of pseudopodium assembly | 2/261 | 13/18723 | 0.01364478 | 0.072166586 | 0.052670846 | CDC42EP1/KIT | 2 |
| GO:0032905 | transforming growth factor beta1 production | 2/261 | 13/18723 | 0.01364478 | 0.072166586 | 0.052670846 | FURIN/TYROBP | 2 |
| GO:0044090 | positive regulation of vacuole organization | 2/261 | 13/18723 | 0.01364478 | 0.072166586 | 0.052670846 | ANXA2/GRN | 2 |
| GO:0061418 | regulation of transcription from RNA polymerase II promoter in response to hypoxia | 2/261 | 13/18723 | 0.01364478 | 0.072166586 | 0.052670846 | EGR1/VEGFA | 2 |
| GO:0071801 | regulation of podosome assembly | 2/261 | 13/18723 | 0.01364478 | 0.072166586 | 0.052670846 | GSN/SRC | 2 |
| GO:1903054 | negative regulation of extracellular matrix organization | 2/261 | 13/18723 | 0.01364478 | 0.072166586 | 0.052670846 | CST3/FAP | 2 |
| GO:2001198 | regulation of dendritic cell differentiation | 2/261 | 13/18723 | 0.01364478 | 0.072166586 | 0.052670846 | FCGR2B/LILRB2 | 2 |
| GO:1905952 | regulation of lipid localization | 7/261 | 181/18723 | 0.013681688 | 0.072259439 | 0.052738614 | ANXA2/C3/CD36/FURIN/GAL/PTCH1/TREM2 | 7 |
| GO:0050768 | negative regulation of neurogenesis | 6/261 | 140/18723 | 0.013800906 | 0.072759483 | 0.053103572 | NOG/SEMA4A/SEMA4C/SEMA6A/SEMA6B/TREM2 | 6 |
| GO:0010506 | regulation of autophagy | 10/261 | 317/18723 | 0.013815338 | 0.072759483 | 0.053103572 | ATP6V0A1/ATP6V1C2/CTTN/HMOX1/LRRK2/PTPN22/QSOX1/TREM2/TRIB3/TRIM14 | 10 |
| GO:0016485 | protein processing | 8/261 | 225/18723 | 0.013864064 | 0.072913263 | 0.053215808 | CHAC1/F3/FURIN/GSN/LRRK2/PTCH1/SRC/SRGN | 8 |
| GO:0016032 | viral process | 12/261 | 415/18723 | 0.013952041 | 0.073272747 | 0.053478178 | CCL5/CD86/FCN1/FURIN/GAS6/GSN/ITGA5/ITGB7/JUN/SLPI/TOP2A/TRIM14 | 12 |
| GO:0061138 | morphogenesis of a branching epithelium | 7/261 | 182/18723 | 0.014065476 | 0.07376473 | 0.053837252 | GPC3/MYC/NOG/PTCH1/SRC/TGFBR2/VEGFA | 7 |
| GO:1902106 | negative regulation of leukocyte differentiation | 5/261 | 102/18723 | 0.014118798 | 0.073940519 | 0.053965552 | FCGR2B/GPR68/LILRB4/MYC/TLR4 | 5 |
| GO:0035914 | skeletal muscle cell differentiation | 4/261 | 67/18723 | 0.014245241 | 0.074115429 | 0.054093211 | CYP26B1/EGR1/PAX5/RBM24 | 4 |
| GO:0046635 | positive regulation of alpha-beta T cell activation | 4/261 | 67/18723 | 0.014245241 | 0.074115429 | 0.054093211 | CD86/LILRB4/PTPN22/TGFBR2 | 4 |
| GO:0030183 | B cell differentiation | 6/261 | 141/18723 | 0.01425144 | 0.074115429 | 0.054093211 | FCGR2B/HDAC9/ITGA4/KIT/XBP1/ZFP36L2 | 6 |
| GO:0031333 | negative regulation of protein-containing complex assembly | 6/261 | 141/18723 | 0.01425144 | 0.074115429 | 0.054093211 | GSN/NLRC3/SRC/TMOD2/TREM2/TWF1 | 6 |
| GO:1903038 | negative regulation of leukocyte cell-cell adhesion | 6/261 | 141/18723 | 0.01425144 | 0.074115429 | 0.054093211 | ASS1/CD86/FCGR2B/LILRB2/LILRB4/PTPN22 | 6 |
| GO:0007411 | axon guidance | 8/261 | 227/18723 | 0.014553247 | 0.075579728 | 0.05516193 | EPHB4/NOG/PTCH1/SEMA4A/SEMA4C/SEMA6A/SEMA6B/VEGFA | 8 |
| GO:0009416 | response to light stimulus | 10/261 | 320/18723 | 0.014666744 | 0.075583231 | 0.055164487 | B4GALT2/DTL/GNA11/GPR88/KIT/MFAP4/MMP2/MMP9/MYC/TIMP1 | 10 |
| GO:0060416 | response to growth hormone | 3/261 | 37/18723 | 0.014674564 | 0.075583231 | 0.055164487 | ASS1/STAR/TRIM16 | 3 |
| GO:0090218 | positive regulation of lipid kinase activity | 3/261 | 37/18723 | 0.014674564 | 0.075583231 | 0.055164487 | FGFR3/KIT/SRC | 3 |
| GO:1900016 | negative regulation of cytokine production involved in inflammatory response | 3/261 | 37/18723 | 0.014674564 | 0.075583231 | 0.055164487 | LILRB4/NLRC3/TREM2 | 3 |
| GO:0030593 | neutrophil chemotaxis | 5/261 | 103/18723 | 0.014675373 | 0.075583231 | 0.055164487 | CCL5/CXCL10/ITGB2/JAML/THBS4 | 5 |
| GO:0045639 | positive regulation of myeloid cell differentiation | 5/261 | 103/18723 | 0.014675373 | 0.075583231 | 0.055164487 | CA2/GPR68/JUN/TREM2/TYROBP | 5 |
| GO:0072006 | nephron development | 6/261 | 142/18723 | 0.014712128 | 0.075668162 | 0.055226474 | EGR1/GPC3/MYC/NOG/PTCH1/VEGFA | 6 |
| GO:0097485 | neuron projection guidance | 8/261 | 228/18723 | 0.014907041 | 0.076565187 | 0.055881169 | EPHB4/NOG/PTCH1/SEMA4A/SEMA4C/SEMA6A/SEMA6B/VEGFA | 8 |
| GO:0042531 | positive regulation of tyrosine phosphorylation of STAT protein | 4/261 | 68/18723 | 0.01497411 | 0.076698664 | 0.055978587 | CCL5/CSF1R/FGFR3/KIT | 4 |
| GO:1900046 | regulation of hemostasis | 4/261 | 68/18723 | 0.01497411 | 0.076698664 | 0.055978587 | ANXA2/CD36/F3/FAP | 4 |
| GO:0002040 | sprouting angiogenesis | 7/261 | 185/18723 | 0.015263031 | 0.077601875 | 0.056637796 | EPHB4/HDAC9/HMOX1/ITGA5/PIK3R3/SEMA6A/VEGFA | 7 |
| GO:0071356 | cellular response to tumor necrosis factor | 8/261 | 229/18723 | 0.015267048 | 0.077601875 | 0.056637796 | ASAH1/ASS1/CCL5/CHI3L1/GAS6/PCK2/TNFRSF19/ZFP36L2 | 8 |
| GO:0071695 | anatomical structure maturation | 8/261 | 229/18723 | 0.015267048 | 0.077601875 | 0.056637796 | C3/FGFR3/GAL/LRRK2/MMP2/RFLNB/VEGFA/XYLT1 | 8 |
| GO:0045055 | regulated exocytosis | 8/261 | 230/18723 | 0.015633327 | 0.077601875 | 0.056637796 | FCGR2B/HMOX1/ITGB2/KIT/LRRK2/PRAM1/PTAFR/RAP1B | 8 |
| GO:0045862 | positive regulation of proteolysis | 11/261 | 372/18723 | 0.015658667 | 0.077601875 | 0.056637796 | F3/FURIN/GRN/GSN/LRRK2/MYC/NLRP12/RNF144A/SRC/TREM2/TRIB3 | 11 |
| GO:0010324 | membrane invagination | 6/261 | 144/18723 | 0.015664389 | 0.077601875 | 0.056637796 | C3/CD36/FCGR2B/GSN/ITGB2/TREM2 | 6 |
| GO:0014065 | phosphatidylinositol 3-kinase signaling | 6/261 | 144/18723 | 0.015664389 | 0.077601875 | 0.056637796 | CCL5/KIT/NCF1/NLRC3/SRC/TREM2 | 6 |
| GO:0106106 | cold-induced thermogenesis | 6/261 | 144/18723 | 0.015664389 | 0.077601875 | 0.056637796 | ADAMTS5/CD36/PRLR/SCD/TLR4/VEGFA | 6 |
| GO:0120161 | regulation of cold-induced thermogenesis | 6/261 | 144/18723 | 0.015664389 | 0.077601875 | 0.056637796 | ADAMTS5/CD36/PRLR/SCD/TLR4/VEGFA | 6 |
| GO:0008217 | regulation of blood pressure | 7/261 | 186/18723 | 0.01567787 | 0.077601875 | 0.056637796 | ADM2/GAS6/HMOX1/NCALD/PTAFR/PTGS1/SUCNR1 | 7 |
| GO:0071478 | cellular response to radiation | 7/261 | 186/18723 | 0.01567787 | 0.077601875 | 0.056637796 | EGR1/GPR88/MFAP4/MMP2/MMP9/MYC/TIMP1 | 7 |
| GO:0050805 | negative regulation of synaptic transmission | 4/261 | 69/18723 | 0.015726114 | 0.077601875 | 0.056637796 | CNR2/LILRB2/LRRK2/RAP1B | 4 |
| GO:1901224 | positive regulation of NIK/NF-kappaB signaling | 4/261 | 69/18723 | 0.015726114 | 0.077601875 | 0.056637796 | CD86/NLRP12/TLR4/TREM2 | 4 |
| GO:0030279 | negative regulation of ossification | 3/261 | 38/18723 | 0.015771521 | 0.077601875 | 0.056637796 | ECM1/RFLNB/SRGN | 3 |
| GO:0032373 | positive regulation of sterol transport | 3/261 | 38/18723 | 0.015771521 | 0.077601875 | 0.056637796 | ANXA2/PTCH1/TREM2 | 3 |
| GO:0032376 | positive regulation of cholesterol transport | 3/261 | 38/18723 | 0.015771521 | 0.077601875 | 0.056637796 | ANXA2/PTCH1/TREM2 | 3 |
| GO:0032717 | negative regulation of interleukin-8 production | 3/261 | 38/18723 | 0.015771521 | 0.077601875 | 0.056637796 | ANXA4/BPI/PTPN22 | 3 |
| GO:0048246 | macrophage chemotaxis | 3/261 | 38/18723 | 0.015771521 | 0.077601875 | 0.056637796 | AZU1/CCL5/CSF1R | 3 |
| GO:0071398 | cellular response to fatty acid | 3/261 | 38/18723 | 0.015771521 | 0.077601875 | 0.056637796 | ASS1/PTAFR/SRC | 3 |
| GO:0001767 | establishment of lymphocyte polarity | 2/261 | 14/18723 | 0.015773922 | 0.077601875 | 0.056637796 | CYP26B1/GSN | 2 |
| GO:0002756 | MyD88-independent toll-like receptor signaling pathway | 2/261 | 14/18723 | 0.015773922 | 0.077601875 | 0.056637796 | CD300LF/TLR4 | 2 |
| GO:0002864 | regulation of acute inflammatory response to antigenic stimulus | 2/261 | 14/18723 | 0.015773922 | 0.077601875 | 0.056637796 | C3/FCGR2B | 2 |
| GO:0035589 | G protein-coupled purinergic nucleotide receptor signaling pathway | 2/261 | 14/18723 | 0.015773922 | 0.077601875 | 0.056637796 | P2RY6/PTAFR | 2 |
| GO:0035672 | oligopeptide transmembrane transport | 2/261 | 14/18723 | 0.015773922 | 0.077601875 | 0.056637796 | CA2/SLC15A4 | 2 |
| GO:0038166 | angiotensin-activated signaling pathway | 2/261 | 14/18723 | 0.015773922 | 0.077601875 | 0.056637796 | CA2/SRC | 2 |
| GO:0043922 | negative regulation by host of viral transcription | 2/261 | 14/18723 | 0.015773922 | 0.077601875 | 0.056637796 | CCL5/JUN | 2 |
| GO:0050930 | induction of positive chemotaxis | 2/261 | 14/18723 | 0.015773922 | 0.077601875 | 0.056637796 | AZU1/VEGFA | 2 |
| GO:0060732 | positive regulation of inositol phosphate biosynthetic process | 2/261 | 14/18723 | 0.015773922 | 0.077601875 | 0.056637796 | P2RY6/PTAFR | 2 |
| GO:0097531 | mast cell migration | 2/261 | 14/18723 | 0.015773922 | 0.077601875 | 0.056637796 | KIT/VEGFA | 2 |
| GO:1901722 | regulation of cell proliferation involved in kidney development | 2/261 | 14/18723 | 0.015773922 | 0.077601875 | 0.056637796 | EGR1/MYC | 2 |
| GO:0008544 | epidermis development | 10/261 | 324/18723 | 0.015862231 | 0.077933646 | 0.056879939 | ASAH1/CD109/CSTA/CYP26B1/GAL/PTCH1/STS/TGM5/TNFRSF19/TRIM16 | 10 |
| GO:0045732 | positive regulation of protein catabolic process | 8/261 | 231/18723 | 0.016005935 | 0.078536346 | 0.057319821 | DTL/FURIN/GPC3/LRRK2/RNF144A/TIPARP/TREM2/TRIB3 | 8 |
| GO:0051961 | negative regulation of nervous system development | 6/261 | 145/18723 | 0.016156173 | 0.079169487 | 0.057781919 | NOG/SEMA4A/SEMA4C/SEMA6A/SEMA6B/TREM2 | 6 |
| GO:1903707 | negative regulation of hemopoiesis | 5/261 | 106/18723 | 0.01643286 | 0.080419787 | 0.058694452 | FCGR2B/GPR68/LILRB4/MYC/TLR4 | 5 |
| GO:0043542 | endothelial cell migration | 9/261 | 279/18723 | 0.016698167 | 0.081611199 | 0.059564005 | EPHB4/FAP/GRN/HDAC9/HMOX1/PIK3R3/S100P/SEMA4A/VEGFA | 9 |
| GO:0007517 | muscle organ development | 10/261 | 327/18723 | 0.016805347 | 0.082027668 | 0.059867965 | ASS1/CXCL10/CYP26B1/EGR1/HDAC9/MSC/NOG/PAX5/RBM24/XBP1 | 10 |
| GO:0002701 | negative regulation of production of molecular mediator of immune response | 3/261 | 39/18723 | 0.016913432 | 0.08244746 | 0.060174351 | FCGR2B/HMOX1/LILRB4 | 3 |
| GO:0031532 | actin cytoskeleton reorganization | 5/261 | 107/18723 | 0.017048435 | 0.082997206 | 0.060575583 | CSF1R/CTTN/GSN/IQGAP2/KIT | 5 |
| GO:0048588 | developmental cell growth | 8/261 | 234/18723 | 0.017162308 | 0.083334274 | 0.060821592 | CTTN/ITGA4/SEMA4A/SEMA4C/SEMA6A/SEMA6B/TGFBR2/VEGFA | 8 |
| GO:0060560 | developmental growth involved in morphogenesis | 8/261 | 234/18723 | 0.017162308 | 0.083334274 | 0.060821592 | CTTN/ITGA4/SEMA4A/SEMA4C/SEMA6A/SEMA6B/TGFBR2/VEGFA | 8 |
| GO:0043550 | regulation of lipid kinase activity | 4/261 | 71/18723 | 0.017300381 | 0.083678267 | 0.061072656 | FGFR3/KIT/PIK3R3/SRC | 4 |
| GO:0045824 | negative regulation of innate immune response | 4/261 | 71/18723 | 0.017300381 | 0.083678267 | 0.061072656 | A2M/GRN/NLRC3/SAMHD1 | 4 |
| GO:0050818 | regulation of coagulation | 4/261 | 71/18723 | 0.017300381 | 0.083678267 | 0.061072656 | ANXA2/CD36/F3/FAP | 4 |
| GO:0051216 | cartilage development | 7/261 | 190/18723 | 0.01741752 | 0.084135859 | 0.06140663 | CHI3L1/ECM1/FGFR3/NOG/RFLNB/TGFBR2/TIMP1 | 7 |
| GO:2000116 | regulation of cysteine-type endopeptidase activity | 8/261 | 235/18723 | 0.017560805 | 0.084718405 | 0.061831802 | F3/GAS6/GSN/MMP9/MYC/NLRP12/SRC/VEGFA | 8 |
| GO:0014074 | response to purine-containing compound | 6/261 | 148/18723 | 0.017695362 | 0.085037941 | 0.062065015 | ASS1/JUN/P2RY6/PTAFR/RAP1B/STAR | 6 |
| GO:0051384 | response to glucocorticoid | 6/261 | 148/18723 | 0.017695362 | 0.085037941 | 0.062065015 | ASS1/IGFBP2/PCK2/PTAFR/STAR/ZFP36L2 | 6 |
| GO:1903900 | regulation of viral life cycle | 6/261 | 148/18723 | 0.017695362 | 0.085037941 | 0.062065015 | CCL5/FCN1/GSN/SLPI/TOP2A/TRIM14 | 6 |
| GO:0002730 | regulation of dendritic cell cytokine production | 2/261 | 15/18723 | 0.018035101 | 0.085138558 | 0.062138451 | KIT/TLR4 | 2 |
| GO:0016446 | somatic hypermutation of immunoglobulin genes | 2/261 | 15/18723 | 0.018035101 | 0.085138558 | 0.062138451 | SAMHD1/UNG | 2 |
| GO:0031269 | pseudopodium assembly | 2/261 | 15/18723 | 0.018035101 | 0.085138558 | 0.062138451 | CDC42EP1/KIT | 2 |
| GO:0032354 | response to follicle-stimulating hormone | 2/261 | 15/18723 | 0.018035101 | 0.085138558 | 0.062138451 | GCLM/STAR | 2 |
| GO:0034310 | primary alcohol catabolic process | 2/261 | 15/18723 | 0.018035101 | 0.085138558 | 0.062138451 | ALDH2/ALDH3B1 | 2 |
| GO:0042976 | activation of Janus kinase activity | 2/261 | 15/18723 | 0.018035101 | 0.085138558 | 0.062138451 | CCL5/PRLR | 2 |
| GO:0048712 | negative regulation of astrocyte differentiation | 2/261 | 15/18723 | 0.018035101 | 0.085138558 | 0.062138451 | NOG/TREM2 | 2 |
| GO:1904251 | regulation of bile acid metabolic process | 2/261 | 15/18723 | 0.018035101 | 0.085138558 | 0.062138451 | KIT/STAR | 2 |
| GO:0002714 | positive regulation of B cell mediated immunity | 3/261 | 40/18723 | 0.018100447 | 0.085138558 | 0.062138451 | C3/FCER2/TREM2 | 3 |
| GO:0002891 | positive regulation of immunoglobulin mediated immune response | 3/261 | 40/18723 | 0.018100447 | 0.085138558 | 0.062138451 | C3/FCER2/TREM2 | 3 |
| GO:0009069 | serine family amino acid metabolic process | 3/261 | 40/18723 | 0.018100447 | 0.085138558 | 0.062138451 | GCLM/PHGDH/PSAT1 | 3 |
| GO:0045429 | positive regulation of nitric oxide biosynthetic process | 3/261 | 40/18723 | 0.018100447 | 0.085138558 | 0.062138451 | ASS1/CD36/TLR4 | 3 |
| GO:0060412 | ventricular septum morphogenesis | 3/261 | 40/18723 | 0.018100447 | 0.085138558 | 0.062138451 | NOG/SOX4/TGFBR2 | 3 |
| GO:1904994 | regulation of leukocyte adhesion to vascular endothelial cell | 3/261 | 40/18723 | 0.018100447 | 0.085138558 | 0.062138451 | ITGA4/ITGB2/PTAFR | 3 |
| GO:0002534 | cytokine production involved in inflammatory response | 4/261 | 72/18723 | 0.018123045 | 0.085138558 | 0.062138451 | LILRB4/NLRC3/TLR4/TREM2 | 4 |
| GO:1900015 | regulation of cytokine production involved in inflammatory response | 4/261 | 72/18723 | 0.018123045 | 0.085138558 | 0.062138451 | LILRB4/NLRC3/TLR4/TREM2 | 4 |
| GO:1903036 | positive regulation of response to wounding | 4/261 | 72/18723 | 0.018123045 | 0.085138558 | 0.062138451 | CD36/F3/GRN/XBP1 | 4 |
| GO:0019216 | regulation of lipid metabolic process | 10/261 | 331/18723 | 0.018126715 | 0.085138558 | 0.062138451 | ASAH1/C3/EGR1/FGFR3/GAL/KIT/SRC/STAR/TREM2/TRIB3 | 10 |
| GO:0009411 | response to UV | 6/261 | 149/18723 | 0.018230038 | 0.085516282 | 0.062414133 | DTL/MFAP4/MMP2/MMP9/MYC/TIMP1 | 6 |
| GO:0072009 | nephron epithelium development | 5/261 | 109/18723 | 0.018325061 | 0.085854174 | 0.062660744 | GPC3/MYC/NOG/PTCH1/VEGFA | 5 |
| GO:0001959 | regulation of cytokine-mediated signaling pathway | 6/261 | 150/18723 | 0.018775683 | 0.08785514 | 0.06412115 | CCL5/CD300LF/ECM1/GAS6/SAMHD1/TREM2 | 6 |
| GO:0001935 | endothelial cell proliferation | 7/261 | 193/18723 | 0.01880864 | 0.087899202 | 0.064153309 | ECM1/EGFL7/F3/HMOX1/ITGA4/THBS4/VEGFA | 7 |
| GO:0002637 | regulation of immunoglobulin production | 4/261 | 73/18723 | 0.018969648 | 0.088215396 | 0.064384084 | CD86/FCGR2B/SLC15A4/XBP1 | 4 |
| GO:0035924 | cellular response to vascular endothelial growth factor stimulus | 4/261 | 73/18723 | 0.018969648 | 0.088215396 | 0.064384084 | MT1G/SEMA6A/TSPAN12/VEGFA | 4 |
| GO:0002698 | negative regulation of immune effector process | 5/261 | 110/18723 | 0.018986384 | 0.088215396 | 0.064384084 | A2M/FCGR2B/GRN/HMOX1/LILRB4 | 5 |
| GO:1904659 | glucose transmembrane transport | 5/261 | 110/18723 | 0.018986384 | 0.088215396 | 0.064384084 | C3/GPC3/SORT1/TERT/TRIB3 | 5 |
| GO:0032535 | regulation of cellular component size | 11/261 | 383/18723 | 0.018994424 | 0.088215396 | 0.064384084 | CDC42EP1/CTTN/GSN/IQGAP2/SEMA4A/SEMA4C/SEMA6A/SEMA6B/TMOD2/TWF1/VEGFA | 11 |
| GO:0042391 | regulation of membrane potential | 12/261 | 434/18723 | 0.019124508 | 0.088709208 | 0.064744493 | CAMK2D/CD36/CNR2/GCLM/GNA11/GPR35/GPR88/JUN/LRRK2/SLC29A1/SRC/TREM2 | 12 |
| GO:0051693 | actin filament capping | 3/261 | 41/18723 | 0.019332682 | 0.089341875 | 0.065206245 | GSN/TMOD2/TWF1 | 3 |
| GO:1904407 | positive regulation of nitric oxide metabolic process | 3/261 | 41/18723 | 0.019332682 | 0.089341875 | 0.065206245 | ASS1/CD36/TLR4 | 3 |
| GO:1905521 | regulation of macrophage migration | 3/261 | 41/18723 | 0.019332682 | 0.089341875 | 0.065206245 | CCL5/CSF1R/TREM2 | 3 |
| GO:0097193 | intrinsic apoptotic signaling pathway | 9/261 | 288/18723 | 0.020065357 | 0.091773955 | 0.066981301 | BCL2A1/CHAC1/HMOX1/LRRK2/MMP9/SRC/TREM2/TRIB3/XBP1 | 9 |
| GO:0022408 | negative regulation of cell-cell adhesion | 7/261 | 196/18723 | 0.020275971 | 0.091773955 | 0.066981301 | ASS1/CD86/FCGR2B/LILRB2/LILRB4/PTPN22/VEGFA | 7 |
| GO:0002526 | acute inflammatory response | 5/261 | 112/18723 | 0.020355707 | 0.091773955 | 0.066981301 | ALOX5AP/ASS1/C3/F3/FCGR2B | 5 |
| GO:0032609 | interferon-gamma production | 5/261 | 112/18723 | 0.020355707 | 0.091773955 | 0.066981301 | GAS6/LILRB4/PTPN22/SLC7A5/TLR4 | 5 |
| GO:0032649 | regulation of interferon-gamma production | 5/261 | 112/18723 | 0.020355707 | 0.091773955 | 0.066981301 | GAS6/LILRB4/PTPN22/SLC7A5/TLR4 | 5 |
| GO:0035601 | protein deacylation | 5/261 | 112/18723 | 0.020355707 | 0.091773955 | 0.066981301 | CAMK2D/FRY/HDAC9/LRRK2/VEGFA | 5 |
| GO:0043406 | positive regulation of MAP kinase activity | 5/261 | 112/18723 | 0.020355707 | 0.091773955 | 0.066981301 | KIT/LRRK2/SRC/TLR4/VEGFA | 5 |
| GO:0002371 | dendritic cell cytokine production | 2/261 | 16/18723 | 0.020424252 | 0.091773955 | 0.066981301 | KIT/TLR4 | 2 |
| GO:0002566 | somatic diversification of immune receptors via somatic mutation | 2/261 | 16/18723 | 0.020424252 | 0.091773955 | 0.066981301 | SAMHD1/UNG | 2 |
| GO:0002921 | negative regulation of humoral immune response | 2/261 | 16/18723 | 0.020424252 | 0.091773955 | 0.066981301 | A2M/FCGR2B | 2 |
| GO:0003198 | epithelial to mesenchymal transition involved in endocardial cushion formation | 2/261 | 16/18723 | 0.020424252 | 0.091773955 | 0.066981301 | NOG/TGFBR2 | 2 |
| GO:0006750 | glutathione biosynthetic process | 2/261 | 16/18723 | 0.020424252 | 0.091773955 | 0.066981301 | CHAC1/GCLM | 2 |
| GO:0006857 | oligopeptide transport | 2/261 | 16/18723 | 0.020424252 | 0.091773955 | 0.066981301 | CA2/SLC15A4 | 2 |
| GO:0010819 | regulation of T cell chemotaxis | 2/261 | 16/18723 | 0.020424252 | 0.091773955 | 0.066981301 | CCL5/CXCL10 | 2 |
| GO:0010919 | regulation of inositol phosphate biosynthetic process | 2/261 | 16/18723 | 0.020424252 | 0.091773955 | 0.066981301 | P2RY6/PTAFR | 2 |
| GO:0019377 | glycolipid catabolic process | 2/261 | 16/18723 | 0.020424252 | 0.091773955 | 0.066981301 | FUCA1/GM2A | 2 |
| GO:0031268 | pseudopodium organization | 2/261 | 16/18723 | 0.020424252 | 0.091773955 | 0.066981301 | CDC42EP1/KIT | 2 |
| GO:0033631 | cell-cell adhesion mediated by integrin | 2/261 | 16/18723 | 0.020424252 | 0.091773955 | 0.066981301 | CCL5/ITGA4 | 2 |
| GO:0035751 | regulation of lysosomal lumen pH | 2/261 | 16/18723 | 0.020424252 | 0.091773955 | 0.066981301 | GRN/LRRK2 | 2 |
| GO:0048385 | regulation of retinoic acid receptor signaling pathway | 2/261 | 16/18723 | 0.020424252 | 0.091773955 | 0.066981301 | CYP26B1/TRIM16 | 2 |
| GO:1901163 | regulation of trophoblast cell migration | 2/261 | 16/18723 | 0.020424252 | 0.091773955 | 0.066981301 | TIMP1/VEGFA | 2 |
| GO:1903236 | regulation of leukocyte tethering or rolling | 2/261 | 16/18723 | 0.020424252 | 0.091773955 | 0.066981301 | ITGA4/PTAFR | 2 |
| GO:2001267 | regulation of cysteine-type endopeptidase activity involved in apoptotic signaling pathway | 2/261 | 16/18723 | 0.020424252 | 0.091773955 | 0.066981301 | GSN/MMP9 | 2 |
| GO:0032689 | negative regulation of interferon-gamma production | 3/261 | 42/18723 | 0.020610219 | 0.091945707 | 0.067106655 | GAS6/LILRB4/TLR4 | 3 |
| GO:0033003 | regulation of mast cell activation | 3/261 | 42/18723 | 0.020610219 | 0.091945707 | 0.067106655 | CD300LF/CNR2/HMOX1 | 3 |
| GO:0046596 | regulation of viral entry into host cell | 3/261 | 42/18723 | 0.020610219 | 0.091945707 | 0.067106655 | FCN1/GSN/TRIM14 | 3 |
| GO:0051452 | intracellular pH reduction | 3/261 | 42/18723 | 0.020610219 | 0.091945707 | 0.067106655 | ATP6V0A1/CA2/GRN | 3 |
| GO:1902895 | positive regulation of pri-miRNA transcription by RNA polymerase II | 3/261 | 42/18723 | 0.020610219 | 0.091945707 | 0.067106655 | EGR1/JUN/TERT | 3 |
| GO:1904037 | positive regulation of epithelial cell apoptotic process | 3/261 | 42/18723 | 0.020610219 | 0.091945707 | 0.067106655 | GSN/ITGA4/SORT1 | 3 |
| GO:0051453 | regulation of intracellular pH | 4/261 | 75/18723 | 0.020735372 | 0.092393653 | 0.067433588 | ATP6V0A1/CA2/GRN/LRRK2 | 4 |
| GO:0009896 | positive regulation of catabolic process | 13/261 | 492/18723 | 0.021043374 | 0.093549164 | 0.068276939 | DTL/FURIN/GPC3/HMOX1/LRRK2/RBM24/RNF144A/SAMD4A/TIPARP/TREM2/TRIB3/TRIM14/ZFP36L2 | 13 |
| GO:0031623 | receptor internalization | 5/261 | 113/18723 | 0.021063961 | 0.093549164 | 0.068276939 | CD36/DNM1/ITGB2/LILRB4/VEGFA | 5 |
| GO:0030856 | regulation of epithelial cell differentiation | 6/261 | 154/18723 | 0.021069857 | 0.093549164 | 0.068276939 | CD109/MMP9/PTCH1/TRIM16/VEGFA/ZEB1 | 6 |
| GO:0060485 | mesenchyme development | 9/261 | 291/18723 | 0.021289769 | 0.094335387 | 0.068850765 | MYC/NOG/RBM24/RFLNB/SEMA4A/SEMA4C/SEMA6A/SEMA6B/TGFBR2 | 9 |
| GO:0050864 | regulation of B cell activation | 7/261 | 198/18723 | 0.021297464 | 0.094335387 | 0.068850765 | FCGR2B/SAMSN1/SLC15A4/TLR4/TYROBP/XBP1/ZFP36L2 | 7 |
| GO:0031100 | animal organ regeneration | 4/261 | 76/18723 | 0.02165482 | 0.09576609 | 0.069894965 | GAS6/HMOX1/PTCH1/TGFBR2 | 4 |
| GO:0007519 | skeletal muscle tissue development | 6/261 | 155/18723 | 0.021671759 | 0.09576609 | 0.069894965 | CYP26B1/EGR1/HDAC9/MSC/PAX5/RBM24 | 6 |
| GO:0042176 | regulation of protein catabolic process | 11/261 | 391/18723 | 0.021733006 | 0.095923222 | 0.070009648 | ANXA2/CST3/DTL/FURIN/GPC3/LRRK2/RNF144A/TIMP1/TIPARP/TREM2/TRIB3 | 11 |
| GO:0008645 | hexose transmembrane transport | 5/261 | 114/18723 | 0.021788105 | 0.096052875 | 0.070104275 | C3/GPC3/SORT1/TERT/TRIB3 | 5 |
| GO:0014002 | astrocyte development | 3/261 | 43/18723 | 0.021933106 | 0.096350845 | 0.070321749 | GRN/TLR4/TREM2 | 3 |
| GO:0031952 | regulation of protein autophosphorylation | 3/261 | 43/18723 | 0.021933106 | 0.096350845 | 0.070321749 | NLRP12/SRC/VEGFA | 3 |
| GO:0034142 | toll-like receptor 4 signaling pathway | 3/261 | 43/18723 | 0.021933106 | 0.096350845 | 0.070321749 | PTPN22/TLR4/TREM2 | 3 |
| GO:0051402 | neuron apoptotic process | 8/261 | 246/18723 | 0.022392895 | 0.098255076 | 0.071711553 | GCLM/GRN/HMOX1/JUN/STAR/TERT/TREM2/TYROBP | 8 |
| GO:0010827 | regulation of glucose transmembrane transport | 4/261 | 77/18723 | 0.022598861 | 0.098446252 | 0.071851083 | C3/GPC3/TERT/TRIB3 | 4 |
| GO:0010975 | regulation of neuron projection development | 12/261 | 445/18723 | 0.022710752 | 0.098446252 | 0.071851083 | GRN/ITGA6/LRRK2/NR2F1/SDC2/SEMA4A/SEMA4C/SEMA6A/SEMA6B/SERPINI1/TWF1/VEGFA | 12 |
| GO:1990845 | adaptive thermogenesis | 6/261 | 157/18723 | 0.022910213 | 0.098446252 | 0.071851083 | ADAMTS5/CD36/PRLR/SCD/TLR4/VEGFA | 6 |
| GO:0010715 | regulation of extracellular matrix disassembly | 2/261 | 17/18723 | 0.022937397 | 0.098446252 | 0.071851083 | CST3/FAP | 2 |
| GO:0010831 | positive regulation of myotube differentiation | 2/261 | 17/18723 | 0.022937397 | 0.098446252 | 0.071851083 | CYP26B1/RBM24 | 2 |
| GO:0010934 | macrophage cytokine production | 2/261 | 17/18723 | 0.022937397 | 0.098446252 | 0.071851083 | CD36/TLR4 | 2 |
| GO:0010935 | regulation of macrophage cytokine production | 2/261 | 17/18723 | 0.022937397 | 0.098446252 | 0.071851083 | CD36/TLR4 | 2 |
| GO:0033623 | regulation of integrin activation | 2/261 | 17/18723 | 0.022937397 | 0.098446252 | 0.071851083 | RAP1B/SRC | 2 |
| GO:0034349 | glial cell apoptotic process | 2/261 | 17/18723 | 0.022937397 | 0.098446252 | 0.071851083 | GAS6/TREM2 | 2 |
| GO:0045591 | positive regulation of regulatory T cell differentiation | 2/261 | 17/18723 | 0.022937397 | 0.098446252 | 0.071851083 | LILRB2/LILRB4 | 2 |
| GO:0055119 | relaxation of cardiac muscle | 2/261 | 17/18723 | 0.022937397 | 0.098446252 | 0.071851083 | CAMK2D/GSN | 2 |
| GO:0060263 | regulation of respiratory burst | 2/261 | 17/18723 | 0.022937397 | 0.098446252 | 0.071851083 | GRN/NCF1 | 2 |
| GO:0061158 | 3'-UTR-mediated mRNA destabilization | 2/261 | 17/18723 | 0.022937397 | 0.098446252 | 0.071851083 | RBM24/ZFP36L2 | 2 |
| GO:0061450 | trophoblast cell migration | 2/261 | 17/18723 | 0.022937397 | 0.098446252 | 0.071851083 | TIMP1/VEGFA | 2 |
| GO:0070293 | renal absorption | 2/261 | 17/18723 | 0.022937397 | 0.098446252 | 0.071851083 | GAS6/GSN | 2 |
| GO:0071371 | cellular response to gonadotropin stimulus | 2/261 | 17/18723 | 0.022937397 | 0.098446252 | 0.071851083 | GCLM/STAR | 2 |
| GO:0090136 | epithelial cell-cell adhesion | 2/261 | 17/18723 | 0.022937397 | 0.098446252 | 0.071851083 | KIT/THBS4 | 2 |
| GO:0097067 | cellular response to thyroid hormone stimulus | 2/261 | 17/18723 | 0.022937397 | 0.098446252 | 0.071851083 | GCLM/KIT | 2 |
| GO:2000811 | negative regulation of anoikis | 2/261 | 17/18723 | 0.022937397 | 0.098446252 | 0.071851083 | ITGA5/SRC | 2 |
| GO:0015749 | monosaccharide transmembrane transport | 5/261 | 116/18723 | 0.023284533 | 0.09875969 | 0.072079846 | C3/GPC3/SORT1/TERT/TRIB3 | 5 |
| GO:0018107 | peptidyl-threonine phosphorylation | 5/261 | 116/18723 | 0.023284533 | 0.09875969 | 0.072079846 | AZU1/CAMK2D/CHI3L1/LRRK2/TGFBR2 | 5 |
| GO:0021782 | glial cell development | 5/261 | 116/18723 | 0.023284533 | 0.09875969 | 0.072079846 | GRN/PHGDH/SOX4/TLR4/TREM2 | 5 |
| GO:0043200 | response to amino acid | 5/261 | 116/18723 | 0.023284533 | 0.09875969 | 0.072079846 | ASS1/CYBB/GCLM/MMP2/XBP1 | 5 |
| GO:0098732 | macromolecule deacylation | 5/261 | 116/18723 | 0.023284533 | 0.09875969 | 0.072079846 | CAMK2D/FRY/HDAC9/LRRK2/VEGFA | 5 |
| GO:0045124 | regulation of bone resorption | 3/261 | 44/18723 | 0.023301362 | 0.09875969 | 0.072079846 | CA2/CSF1R/SRC | 3 |
| GO:0046427 | positive regulation of receptor signaling pathway via JAK-STAT | 3/261 | 44/18723 | 0.023301362 | 0.09875969 | 0.072079846 | CCL5/KIT/PRLR | 3 |
| GO:0048066 | developmental pigmentation | 3/261 | 44/18723 | 0.023301362 | 0.09875969 | 0.072079846 | GNA11/HPS4/KIT | 3 |
| GO:0050999 | regulation of nitric-oxide synthase activity | 3/261 | 44/18723 | 0.023301362 | 0.09875969 | 0.072079846 | CNR2/FCER2/TERT | 3 |
| GO:0060324 | face development | 3/261 | 44/18723 | 0.023301362 | 0.09875969 | 0.072079846 | MMP2/NOG/TIPARP | 3 |
| GO:0062208 | positive regulation of pattern recognition receptor signaling pathway | 3/261 | 44/18723 | 0.023301362 | 0.09875969 | 0.072079846 | PTPN22/SLC15A4/TLR4 | 3 |
| GO:0017157 | regulation of exocytosis | 7/261 | 202/18723 | 0.023446746 | 0.099263209 | 0.072447339 | FCGR2B/HMOX1/ITGB2/LRRK2/PRAM1/PTAFR/RAP1B | 7 |
| GO:0032371 | regulation of sterol transport | 4/261 | 78/18723 | 0.023567634 | 0.099436773 | 0.072574016 | ANXA2/FURIN/PTCH1/TREM2 | 4 |
| GO:0032374 | regulation of cholesterol transport | 4/261 | 78/18723 | 0.023567634 | 0.099436773 | 0.072574016 | ANXA2/FURIN/PTCH1/TREM2 | 4 |
| GO:0043154 | negative regulation of cysteine-type endopeptidase activity involved in apoptotic process | 4/261 | 78/18723 | 0.023567634 | 0.099436773 | 0.072574016 | GAS6/MMP9/SRC/VEGFA | 4 |
| GO:0031647 | regulation of protein stability | 9/261 | 298/18723 | 0.024354558 | 0.102640992 | 0.074912617 | GRN/GSN/HPS4/LMNA/LRRK2/SOX4/SRC/TERT/TYROBP | 9 |
| GO:0003197 | endocardial cushion development | 3/261 | 45/18723 | 0.024714975 | 0.103459323 | 0.075509877 | NOG/RBM24/TGFBR2 | 3 |
| GO:0030835 | negative regulation of actin filament depolymerization | 3/261 | 45/18723 | 0.024714975 | 0.103459323 | 0.075509877 | GSN/TMOD2/TWF1 | 3 |
| GO:0031670 | cellular response to nutrient | 3/261 | 45/18723 | 0.024714975 | 0.103459323 | 0.075509877 | GAS6/HMOX1/XBP1 | 3 |
| GO:0044088 | regulation of vacuole organization | 3/261 | 45/18723 | 0.024714975 | 0.103459323 | 0.075509877 | ANXA2/GRN/LRRK2 | 3 |
| GO:0045933 | positive regulation of muscle contraction | 3/261 | 45/18723 | 0.024714975 | 0.103459323 | 0.075509877 | CTTN/KIT/PTAFR | 3 |
| GO:0085029 | extracellular matrix assembly | 3/261 | 45/18723 | 0.024714975 | 0.103459323 | 0.075509877 | GAS6/MFAP4/QSOX1 | 3 |
| GO:0030203 | glycosaminoglycan metabolic process | 5/261 | 118/18723 | 0.024845891 | 0.10377467 | 0.075740034 | FUCA1/GNS/NDST1/SPOCK2/XYLT1 | 5 |
| GO:0034219 | carbohydrate transmembrane transport | 5/261 | 118/18723 | 0.024845891 | 0.10377467 | 0.075740034 | C3/GPC3/SORT1/TERT/TRIB3 | 5 |
| GO:0042742 | defense response to bacterium | 10/261 | 350/18723 | 0.025467736 | 0.105039358 | 0.076663067 | ADAMTS5/AZU1/BPI/CD36/GRN/LYZ/RNASE3/SLPI/TLR4/TREM2 | 10 |
| GO:0002223 | stimulatory C-type lectin receptor signaling pathway | 2/261 | 18/18723 | 0.025570642 | 0.105039358 | 0.076663067 | SRC/TYROBP | 2 |
| GO:0006957 | complement activation, alternative pathway | 2/261 | 18/18723 | 0.025570642 | 0.105039358 | 0.076663067 | C3/CFH | 2 |
| GO:0019184 | nonribosomal peptide biosynthetic process | 2/261 | 18/18723 | 0.025570642 | 0.105039358 | 0.076663067 | CHAC1/GCLM | 2 |
| GO:0030889 | negative regulation of B cell proliferation | 2/261 | 18/18723 | 0.025570642 | 0.105039358 | 0.076663067 | FCGR2B/TYROBP | 2 |
| GO:0034162 | toll-like receptor 9 signaling pathway | 2/261 | 18/18723 | 0.025570642 | 0.105039358 | 0.076663067 | PTPN22/SLC15A4 | 2 |
| GO:0051782 | negative regulation of cell division | 2/261 | 18/18723 | 0.025570642 | 0.105039358 | 0.076663067 | MYC/PTCH1 | 2 |
| GO:0060192 | negative regulation of lipase activity | 2/261 | 18/18723 | 0.025570642 | 0.105039358 | 0.076663067 | HDAC9/SORT1 | 2 |
| GO:0071800 | podosome assembly | 2/261 | 18/18723 | 0.025570642 | 0.105039358 | 0.076663067 | GSN/SRC | 2 |
| GO:0090190 | positive regulation of branching involved in ureteric bud morphogenesis | 2/261 | 18/18723 | 0.025570642 | 0.105039358 | 0.076663067 | NOG/VEGFA | 2 |
| GO:0150078 | positive regulation of neuroinflammatory response | 2/261 | 18/18723 | 0.025570642 | 0.105039358 | 0.076663067 | LRRK2/TREM2 | 2 |
| GO:0150079 | negative regulation of neuroinflammatory response | 2/261 | 18/18723 | 0.025570642 | 0.105039358 | 0.076663067 | GRN/TREM2 | 2 |
| GO:1900242 | regulation of synaptic vesicle endocytosis | 2/261 | 18/18723 | 0.025570642 | 0.105039358 | 0.076663067 | DNM1/LRRK2 | 2 |
| GO:1990840 | response to lectin | 2/261 | 18/18723 | 0.025570642 | 0.105039358 | 0.076663067 | SRC/TYROBP | 2 |
| GO:1990858 | cellular response to lectin | 2/261 | 18/18723 | 0.025570642 | 0.105039358 | 0.076663067 | SRC/TYROBP | 2 |
| GO:0030282 | bone mineralization | 5/261 | 119/18723 | 0.025651183 | 0.105254414 | 0.076820026 | ECM1/FGFR3/GPC3/RFLNB/SRGN | 5 |
| GO:0034612 | response to tumor necrosis factor | 8/261 | 253/18723 | 0.025917316 | 0.106229702 | 0.077531841 | ASAH1/ASS1/CCL5/CHI3L1/GAS6/PCK2/TNFRSF19/ZFP36L2 | 8 |
| GO:0010828 | positive regulation of glucose transmembrane transport | 3/261 | 46/18723 | 0.026173902 | 0.106812405 | 0.077957127 | C3/GPC3/TERT | 3 |
| GO:0045851 | pH reduction | 3/261 | 46/18723 | 0.026173902 | 0.106812405 | 0.077957127 | ATP6V0A1/CA2/GRN | 3 |
| GO:0048701 | embryonic cranial skeleton morphogenesis | 3/261 | 46/18723 | 0.026173902 | 0.106812405 | 0.077957127 | NDST1/PAX5/TGFBR2 | 3 |
| GO:0060976 | coronary vasculature development | 3/261 | 46/18723 | 0.026173902 | 0.106812405 | 0.077957127 | GPC3/NDST1/VEGFA | 3 |
| GO:0051100 | negative regulation of binding | 6/261 | 162/18723 | 0.026212407 | 0.10685276 | 0.07798658 | HMOX1/ITGA4/JUN/LRRK2/NOG/SLPI | 6 |
| GO:0014032 | neural crest cell development | 4/261 | 81/18723 | 0.02662361 | 0.108174712 | 0.078951408 | SEMA4A/SEMA4C/SEMA6A/SEMA6B | 4 |
| GO:0030641 | regulation of cellular pH | 4/261 | 81/18723 | 0.02662361 | 0.108174712 | 0.078951408 | ATP6V0A1/CA2/GRN/LRRK2 | 4 |
| GO:0048708 | astrocyte differentiation | 4/261 | 81/18723 | 0.02662361 | 0.108174712 | 0.078951408 | GRN/NOG/TLR4/TREM2 | 4 |
| GO:0051222 | positive regulation of protein transport | 9/261 | 303/18723 | 0.026727981 | 0.108480742 | 0.079174764 | GAS6/GPR68/HPS4/ITGB2/PCK2/SOX4/SRC/TLR4/TREM2 | 9 |
| GO:0007611 | learning or memory | 8/261 | 255/18723 | 0.026991547 | 0.109312837 | 0.079782069 | B4GALT2/GM2A/GPR88/JUN/KIT/LILRB2/TMOD2/TREM2 | 8 |
| GO:0051924 | regulation of calcium ion transport | 8/261 | 255/18723 | 0.026991547 | 0.109312837 | 0.079782069 | CAMK2D/CCL5/CXCL10/GPR35/HOMER3/LILRB2/P2RY6/TSPAN13 | 8 |
| GO:0043500 | muscle adaptation | 5/261 | 121/18723 | 0.027311495 | 0.110488755 | 0.080640314 | CAMK2D/GSN/HMOX1/LMNA/TWF1 | 5 |
| GO:0050792 | regulation of viral process | 6/261 | 164/18723 | 0.027617386 | 0.111186996 | 0.081149926 | CCL5/FCN1/GSN/SLPI/TOP2A/TRIM14 | 6 |
| GO:0010712 | regulation of collagen metabolic process | 3/261 | 47/18723 | 0.027678075 | 0.111186996 | 0.081149926 | CST3/FAP/MFAP4 | 3 |
| GO:0043114 | regulation of vascular permeability | 3/261 | 47/18723 | 0.027678075 | 0.111186996 | 0.081149926 | AZU1/SRC/VEGFA | 3 |
| GO:0050885 | neuromuscular process controlling balance | 3/261 | 47/18723 | 0.027678075 | 0.111186996 | 0.081149926 | GM2A/GPR88/TPP1 | 3 |
| GO:0051972 | regulation of telomerase activity | 3/261 | 47/18723 | 0.027678075 | 0.111186996 | 0.081149926 | MYC/PKIB/SRC | 3 |
| GO:0008543 | fibroblast growth factor receptor signaling pathway | 4/261 | 82/18723 | 0.02769253 | 0.111186996 | 0.081149926 | FGFR3/NDST1/NOG/SHISA2 | 4 |
| GO:0016575 | histone deacetylation | 4/261 | 82/18723 | 0.02769253 | 0.111186996 | 0.081149926 | CAMK2D/HDAC9/LRRK2/VEGFA | 4 |
| GO:0019730 | antimicrobial humoral response | 5/261 | 122/18723 | 0.028166702 | 0.112617191 | 0.082193755 | AZU1/CXCL10/LYZ/RNASE3/SLPI | 5 |
| GO:0050868 | negative regulation of T cell activation | 5/261 | 122/18723 | 0.028166702 | 0.112617191 | 0.082193755 | CD86/FCGR2B/LILRB2/LILRB4/PTPN22 | 5 |
| GO:0002460 | adaptive immune response based on somatic recombination of immune receptors built from immunoglobulin superfamily domains | 10/261 | 356/18723 | 0.028176275 | 0.112617191 | 0.082193755 | C3/CD1D/FCER2/FCGR2B/LILRB4/SEMA4A/SLC15A4/TLR4/TREM2/UNG | 10 |
| GO:0010738 | regulation of protein kinase A signaling | 2/261 | 19/18723 | 0.028320177 | 0.112617191 | 0.082193755 | AKAP7/LRRK2 | 2 |
| GO:0010759 | positive regulation of macrophage chemotaxis | 2/261 | 19/18723 | 0.028320177 | 0.112617191 | 0.082193755 | CCL5/CSF1R | 2 |
| GO:0010885 | regulation of cholesterol storage | 2/261 | 19/18723 | 0.028320177 | 0.112617191 | 0.082193755 | CD36/TREM2 | 2 |
| GO:0014067 | negative regulation of phosphatidylinositol 3-kinase signaling | 2/261 | 19/18723 | 0.028320177 | 0.112617191 | 0.082193755 | NLRC3/TREM2 | 2 |
| GO:0031065 | positive regulation of histone deacetylation | 2/261 | 19/18723 | 0.028320177 | 0.112617191 | 0.082193755 | LRRK2/VEGFA | 2 |
| GO:2001185 | regulation of CD8-positive, alpha-beta T cell activation | 2/261 | 19/18723 | 0.028320177 | 0.112617191 | 0.082193755 | LILRB4/PTPN22 | 2 |
| GO:0002673 | regulation of acute inflammatory response | 3/261 | 48/18723 | 0.029227397 | 0.115977791 | 0.084646492 | ALOX5AP/C3/FCGR2B | 3 |
| GO:0048146 | positive regulation of fibroblast proliferation | 3/261 | 48/18723 | 0.029227397 | 0.115977791 | 0.084646492 | ANXA2/GAS6/MYC | 3 |
| GO:0071900 | regulation of protein serine/threonine kinase activity | 10/261 | 359/18723 | 0.029604936 | 0.117351201 | 0.085648876 | CSF1R/KIT/KSR1/LRRK2/PKIB/PTPN22/SRC/TLR4/TRIB3/VEGFA | 10 |
| GO:0022604 | regulation of cell morphogenesis | 9/261 | 309/18723 | 0.029786593 | 0.117946065 | 0.086083039 | CDC42EP1/CSF1R/ITGB2/KIT/MYO10/SEMA4A/SRC/STRIP2/VEGFA | 9 |
| GO:0032370 | positive regulation of lipid transport | 4/261 | 84/18723 | 0.02990634 | 0.118128867 | 0.086216457 | ANXA2/GAL/PTCH1/TREM2 | 4 |
| GO:0097061 | dendritic spine organization | 4/261 | 84/18723 | 0.02990634 | 0.118128867 | 0.086216457 | CTTN/FCGR2B/LRRK2/TREM2 | 4 |
| GO:0001909 | leukocyte mediated cytotoxicity | 5/261 | 124/18723 | 0.029927667 | 0.118128867 | 0.086216457 | AZU1/CD1D/FCGR2B/PRDX1/TYROBP | 5 |
| GO:0051047 | positive regulation of secretion | 9/261 | 310/18723 | 0.030319215 | 0.119547993 | 0.087252207 | GAL/GPR68/ITGB2/PCK2/PTAFR/SOX4/TLR4/TREM2/XBP1 | 9 |
| GO:0014075 | response to amine | 3/261 | 49/18723 | 0.030821746 | 0.120283698 | 0.087789162 | ASS1/CNR2/HDAC9 | 3 |
| GO:0030225 | macrophage differentiation | 3/261 | 49/18723 | 0.030821746 | 0.120283698 | 0.087789162 | CSF1R/MMP9/VEGFA | 3 |
| GO:0030857 | negative regulation of epithelial cell differentiation | 3/261 | 49/18723 | 0.030821746 | 0.120283698 | 0.087789162 | MMP9/VEGFA/ZEB1 | 3 |
| GO:0038084 | vascular endothelial growth factor signaling pathway | 3/261 | 49/18723 | 0.030821746 | 0.120283698 | 0.087789162 | SEMA6A/TSPAN12/VEGFA | 3 |
| GO:0052372 | modulation by symbiont of entry into host | 3/261 | 49/18723 | 0.030821746 | 0.120283698 | 0.087789162 | FCN1/GSN/TRIM14 | 3 |
| GO:1904894 | positive regulation of receptor signaling pathway via STAT | 3/261 | 49/18723 | 0.030821746 | 0.120283698 | 0.087789162 | CCL5/KIT/PRLR | 3 |
| GO:0018210 | peptidyl-threonine modification | 5/261 | 125/18723 | 0.03083359 | 0.120283698 | 0.087789162 | AZU1/CAMK2D/CHI3L1/LRRK2/TGFBR2 | 5 |
| GO:0001656 | metanephros development | 4/261 | 85/18723 | 0.031051393 | 0.120283698 | 0.087789162 | EGR1/GPC3/MYC/PTCH1 | 4 |
| GO:0042509 | regulation of tyrosine phosphorylation of STAT protein | 4/261 | 85/18723 | 0.031051393 | 0.120283698 | 0.087789162 | CCL5/CSF1R/FGFR3/KIT | 4 |
| GO:0071277 | cellular response to calcium ion | 4/261 | 85/18723 | 0.031051393 | 0.120283698 | 0.087789162 | ALOX5AP/CAMK2D/JUNB/RASGRP2 | 4 |
| GO:0097006 | regulation of plasma lipoprotein particle levels | 4/261 | 85/18723 | 0.031051393 | 0.120283698 | 0.087789162 | ANXA2/CD36/HMOX1/TREM2 | 4 |
| GO:0015669 | gas transport | 2/261 | 20/18723 | 0.031182276 | 0.120283698 | 0.087789162 | CA2/MYC | 2 |
| GO:0032930 | positive regulation of superoxide anion generation | 2/261 | 20/18723 | 0.031182276 | 0.120283698 | 0.087789162 | ITGB2/TYROBP | 2 |
| GO:0032986 | protein-DNA complex disassembly | 2/261 | 20/18723 | 0.031182276 | 0.120283698 | 0.087789162 | MYC/NFE2 | 2 |
| GO:0043586 | tongue development | 2/261 | 20/18723 | 0.031182276 | 0.120283698 | 0.087789162 | CYP26B1/KIT | 2 |
| GO:0046629 | gamma-delta T cell activation | 2/261 | 20/18723 | 0.031182276 | 0.120283698 | 0.087789162 | JAML/SOX4 | 2 |
| GO:0060044 | negative regulation of cardiac muscle cell proliferation | 2/261 | 20/18723 | 0.031182276 | 0.120283698 | 0.087789162 | NOG/TGFBR2 | 2 |
| GO:0072567 | chemokine (C-X-C motif) ligand 2 production | 2/261 | 20/18723 | 0.031182276 | 0.120283698 | 0.087789162 | MCOLN2/TLR4 | 2 |
| GO:0098543 | detection of other organism | 2/261 | 20/18723 | 0.031182276 | 0.120283698 | 0.087789162 | CD1D/TLR4 | 2 |
| GO:1902236 | negative regulation of endoplasmic reticulum stress-induced intrinsic apoptotic signaling pathway | 2/261 | 20/18723 | 0.031182276 | 0.120283698 | 0.087789162 | LRRK2/XBP1 | 2 |
| GO:2000341 | regulation of chemokine (C-X-C motif) ligand 2 production | 2/261 | 20/18723 | 0.031182276 | 0.120283698 | 0.087789162 | MCOLN2/TLR4 | 2 |
| GO:0043588 | skin development | 8/261 | 263/18723 | 0.031598041 | 0.121761697 | 0.088867881 | ASAH1/CD109/CSTA/CYP26B1/GAL/ITGA6/TNFRSF19/TRIM16 | 8 |
| GO:0014031 | mesenchymal cell development | 4/261 | 86/18723 | 0.032221971 | 0.123655541 | 0.090250104 | SEMA4A/SEMA4C/SEMA6A/SEMA6B | 4 |
| GO:0048864 | stem cell development | 4/261 | 86/18723 | 0.032221971 | 0.123655541 | 0.090250104 | SEMA4A/SEMA4C/SEMA6A/SEMA6B | 4 |
| GO:1903510 | mucopolysaccharide metabolic process | 4/261 | 86/18723 | 0.032221971 | 0.123655541 | 0.090250104 | GNS/NDST1/SPOCK2/XYLT1 | 4 |
| GO:2000117 | negative regulation of cysteine-type endopeptidase activity | 4/261 | 86/18723 | 0.032221971 | 0.123655541 | 0.090250104 | GAS6/MMP9/SRC/VEGFA | 4 |
| GO:0001961 | positive regulation of cytokine-mediated signaling pathway | 3/261 | 50/18723 | 0.032460975 | 0.123809276 | 0.090362308 | CD300LF/GAS6/TREM2 | 3 |
| GO:0019083 | viral transcription | 3/261 | 50/18723 | 0.032460975 | 0.123809276 | 0.090362308 | CCL5/JUN/TRIM14 | 3 |
| GO:0038093 | Fc receptor signaling pathway | 3/261 | 50/18723 | 0.032460975 | 0.123809276 | 0.090362308 | FCGR2B/KIT/SRC | 3 |
| GO:0046638 | positive regulation of alpha-beta T cell differentiation | 3/261 | 50/18723 | 0.032460975 | 0.123809276 | 0.090362308 | CD86/LILRB4/TGFBR2 | 3 |
| GO:0060688 | regulation of morphogenesis of a branching structure | 3/261 | 50/18723 | 0.032460975 | 0.123809276 | 0.090362308 | LRRK2/NOG/VEGFA | 3 |
| GO:1904036 | negative regulation of epithelial cell apoptotic process | 3/261 | 50/18723 | 0.032460975 | 0.123809276 | 0.090362308 | GAS6/HMOX1/TERT | 3 |
| GO:0030522 | intracellular receptor signaling pathway | 8/261 | 265/18723 | 0.03282883 | 0.125084542 | 0.091293062 | CYP26B1/NR2F1/PTPN22/SLC15A4/SRC/TLR4/TRIM16/ZNF366 | 8 |
| GO:0019915 | lipid storage | 4/261 | 87/18723 | 0.033418134 | 0.126516259 | 0.092338002 | C3/CD36/GM2A/TREM2 | 4 |
| GO:0042445 | hormone metabolic process | 7/261 | 218/18723 | 0.033534267 | 0.126516259 | 0.092338002 | CYP26B1/EGR1/FURIN/GAL/STAR/STC2/TIPARP | 7 |
| GO:0007219 | Notch signaling pathway | 6/261 | 172/18723 | 0.033732367 | 0.126516259 | 0.092338002 | ANXA4/CHAC1/EGFL7/KIT/SRC/TGFBR2 | 6 |
| GO:0030833 | regulation of actin filament polymerization | 6/261 | 172/18723 | 0.033732367 | 0.126516259 | 0.092338002 | CDC42EP1/CTTN/GSN/IQGAP2/TMOD2/TWF1 | 6 |
| GO:0048736 | appendage development | 6/261 | 172/18723 | 0.033732367 | 0.126516259 | 0.092338002 | CYP26B1/GPC3/ITGA6/NOG/PTCH1/SOX4 | 6 |
| GO:0060173 | limb development | 6/261 | 172/18723 | 0.033732367 | 0.126516259 | 0.092338002 | CYP26B1/GPC3/ITGA6/NOG/PTCH1/SOX4 | 6 |
| GO:0006636 | unsaturated fatty acid biosynthetic process | 3/261 | 51/18723 | 0.034144913 | 0.126516259 | 0.092338002 | FADS2/PTGS1/SCD | 3 |
| GO:0032873 | negative regulation of stress-activated MAPK cascade | 3/261 | 51/18723 | 0.034144913 | 0.126516259 | 0.092338002 | MYC/PTPN22/TREM2 | 3 |
| GO:0046850 | regulation of bone remodeling | 3/261 | 51/18723 | 0.034144913 | 0.126516259 | 0.092338002 | CA2/CSF1R/SRC | 3 |
| GO:0070303 | negative regulation of stress-activated protein kinase signaling cascade | 3/261 | 51/18723 | 0.034144913 | 0.126516259 | 0.092338002 | MYC/PTPN22/TREM2 | 3 |
| GO:0071622 | regulation of granulocyte chemotaxis | 3/261 | 51/18723 | 0.034144913 | 0.126516259 | 0.092338002 | CCL5/CSF1R/THBS4 | 3 |
| GO:0006067 | ethanol metabolic process | 2/261 | 21/18723 | 0.034153291 | 0.126516259 | 0.092338002 | ALDH2/ALDH3B1 | 2 |
| GO:0009070 | serine family amino acid biosynthetic process | 2/261 | 21/18723 | 0.034153291 | 0.126516259 | 0.092338002 | PHGDH/PSAT1 | 2 |
| GO:0010878 | cholesterol storage | 2/261 | 21/18723 | 0.034153291 | 0.126516259 | 0.092338002 | CD36/TREM2 | 2 |
| GO:0010988 | regulation of low-density lipoprotein particle clearance | 2/261 | 21/18723 | 0.034153291 | 0.126516259 | 0.092338002 | ANXA2/TREM2 | 2 |
| GO:0035455 | response to interferon-alpha | 2/261 | 21/18723 | 0.034153291 | 0.126516259 | 0.092338002 | GAS6/STAR | 2 |
| GO:0038083 | peptidyl-tyrosine autophosphorylation | 2/261 | 21/18723 | 0.034153291 | 0.126516259 | 0.092338002 | SRC/VEGFA | 2 |
| GO:0045056 | transcytosis | 2/261 | 21/18723 | 0.034153291 | 0.126516259 | 0.092338002 | PTAFR/SRC | 2 |
| GO:0046128 | purine ribonucleoside metabolic process | 2/261 | 21/18723 | 0.034153291 | 0.126516259 | 0.092338002 | ACP3/ADA2 | 2 |
| GO:0046514 | ceramide catabolic process | 2/261 | 21/18723 | 0.034153291 | 0.126516259 | 0.092338002 | ASAH1/GM2A | 2 |
| GO:0046597 | negative regulation of viral entry into host cell | 2/261 | 21/18723 | 0.034153291 | 0.126516259 | 0.092338002 | FCN1/GSN | 2 |
| GO:0046641 | positive regulation of alpha-beta T cell proliferation | 2/261 | 21/18723 | 0.034153291 | 0.126516259 | 0.092338002 | PTPN22/TGFBR2 | 2 |
| GO:0046885 | regulation of hormone biosynthetic process | 2/261 | 21/18723 | 0.034153291 | 0.126516259 | 0.092338002 | EGR1/STC2 | 2 |
| GO:0048745 | smooth muscle tissue development | 2/261 | 21/18723 | 0.034153291 | 0.126516259 | 0.092338002 | PTCH1/TIPARP | 2 |
| GO:0090026 | positive regulation of monocyte chemotaxis | 2/261 | 21/18723 | 0.034153291 | 0.126516259 | 0.092338002 | CCL5/CXCL10 | 2 |
| GO:0090189 | regulation of branching involved in ureteric bud morphogenesis | 2/261 | 21/18723 | 0.034153291 | 0.126516259 | 0.092338002 | NOG/VEGFA | 2 |
| GO:0150146 | cell junction disassembly | 2/261 | 21/18723 | 0.034153291 | 0.126516259 | 0.092338002 | C3/TREM2 | 2 |
| GO:2001014 | regulation of skeletal muscle cell differentiation | 2/261 | 21/18723 | 0.034153291 | 0.126516259 | 0.092338002 | CYP26B1/RBM24 | 2 |
| GO:0007260 | tyrosine phosphorylation of STAT protein | 4/261 | 88/18723 | 0.034639933 | 0.127938188 | 0.093375798 | CCL5/CSF1R/FGFR3/KIT | 4 |
| GO:0030101 | natural killer cell activation | 4/261 | 88/18723 | 0.034639933 | 0.127938188 | 0.093375798 | GAS6/PRDX1/PTPN22/TYROBP | 4 |
| GO:0070098 | chemokine-mediated signaling pathway | 4/261 | 88/18723 | 0.034639933 | 0.127938188 | 0.093375798 | CCL5/CXCL10/GPR35/TREM2 | 4 |
| GO:0001894 | tissue homeostasis | 8/261 | 268/18723 | 0.034735752 | 0.128165315 | 0.093541567 | CA2/CSF1R/LYZ/PRDX1/SRC/TLR4/TPP1/VEGFA | 8 |
| GO:0006816 | calcium ion transport | 11/261 | 422/18723 | 0.035119973 | 0.129455063 | 0.094482891 | ANXA2/CAMK2D/CCL5/CXCL10/GAS6/GPR35/HOMER3/LILRB2/MCOLN2/P2RY6/TSPAN13 | 11 |
| GO:0008202 | steroid metabolic process | 9/261 | 319/18723 | 0.035415189 | 0.130286024 | 0.095089368 | ASAH1/CYP26B1/EGR1/GAL/KIT/PRLR/STAR/STS/TIPARP | 9 |
| GO:1904951 | positive regulation of establishment of protein localization | 9/261 | 319/18723 | 0.035415189 | 0.130286024 | 0.095089368 | GAS6/GPR68/HPS4/ITGB2/PCK2/SOX4/SRC/TLR4/TREM2 | 9 |
| GO:0048260 | positive regulation of receptor-mediated endocytosis | 3/261 | 52/18723 | 0.035873365 | 0.131634168 | 0.096073312 | ANXA2/C3/VEGFA | 3 |
| GO:0072132 | mesenchyme morphogenesis | 3/261 | 52/18723 | 0.035873365 | 0.131634168 | 0.096073312 | MYC/NOG/TGFBR2 | 3 |
| GO:1900407 | regulation of cellular response to oxidative stress | 4/261 | 89/18723 | 0.035887408 | 0.131634168 | 0.096073312 | CD36/LRRK2/TLR4/TREM2 | 4 |
| GO:0045638 | negative regulation of myeloid cell differentiation | 4/261 | 90/18723 | 0.037160595 | 0.133926331 | 0.097746249 | GPR68/LILRB4/MYC/TLR4 | 4 |
| GO:0046849 | bone remodeling | 4/261 | 90/18723 | 0.037160595 | 0.133926331 | 0.097746249 | CA2/CSF1R/SRC/TPP1 | 4 |
| GO:0097581 | lamellipodium organization | 4/261 | 90/18723 | 0.037160595 | 0.133926331 | 0.097746249 | CTTN/KIT/SRC/TWF1 | 4 |
| GO:0099175 | regulation of postsynapse organization | 4/261 | 90/18723 | 0.037160595 | 0.133926331 | 0.097746249 | FCGR2B/LILRB2/LRRK2/SRGN | 4 |
| GO:0050808 | synapse organization | 11/261 | 426/18723 | 0.037195443 | 0.133926331 | 0.097746249 | C3/COLQ/CTTN/FCGR2B/LILRB2/LRRK2/SEMA4A/SEPTIN11/SPOCK2/SRGN/TREM2 | 11 |
| GO:0002053 | positive regulation of mesenchymal cell proliferation | 2/261 | 22/18723 | 0.037229655 | 0.133926331 | 0.097746249 | MYC/TGFBR2 | 2 |
| GO:0002320 | lymphoid progenitor cell differentiation | 2/261 | 22/18723 | 0.037229655 | 0.133926331 | 0.097746249 | KIT/SOX4 | 2 |
| GO:0008211 | glucocorticoid metabolic process | 2/261 | 22/18723 | 0.037229655 | 0.133926331 | 0.097746249 | GAL/STAR | 2 |
| GO:0022616 | DNA strand elongation | 2/261 | 22/18723 | 0.037229655 | 0.133926331 | 0.097746249 | RFC3/TERT | 2 |
| GO:0032816 | positive regulation of natural killer cell activation | 2/261 | 22/18723 | 0.037229655 | 0.133926331 | 0.097746249 | GAS6/TYROBP | 2 |
| GO:0036120 | cellular response to platelet-derived growth factor stimulus | 2/261 | 22/18723 | 0.037229655 | 0.133926331 | 0.097746249 | SRC/TLR4 | 2 |
| GO:0044346 | fibroblast apoptotic process | 2/261 | 22/18723 | 0.037229655 | 0.133926331 | 0.097746249 | GAS6/MYC | 2 |
| GO:0050860 | negative regulation of T cell receptor signaling pathway | 2/261 | 22/18723 | 0.037229655 | 0.133926331 | 0.097746249 | LILRB4/PTPN22 | 2 |
| GO:0060563 | neuroepithelial cell differentiation | 2/261 | 22/18723 | 0.037229655 | 0.133926331 | 0.097746249 | FAM20C/SOX4 | 2 |
| GO:0061042 | vascular wound healing | 2/261 | 22/18723 | 0.037229655 | 0.133926331 | 0.097746249 | VEGFA/XBP1 | 2 |
| GO:0071404 | cellular response to low-density lipoprotein particle stimulus | 2/261 | 22/18723 | 0.037229655 | 0.133926331 | 0.097746249 | CD36/ITGB2 | 2 |
| GO:0071467 | cellular response to pH | 2/261 | 22/18723 | 0.037229655 | 0.133926331 | 0.097746249 | GNA11/GPR68 | 2 |
| GO:1901522 | positive regulation of transcription from RNA polymerase II promoter involved in cellular response to chemical stimulus | 2/261 | 22/18723 | 0.037229655 | 0.133926331 | 0.097746249 | VEGFA/XBP1 | 2 |
| GO:1903589 | positive regulation of blood vessel endothelial cell proliferation involved in sprouting angiogenesis | 2/261 | 22/18723 | 0.037229655 | 0.133926331 | 0.097746249 | HMOX1/VEGFA | 2 |
| GO:2000114 | regulation of establishment of cell polarity | 2/261 | 22/18723 | 0.037229655 | 0.133926331 | 0.097746249 | GSN/RAP1B | 2 |
| GO:0001541 | ovarian follicle development | 3/261 | 53/18723 | 0.037646115 | 0.135294124 | 0.098744535 | KIT/SRC/VEGFA | 3 |
| GO:0030324 | lung development | 6/261 | 177/18723 | 0.037966774 | 0.136315322 | 0.099489857 | CHI3L1/GPC3/NOG/RXFP1/TGFBR2/VEGFA | 6 |
| GO:0006885 | regulation of pH | 4/261 | 91/18723 | 0.038459519 | 0.13768729 | 0.100491189 | ATP6V0A1/CA2/GRN/LRRK2 | 4 |
| GO:0014033 | neural crest cell differentiation | 4/261 | 91/18723 | 0.038459519 | 0.13768729 | 0.100491189 | SEMA4A/SEMA4C/SEMA6A/SEMA6B | 4 |
| GO:0019935 | cyclic-nucleotide-mediated signaling | 4/261 | 91/18723 | 0.038459519 | 0.13768729 | 0.100491189 | CAP2/CD36/GAL/KSR1 | 4 |
| GO:0030834 | regulation of actin filament depolymerization | 3/261 | 54/18723 | 0.039462928 | 0.141144226 | 0.103014236 | GSN/TMOD2/TWF1 | 3 |
| GO:0010640 | regulation of platelet-derived growth factor receptor signaling pathway | 2/261 | 23/18723 | 0.040407877 | 0.143152763 | 0.104480169 | F3/SRC | 2 |
| GO:0019430 | removal of superoxide radicals | 2/261 | 23/18723 | 0.040407877 | 0.143152763 | 0.104480169 | CD36/PRDX1 | 2 |
| GO:0030194 | positive regulation of blood coagulation | 2/261 | 23/18723 | 0.040407877 | 0.143152763 | 0.104480169 | CD36/F3 | 2 |
| GO:0031338 | regulation of vesicle fusion | 2/261 | 23/18723 | 0.040407877 | 0.143152763 | 0.104480169 | ANXA2/TBC1D4 | 2 |
| GO:0036119 | response to platelet-derived growth factor | 2/261 | 23/18723 | 0.040407877 | 0.143152763 | 0.104480169 | SRC/TLR4 | 2 |
| GO:0036303 | lymph vessel morphogenesis | 2/261 | 23/18723 | 0.040407877 | 0.143152763 | 0.104480169 | PTPN14/VEGFA | 2 |
| GO:0043931 | ossification involved in bone maturation | 2/261 | 23/18723 | 0.040407877 | 0.143152763 | 0.104480169 | RFLNB/XYLT1 | 2 |
| GO:0050765 | negative regulation of phagocytosis | 2/261 | 23/18723 | 0.040407877 | 0.143152763 | 0.104480169 | CD300LF/FCGR2B | 2 |
| GO:0060143 | positive regulation of syncytium formation by plasma membrane fusion | 2/261 | 23/18723 | 0.040407877 | 0.143152763 | 0.104480169 | TREM2/TYROBP | 2 |
| GO:1900048 | positive regulation of hemostasis | 2/261 | 23/18723 | 0.040407877 | 0.143152763 | 0.104480169 | CD36/F3 | 2 |
| GO:0072503 | cellular divalent inorganic cation homeostasis | 12/261 | 486/18723 | 0.040510891 | 0.143381675 | 0.104647241 | CAMK2D/CCL5/CD36/CXCL10/GPR35/MCOLN2/MT1E/MT1G/MT1X/MT2A/P2RY6/STC2 | 12 |
| GO:0071897 | DNA biosynthetic process | 6/261 | 180/18723 | 0.040663259 | 0.143784667 | 0.104941364 | DTL/MYC/PKIB/RFC3/SRC/TERT | 6 |
| GO:0033273 | response to vitamin | 4/261 | 93/18723 | 0.041134637 | 0.145176499 | 0.105957195 | CXCL10/CYP26B1/GAS6/STC2 | 4 |
| GO:0106027 | neuron projection organization | 4/261 | 93/18723 | 0.041134637 | 0.145176499 | 0.105957195 | CTTN/FCGR2B/LRRK2/TREM2 | 4 |
| GO:0010676 | positive regulation of cellular carbohydrate metabolic process | 3/261 | 55/18723 | 0.041323544 | 0.145705488 | 0.106343278 | P2RY6/PTAFR/SRC | 3 |
| GO:0008361 | regulation of cell size | 6/261 | 181/18723 | 0.041588341 | 0.146362739 | 0.106822973 | CTTN/SEMA4A/SEMA4C/SEMA6A/SEMA6B/VEGFA | 6 |
| GO:0030323 | respiratory tube development | 6/261 | 181/18723 | 0.041588341 | 0.146362739 | 0.106822973 | CHI3L1/GPC3/NOG/RXFP1/TGFBR2/VEGFA | 6 |
| GO:0072073 | kidney epithelium development | 5/261 | 136/18723 | 0.041939553 | 0.147321065 | 0.107522408 | GPC3/MYC/NOG/PTCH1/VEGFA | 5 |
| GO:1900180 | regulation of protein localization to nucleus | 5/261 | 136/18723 | 0.041939553 | 0.147321065 | 0.107522408 | CD36/LILRB4/LMNA/SRC/TERT | 5 |
| GO:0019080 | viral gene expression | 4/261 | 94/18723 | 0.042510843 | 0.149047406 | 0.108782379 | CCL5/FURIN/JUN/TRIM14 | 4 |
| GO:0032091 | negative regulation of protein binding | 4/261 | 94/18723 | 0.042510843 | 0.149047406 | 0.108782379 | ITGA4/LRRK2/NOG/SLPI | 4 |
| GO:0050714 | positive regulation of protein secretion | 5/261 | 137/18723 | 0.043054341 | 0.150339249 | 0.109725232 | GPR68/PCK2/SOX4/TLR4/TREM2 | 5 |
| GO:0021700 | developmental maturation | 8/261 | 280/18723 | 0.043111611 | 0.150339249 | 0.109725232 | C3/FGFR3/GAL/LRRK2/MMP2/RFLNB/VEGFA/XYLT1 | 8 |
| GO:0010332 | response to gamma radiation | 3/261 | 56/18723 | 0.043227687 | 0.150339249 | 0.109725232 | CXCL10/EGR1/MYC | 3 |
| GO:0045599 | negative regulation of fat cell differentiation | 3/261 | 56/18723 | 0.043227687 | 0.150339249 | 0.109725232 | SORT1/TRIB3/ZFP36L2 | 3 |
| GO:0014706 | striated muscle tissue development | 10/261 | 384/18723 | 0.043556028 | 0.150339249 | 0.109725232 | CYP26B1/EGR1/HDAC9/LMNA/MSC/NOG/PAX5/RBM24/TGFBR2/VEGFA | 10 |
| GO:0002438 | acute inflammatory response to antigenic stimulus | 2/261 | 24/18723 | 0.043684543 | 0.150339249 | 0.109725232 | C3/FCGR2B | 2 |
| GO:0002755 | MyD88-dependent toll-like receptor signaling pathway | 2/261 | 24/18723 | 0.043684543 | 0.150339249 | 0.109725232 | CD300LF/TLR4 | 2 |
| GO:0003181 | atrioventricular valve morphogenesis | 2/261 | 24/18723 | 0.043684543 | 0.150339249 | 0.109725232 | SOX4/TGFBR2 | 2 |
| GO:0007035 | vacuolar acidification | 2/261 | 24/18723 | 0.043684543 | 0.150339249 | 0.109725232 | ATP6V0A1/GRN | 2 |
| GO:0032928 | regulation of superoxide anion generation | 2/261 | 24/18723 | 0.043684543 | 0.150339249 | 0.109725232 | ITGB2/TYROBP | 2 |
| GO:0043302 | positive regulation of leukocyte degranulation | 2/261 | 24/18723 | 0.043684543 | 0.150339249 | 0.109725232 | ITGB2/PTAFR | 2 |
| GO:0048011 | neurotrophin TRK receptor signaling pathway | 2/261 | 24/18723 | 0.043684543 | 0.150339249 | 0.109725232 | SORT1/SRC | 2 |
| GO:0050820 | positive regulation of coagulation | 2/261 | 24/18723 | 0.043684543 | 0.150339249 | 0.109725232 | CD36/F3 | 2 |
| GO:0050855 | regulation of B cell receptor signaling pathway | 2/261 | 24/18723 | 0.043684543 | 0.150339249 | 0.109725232 | FCGR2B/PTPN22 | 2 |
| GO:0051000 | positive regulation of nitric-oxide synthase activity | 2/261 | 24/18723 | 0.043684543 | 0.150339249 | 0.109725232 | FCER2/TERT | 2 |
| GO:0051043 | regulation of membrane protein ectodomain proteolysis | 2/261 | 24/18723 | 0.043684543 | 0.150339249 | 0.109725232 | FURIN/TIMP1 | 2 |
| GO:0051570 | regulation of histone H3-K9 methylation | 2/261 | 24/18723 | 0.043684543 | 0.150339249 | 0.109725232 | LMNA/PAX5 | 2 |
| GO:0120255 | olefinic compound biosynthetic process | 2/261 | 24/18723 | 0.043684543 | 0.150339249 | 0.109725232 | EGR1/STAR | 2 |
| GO:1903428 | positive regulation of reactive oxygen species biosynthetic process | 2/261 | 24/18723 | 0.043684543 | 0.150339249 | 0.109725232 | CD36/TLR4 | 2 |
| GO:2000209 | regulation of anoikis | 2/261 | 24/18723 | 0.043684543 | 0.150339249 | 0.109725232 | ITGA5/SRC | 2 |
| GO:0007044 | cell-substrate junction assembly | 4/261 | 95/18723 | 0.043912806 | 0.150708104 | 0.109994441 | CTTN/ITGA6/SRC/VEGFA | 4 |
| GO:0008585 | female gonad development | 4/261 | 95/18723 | 0.043912806 | 0.150708104 | 0.109994441 | KIT/SRC/TIPARP/VEGFA | 4 |
| GO:0008593 | regulation of Notch signaling pathway | 4/261 | 95/18723 | 0.043912806 | 0.150708104 | 0.109994441 | CHAC1/EGFL7/KIT/SRC | 4 |
| GO:0035107 | appendage morphogenesis | 5/261 | 138/18723 | 0.04418684 | 0.151370332 | 0.110477769 | CYP26B1/GPC3/NOG/PTCH1/SOX4 | 5 |
| GO:0035108 | limb morphogenesis | 5/261 | 138/18723 | 0.04418684 | 0.151370332 | 0.110477769 | CYP26B1/GPC3/NOG/PTCH1/SOX4 | 5 |
| GO:2001257 | regulation of cation channel activity | 6/261 | 184/18723 | 0.044442925 | 0.152108049 | 0.111016192 | CAMK2D/GAL/GPR35/MMP9/P2RY6/TREM2 | 6 |
| GO:0050804 | modulation of chemical synaptic transmission | 11/261 | 439/18723 | 0.044532691 | 0.152275702 | 0.111138554 | AKAP7/CNR2/DNM1/KIT/LILRB2/LRRK2/RAP1B/SRC/SRGN/STAR/TYROBP | 11 |
| GO:1903532 | positive regulation of secretion by cell | 8/261 | 282/18723 | 0.044626708 | 0.152457573 | 0.111271293 | GAL/GPR68/ITGB2/PCK2/PTAFR/SOX4/TLR4/TREM2 | 8 |
| GO:0051090 | regulation of DNA-binding transcription factor activity | 11/261 | 440/18723 | 0.045135485 | 0.153908466 | 0.112330228 | ANXA4/CD36/GAS6/HMOX1/KIT/NLRC3/NLRP12/PTCH1/TLR4/TRIM14/VEGFA | 11 |
| GO:0099177 | regulation of trans-synaptic signaling | 11/261 | 440/18723 | 0.045135485 | 0.153908466 | 0.112330228 | AKAP7/CNR2/DNM1/KIT/LILRB2/LRRK2/RAP1B/SRC/SRGN/STAR/TYROBP | 11 |
| GO:0010883 | regulation of lipid storage | 3/261 | 57/18723 | 0.045175061 | 0.153908466 | 0.112330228 | C3/CD36/TREM2 | 3 |
| GO:0007160 | cell-matrix adhesion | 7/261 | 233/18723 | 0.045301727 | 0.154179105 | 0.112527754 | CD36/CTTN/ITGA4/ITGB2/ITGB7/SRC/VEGFA | 7 |
| GO:0030216 | keratinocyte differentiation | 5/261 | 139/18723 | 0.04533708 | 0.154179105 | 0.112527754 | ASAH1/CD109/CSTA/CYP26B1/TRIM16 | 5 |
| GO:0043433 | negative regulation of DNA-binding transcription factor activity | 6/261 | 185/18723 | 0.045421022 | 0.1542966 | 0.112613508 | ANXA4/GAS6/HMOX1/NLRC3/NLRP12/PTCH1 | 6 |
| GO:1901361 | organic cyclic compound catabolic process | 12/261 | 495/18723 | 0.045454274 | 0.1542966 | 0.112613508 | ADA2/ALDH1L2/HMOX1/HPD/RBM24/RNASE3/SAMD4A/SAMHD1/STS/TENT5A/UNG/ZFP36L2 | 12 |
| GO:0031345 | negative regulation of cell projection organization | 6/261 | 186/18723 | 0.04641246 | 0.156408022 | 0.114154532 | LRRK2/NR2F1/SEMA4A/SEMA4C/SEMA6A/SEMA6B | 6 |
| GO:0043123 | positive regulation of I-kappaB kinase/NF-kappaB signaling | 6/261 | 186/18723 | 0.04641246 | 0.156408022 | 0.114154532 | CD36/ECM1/HMOX1/TLR4/TNFRSF19/TRIM14 | 6 |
| GO:0070167 | regulation of biomineral tissue development | 4/261 | 97/18723 | 0.04679394 | 0.156408022 | 0.114154532 | ECM1/GAS6/RFLNB/SRGN | 4 |
| GO:0120162 | positive regulation of cold-induced thermogenesis | 4/261 | 97/18723 | 0.04679394 | 0.156408022 | 0.114154532 | CD36/PRLR/SCD/VEGFA | 4 |
| GO:1990868 | response to chemokine | 4/261 | 97/18723 | 0.04679394 | 0.156408022 | 0.114154532 | CCL5/CXCL10/GPR35/TREM2 | 4 |
| GO:1990869 | cellular response to chemokine | 4/261 | 97/18723 | 0.04679394 | 0.156408022 | 0.114154532 | CCL5/CXCL10/GPR35/TREM2 | 4 |
| GO:0003071 | renal system process involved in regulation of systemic arterial blood pressure | 2/261 | 25/18723 | 0.047056315 | 0.156408022 | 0.114154532 | GAS6/SUCNR1 | 2 |
| GO:0010954 | positive regulation of protein processing | 2/261 | 25/18723 | 0.047056315 | 0.156408022 | 0.114154532 | GSN/SRC | 2 |
| GO:0015012 | heparan sulfate proteoglycan biosynthetic process | 2/261 | 25/18723 | 0.047056315 | 0.156408022 | 0.114154532 | NDST1/XYLT1 | 2 |
| GO:0031579 | membrane raft organization | 2/261 | 25/18723 | 0.047056315 | 0.156408022 | 0.114154532 | ANXA2/GSN | 2 |
| GO:0031664 | regulation of lipopolysaccharide-mediated signaling pathway | 2/261 | 25/18723 | 0.047056315 | 0.156408022 | 0.114154532 | CD180/CD36 | 2 |
| GO:0032753 | positive regulation of interleukin-4 production | 2/261 | 25/18723 | 0.047056315 | 0.156408022 | 0.114154532 | CD86/SLC7A5 | 2 |
| GO:0032878 | regulation of establishment or maintenance of cell polarity | 2/261 | 25/18723 | 0.047056315 | 0.156408022 | 0.114154532 | GSN/RAP1B | 2 |
| GO:0033622 | integrin activation | 2/261 | 25/18723 | 0.047056315 | 0.156408022 | 0.114154532 | RAP1B/SRC | 2 |
| GO:0042730 | fibrinolysis | 2/261 | 25/18723 | 0.047056315 | 0.156408022 | 0.114154532 | ANXA2/FAP | 2 |
| GO:0048143 | astrocyte activation | 2/261 | 25/18723 | 0.047056315 | 0.156408022 | 0.114154532 | GRN/TREM2 | 2 |
| GO:0071450 | cellular response to oxygen radical | 2/261 | 25/18723 | 0.047056315 | 0.156408022 | 0.114154532 | CD36/PRDX1 | 2 |
| GO:0071451 | cellular response to superoxide | 2/261 | 25/18723 | 0.047056315 | 0.156408022 | 0.114154532 | CD36/PRDX1 | 2 |
| GO:0090312 | positive regulation of protein deacetylation | 2/261 | 25/18723 | 0.047056315 | 0.156408022 | 0.114154532 | LRRK2/VEGFA | 2 |
| GO:1901623 | regulation of lymphocyte chemotaxis | 2/261 | 25/18723 | 0.047056315 | 0.156408022 | 0.114154532 | CCL5/CXCL10 | 2 |
| GO:1902932 | positive regulation of alcohol biosynthetic process | 2/261 | 25/18723 | 0.047056315 | 0.156408022 | 0.114154532 | P2RY6/PTAFR | 2 |
| GO:1903901 | negative regulation of viral life cycle | 2/261 | 25/18723 | 0.047056315 | 0.156408022 | 0.114154532 | FCN1/GSN | 2 |
| GO:1904385 | cellular response to angiotensin | 2/261 | 25/18723 | 0.047056315 | 0.156408022 | 0.114154532 | CA2/SRC | 2 |
| GO:0002931 | response to ischemia | 3/261 | 58/18723 | 0.047165354 | 0.156408022 | 0.114154532 | CSF1R/EGR1/TREM2 | 3 |
| GO:0043525 | positive regulation of neuron apoptotic process | 3/261 | 58/18723 | 0.047165354 | 0.156408022 | 0.114154532 | GRN/JUN/TYROBP | 3 |
| GO:0045604 | regulation of epidermal cell differentiation | 3/261 | 58/18723 | 0.047165354 | 0.156408022 | 0.114154532 | CD109/PTCH1/TRIM16 | 3 |
| GO:0006631 | fatty acid metabolic process | 10/261 | 390/18723 | 0.047475702 | 0.157297492 | 0.114803712 | ALOX5AP/ASAH1/C3/CD36/FADS2/PCK2/PTGS1/SCD/TRIB3/XBP1 | 10 |
| GO:0016241 | regulation of macroautophagy | 5/261 | 141/18723 | 0.047690882 | 0.15787035 | 0.115221812 | ATP6V0A1/ATP6V1C2/HMOX1/LRRK2/QSOX1 | 5 |
| GO:0048762 | mesenchymal cell differentiation | 7/261 | 236/18723 | 0.047936765 | 0.158543737 | 0.115713285 | NOG/RFLNB/SEMA4A/SEMA4C/SEMA6A/SEMA6B/TGFBR2 | 7 |
| GO:1902882 | regulation of response to oxidative stress | 4/261 | 98/18723 | 0.04827306 | 0.159373657 | 0.116319002 | CD36/LRRK2/TLR4/TREM2 | 4 |
| GO:2001243 | negative regulation of intrinsic apoptotic signaling pathway | 4/261 | 98/18723 | 0.04827306 | 0.159373657 | 0.116319002 | LRRK2/MMP9/SRC/XBP1 | 4 |
| GO:0008064 | regulation of actin polymerization or depolymerization | 6/261 | 188/18723 | 0.048435484 | 0.159768637 | 0.116607279 | CDC42EP1/CTTN/GSN/IQGAP2/TMOD2/TWF1 | 6 |
| GO:0001836 | release of cytochrome c from mitochondria | 3/261 | 59/18723 | 0.049198236 | 0.16128728 | 0.117715662 | JUN/LMNA/MMP9 | 3 |
| GO:0002820 | negative regulation of adaptive immune response | 3/261 | 59/18723 | 0.049198236 | 0.16128728 | 0.117715662 | FCGR2B/LILRB4/SAMSN1 | 3 |
| GO:0030042 | actin filament depolymerization | 3/261 | 59/18723 | 0.049198236 | 0.16128728 | 0.117715662 | GSN/TMOD2/TWF1 | 3 |
| GO:0032768 | regulation of monooxygenase activity | 3/261 | 59/18723 | 0.049198236 | 0.16128728 | 0.117715662 | CNR2/FCER2/TERT | 3 |
| GO:0048010 | vascular endothelial growth factor receptor signaling pathway | 3/261 | 59/18723 | 0.049198236 | 0.16128728 | 0.117715662 | ITGA5/SRC/VEGFA | 3 |
| GO:0090303 | positive regulation of wound healing | 3/261 | 59/18723 | 0.049198236 | 0.16128728 | 0.117715662 | CD36/F3/XBP1 | 3 |
| GO:2000351 | regulation of endothelial cell apoptotic process | 3/261 | 59/18723 | 0.049198236 | 0.16128728 | 0.117715662 | GAS6/ITGA4/TERT | 3 |
| GO:0030832 | regulation of actin filament length | 6/261 | 189/18723 | 0.049467126 | 0.162026533 | 0.118255206 | CDC42EP1/CTTN/GSN/IQGAP2/TMOD2/TWF1 | 6 |
| GO:0021510 | spinal cord development | 4/261 | 99/18723 | 0.049777834 | 0.162332255 | 0.118478338 | NOG/PHGDH/PTCH1/SOX4 | 4 |
| GO:0030838 | positive regulation of actin filament polymerization | 4/261 | 99/18723 | 0.049777834 | 0.162332255 | 0.118478338 | CDC42EP1/CTTN/GSN/IQGAP2 | 4 |
| GO:0042100 | B cell proliferation | 4/261 | 99/18723 | 0.049777834 | 0.162332255 | 0.118478338 | CD180/FCGR2B/TLR4/TYROBP | 4 |
| GO:0051153 | regulation of striated muscle cell differentiation | 4/261 | 99/18723 | 0.049777834 | 0.162332255 | 0.118478338 | CYP26B1/HDAC9/RBM24/XBP1 | 4 |
| GO:0110149 | regulation of biomineralization | 4/261 | 99/18723 | 0.049777834 | 0.162332255 | 0.118478338 | ECM1/GAS6/RFLNB/SRGN | 4 |

- **Supplementary Table S9. GO Cellular Component (CC) enrichment of PSPC1-specific DEGs.**

Enriched CC terms unique to PSPC1 knockdown.

| **ID** | **Description** | **GeneRatio** | **BgRatio** | **pvalue** | **p.adjust** | **qvalue** | **geneID** | **Count** |
| --- | --- | --- | --- | --- | --- | --- | --- | --- |
| GO:0034774 | secretory granule lumen | 26/264 | 322/19550 | 2.9481E-13 | 4.4391E-11 | 3.65242E-11 | A2M/ADA2/ANXA2/AZU1/BPI/C3/CHI3L1/ECM1/FCN1/FUCA1/GAS6/GM2A/GNS/GRN/GSN/LGALS3BP/LYZ/PRSS57/QSOX1/RNASE3/S100P/SERPINI1/SLPI/SRGN/TIMP1/VEGFA | 26 |
| GO:0060205 | cytoplasmic vesicle lumen | 26/264 | 325/19550 | 3.65597E-13 | 4.4391E-11 | 3.65242E-11 | A2M/ADA2/ANXA2/AZU1/BPI/C3/CHI3L1/ECM1/FCN1/FUCA1/GAS6/GM2A/GNS/GRN/GSN/LGALS3BP/LYZ/PRSS57/QSOX1/RNASE3/S100P/SERPINI1/SLPI/SRGN/TIMP1/VEGFA | 26 |
| GO:0031983 | vesicle lumen | 26/264 | 327/19550 | 4.21433E-13 | 4.4391E-11 | 3.65242E-11 | A2M/ADA2/ANXA2/AZU1/BPI/C3/CHI3L1/ECM1/FCN1/FUCA1/GAS6/GM2A/GNS/GRN/GSN/LGALS3BP/LYZ/PRSS57/QSOX1/RNASE3/S100P/SERPINI1/SLPI/SRGN/TIMP1/VEGFA | 26 |
| GO:0005766 | primary lysosome | 17/264 | 155/19550 | 3.63894E-11 | 2.29981E-09 | 1.89225E-09 | ACP3/ADA2/ANXA2/AZU1/BPI/C3/CKAP4/FUCA1/GM2A/GNS/GRN/LYZ/PRSS57/RAB37/RAP1B/RNASE3/SLCO4C1 | 17 |
| GO:0042582 | azurophil granule | 17/264 | 155/19550 | 3.63894E-11 | 2.29981E-09 | 1.89225E-09 | ACP3/ADA2/ANXA2/AZU1/BPI/C3/CKAP4/FUCA1/GM2A/GNS/GRN/LYZ/PRSS57/RAB37/RAP1B/RNASE3/SLCO4C1 | 17 |
| GO:0005775 | vacuolar lumen | 17/264 | 174/19550 | 2.29118E-10 | 1.20669E-08 | 9.92844E-09 | ADA2/ANXA2/ASAH1/AZU1/BPI/C3/FUCA1/GM2A/GNS/GPC3/GRN/HSPG2/LYZ/PRSS57/RNASE3/SDC2/TPP1 | 17 |
| GO:0035578 | azurophil granule lumen | 12/264 | 91/19550 | 3.5098E-09 | 1.58442E-07 | 1.30364E-07 | ADA2/ANXA2/AZU1/BPI/C3/FUCA1/GM2A/GNS/GRN/LYZ/PRSS57/RNASE3 | 12 |
| GO:0042581 | specific granule | 14/264 | 160/19550 | 3.78273E-08 | 1.49418E-06 | 1.22939E-06 | ALDH3B1/BPI/CD36/CHI3L1/CKAP4/CLEC12A/CYBB/ITGB2/LYZ/QSOX1/RAB37/SLC15A4/SLCO4C1/SLPI | 14 |
| GO:0030667 | secretory granule membrane | 19/264 | 311/19550 | 5.06387E-08 | 1.77798E-06 | 1.4629E-06 | ACP3/ALDH3B1/ATP6V0A1/AZU1/CD109/CD36/CKAP4/CLEC12A/CYBB/FCGR2A/IQGAP2/ITGB2/LILRB2/PTAFR/RAB37/RAP1B/SLC15A4/SLCO4C1/TYROBP | 19 |
| GO:0062023 | collagen-containing extracellular matrix | 22/264 | 425/19550 | 8.34558E-08 | 2.6372E-06 | 2.16985E-06 | A2M/ADAMDEC1/ADAMTS10/ADAMTS5/ANXA2/ANXA4/COL9A2/COLQ/ECM1/EGFL7/F3/FCN1/GPC3/HSPG2/LGALS3BP/MFAP4/MMP2/MMP9/SDC2/SLPI/THBS4/TIMP1 | 22 |
| GO:0070820 | tertiary granule | 12/264 | 164/19550 | 2.43636E-06 | 6.99899E-05 | 5.75866E-05 | ASAH1/ATP6V0A1/CLEC12A/CST3/CYBB/FTH1/ITGB2/LILRB2/LYZ/MMP9/PTAFR/QSOX1 | 12 |
| GO:0008305 | integrin complex | 6/264 | 31/19550 | 3.17697E-06 | 8.36603E-05 | 6.88344E-05 | ITGA4/ITGA5/ITGA6/ITGB2/ITGB7/TSPAN32 | 6 |
| GO:0035579 | specific granule membrane | 9/264 | 91/19550 | 3.88942E-06 | 9.45429E-05 | 7.77884E-05 | ALDH3B1/CD36/CKAP4/CLEC12A/CYBB/ITGB2/RAB37/SLC15A4/SLCO4C1 | 9 |
| GO:0098636 | protein complex involved in cell adhesion | 6/264 | 36/19550 | 7.94213E-06 | 0.000179265 | 0.000147497 | ITGA4/ITGA5/ITGA6/ITGB2/ITGB7/TSPAN32 | 6 |
| GO:0043020 | NADPH oxidase complex | 4/264 | 14/19550 | 2.92485E-05 | 0.000597142 | 0.000491319 | CYBB/NCF1/NCF1B/NCF1C | 4 |
| GO:0001726 | ruffle | 11/264 | 178/19550 | 3.15069E-05 | 0.000597142 | 0.000491319 | ANXA2/CTTN/FAP/ITGA5/KSR1/MYO10/RASGRP2/SAMSN1/SRC/TLR4/TWF1 | 11 |
| GO:0031091 | platelet alpha granule | 8/264 | 91/19550 | 3.21247E-05 | 0.000597142 | 0.000491319 | A2M/CD109/CD36/GAS6/QSOX1/SRGN/TIMP1/VEGFA | 8 |
| GO:0043202 | lysosomal lumen | 8/264 | 97/19550 | 5.09563E-05 | 0.000894566 | 0.000736035 | ASAH1/FUCA1/GM2A/GNS/GPC3/HSPG2/SDC2/TPP1 | 8 |
| GO:1904724 | tertiary granule lumen | 6/264 | 55/19550 | 9.53876E-05 | 0.001512002 | 0.001244053 | ASAH1/CST3/FTH1/LYZ/MMP9/QSOX1 | 6 |
| GO:0005765 | lysosomal membrane | 16/264 | 395/19550 | 0.000100481 | 0.001512002 | 0.001244053 | ACP3/ANXA2/ATP6V0A1/ATP6V1C2/AZU1/CD1D/CKAP4/GNA11/GRN/HPS4/RAB37/RAP1B/SLC15A4/SLC7A5/SLCO4C1/SORT1 | 16 |
| GO:0098852 | lytic vacuole membrane | 16/264 | 395/19550 | 0.000100481 | 0.001512002 | 0.001244053 | ACP3/ANXA2/ATP6V0A1/ATP6V1C2/AZU1/CD1D/CKAP4/GNA11/GRN/HPS4/RAB37/RAP1B/SLC15A4/SLC7A5/SLCO4C1/SORT1 | 16 |
| GO:0035577 | azurophil granule membrane | 6/264 | 58/19550 | 0.000128756 | 0.001849406 | 0.001521664 | ACP3/AZU1/CKAP4/RAB37/RAP1B/SLCO4C1 | 6 |
| GO:0044853 | plasma membrane raft | 8/264 | 116/19550 | 0.00017977 | 0.002469887 | 0.002032186 | CD36/HMOX1/ITGB2/LRRK2/PTCH1/SRC/TGFBR2/TREM2 | 8 |
| GO:0005925 | focal adhesion | 16/264 | 418/19550 | 0.000191915 | 0.002526878 | 0.002079077 | CDC42EP1/CTTN/DIXDC1/FAP/FGFR3/GSN/HSPG2/ITGA4/ITGA5/ITGA6/ITGB2/ITGB7/SRC/SYNPO2/TNS1/TWF1 | 16 |
| GO:0101002 | ficolin-1-rich granule | 10/264 | 185/19550 | 0.000217136 | 0.002744603 | 0.002258218 | ASAH1/ATP6V0A1/CST3/FCN1/FTH1/GNS/GSN/ITGB2/LILRB2/MMP9 | 10 |
| GO:0030055 | cell-substrate junction | 16/264 | 425/19550 | 0.000231314 | 0.00281136 | 0.002313145 | CDC42EP1/CTTN/DIXDC1/FAP/FGFR3/GSN/HSPG2/ITGA4/ITGA5/ITGA6/ITGB2/ITGB7/SRC/SYNPO2/TNS1/TWF1 | 16 |
| GO:0031093 | platelet alpha granule lumen | 6/264 | 67/19550 | 0.000286903 | 0.003357831 | 0.002762772 | A2M/GAS6/QSOX1/SRGN/TIMP1/VEGFA | 6 |
| GO:0005788 | endoplasmic reticulum lumen | 13/264 | 313/19550 | 0.000352991 | 0.00398376 | 0.003277777 | ADAMTS5/C3/CKAP4/COL9A2/CST3/FAM20C/GAS6/GPC3/QSOX1/SDC2/STC2/STS/TIMP1 | 13 |
| GO:0005774 | vacuolar membrane | 16/264 | 444/19550 | 0.000375565 | 0.004092365 | 0.003367135 | ACP3/ANXA2/ATP6V0A1/ATP6V1C2/AZU1/CD1D/CKAP4/GNA11/GRN/HPS4/RAB37/RAP1B/SLC15A4/SLC7A5/SLCO4C1/SORT1 | 16 |
| GO:0005901 | caveola | 6/264 | 84/19550 | 0.000965817 | 0.010173273 | 0.008370414 | CD36/HMOX1/LRRK2/PTCH1/SRC/TGFBR2 | 6 |
| GO:0005767 | secondary lysosome | 3/264 | 17/19550 | 0.001438987 | 0.014304315 | 0.011769373 | FTH1/LRRK2/NCF1 | 3 |
| GO:1904813 | ficolin-1-rich granule lumen | 7/264 | 124/19550 | 0.001488568 | 0.014304315 | 0.011769373 | ASAH1/CST3/FCN1/FTH1/GNS/GSN/MMP9 | 7 |
| GO:0035580 | specific granule lumen | 5/264 | 62/19550 | 0.001493805 | 0.014304315 | 0.011769373 | BPI/CHI3L1/LYZ/QSOX1/SLPI | 5 |
| GO:0009897 | external side of plasma membrane | 14/264 | 421/19550 | 0.001842455 | 0.017123991 | 0.01408936 | CD1D/CD36/CD86/CXCL10/F3/FCER2/FCGR2B/FCN1/ITGB2/KIT/PRLR/SLC7A5/TGFBR2/TLR4 | 14 |
| GO:0032587 | ruffle membrane | 6/264 | 99/19550 | 0.002251809 | 0.020330615 | 0.016727721 | FAP/ITGA5/KSR1/RASGRP2/SRC/TWF1 | 6 |
| GO:0042827 | platelet dense granule | 3/264 | 21/19550 | 0.002704526 | 0.023302618 | 0.01917304 | ECM1/HPS4/LGALS3BP | 3 |
| GO:0031253 | cell projection membrane | 12/264 | 346/19550 | 0.002728471 | 0.023302618 | 0.01917304 | CD36/DDN/FAP/ITGA5/KSR1/MYO10/PTCH1/RASGRP2/S100P/SLC7A5/SRC/TWF1 | 12 |
| GO:0070821 | tertiary granule membrane | 5/264 | 73/19550 | 0.003075718 | 0.025577024 | 0.021044387 | CLEC12A/CYBB/ITGB2/LILRB2/PTAFR | 5 |
| GO:0098858 | actin-based cell projection | 9/264 | 221/19550 | 0.003206861 | 0.0259838 | 0.021379076 | ACP3/CA2/IQGAP2/ITGA6/LRRK2/MYO10/S100P/SLC7A5/TWF1 | 9 |
| GO:0031252 | cell leading edge | 13/264 | 422/19550 | 0.005043141 | 0.039840814 | 0.032780417 | ANXA2/CTTN/DDN/FAP/IQGAP2/ITGA5/KSR1/MYO10/RASGRP2/SAMSN1/SRC/TLR4/TWF1 | 13 |
| GO:0098562 | cytoplasmic side of membrane | 8/264 | 197/19550 | 0.005435143 | 0.041890374 | 0.034466764 | CNR2/GM2A/GNA11/KIT/LILRB4/LRRK2/PTPN22/SRC | 8 |
| GO:0045121 | membrane raft | 11/264 | 335/19550 | 0.006052597 | 0.04447955 | 0.036597098 | ANXA2/CD36/FURIN/HMOX1/ITGB2/LRRK2/PTCH1/SRC/TGFBR2/TPP1/TREM2 | 11 |
| GO:0098857 | membrane microdomain | 11/264 | 335/19550 | 0.006052597 | 0.04447955 | 0.036597098 | ANXA2/CD36/FURIN/HMOX1/ITGB2/LRRK2/PTCH1/SRC/TGFBR2/TPP1/TREM2 | 11 |
| GO:0002102 | podosome | 3/264 | 29/19550 | 0.006863267 | 0.049290738 | 0.040555671 | CTTN/GSN/SRC | 3 |
| GO:0005902 | microvillus | 5/264 | 91/19550 | 0.007834324 | 0.055014361 | 0.045264981 | CA2/IQGAP2/LRRK2/S100P/SLC7A5 | 5 |
| GO:0009898 | cytoplasmic side of plasma membrane | 7/264 | 172/19550 | 0.008979804 | 0.06168735 | 0.050755415 | CNR2/GM2A/GNA11/KIT/LILRB4/PTPN22/SRC | 7 |
| GO:0044754 | autolysosome | 2/264 | 11/19550 | 0.009219841 | 0.061988719 | 0.051003376 | FTH1/LRRK2 | 2 |
| GO:0005604 | basement membrane | 5/264 | 96/19550 | 0.009755969 | 0.064226795 | 0.052844831 | ANXA2/COLQ/HSPG2/THBS4/TIMP1 | 5 |
| GO:0030140 | trans-Golgi network transport vesicle | 3/264 | 34/19550 | 0.010697982 | 0.068991069 | 0.056764803 | FURIN/NCALD/SORT1 | 3 |
| GO:0001527 | microfibril | 2/264 | 12/19550 | 0.010965928 | 0.069304663 | 0.057022824 | ADAMTS10/MFAP4 | 2 |
| GO:0031256 | leading edge membrane | 7/264 | 180/19550 | 0.011347408 | 0.070309428 | 0.057849529 | DDN/FAP/ITGA5/KSR1/RASGRP2/SRC/TWF1 | 7 |
| GO:0045178 | basal part of cell | 9/264 | 276/19550 | 0.013010102 | 0.078880472 | 0.064901654 | ANXA2/CA2/CD1D/FAP/GM2A/P2RY6/SLC29A1/SLC7A5/SLCO4C1 | 9 |
| GO:0005796 | Golgi lumen | 5/264 | 104/19550 | 0.013466043 | 0.078880472 | 0.064901654 | FURIN/GAS6/GPC3/HSPG2/SDC2 | 5 |
| GO:0016323 | basolateral plasma membrane | 8/264 | 231/19550 | 0.013479574 | 0.078880472 | 0.064901654 | ANXA2/CA2/CD1D/GM2A/P2RY6/SLC29A1/SLC7A5/SLCO4C1 | 8 |
| GO:0072562 | blood microparticle | 6/264 | 146/19550 | 0.014475465 | 0.083168126 | 0.06842947 | A2M/C3/CFH/GSN/HSPA7/LGALS3BP | 6 |
| GO:0031089 | platelet dense granule lumen | 2/264 | 14/19550 | 0.014853789 | 0.083560755 | 0.06875252 | ECM1/LGALS3BP | 2 |
| GO:0030175 | filopodium | 5/264 | 107/19550 | 0.015072668 | 0.083560755 | 0.06875252 | ACP3/IQGAP2/ITGA6/MYO10/TWF1 | 5 |
| GO:0042470 | melanosome | 5/264 | 109/19550 | 0.016212142 | 0.086831134 | 0.071443338 | ANXA2/ATP6V0A1/HPS4/PRDX1/TPP1 | 5 |
| GO:0048770 | pigment granule | 5/264 | 109/19550 | 0.016212142 | 0.086831134 | 0.071443338 | ANXA2/ATP6V0A1/HPS4/PRDX1/TPP1 | 5 |
| GO:0031092 | platelet alpha granule membrane | 2/264 | 17/19550 | 0.021617681 | 0.113853119 | 0.093676617 | CD109/CD36 | 2 |
| GO:0016528 | sarcoplasm | 4/264 | 79/19550 | 0.022182425 | 0.114912234 | 0.094548041 | CAMK2D/GSN/RASD1/THBS4 | 4 |
| GO:0009925 | basal plasma membrane | 8/264 | 257/19550 | 0.023875868 | 0.121689908 | 0.100124608 | ANXA2/CA2/CD1D/GM2A/P2RY6/SLC29A1/SLC7A5/SLCO4C1 | 8 |
| GO:0099091 | postsynaptic specialization, intracellular component | 2/264 | 19/19550 | 0.026705728 | 0.133952539 | 0.110214114 | SRC/SRGN | 2 |
| GO:0016471 | vacuolar proton-transporting V-type ATPase complex | 2/264 | 20/19550 | 0.02941288 | 0.145226096 | 0.119489826 | ATP6V0A1/ATP6V1C2 | 2 |
| GO:0030427 | site of polarized growth | 6/264 | 183/19550 | 0.038252032 | 0.18405592 | 0.151438415 | CTTN/FRY/ITGA4/LRRK2/PTCH1/TMOD2 | 6 |
| GO:0045335 | phagocytic vesicle | 5/264 | 137/19550 | 0.038442059 | 0.18405592 | 0.151438415 | ATP6V0A1/CD36/CYBB/NCF1/TRIM14 | 5 |
| GO:0005798 | Golgi-associated vesicle | 4/264 | 96/19550 | 0.04117096 | 0.194179453 | 0.159767904 | FURIN/LRRK2/NCALD/SORT1 | 4 |
| GO:0033176 | proton-transporting V-type ATPase complex | 2/264 | 26/19550 | 0.047732482 | 0.221815652 | 0.182506549 | ATP6V0A1/ATP6V1C2 | 2 |
| GO:0101003 | ficolin-1-rich granule membrane | 3/264 | 61/19550 | 0.049427641 | 0.226364268 | 0.186249081 | ATP6V0A1/ITGB2/LILRB2 | 3 |

- **Supplementary Table S10. GO Molecular Function (MF) enrichment of PSPC1-specific DEGs.**

Enriched MF terms unique to PSPC1 knockdown.

| **ID** | **Description** | **GeneRatio** | **BgRatio** | **pvalue** | **p.adjust** | **qvalue** | **geneID** | **Count** |
| --- | --- | --- | --- | --- | --- | --- | --- | --- |
| GO:0030414 | peptidase inhibitor activity | 13/263 | 187/18368 | 3.10395E-06 | 0.001158354 | 0.001000358 | A2M/ANXA2/C3/CD109/CST3/CSTA/FURIN/GAS6/GPC3/SERPINI1/SLPI/SPOCK2/TIMP1 | 13 |
| GO:0005539 | glycosaminoglycan binding | 14/263 | 230/18368 | 6.11807E-06 | 0.001158354 | 0.001000358 | ADA2/ADAMTS5/AZU1/CFH/COLQ/CXCL10/GNS/PRSS57/PTCH1/SPOCK2/TGFBR2/THBS4/TREM2/VEGFA | 14 |
| GO:0061134 | peptidase regulator activity | 14/263 | 230/18368 | 6.11807E-06 | 0.001158354 | 0.001000358 | A2M/ANXA2/C3/CD109/CST3/CSTA/FURIN/GAS6/GPC3/NLRP12/SERPINI1/SLPI/SPOCK2/TIMP1 | 14 |
| GO:0004866 | endopeptidase inhibitor activity | 12/263 | 180/18368 | 1.1421E-05 | 0.00162178 | 0.001400574 | A2M/ANXA2/C3/CD109/CST3/CSTA/FURIN/GAS6/SERPINI1/SLPI/SPOCK2/TIMP1 | 12 |
| GO:0061135 | endopeptidase regulator activity | 12/263 | 194/18368 | 2.41733E-05 | 0.002195813 | 0.00189631 | A2M/ANXA2/C3/CD109/CST3/CSTA/FURIN/GAS6/SERPINI1/SLPI/SPOCK2/TIMP1 | 12 |
| GO:0002020 | protease binding | 10/263 | 135/18368 | 2.50276E-05 | 0.002195813 | 0.00189631 | A2M/ANXA2/CST3/CSTA/ECM1/F3/FAP/FURIN/KIT/TIMP1 | 10 |
| GO:0001540 | amyloid-beta binding | 8/263 | 84/18368 | 2.70611E-05 | 0.002195813 | 0.00189631 | CD36/CST3/FCGR2B/HSPG2/ITGB2/LILRB2/TLR4/TREM2 | 8 |
| GO:0019955 | cytokine binding | 10/263 | 139/18368 | 3.22027E-05 | 0.002286395 | 0.001974537 | A2M/CD109/CD36/CSF1R/ITGA4/KIT/NOG/PRLR/TGFBR2/TRIM16 | 10 |
| GO:0019838 | growth factor binding | 10/263 | 141/18368 | 3.64062E-05 | 0.002297636 | 0.001984245 | A2M/CD109/CD36/FGFR3/FURIN/IGFBP2/ITGA6/SORT1/TGFBR2/TRIM16 | 10 |
| GO:0001530 | lipopolysaccharide binding | 5/263 | 33/18368 | 9.90439E-05 | 0.005625696 | 0.004858366 | BPI/PTAFR/RNASE3/TLR4/TREM2 | 5 |
| GO:0008201 | heparin binding | 10/263 | 166/18368 | 0.000143348 | 0.006866143 | 0.00592962 | ADA2/ADAMTS5/AZU1/CFH/COLQ/CXCL10/PRSS57/PTCH1/THBS4/VEGFA | 10 |
| GO:0050840 | extracellular matrix binding | 6/263 | 56/18368 | 0.000145059 | 0.006866143 | 0.00592962 | ADAMTS5/ANXA2/ECM1/ITGA6/SPOCK2/VEGFA | 6 |
| GO:0005178 | integrin binding | 9/263 | 144/18368 | 0.000233559 | 0.010204743 | 0.008812843 | ADAMTS5/FAP/FCER2/ITGA4/ITGB2/ITGB7/JAML/SRC/THBS4 | 9 |
| GO:0019865 | immunoglobulin binding | 4/263 | 23/18368 | 0.000293557 | 0.010801338 | 0.009328065 | FCER2/FCGR2A/FCGR2B/FCGR2C | 4 |
| GO:0030215 | semaphorin receptor binding | 4/263 | 23/18368 | 0.000293557 | 0.010801338 | 0.009328065 | SEMA4A/SEMA4C/SEMA6A/SEMA6B | 4 |
| GO:0015173 | aromatic amino acid transmembrane transporter activity | 3/263 | 10/18368 | 0.000323279 | 0.010801338 | 0.009328065 | SLC15A4/SLC38A5/SLC7A5 | 3 |
| GO:0016176 | superoxide-generating NADPH oxidase activator activity | 3/263 | 10/18368 | 0.000323279 | 0.010801338 | 0.009328065 | NCF1/NCF1B/NCF1C | 3 |
| GO:0019864 | IgG binding | 3/263 | 11/18368 | 0.000439812 | 0.013878508 | 0.011985517 | FCGR2A/FCGR2B/FCGR2C | 3 |
| GO:0038187 | pattern recognition receptor activity | 4/263 | 26/18368 | 0.000479174 | 0.014324783 | 0.012370921 | CD36/FCN1/PTAFR/TLR4 | 4 |
| GO:0045499 | chemorepellent activity | 4/263 | 27/18368 | 0.000556224 | 0.015796758 | 0.013642123 | SEMA4A/SEMA4C/SEMA6A/SEMA6B | 4 |
| GO:0042277 | peptide binding | 13/263 | 321/18368 | 0.000768853 | 0.020795656 | 0.017959184 | CD1D/CD36/CST3/FCGR2B/FURIN/HSPG2/ITGB2/LILRB2/PRLR/SLC7A5/TLR4/TPP1/TREM2 | 13 |
| GO:0008047 | enzyme activator activity | 7/263 | 110/18368 | 0.00103528 | 0.026729052 | 0.023083281 | ALOX5AP/CCL5/GM2A/NCF1/NCF1B/NCF1C/SRC | 7 |
| GO:0004029 | aldehyde dehydrogenase (NAD+) activity | 3/263 | 15/18368 | 0.001162441 | 0.028707233 | 0.024791643 | ALDH1L2/ALDH2/ALDH3B1 | 3 |
| GO:0004030 | aldehyde dehydrogenase [NAD(P)+] activity | 3/263 | 16/18368 | 0.00141563 | 0.032163103 | 0.027776141 | ALDH1L2/ALDH2/ALDH3B1 | 3 |
| GO:0016004 | phospholipase activator activity | 3/263 | 16/18368 | 0.00141563 | 0.032163103 | 0.027776141 | CCL5/GM2A/SRC | 3 |
| GO:0033218 | amide binding | 14/263 | 400/18368 | 0.001961781 | 0.042486439 | 0.036691402 | CD1D/CD300LF/CD36/CST3/FCGR2B/FURIN/HSPG2/ITGB2/LILRB2/PRLR/SLC7A5/TLR4/TPP1/TREM2 | 14 |
| GO:0060229 | lipase activator activity | 3/263 | 18/18368 | 0.002019602 | 0.042486439 | 0.036691402 | CCL5/GM2A/SRC | 3 |
| GO:0004867 | serine-type endopeptidase inhibitor activity | 6/263 | 98/18368 | 0.00285617 | 0.057939457 | 0.05003667 | A2M/ANXA2/CD109/FURIN/SERPINI1/SLPI | 6 |
| GO:0005543 | phospholipid binding | 15/263 | 466/18368 | 0.003056733 | 0.059869796 | 0.051703716 | ANXA2/ANXA4/CD300LF/F3/GAS6/GSN/IQGAP2/MYO10/NCF1/NCF1B/NCF1C/PTAFR/TPP1/TREM2/TWF1 | 15 |
| GO:0004252 | serine-type endopeptidase activity | 8/263 | 174/18368 | 0.003656411 | 0.069228047 | 0.059785526 | AZU1/F3/FAP/FURIN/MMP2/MMP9/PRSS57/TPP1 | 8 |
| GO:0048018 | receptor ligand activity | 15/263 | 487/18368 | 0.004589038 | 0.083465089 | 0.072080674 | ADA2/ADM2/CCL5/CXCL10/GAL/GAS6/GRN/SEMA4A/SEMA4C/SEMA6A/SEMA6B/STC2/THBS4/TIMP1/VEGFA | 15 |
| GO:0050431 | transforming growth factor beta binding | 3/263 | 24/18368 | 0.004702259 | 0.083465089 | 0.072080674 | CD109/CD36/TGFBR2 | 3 |
| GO:1901681 | sulfur compound binding | 10/263 | 265/18368 | 0.005014613 | 0.086282413 | 0.074513722 | ADA2/ADAMTS5/AZU1/CFH/COLQ/CXCL10/PRSS57/PTCH1/THBS4/VEGFA | 10 |
| GO:0004222 | metalloendopeptidase activity | 6/263 | 111/18368 | 0.005271028 | 0.086282413 | 0.074513722 | ADAM28/ADAMDEC1/ADAMTS10/ADAMTS5/MMP2/MMP9 | 6 |
| GO:0030546 | signaling receptor activator activity | 15/263 | 495/18368 | 0.005316698 | 0.086282413 | 0.074513722 | ADA2/ADM2/CCL5/CXCL10/GAL/GAS6/GRN/SEMA4A/SEMA4C/SEMA6A/SEMA6B/STC2/THBS4/TIMP1/VEGFA | 15 |
| GO:0140677 | molecular function activator activity | 7/263 | 150/18368 | 0.005919842 | 0.093401951 | 0.080662174 | ALOX5AP/CCL5/GM2A/NCF1/NCF1B/NCF1C/SRC | 7 |
| GO:0008236 | serine-type peptidase activity | 8/263 | 191/18368 | 0.006366318 | 0.097731583 | 0.084401256 | AZU1/F3/FAP/FURIN/MMP2/MMP9/PRSS57/TPP1 | 8 |
| GO:0017171 | serine hydrolase activity | 8/263 | 195/18368 | 0.007181865 | 0.107349978 | 0.092707728 | AZU1/F3/FAP/FURIN/MMP2/MMP9/PRSS57/TPP1 | 8 |
| GO:0015643 | toxic substance binding | 2/263 | 10/18368 | 0.008519837 | 0.118030916 | 0.101931814 | ASS1/AZU1 | 2 |
| GO:0016175 | superoxide-generating NAD(P)H oxidase activity | 2/263 | 10/18368 | 0.008519837 | 0.118030916 | 0.101931814 | CYBB/NCF1 | 2 |
| GO:0016717 | oxidoreductase activity, acting on paired donors, with oxidation of a pair of donors resulting in the reduction of molecular oxygen to two molecules of water | 2/263 | 10/18368 | 0.008519837 | 0.118030916 | 0.101931814 | FADS2/SCD | 2 |
| GO:0001664 | G protein-coupled receptor binding | 10/263 | 295/18368 | 0.010314786 | 0.133162416 | 0.114999418 | ADA2/C3/CCL5/CXCL10/DNM1/FCN1/GAL/GNA11/HOMER3/PTCH1 | 10 |
| GO:0008603 | cAMP-dependent protein kinase regulator activity | 2/263 | 11/18368 | 0.010315398 | 0.133162416 | 0.114999418 | CXCL10/PKIB | 2 |
| GO:1901474 | azole transmembrane transporter activity | 2/263 | 11/18368 | 0.010315398 | 0.133162416 | 0.114999418 | SLC15A4/SLC38A5 | 2 |
| GO:0001786 | phosphatidylserine binding | 4/263 | 60/18368 | 0.01069928 | 0.135048692 | 0.116628411 | ANXA2/CD300LF/GAS6/TREM2 | 4 |
| GO:0003779 | actin binding | 13/263 | 441/18368 | 0.01136009 | 0.140272419 | 0.121139635 | CAP2/CTTN/DIXDC1/GSN/IQGAP2/KLHL4/LRRK2/MYO10/NCALD/SYNPO2/TMOD2/TNS1/TWF1 | 13 |
| GO:0004859 | phospholipase inhibitor activity | 2/263 | 13/18368 | 0.014356271 | 0.15973642 | 0.137948799 | ANXA2/ANXA4 | 2 |
| GO:0008035 | high-density lipoprotein particle binding | 2/263 | 13/18368 | 0.014356271 | 0.15973642 | 0.137948799 | CD36/TREM2 | 2 |
| GO:0031994 | insulin-like growth factor I binding | 2/263 | 13/18368 | 0.014356271 | 0.15973642 | 0.137948799 | IGFBP2/ITGA6 | 2 |
| GO:0043394 | proteoglycan binding | 3/263 | 36/18368 | 0.014628298 | 0.15973642 | 0.137948799 | ADA2/AZU1/CFH | 3 |
| GO:0020037 | heme binding | 6/263 | 139/18368 | 0.01505806 | 0.15973642 | 0.137948799 | CYBB/CYP26B1/HMOX1/PTGS1/SRC/STC2 | 6 |
| GO:0005085 | guanyl-nucleotide exchange factor activity | 8/263 | 223/18368 | 0.015271361 | 0.15973642 | 0.137948799 | ARHGEF40/DOCK9/HPS4/IQSEC3/NUCB2/RASGRP2/RASGRP4/RCBTB2 | 8 |
| GO:0016620 | oxidoreductase activity, acting on the aldehyde or oxo group of donors, NAD or NADP as acceptor | 3/263 | 37/18368 | 0.015753915 | 0.15973642 | 0.137948799 | ALDH1L2/ALDH2/ALDH3B1 | 3 |
| GO:0042056 | chemoattractant activity | 3/263 | 37/18368 | 0.015753915 | 0.15973642 | 0.137948799 | CCL5/CXCL10/VEGFA | 3 |
| GO:0005096 | GTPase activator activity | 13/263 | 462/18368 | 0.01614372 | 0.15973642 | 0.137948799 | ARHGAP11A/ARHGEF40/DOCK9/HPS4/IQGAP2/IQSEC3/JUN/LRRK2/NUCB2/RASGRP2/RASGRP4/RCBTB2/TBC1D4 | 13 |
| GO:0005172 | vascular endothelial growth factor receptor binding | 2/263 | 14/18368 | 0.016592339 | 0.15973642 | 0.137948799 | ITGA5/VEGFA | 2 |
| GO:0019992 | diacylglycerol binding | 2/263 | 14/18368 | 0.016592339 | 0.15973642 | 0.137948799 | RASGRP2/RASGRP4 | 2 |
| GO:0031005 | filamin binding | 2/263 | 14/18368 | 0.016592339 | 0.15973642 | 0.137948799 | RFLNB/SYNPO2 | 2 |
| GO:0045028 | G protein-coupled purinergic nucleotide receptor activity | 2/263 | 14/18368 | 0.016592339 | 0.15973642 | 0.137948799 | P2RY6/PTAFR | 2 |
| GO:0019199 | transmembrane receptor protein kinase activity | 6/263 | 143/18368 | 0.017091896 | 0.161803285 | 0.139733748 | CSF1R/EPHB4/FGFR3/KIT/SRC/TGFBR2 | 6 |
| GO:0004857 | enzyme inhibitor activity | 4/263 | 70/18368 | 0.018017977 | 0.167773952 | 0.144890032 | ANXA2/ANXA4/FRY/LRRK2 | 4 |
| GO:0005161 | platelet-derived growth factor receptor binding | 2/263 | 15/18368 | 0.01896617 | 0.169037564 | 0.145981291 | ITGA5/VEGFA | 2 |
| GO:0050664 | oxidoreductase activity, acting on NAD(P)H, oxygen as acceptor | 2/263 | 15/18368 | 0.01896617 | 0.169037564 | 0.145981291 | CYBB/NCF1 | 2 |
| GO:0008022 | protein C-terminus binding | 7/263 | 189/18368 | 0.019334217 | 0.169037564 | 0.145981291 | DNM1/ECM1/HSPG2/KSR1/SRC/TERT/TOP2A | 7 |
| GO:0019207 | kinase regulator activity | 8/263 | 233/18368 | 0.019344087 | 0.169037564 | 0.145981291 | CCL5/CXCL10/GAS6/LILRB4/PIK3R3/PKIB/TREM2/TRIB3 | 8 |
| GO:0046906 | tetrapyrrole binding | 6/263 | 149/18368 | 0.020485351 | 0.176298169 | 0.152251569 | CYBB/CYP26B1/HMOX1/PTGS1/SRC/STC2 | 6 |
| GO:0008191 | metalloendopeptidase inhibitor activity | 2/263 | 16/18368 | 0.02147339 | 0.180127613 | 0.155558687 | SPOCK2/TIMP1 | 2 |
| GO:0004175 | endopeptidase activity | 12/263 | 430/18368 | 0.021564573 | 0.180127613 | 0.155558687 | ADAM28/ADAMDEC1/ADAMTS10/ADAMTS5/AZU1/F3/FAP/FURIN/MMP2/MMP9/PRSS57/TPP1 | 12 |
| GO:0030695 | GTPase regulator activity | 13/263 | 488/18368 | 0.024032876 | 0.185058419 | 0.159816944 | ARHGAP11A/ARHGEF40/DOCK9/HPS4/IQGAP2/IQSEC3/JUN/LRRK2/NUCB2/RASGRP2/RASGRP4/RCBTB2/TBC1D4 | 13 |
| GO:0060589 | nucleoside-triphosphatase regulator activity | 13/263 | 488/18368 | 0.024032876 | 0.185058419 | 0.159816944 | ARHGAP11A/ARHGEF40/DOCK9/HPS4/IQGAP2/IQSEC3/JUN/LRRK2/NUCB2/RASGRP2/RASGRP4/RCBTB2/TBC1D4 | 13 |
| GO:0008484 | sulfuric ester hydrolase activity | 2/263 | 17/18368 | 0.024109724 | 0.185058419 | 0.159816944 | GNS/STS | 2 |
| GO:0015174 | basic amino acid transmembrane transporter activity | 2/263 | 17/18368 | 0.024109724 | 0.185058419 | 0.159816944 | SLC15A4/SLC38A5 | 2 |
| GO:0030169 | low-density lipoprotein particle binding | 2/263 | 17/18368 | 0.024109724 | 0.185058419 | 0.159816944 | CD36/TREM2 | 2 |
| GO:0043395 | heparan sulfate proteoglycan binding | 2/263 | 17/18368 | 0.024109724 | 0.185058419 | 0.159816944 | AZU1/CFH | 2 |
| GO:0016903 | oxidoreductase activity, acting on the aldehyde or oxo group of donors | 3/263 | 44/18368 | 0.024968142 | 0.189092064 | 0.163300411 | ALDH1L2/ALDH2/ALDH3B1 | 3 |
| GO:0055102 | lipase inhibitor activity | 2/263 | 18/18368 | 0.026870989 | 0.198217164 | 0.171180872 | ANXA2/ANXA4 | 2 |
| GO:0098641 | cadherin binding involved in cell-cell adhesion | 2/263 | 18/18368 | 0.026870989 | 0.198217164 | 0.171180872 | ANXA2/CDC42EP1 | 2 |
| GO:0030296 | protein tyrosine kinase activator activity | 2/263 | 20/18368 | 0.032752048 | 0.237400175 | 0.205019424 | CCL5/GAS6 | 2 |
| GO:0004714 | transmembrane receptor protein tyrosine kinase activity | 5/263 | 124/18368 | 0.033018686 | 0.237400175 | 0.205019424 | CSF1R/EPHB4/FGFR3/KIT/SRC | 5 |
| GO:0016810 | hydrolase activity, acting on carbon-nitrogen (but not peptide) bonds | 5/263 | 125/18368 | 0.034008444 | 0.241459954 | 0.20852546 | ADA2/ASAH1/HDAC9/NDST1/PADI4 | 5 |
| GO:0009055 | electron transfer activity | 5/263 | 126/18368 | 0.035016461 | 0.245430293 | 0.211954256 | ALDH2/CYBB/ME1/NCF1/PHGDH | 5 |
| GO:0001614 | purinergic nucleotide receptor activity | 2/263 | 21/18368 | 0.035863934 | 0.245430293 | 0.211954256 | P2RY6/PTAFR | 2 |
| GO:0016502 | nucleotide receptor activity | 2/263 | 21/18368 | 0.035863934 | 0.245430293 | 0.211954256 | P2RY6/PTAFR | 2 |
| GO:0004601 | peroxidase activity | 3/263 | 51/18368 | 0.036519265 | 0.245808972 | 0.212281284 | ALOX5AP/PRDX1/PTGS1 | 3 |
| GO:0140678 | molecular function inhibitor activity | 5/263 | 128/18368 | 0.037087535 | 0.245808972 | 0.212281284 | ANXA2/ANXA4/CAMK2D/FRY/LRRK2 | 5 |
| GO:0048306 | calcium-dependent protein binding | 4/263 | 88/18368 | 0.037633285 | 0.245808972 | 0.212281284 | A2M/ANXA2/ANXA4/S100P | 4 |
| GO:0005201 | extracellular matrix structural constituent | 6/263 | 172/18368 | 0.037650318 | 0.245808972 | 0.212281284 | CHI3L1/COL9A2/COLQ/ECM1/HSPG2/MFAP4 | 6 |
| GO:0005126 | cytokine receptor binding | 8/263 | 271/18368 | 0.041868531 | 0.259664839 | 0.224247248 | CCL5/CD300LF/CXCL10/ECM1/ITGA5/PRLR/TGFBR2/VEGFA | 8 |
| GO:0035091 | phosphatidylinositol binding | 8/263 | 271/18368 | 0.041868531 | 0.259664839 | 0.224247248 | ANXA2/GSN/IQGAP2/MYO10/NCF1/NCF1B/NCF1C/TWF1 | 8 |
| GO:0008483 | transaminase activity | 2/263 | 23/18368 | 0.042411304 | 0.259664839 | 0.224247248 | GPT2/PSAT1 | 2 |
| GO:0016702 | oxidoreductase activity, acting on single donors with incorporation of molecular oxygen, incorporation of two atoms of oxygen | 2/263 | 23/18368 | 0.042411304 | 0.259664839 | 0.224247248 | ALOX5AP/HPD | 2 |
| GO:0017116 | single-stranded DNA helicase activity | 2/263 | 23/18368 | 0.042411304 | 0.259664839 | 0.224247248 | MCM6/RFC3 | 2 |
| GO:0051213 | dioxygenase activity | 4/263 | 92/18368 | 0.043174391 | 0.259664839 | 0.224247248 | ALOX5AP/HPD/JMJD1C/PTGS1 | 4 |
| GO:0005080 | protein kinase C binding | 3/263 | 55/18368 | 0.044150509 | 0.259664839 | 0.224247248 | HDAC9/SRC/TOP2A | 3 |
| GO:0016684 | oxidoreductase activity, acting on peroxide as acceptor | 3/263 | 55/18368 | 0.044150509 | 0.259664839 | 0.224247248 | ALOX5AP/PRDX1/PTGS1 | 3 |
| GO:0072341 | modified amino acid binding | 4/263 | 93/18368 | 0.044627631 | 0.259664839 | 0.224247248 | ANXA2/CD300LF/GAS6/TREM2 | 4 |
| GO:0016701 | oxidoreductase activity, acting on single donors with incorporation of molecular oxygen | 2/263 | 24/18368 | 0.045839399 | 0.259664839 | 0.224247248 | ALOX5AP/HPD | 2 |
| GO:0016769 | transferase activity, transferring nitrogenous groups | 2/263 | 24/18368 | 0.045839399 | 0.259664839 | 0.224247248 | GPT2/PSAT1 | 2 |
| GO:0004713 | protein tyrosine kinase activity | 5/263 | 136/18368 | 0.04611352 | 0.259664839 | 0.224247248 | CSF1R/EPHB4/FGFR3/KIT/SRC | 5 |
| GO:0004869 | cysteine-type endopeptidase inhibitor activity | 3/263 | 56/18368 | 0.046172797 | 0.259664839 | 0.224247248 | CST3/CSTA/GAS6 | 3 |
| GO:0051287 | NAD binding | 3/263 | 56/18368 | 0.046172797 | 0.259664839 | 0.224247248 | ALDH2/ME1/PHGDH | 3 |
| GO:0035925 | mRNA 3'-UTR AU-rich region binding | 2/263 | 25/18368 | 0.049365646 | 0.27489889 | 0.237403415 | RBM24/ZFP36L2 | 2 |

- **Supplementary Table S11. KEGG pathway enrichment of PSPC1-specific DEGs.**

KEGG pathways uniquely enriched in PSPC1 knockdown.

| **ID** | **Description** | **GeneRatio** | **BgRatio** | **pvalue** | **p.adjust** | **qvalue** | **geneID** | **Count** |
| --- | --- | --- | --- | --- | --- | --- | --- | --- |
| hsa04145 | Phagosome | 14/160 | 159/8535 | 1.50347E-06 | 0.000357693 | 0.000296692 | ATP6V0A1/ATP6V1C2/C3/CD36/CYBB/DYNC1I1/FCGR2A/FCGR2B/FCGR2C/ITGA5/ITGB2/NCF1/THBS4/TLR4 | 14 |
| hsa04380 | Osteoclast differentiation | 13/160 | 143/8535 | 2.52787E-06 | 0.000357693 | 0.000296692 | CSF1R/FCGR2A/FCGR2B/FCGR2C/JUN/JUNB/LILRB2/LILRB4/NCF1/PIK3R3/TGFBR2/TREM2/TYROBP | 13 |
| hsa05140 | Leishmaniasis | 9/160 | 79/8535 | 1.53018E-05 | 0.001443474 | 0.001197303 | C3/CYBB/FCGR2A/FCGR2C/ITGA4/ITGB2/JUN/NCF1/TLR4 | 9 |
| hsa05205 | Proteoglycans in cancer | 14/160 | 204/8535 | 2.71563E-05 | 0.001921306 | 0.001593644 | CAMK2D/CTTN/GPC3/HSPG2/ITGA5/MMP2/MMP9/MYC/PIK3R3/PTCH1/SDC2/SRC/TLR4/VEGFA | 14 |
| hsa05219 | Bladder cancer | 6/160 | 41/8535 | 0.000103232 | 0.005154096 | 0.004275111 | FGFR3/MMP2/MMP9/MYC/SRC/VEGFA | 6 |
| hsa04933 | AGE-RAGE signaling pathway in diabetic complications | 9/160 | 101/8535 | 0.000109274 | 0.005154096 | 0.004275111 | CYBB/EGR1/F3/JUN/MMP2/PIK3R3/PLCB2/TGFBR2/VEGFA | 9 |
| hsa05417 | Lipid and atherosclerosis | 13/160 | 216/8535 | 0.000200866 | 0.008120722 | 0.006735804 | CAMK2D/CCL5/CD36/CYBB/JUN/MMP9/NCF1/PIK3R3/PLCB2/RAP1B/SRC/TLR4/XBP1 | 13 |
| hsa04512 | ECM-receptor interaction | 8/160 | 89/8535 | 0.000249575 | 0.008267024 | 0.006857156 | CD36/COL9A2/HSPG2/ITGA4/ITGA5/ITGA6/ITGB7/THBS4 | 8 |
| hsa04613 | Neutrophil extracellular trap formation | 12/160 | 193/8535 | 0.000262909 | 0.008267024 | 0.006857156 | AZU1/C3/CYBB/FCGR2A/HDAC9/ITGB2/NCF1/PADI4/PIK3R3/PLCB2/SRC/TLR4 | 12 |
| hsa05418 | Fluid shear stress and atherosclerosis | 10/160 | 141/8535 | 0.000303919 | 0.00860092 | 0.007134109 | ASS1/HMOX1/JUN/MMP2/MMP9/NCF1/PIK3R3/SDC2/SRC/VEGFA | 10 |
| hsa05323 | Rheumatoid arthritis | 8/160 | 95/8535 | 0.000390672 | 0.010050926 | 0.008336829 | ATP6V0A1/ATP6V1C2/CCL5/CD86/ITGB2/JUN/TLR4/VEGFA | 8 |
| hsa04640 | Hematopoietic cell lineage | 8/160 | 100/8535 | 0.000552688 | 0.013034224 | 0.010811352 | CD1D/CD36/CSF1R/FCER2/ITGA4/ITGA5/ITGA6/KIT | 8 |
| hsa05142 | Chagas disease | 8/160 | 103/8535 | 0.000673508 | 0.014661741 | 0.01216131 | C3/CCL5/GNA11/JUN/PIK3R3/PLCB2/TGFBR2/TLR4 | 8 |
| hsa04926 | Relaxin signaling pathway | 9/160 | 130/8535 | 0.000726006 | 0.014675689 | 0.012172879 | JUN/MMP2/MMP9/PIK3R3/PLCB2/RXFP1/SRC/TGFBR2/VEGFA | 9 |
| hsa04978 | Mineral absorption | 6/160 | 61/8535 | 0.000936453 | 0.017667751 | 0.014654672 | FTH1/HMOX1/MT1E/MT1G/MT1X/MT2A | 6 |
| hsa04820 | Cytoskeleton in muscle cells | 12/160 | 232/8535 | 0.001355553 | 0.023273465 | 0.019304381 | COL9A2/HSPG2/ITGA4/ITGA5/ITGA6/ITGB7/LMNA/MYBPH/SDC2/SYNPO2/THBS4/TMOD2 | 12 |
| hsa04670 | Leukocyte transendothelial migration | 8/160 | 116/8535 | 0.001466215 | 0.023273465 | 0.019304381 | CYBB/ITGA4/ITGB2/MMP2/MMP9/NCF1/PIK3R3/RAP1B | 8 |
| hsa04510 | Focal adhesion | 11/160 | 203/8535 | 0.001480291 | 0.023273465 | 0.019304381 | COL9A2/ITGA4/ITGA5/ITGA6/ITGB7/JUN/PIK3R3/RAP1B/SRC/THBS4/VEGFA | 11 |
| hsa05415 | Diabetic cardiomyopathy | 11/160 | 205/8535 | 0.001601216 | 0.023849689 | 0.019782334 | CAMK2D/CD36/CYBB/MMP2/MMP9/NCF1/NDUFA4L2/PIK3R3/PLCB2/TBC1D4/TGFBR2 | 11 |
| hsa04912 | GnRH signaling pathway | 7/160 | 93/8535 | 0.0017524 | 0.024796459 | 0.020567641 | CAMK2D/EGR1/GNA11/JUN/MMP2/PLCB2/SRC | 7 |
| hsa04015 | Rap1 signaling pathway | 11/160 | 212/8535 | 0.002089893 | 0.028099396 | 0.023307291 | CSF1R/FGFR3/FYB1/ITGB2/KIT/PIK3R3/PLCB2/RAP1B/RASGRP2/SRC/VEGFA | 11 |
| hsa05152 | Tuberculosis | 10/160 | 182/8535 | 0.002184405 | 0.028099396 | 0.023307291 | ATP6V0A1/C3/CAMK2D/FCGR2A/FCGR2B/FCGR2C/ITGB2/KSR1/SRC/TLR4 | 10 |
| hsa05144 | Malaria | 5/160 | 50/8535 | 0.002333274 | 0.028709412 | 0.023813274 | CD36/ITGB2/SDC2/THBS4/TLR4 | 5 |
| hsa05150 | Staphylococcus aureus infection | 7/160 | 102/8535 | 0.002969802 | 0.034886584 | 0.028936984 | C3/CFH/FCGR2A/FCGR2B/FCGR2C/ITGB2/PTAFR | 7 |
| hsa05146 | Amoebiasis | 7/160 | 103/8535 | 0.00313724 | 0.034886584 | 0.028936984 | CD1D/GNA11/ITGB2/PIK3R3/PLCB2/PRDX1/TLR4 | 7 |
| hsa04151 | PI3K-Akt signaling pathway | 15/160 | 362/8535 | 0.003205128 | 0.034886584 | 0.028936984 | COL9A2/CSF1R/FGFR3/ITGA4/ITGA5/ITGA6/ITGB7/KIT/MYC/PCK2/PIK3R3/PRLR/THBS4/TLR4/VEGFA | 15 |
| hsa05100 | Bacterial invasion of epithelial cells | 6/160 | 78/8535 | 0.003337617 | 0.034983168 | 0.029017097 | CTTN/DNM1/ITGA5/PIK3R3/SEPTIN11/SRC | 6 |
| hsa05202 | Transcriptional misregulation in cancer | 10/160 | 198/8535 | 0.004013983 | 0.0405699 | 0.03365106 | BCL2A1/CD86/CSF1R/ITGB7/JMJD1C/MMP9/MYC/PAX5/TGFBR2/ZEB1 | 10 |
| hsa05135 | Yersinia infection | 8/160 | 138/8535 | 0.004333759 | 0.042291507 | 0.035079063 | FCGR2A/FYB1/ITGA4/ITGA5/JUN/PIK3R3/SRC/TLR4 | 8 |
| hsa04066 | HIF-1 signaling pathway | 7/160 | 110/8535 | 0.004519995 | 0.042638624 | 0.035366982 | CAMK2D/CYBB/HMOX1/PIK3R3/TIMP1/TLR4/VEGFA | 7 |
| hsa04216 | Ferroptosis | 4/160 | 42/8535 | 0.007652274 | 0.067731008 | 0.056180081 | CYBB/FTH1/GCLM/HMOX1 | 4 |
| hsa04360 | Axon guidance | 9/160 | 184/8535 | 0.00765863 | 0.067731008 | 0.056180081 | CAMK2D/EPHB4/PIK3R3/PTCH1/SEMA4A/SEMA4C/SEMA6A/SEMA6B/SRC | 9 |
| hsa05221 | Acute myeloid leukemia | 5/160 | 68/8535 | 0.008772104 | 0.075227441 | 0.062398063 | BCL2A1/CSF1R/KIT/MYC/PIK3R3 | 5 |
| hsa04611 | Platelet activation | 7/160 | 126/8535 | 0.009362925 | 0.077932582 | 0.064641867 | FCGR2A/PIK3R3/PLCB2/PTGS1/RAP1B/RASGRP2/SRC | 7 |
| hsa04062 | Chemokine signaling pathway | 9/160 | 193/8535 | 0.010311278 | 0.07873246 | 0.065305333 | CCL5/CXCL10/GRK6/NCF1/PIK3R3/PLCB2/RAP1B/RASGRP2/SRC | 9 |
| hsa05120 | Epithelial cell signaling in Helicobacter pylori infection | 5/160 | 71/8535 | 0.010478367 | 0.07873246 | 0.065305333 | ATP6V0A1/ATP6V1C2/CCL5/JUN/SRC | 5 |
| hsa05230 | Central carbon metabolism in cancer | 5/160 | 71/8535 | 0.010478367 | 0.07873246 | 0.065305333 | FGFR3/KIT/MYC/PIK3R3/SLC7A5 | 5 |
| hsa04666 | Fc gamma R-mediated phagocytosis | 6/160 | 99/8535 | 0.01057185 | 0.07873246 | 0.065305333 | FCGR2A/FCGR2B/GSN/MYO10/NCF1/PIK3R3 | 6 |
| hsa05161 | Hepatitis B | 8/160 | 163/8535 | 0.011469382 | 0.083226544 | 0.06903299 | HSPG2/JUN/MMP9/MYC/PIK3R3/SRC/TGFBR2/TLR4 | 8 |
| hsa04810 | Regulation of actin cytoskeleton | 10/160 | 232/8535 | 0.011810862 | 0.083561849 | 0.069311111 | FGFR3/GSN/IQGAP2/ITGA4/ITGA5/ITGA6/ITGB2/ITGB7/PIK3R3/SRC | 10 |
| hsa04142 | Lysosome | 7/160 | 133/8535 | 0.012383234 | 0.085474515 | 0.070897589 | ASAH1/ATP6V0A1/FUCA1/GM2A/GNS/SORT1/TPP1 | 7 |
| hsa03320 | PPAR signaling pathway | 5/160 | 76/8535 | 0.013804774 | 0.093017882 | 0.077154501 | CD36/FADS2/ME1/PCK2/SCD | 5 |
| hsa04966 | Collecting duct acid secretion | 3/160 | 28/8535 | 0.015022813 | 0.098496227 | 0.081698563 | ATP6V0A1/ATP6V1C2/CA2 | 3 |
| hsa05133 | Pertussis | 5/160 | 78/8535 | 0.015313901 | 0.098496227 | 0.081698563 | C3/ITGA5/ITGB2/JUN/TLR4 | 5 |
| hsa04620 | Toll-like receptor signaling pathway | 6/160 | 109/8535 | 0.016417699 | 0.103249087 | 0.085640865 | CCL5/CD86/CXCL10/JUN/PIK3R3/TLR4 | 6 |
| hsa01521 | EGFR tyrosine kinase inhibitor resistance | 5/160 | 80/8535 | 0.016929685 | 0.104154364 | 0.086391754 | FGFR3/GAS6/PIK3R3/SRC/VEGFA | 5 |
| hsa05131 | Shigellosis | 10/160 | 250/8535 | 0.019027725 | 0.114571195 | 0.095032087 | C3/CCL5/CTTN/ITGA5/JUN/PIK3R3/PLCB2/SEPTIN11/SRC/TLR4 | 10 |
| hsa04012 | ErbB signaling pathway | 5/160 | 86/8535 | 0.022444685 | 0.129629507 | 0.107522337 | CAMK2D/JUN/MYC/PIK3R3/SRC | 5 |
| hsa05412 | Arrhythmogenic right ventricular cardiomyopathy | 5/160 | 86/8535 | 0.022444685 | 0.129629507 | 0.107522337 | ITGA4/ITGA5/ITGA6/ITGB7/LMNA | 5 |
| hsa04668 | TNF signaling pathway | 6/160 | 119/8535 | 0.024189345 | 0.136028273 | 0.112829849 | CCL5/CXCL10/JUN/JUNB/MMP9/PIK3R3 | 6 |
| hsa04610 | Complement and coagulation cascades | 5/160 | 88/8535 | 0.024513929 | 0.136028273 | 0.112829849 | A2M/C3/CFH/F3/ITGB2 | 5 |
| hsa04662 | B cell receptor signaling pathway | 5/160 | 91/8535 | 0.027841876 | 0.151524055 | 0.125682962 | FCGR2B/JUN/LILRB2/LILRB4/PIK3R3 | 5 |
| hsa04014 | Ras signaling pathway | 9/160 | 238/8535 | 0.034825553 | 0.184905827 | 0.153371767 | CSF1R/FGFR3/KIT/KSR1/PIK3R3/RAP1B/RASGRP2/RASGRP4/VEGFA | 9 |
| hsa00360 | Phenylalanine metabolism | 2/160 | 16/8535 | 0.035282384 | 0.184905827 | 0.153371767 | ALDH3B1/HPD | 2 |
| hsa01522 | Endocrine resistance | 5/160 | 99/8535 | 0.038072992 | 0.192404585 | 0.159591677 | JUN/MMP2/MMP9/PIK3R3/SRC | 5 |
| hsa05410 | Hypertrophic cardiomyopathy | 5/160 | 99/8535 | 0.038072992 | 0.192404585 | 0.159591677 | ITGA4/ITGA5/ITGA6/ITGB7/LMNA | 5 |
| hsa05211 | Renal cell carcinoma | 4/160 | 70/8535 | 0.042011243 | 0.208582138 | 0.173010292 | JUN/PIK3R3/RAP1B/VEGFA | 4 |
| hsa04915 | Estrogen signaling pathway | 6/160 | 139/8535 | 0.046356733 | 0.225824329 | 0.187311979 | JUN/MMP2/MMP9/PIK3R3/PLCB2/SRC | 6 |
| hsa05414 | Dilated cardiomyopathy | 5/160 | 105/8535 | 0.047079984 | 0.225824329 | 0.187311979 | ITGA4/ITGA5/ITGA6/ITGB7/LMNA | 5 |
| hsa05165 | Human papillomavirus infection | 11/160 | 333/8535 | 0.048234403 | 0.227505602 | 0.188706525 | ATP6V0A1/ATP6V1C2/COL9A2/ITGA4/ITGA5/ITGA6/ITGB7/PIK3R3/TERT/THBS4/VEGFA | 11 |
